# *In Vivo* Tumorigenesis Was Observed after Injection of *In Vitro* Expanded Neural Crest Stem Cells Isolated from Adult Bone Marrow

**DOI:** 10.1371/journal.pone.0046425

**Published:** 2012-10-05

**Authors:** Sabine Wislet-Gendebien, Christophe Poulet, Virginie Neirinckx, Benoit Hennuy, James T. Swingland, Emerence Laudet, Lukas Sommer, Olga Shakova, Vincent Bours, Bernard Rogister

**Affiliations:** 1 Groupe Interdisciplinaire de Génoprotéomique appliquée (GIGA), Unit of Neurosciences, University of Liege, Liège, Belgium; 2 Groupe Interdisciplinaire de Génoprotéomique appliquée (GIGA), Unit of Human Genetics, University of Liege, Liège, Belgium; 3 Institute of Anatomy, University of Zurich, Zurich, Switzerland; 4 Groupe Interdisciplinaire de Génoprotéomique appliquée (GIGA), Unit of Development, Stem Cells and Regenerative Medicine, University of Liège, Liège, Belgium; 5 Department of Neurology, Centre Hospitalier Universitaire de Liège, Liège, Belgium; 6 GIGA Genomics Platform, University of Liege, Liège, Belgium; 7 Division of Experimental Medicine, Department of Clinical Neuroscience, King's College, London, United Kingdom; Instituto Butantan, Brazil

## Abstract

Bone marrow stromal cells are adult multipotent cells that represent an attractive tool in cellular therapy strategies. Several studies have reported that *in vitro* passaging of mesenchymal stem cells alters the functional and biological properties of those cells, leading to the accumulation of genetic aberrations. Recent studies described bone marrow stromal cells (BMSC) as mixed populations of cells including mesenchymal (MSC) and neural crest stem cells (NCSC). Here, we report the transformation of NCSC into tumorigenic cells, after *in vitro* long-term passaging. Indeed, the characterization of 6 neural crest-derived clones revealed the presence of one tumorigenic clone. Transcriptomic analyses of this clone highlighted, among others, numerous cell cycle checkpoint modifications and chromosome 11q down-regulation (suggesting a deletion of chromosome 11q) compared with the other clones. Moreover, unsupervised analysis such as a dendrogram generated after agglomerative hierarchical clustering comparing several transcriptomic data showed important similarities between the tumorigenic neural crest-derived clone and mammary tumor cell lines. Altogether, it appeared that NCSC isolated from adult bone marrow represents a potential danger for cellular therapy, and consequently, we recommend that phenotypic, functional and genetic assays should be performed on bone marrow mesenchymal and neural crest stem cells before *in vivo* use, to demonstrate whether their biological properties, after *ex vivo* expansion, remain suitable for clinical application.

## Introduction

Although the adult brain contains small numbers of stem cells in restricted areas, the central nervous system exhibits limited capacity of regenerating lost tissue. Therefore, cell replacement therapies of damaged brain have provided the basis for the development of potentially powerful new therapeutic strategies for a broad spectrum of human neurological diseases. In recent years, neurons and glial cells have been successfully generated from embryonic stem cells [1], induced pluripotent stem cells [2], mesenchymal stem cells [3, 4 retracted in 39], and adult neural stem cells [5]. There have also been extensive efforts made by researchers to develop stem cell-based brain transplantation therapies. The generation of neural cells from bone marrow is of important clinical interest as, beside the unlimited number of cells, those cells would allow autologous grafts. In the meantime, multipotent neural crest stem cells were discovered as a minor population of bone marrow cells [6]. The potential impact of those cells in regenerative medicine is significant [7], however, it is important to further characterize those cells with extensive proliferation both *in vivo* and *in vitro*. Indeed, recent studies report the contribution of BMSC in cancer formation and their possible capacity for spontaneous immortalization under long term *in vitro* culturing [8]–[10]. Moreover, as only a few NCSC are available in adult bone marrow, several passages are necessary to obtain a sufficient amount of cells [11].

To characterize the NCSC present in bone marrow, we isolated and cultivated 6 neural crest derived clones. These clones were first characterized *in vitro*, then, were injected into mice striatum to analyze their ability to survive and differentiate *in vivo*. One of those clones (*Asclepios*) had the highest ability to differentiate into neuronal cells (*in vitro*), and also showed a very high rate of proliferation after injection into mice striatum, when compared to the other clones. We therefore hypothesized that this abnormal proliferation was the result of the evolution of *Asclepios* into a tumoral clone.

To evaluate the tumorigenic potential of the *Asclepios* clone, we performed a whole genome mRNA expression assay on non-injected cells. We compared *Asclepios* to its direct NCSC reference (Mix of 5 NCSC clones), as well as to several tumor cell types and highlighted numerous similarities between the *Asclepios* clone and mammary tumor types. Additionally, we observed a deep modification of the cell cycle checkpoints in the Asclepios clone that may lead to uncontrolled proliferation. Likewise, chromosomal patterns of mRNA expression levels revealed blocks of differentially expressed chromosomal regions with a striking down regulation of the major part of the chromosome 11. Altogether, this report strongly highlights the prudence that should be taken in cellular therapy protocols when using adult bone marrow NCSC as previously suggested for MSC.

## Materials and Methods

### Animal care

*Wnt1-Cre/R26R*-LACZ double transgenic mice were used to confirm the presence of neural crest cells in adult bone marrow and to discriminate NCSC clones. Transgenic green fluorescent protein (GFP) C57BL/6J mice (The Jackson Laboratory, Bar Harbor, Maine) were used to produce cerebellar granule neurons (CGN) cultures. Likewise, wild type C57BL/6J mice (The Jackson Laboratory) were used as recipient mice for graft experiments. Rodents were bred at the University of Liège Central Animal facility and euthanized in accordance with the rules set by the local animal ethics committee as well as the Swiss Academy of Medical Sciences.

### Intrastriatal grafts

Animals were anesthetized with 100 mg/kg of a solution containing equal volumes of xylazine (Rompun) and ketamine (Ketalar). Mice were then placed into a stereotaxic frame (Benchmark, MyNeuroLab.com) and received one injection of 5×10^4^ cells suspended in 2 µL PBS (GIBCO, Invitrogen) in the right striatum (0,5 mm anterior, 2 mm lateral and 3 mm ventral, with respect to bregma). The intracerebral injection was performed using a Hamilton's 5 µl syringe, coupled with a 26-gauge needle. The needle was left in place for few minutes before being retracted, to avoid reflux along the injection track. After the operation, mice were placed under a warm lamp until their complete awakening.

### Brain processing

28 days after the cell transplantation, animals were deeply anesthetized and sacrificed by intracardiac perfusion of cold PBS, followed by paraformaldehyde (PFA) 4% (in 0,1 M PBS). Brains were immediately removed, post-fixed for 2 hours at 4°C in the same fixative and immersed overnight in a solution of sucrose 20% (in 0,1 M PBS). They were then rapidly frozen in isopentane and stored at −20°C. Coronal 14 µm-sections (containing the entirety of the striatum) were cut using a cryostat and mounted on positively charged slides and stored in −80°C for further experiments.

### Bone marrow stromal cell (BMSC) culture

Bone marrow cells from adult (8–10 week-old) mice were obtained from femoral and tibial bones by aspiration and were resuspended in 5 ml of MesenCult Medium (StemCells Technologies). After 24 hours, non-adherent cells were removed. When the BMSC became confluent, they were resuspended using 0.05% trypsin-EDTA (Invitrogen) and then cultured (750,000 cells/25 cm^2^).

### Preparation and culture of Mouse cerebellar granule neurons

Mouse cerebellar granule neuron (CGN) cultures were prepared from 3-day-old GFP or wild type C57BL/6J mice (The Jackson Laboratory) [3]. Green mice express green fluorescent protein (GFP) under control of the beta-actin promoter [3]. Briefly, cerebella were removed and freed of meninges. They were then minced into small fragments and incubated at 37°C for 25 minutes in 0.25% trypsin and 0.01% DNAse (w/v, in a cation-free solution). Fragments were then washed with minimum essential medium (Invitrogen) supplemented with glucose (final concentration 6 g 1^−1^), insulin (Sigma-Aldrich; 5 µg ml^−1^) and pyruvate (Invitrogen; 1 mM). The potassium concentration was increased to 25 mM, while the sodium concentration was decreased in an equimolar amount (MEM-25HS). The dissociation was achieved mechanically by up-and-down aspirations in a 5-ml plastic pipette. The resulting cell suspension was then filtered on a 15-µm nylon sieve. Cells were then counted and diluted to a final concentration of 2.5×10^6^ ml^−1^. The cell suspension was finally plated on a substrate previously coated with polyornithine (0.1 mg ml^−1^). The cells were cultured for 24 hours before any other experimental procedure was performed.

### Clonal selection

Passage 5 BMSC (from *Wnt1-Cre/R26R*-LACZ double transgenic mice) have been seeded in a 96 well plate (Nunc) at a dilution of 0.7 cell/well, in MesenCult Medium (Stem Cells Technologies). When cells reached confluence, they were dissociated with Trypsin-EDTA (0.05%) and cultured at 150,000 cells/ml.

### Immunofluorescence

Briefly, cell cultures were fixed with 4% PFA for 10 min at room temperature, then blocked with 10% normal donkey serum (NDS) for 45 min. Anti-Sox10 (1∶200; Affinity Bioreagents), anti-nestin (1∶300; Novus Biologicals), anti-betaIII-tubulin (1∶1000; Covance), anti-p75^NTR^ (1∶100; Millipore), anti-NrCAM (1∶400; Abcam), anti-N-Cadherin (1∶500, BD-Biosciences) and anti-E-Cadherin (1∶400, BD-Biosciences) were used overnight at 4°C. After four washes, cell cultures were incubated with FITC- or rhodamine-conjugated secondary antibodies (1∶500; Jackson Immunoresearch Laboratories) for 1 h at room temperature and finally, and finally mounted in Vectashield HardSet Mounting Medium with DAPI (Vector Laboratories). Preparations were observed using a Nikon TE 2000-U epifluorescent microscope (Nikon, Amstelveen, The Netherlands) or an Olympus laser scanning confocal microscope (Olympus, Tokyo, Japan). The digitized images were adjusted for brightness and contrast, color-coded, and merged, when appropriate, using the NIH program ImageJ or the Adobe Photoshop 6.0 program (Adobe Systems Incorporated, San Jose, CA). The fraction of positive cells was determined by analyzing 10 non-overlapping fields for each coverslip (with a minimum of 3 coverslips per experiment) in at least three separate experiments (n represents the number of experiments).

### Other Stainings

X-gal staining was performed on 2% PFA-fixed cells and on striatum slices (14 µm). Cells and sections were incubated for 2 hours in PBS supplemented with Tris (pH 7,4) 20 mM, MgCl_2_ 2 mM, 0.02% NP-40, 0.01% Na-deoxycholate, K_3_Fe(CN)_6_ 5 mM (Sigma-Aldrich), K_4_Fe(CN)_6_ 5 mM (Sigma-Aldrich) and 1-Methyl-3-indolyl-beta-D-galactopyranoside 1 mg/ml (Sigma–Aldrich) in DMSO. The reaction was stopped by PBS washes. Hematoxylin/eosin coloration. Dry brain sections were placed in denatured ethanol and slightly heated for approximately 4 minutes, then were washed three times in milliQ water, before an incubation of 10 minutes in Carazzi hematoxylin. After three washes in water, sections were incubated for 2 minutes in eosin. Once colored, sections were washed again in milliQ water for three times, dehydrated in successive alcohol solutions and finally mounted with Q Path Safemount (Labonord).

### Cell Proliferation Assay

Cell proliferation assay was performed using tetrazolium compound based CellTiter 96® AQ_ueous_ One Solution Cell Proliferation (MTS) assay (Promega). 5×10^3^ cells of each NCSC clone were seeded into wells of a 96-well plate. After 24 and 48 hours of culture under regular growth conditions (Mesencult medium), MTS assay was performed according to the manufacturer's instructions. Each experience was performed in triplicate and repeated 3 times (n = 3).

### RNA extraction, quality control and microarray experiments

Total RNA was prepared using the RNeasy total RNA purification kit (Qiagen) [11]. RNA quality was assessed by the Experion automated electrophoresis system using the RNA StdSens Analysis Kit (Bio-Rad). Four micrograms of total RNA were labeled using the GeneChip Expression 3′-Amplification One-Cycle Target Labeling Kit (Affymetrix) following the manufacturer's protocol. The cRNA was hybridized to GeneChip Mouse Genome 430 2.0 (Affymetrix) according to the manufacturer's protocol. Briefly, double-stranded cDNA was synthesized from 4 µg of total RNA primed with a poly-(dT)-T7 oligonucleotide. The cDNA was used in an *in vitro* transcription reaction in the presence of T7 RNA polymerase and biotin-labeled modified nucleotides for 16 h at 37°C. Biotinylated cRNA was purified and then fragmented (35–200 nucleotides) together with hybridization controls and hybridized to the microarrays for 16 h at 45°C. Using Fluidics Station (Affymetrix), the hybridized biotin-labeled cRNA was revealed by successive reactions with streptavidin R-phycoerythrin conjugate, biotinylated anti-streptavidin antibody and streptavidin R-phycoerythrin conjugate. The arrays were finally scanned with an Affymetrix/Hewlett-Packard GeneChip Scanner 3000 7G. The data were generated with the PLIER algorithm included in Affymetrix GeneChip Command Console Software (AGCC) and Expression Console.

### Microarray normalization and data filtering

Microarray normalization and data filtering were performed using BRB-ArrayTools software version 3.8.1 developed by Dr. Richard Simons and the BRB-ArrayTools Development Team, http://linus.nci.nih.gov./BRB-ArrayTools.html. We used the GCRMA algorithm as normalization step. Quartiles of each expression array were compared in a boxplot view. Medians, first and third quartiles were similar in each case (data not shown). This similarity allowed the comparison of the arrays under the same analysis process. Background noise has been removed with the “Log Intensity variation” function of “BRB-ArrayTools” at a *p*-value>0.05.

### Chromowave analysis of expression pattern

Spatial expression patterns were investigated using Chromowave [12] written in MATLAB 6.5 (The Mathworks Inc., Natick MA, USA). Briefly, mRNA expression values were log2 transformed and mapped to their corresponding chromosomal location using information from the Affymetrix NetAffx file for the MOE430_2 array. The mRNA expression values were then transformed into wavelet coefficients using the Haar wavelet transform. The wavelet transform is an orthogonal mathematical operator, with identical noise levels in the original data and at all wavelet levels. Wavelet coefficients are functions of the difference in expression of adjacent genes or clusters of genes. Clusters of genes with similar expression are therefore transformed into a wavelet coefficient whose the size depends on the number of genes represented in the cluster. Consequently, individual genes with expression below the noise level are identified when clustered together, but undetectable individually. For the Chromowave pattern analysis, singular value decomposition (SVD) was applied to the wavelet coefficients from all chromosomes. The profile associated with the case loadings was filtered using a conservative threshold that accounted for statistical noise, the number of wavelets and the probe–probe genomic distance, with the contribution of individual probes zeroed to only allow spatially extended patterns. After filtering, the remaining coefficients had the inverse wavelet transform applied to produce a spatial expression pattern for each chromosome associated with the primary genome-wide pattern of variations. Case loadings were analyzed using t-tests to verify the association with groupings. The *P*-values were then corrected for the number of multiple comparisons using the false discovery rate criterion fixed at 5%. For the cluster analysis the case loadings from the 3 first eigenvectors were calculated by applying SVD to all wavelet coefficients from all chromosomes.

### Class comparisons and pathway analysis

Class comparisons between *Asclepios* and NCSC clones were performed using BRB-ArrayTools with a significance threshold of 0.001, random variance and 10,000 permutations for univariate tests. The chromosome distribution was performed using BRB-ArrayTools and compares the percentage of genes for each chromosome between the 19,667 filtered genes and the 1,544 differentially expressed genes. To determine which pathways were significantly regulated, the 1,544 differentially expressed genes were uploaded into Ingenuity Pathway Analysis software (IPA 6.0; Ingenuity Systems).

### Hierarchical clustering

Clustering was generated in an unsupervised analysis after background noise filtering. This hierarchical cluster compares expression array data with several expression array data available from the Gene Expression Omnibus database (GEO) http://www.ncbi.nlm.nih.gov/geo/. All samples from GEO datasets have been processed on Affymetrix Mouse Genome Expression Set 430 GeneChips. The package “pvclust” [13] from R-cran [14] was used on the remaining filtered genes to build and test the architecture of each cluster of samples. “pvclust” is used for assessing the uncertainty in hierarchical cluster analysis. For each cluster in hierarchical clustering, p-values are calculated via multiscale bootstrap resampling. This indicates how strong the cluster is supported by data. “pvclust” provides two types of p-values: AU (Approximately Unbiased) p-value in red and BP (Bootstrap Probability) value in green. AU p-value, which is computed by multiscale bootstrap resampling, is a better approximation to unbiased p-value than BP value computed by normal bootstrap resampling. We choose the most commonly used “Euclidean distance” as dissimilarity metric and 3 different methods of linkage (single, complete, average) to obtain dendrogram structures. The relevance of dendrograms architecture was then tested by data permutations. We set the multiscale boostrap resampling argument of “pvclust” at 10,000 permutations of genes to test those dendrograms. Only the “complete linkage” method showed the best stable structure. Microarray results from *Asclepios* and NCSC clones are accessible on GEO datasets/NCBI (http://www.ncbi.nlm.nih.gov/gds).

## Results

### 1. Isolation and characterization of neural-crest derived clones from adult bone marrow

**Figure 1:**
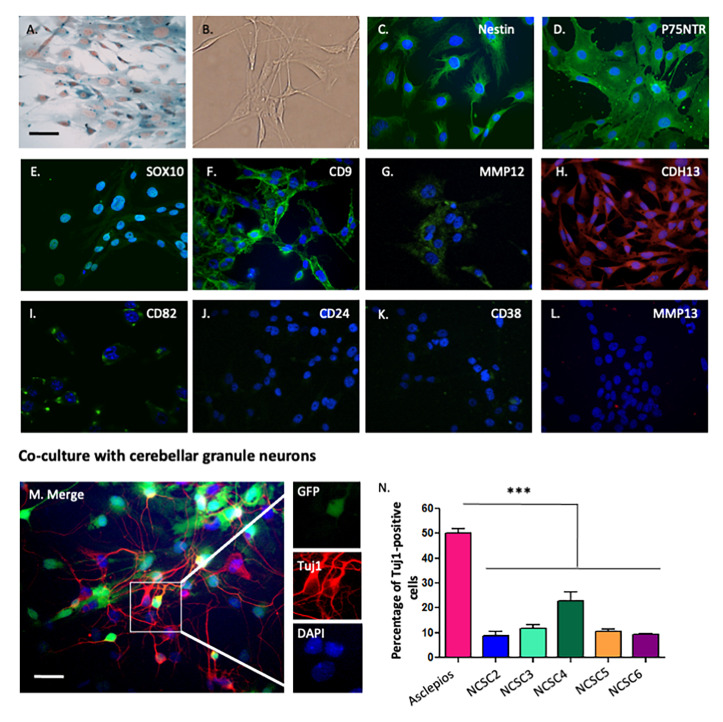
Phenotypic characterization of neural-crest derived cells isolated from adult bone marrow. Neural crest stem cells were isolated from double transgenic Wnt1/Cre-R26R/LacZ mice and cultured under clonal conditions. **A–B**. Neural crest derived clones were morphologically similar to classical BMSC. As clones have been isolated from double transgenic mice Wnt1-CRE/R26R-LacZ, neural crest-derived cells are expressing beta-galactosidase, visualized after an X-gal staining (A). **C–L**. Immunological characterization revealed that neural crest derived cells were nestin (C), P75^NTR^ (D), Sox10 (E), CD9 (F), MMP12 (G), CDH13 (H), CD82 (I) positives, but CD24 (J), CD38 (K) and MMP13 (L) negatives. **M–N**. A percentage of neural crest stem cells were able to differentiate into beta-III-tubulin-positive cells when co-cultivated with GFP-positive cerebellar granule neurons (M), however, *Asclepios* showed a higher percentage of positive cells as 50.25%±1.70% of cells were beta-III-tubulin-positive, when around 15% of cells were observed with the other clones (N) (mean ± SEM, *n* = 3, *p*<0.001, ANOVA followed by Bonferroni *post hoc* test). Nuclei were counterstained with Dapi (blue) on panels C to N. Scale bars = 30 µm.

Since 2008, several studies reported the presence of NCSC in adult bone marrow [6]–[7], [11]. We cultured BMSC from adult Wnt1-cre/R26R-LacZ mice under clonal conditions to selectively isolate NCSC-derived clones. After 5 passages, BMSC were placed in 96 well plates at a density of 0.7 cells per well. Around 2% of cells were able to proliferate under those conditions and 6 neural crest derived clones were maintained for further characterization. NCSC were discriminated among other cells as they expressed beta-galactosidase (Fig. 1A). NCSC were morphologically similar to classical bone marrow mesenchymal cells (Fig. 1B). As no specific neural crest markers have been described so far to discriminate NCSC from MSC, we used a panel of markers to characterize those cells. As observed on Figure 1, NCSC clones were nestin+ (Fig. 1C), P75^NTR^+ (Fig. 1D), Sox10+ (Fig. 1E), CD9+ (Fig. 1F), MMP12+ (Fig. 1G), CDH13+ (Fig. 1H), and CD82+ (Fig. 1I), but CD24− (Fig. 1J), CD38− (Fig. 1K) and MMP13− (Fig. 1L).

### 2. In vitro characterization of NCSC neuronal differentiating capacities

As one of our interests to isolate NCSC from adult bone marrow was their potential use in brain regenerative medicine, we decided first to characterize their ability to differentiate into neurons when co-cultivated with cerebellar granule neurons (CGN), as previously described in Wislet-Gendebien et al. [3], [7]. Consequently, we co-cultivated each clone with GFP-positive CGN. As observed on Figure 1. (M–N), different proportions of beta-III-tubulin-positive cells were obtained from clones: NCSC1 (referenced as *Asclepios*): 50.25%±1.70%; NCSC2: 8.68%±1.94%; NCSC3: 11.80%±1.31%; NCSC4: 22.82%±3.54%; NCSC5: 10.59%±0.95% and NCSC6: 11.29%±1.55% of beta-III-tubulin-positive cells, suggesting that some subgroups may exist among NCSC clones (*n* = 3, *p*<0.001), ANOVA followed by Bonferroni *post hoc* test).

### 3. *Asclepios* induces tumor formation when injected into mouse striatum

**Figure 2:**
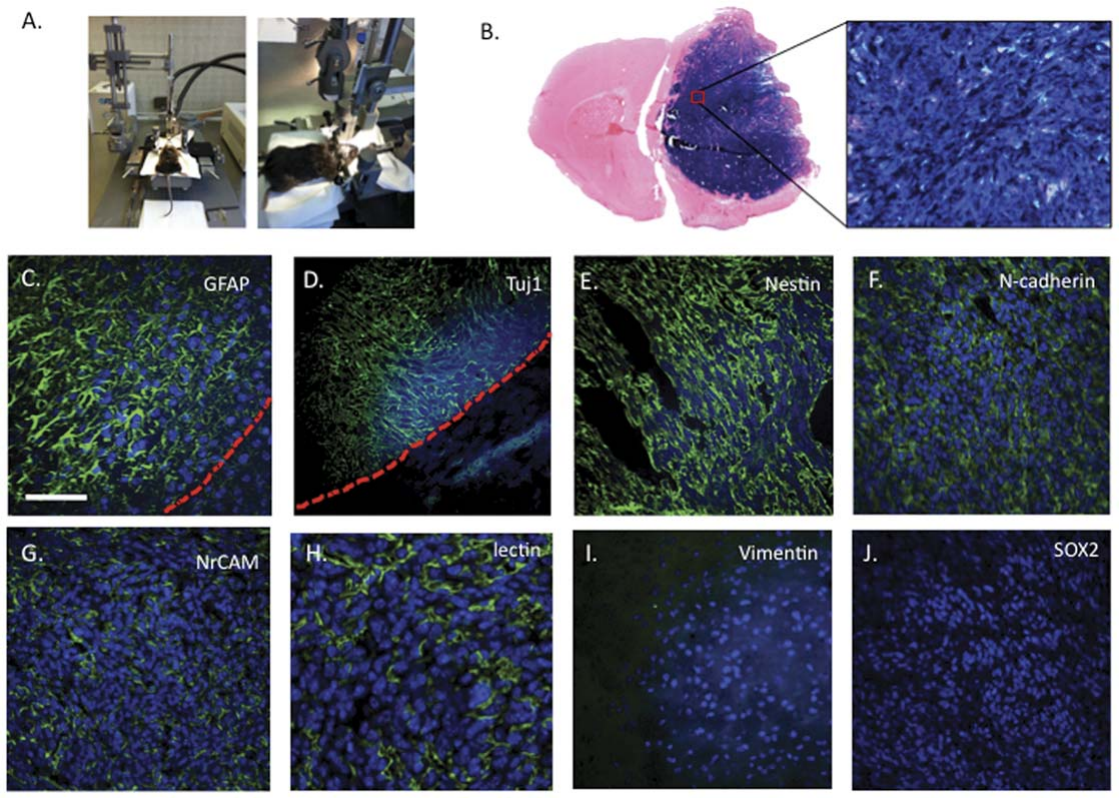
*In vivo* characterization of neural crest derived cells. To characterize neural crest-derived clones *in vivo*, we stereotaxically injected 50,000 cells of each NCSC clones (separetly) in mice striatum (**A**). *Asclepios* induced massive tumors after 4 weeks as attested by the beta-galactosidase expression of the tumor cells. (**B**). Immunological characterization of those tumors revealed that they were GFAP (**c**), beta-III-tubulin (**D**), nestin (**E**), N-cadherin (**F**) and NrCAM-positive (G). Lectin labeling (**H**) confirmed the presence of blood vessels in the tumors. Finally, no vimentin (**I**) or Sox2 (**J**) expressions were observed. Nuclei were counterstained with Dapi (blue). Scale bars = 50 µm.

As all clones were able to differentiate into beta-III-tubulin-positive cells, we decided to investigate their ability to differentiate into neurons and survive when injected into adult mouse striatum. Therefore, 50,000 cells were stereotaxically injected into the right striatum (coordinate 0.5 mm before bregma, 2 mm right, 3 mm deep, Fig. 2A) of healthy mice. Surprisingly, *Asclepios* induced massive tumors after 4 weeks (Fig. 2B), whereas really few cells remained after injection of the other clones (data not shown). Immunohistological analysis of the tumors revealed the presence of GFAP (Fig. 2C), beta-III-tubulin (Fig. 2D), nestin (Fig. 2E), N-cadherin (Fig. 2F) and NrCAM positive cells (Fig. 2G). However, no vimentin (found in glial-derived tumors- Fig. 2I), or Sox2 positive cells (embryonic stem cell marker expressed in several brain tumors - Fig. 2J) were observed. Moreover, labeling tumor sections with lectin (which binds oligosaccharides on the membrane of endothelial cells) specifically revealed a positive staining within the tumor mass, therefore indicating a vascularization of the tumors (Fig. 2H).

### 4. Validation of NCSC mix as a reference for *Asclepios* analyses

**Figure 3:**
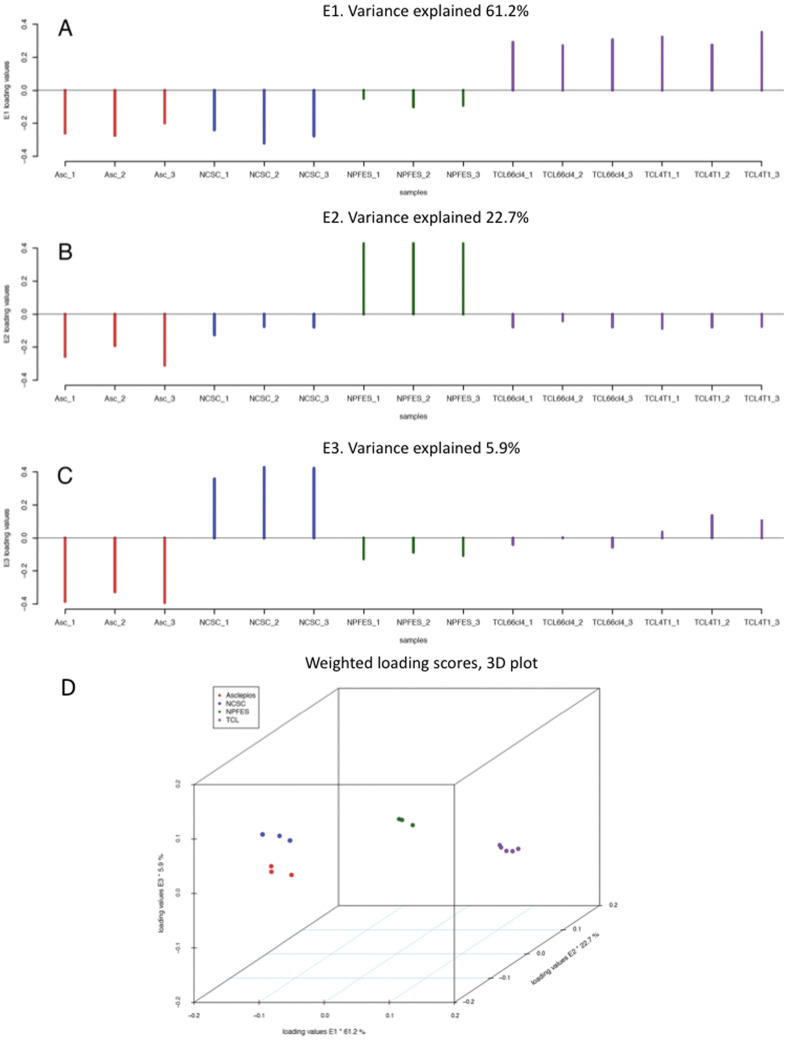
Clusters based on wavelet coefficients. The first, second and third components are represented in figure A, B and C respectively and summarized on figure D. These components respectively explain 61.2, 22.7 and 5.9% of the variance of the dataset. For each component, samples in the same orientation over the y-axis are clustered together. *Asclepios* (Asc); Neural Crest Stem Cells mix (NCSC); Tumor cell lines 67NR, 66cl4 and 4T1 (TCL, GSE11259); Neural Precursor From Embryonic Stem Cells (NPFES, GSE8024) are represented in these clusters. The difference between *Asclepios* and NCSC represents only 5.9% of the variance, making NCSC the best reference to study *Asclepios* mRNA expression.

To understand the molecular basis of the tumorigenic properties of Asclepios, we decided to compare the gene expression profile of Asclepios with the one of a reference clone, which did not show aberrant proliferation and tumorigenic properties. As all of our NCSC clones share the same origin (from adult BM), as well as the same immunological and functional characteristics (except *Asclepios)*, we decided to use a mix of NCSC2, NCSC3, NCSC4, NCSC5 and NCSC6 clones as a reference. To validate this set as a suitable reference, we first compared Microarray data from *Asclepios* and NCSC to the expression profile of other cell types selected from GEO database (Tumor Cell Line (TCL) -GSE11259 and Neural Precursor From Embryonic Stem cells (NPFES) - GSE8024). We analyzed the deep variation of mRNA expression levels for these datasets. Using CHROMOWAVE software, the three first eigenvector components were analyzed and 61.2% of the global variance was explained by the difference between TCL and the rest (Fig. 3A and 3D), 22.7% of the global variance was explained by the difference between NPFES and the rest (Fig. 3B and 3D), and only 5.9% of the global variance was explained by the difference between *Asclepios* and NCSC (Fig. 3C and 3D). These results highlighted the genetic similarities between *Asclepios* and NCSC clones, making the NCSC mix a good reference to study *Asclepios*.

### 5. Chromosome 11 as one of the major modifications in *Asclepios*

**Figure 4:**
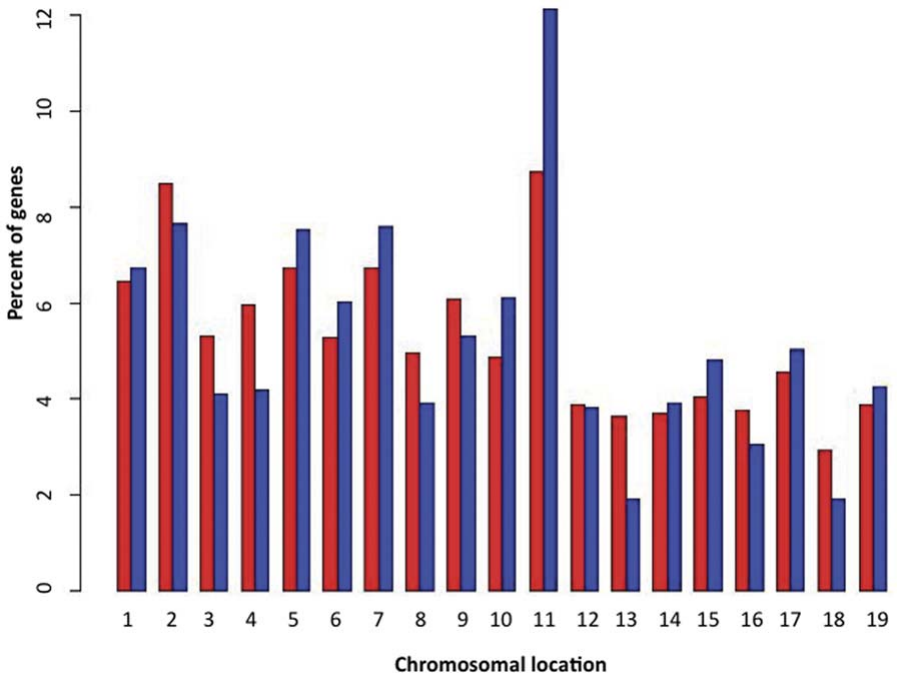
Chromosomal distribution of *Asclepios* genes. Barplot comparing the chromosomal distribution of the differentially expressed genes (*p*-value<0.001–1,544 probesets) in blue to the overall background filtered dataset (19,667 probesets) in red. This barplot, based on the comparison between *Asclepios* and NCSCs, highlights the chromosome 11 enrichment after statistical univariate tests.

**Figure 5:**
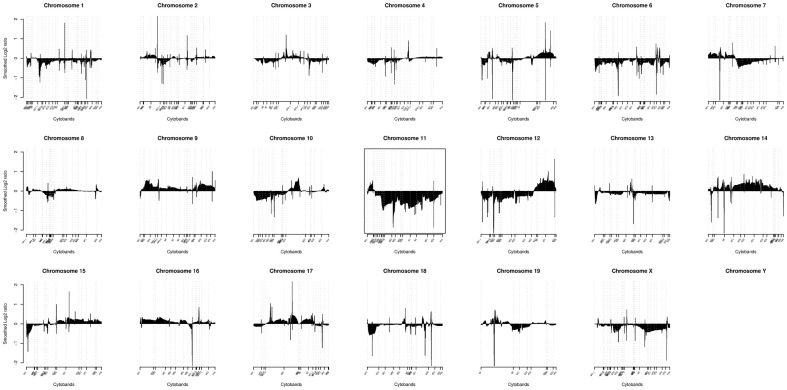
Chromowave profile of *Asclepios*, displays the first eigenvector that explains 84% of the variance between *Asclepios* and NCSC mix. Each chromosomal signal has been wavelet transformed. Under and over expressions are respectively below or above 0 on the y-axis. The x-axis displays the chromosomal position labeled with cytoband names. A large part of the chromosome 11 is under expressed in *Asclepios*.

To assess whether the malignant transformation of *Asclepios* is associated with the deregulation of a particular chromosome, we performed an mRNA expression microarray comparison between *Asclepios* and the NCSC mix. The class comparison revealed 1,544 differentially expressed genes (*p*-value<0.001, supplementary data - Table S1). Interestingly, an enrichment of chromosome 11 genes was observed in these 1,544 relevant genes compared to the 19,667 genes from the background-filtering step (Fig. 4). To further identify the spatial level of expression of the chromosome 11 genes, a chromosomal pattern was generated with CHROMOWAVE. Eigenvectors were computed and 84% of the variance of the dataset was explained by the first component. The expression pattern of this component is shown in figure 5. Surprisingly, the major part of chromosome 11 showed a low level of expression in *Asclepios*. Even if the chromosome 11 deletion is well described in several cancers [15]–[18], its genes may be involved in many biological functions. Therefore, an independent pathway analysis was performed using Ingenuity Pathway Analysis (IPA), taking all chromosome 11 genes (probe-sets) extracted from the UCSC genome browser [19] and without considering the expression levels from our data. Interestingly, chromosome 11 genes are strongly involved in cancer functions (*p*-value = 1e^−23^) such as neoplasia (*p*-value = 1e^−23^) or tumorigenesis (*p*-value = 3.3e^−23^) as described in Table 1. Nevertheless, only a part of the chromosome 11 genes are relevant (p-value<0.001) in our study. Therefore, the same analysis was performed based on the 165 significant genes of the chromosome 11 that are differentially expressed in *Asclepios* (p-value<0.001) compared to the NCSC mix. These genes are involved in cancer functions (p-value = 1.5e^−4^) and also in the cancer signaling pathway PI3K/AKT (p-value = 3.06e^−3^). Combined together, these results highlight the importance of the instability of the chromosome 11 in tumorigenesis and explain a lot about the aberrant expansion of *Asclepios* in mouse striatum.

**Table 1:** Gene type expression on chromosome 11.

*Category*	*Functions Annotation*	*p-Value*
Cancer	neoplasia	1.00E−23
Cancer	cancer	2.44E−23
Cancer	tumorigenesis	3.30E−23
Cancer	solid tumor	4.74E−18
Cancer	carcinoma	1.13E−17
Cancer	digestive organ tumor	2.21E−11
Cancer	gastrointestinal tract cancer	6.86E−10
Cancer	breast cancer	2.64E−09
Cancer	cell transformation	6.30E−09
Cancer	mammary tumor	1.19E−08
Cancer	colorectal tumor	1.59E−08
Cancer	transformation	2.10E−08
Cancer	colorectal cancer	2.29E−08
Cancer	hematological neoplasia	3.64E−07
Cancer	head and neck cancer	9.95E−07
Cancer	genital tumor	1.28E−06
Cancer	prostate cancer	1.71E−06
Cancer	prostatic tumor	2.58E−06
Cancer	metastasis	3.85E−06
Cancer	malignant glioma	4.29E−06
Cancer	tumorigenesis of malignant tumor	7.33E−06
Cancer	transformation of fibroblast cell lines	9.45E−06
Cancer	glioblastoma	1.08E−05
Cancer	glioma	1.17E−05
Cancer	tumorigenesis of tumor cell lines	1.46E−05
Cancer	neuroepithelial tumor	2.19E−05
Cancer	astrocytoma	3.06E−05
Cancer	central nervous system tumor	3.49E−05
Cancer	hematologic cancer	4.30E−05
Cancer	liver tumor	6.91E−05
Cancer	tumorigenesis of cells	9.26E−05
Cancer	tumorigenesis of blood tumor	1.26E−04
Cancer	endocrine gland tumor	1.34E−04
Cancer	liver cancer	1.36E−04
Cancer	tumorigenesis of lymphoma	1.73E−04
Cancer	benign tumor	2.10E−04
Cancer	thyroid cancer	2.22E−04
Cancer	brain cancer	2.45E−04
Cancer	metastatic colorectal cancer	3.77E−04
Cancer	transformation of fibroblasts	3.96E−04
Cancer	melanoma	5.26E−04
Cancer	carcinoma in situ	5.75E−04
Cancer	lymphoid cancer	6.21E−04
Cancer	renal cancer	7.97E−04
Cancer	uterine cancer	8.33E−04
Cancer	leukemia	8.88E−04
Cancer	renal tumor	1.11E−03
Cancer	tumorigenesis of carcinoma	1.33E−03
Cancer	tumorigenesis of digestive organ tumor	1.40E−03
Cancer	polycystic ovary syndrome	1.56E−03
Cancer	lung tumor	2.53E−03
Cancer	myeloid leukemia	2.62E−03
Cancer	leiomyomatosis	2.69E−03
Cancer	plasma cell dyscrasia	2.78E−03
Cancer	stomach tumor	2.78E−03
Cancer	ductal carcinoma	3.00E−03
Cancer	infection of tumor cell lines	3.01E−03
Cancer	cancer of organ	3.12E−03
Cancer	metastasis of tumor	3.14E−03
Cancer	colon cancer	4.39E−03
Cancer	colon tumor	4.61E−03
Cancer	infection of cervical cancer cell lines	4.87E−03
Cancer	lung cancer	5.17E−03
Cancer	malignant lymphocytic neoplasm	5.28E−03
Cancer	lymphomagenesis	5.60E−03
Cancer	pancreatic tumor	5.65E−03

Pathway analysis was performed on chromosome 11 genes (probe-sets), using Ingenuity Pathway Analysis (IPA) and without considering the expression levels from our data. As shown, chromosome 11 genes are strongly involved in cancer functions (*p*-value = 1e^−23^) such as neoplasia (*p*-value = 1e^−23^) or tumorigenesis (*p*-value = 3.3e^−23^).

### 6. Significant genes revealed the tumor phenotype of *Asclepios*

**Figure 6:**
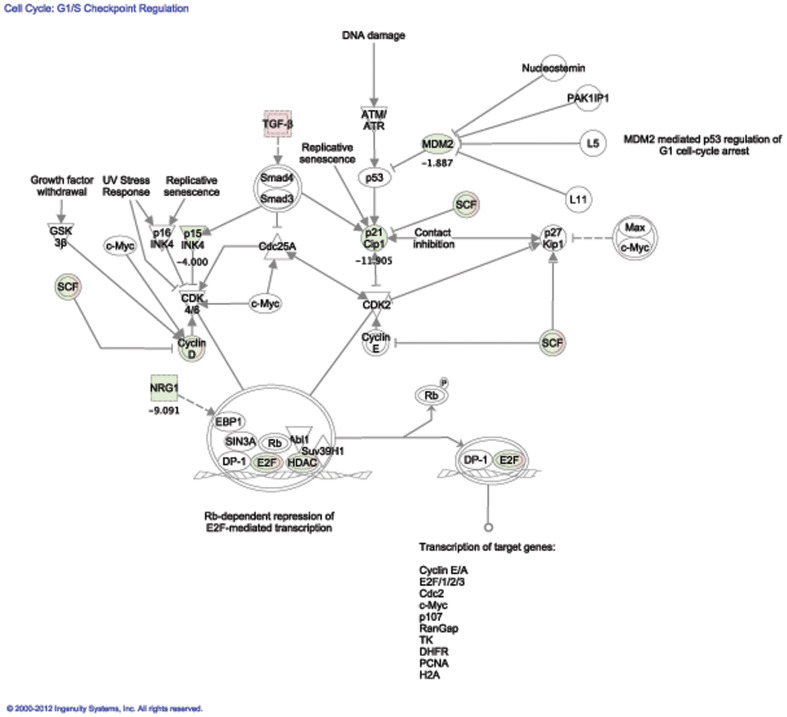
Cell cycle: G1/S checkpoints. G1/S checkpoints regulation pathway highlights regulated genes in the comparison between *Asclepios* and NCSC mix. Red genes are over-expressed and Green genes are down-expressed in *Asclepios*. Fold changes from the comparison are written for single (non complex) genes. This pathway was build with Ingenuity Pathway Analysis software. Major tumor suppressors are down regulated suggesting a high proliferation of *Asclepios* cells.

**Figure 7:**
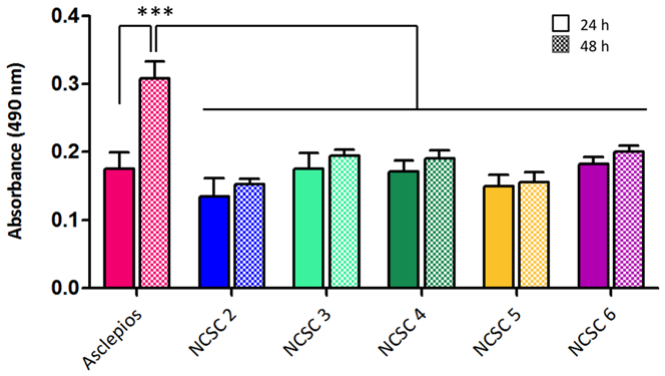
MTS Cell Proliferation Assay. Cell proliferation assay was performed using tetrazolium compound based CellTiter 96® AQ_ueous_ One Solution Cell Proliferation (MTS) assay (Promega). 5×10^3^ cells of each NCSC clone were seeded into wells of a 96-well plate. After 24 and 48 hours of culture under regular growth conditions (Mesencult medium), MTS assay was performed according to the manufacturer's instructions. Each experience was performed in triplicate and repeated 3 times (n = 3). No difference was observed after 24 hours of culture, however, a highly significant increase in absorbance was detected for *Asclepios* in comparison with the other clones (p<0,001; repeated measures ANOVA, followed by Tuckey post-test), reflecting a higher proliferation rate in an interval of 48 hours.

Even if the low level of expression of many genes located on the chromosome 11 could mainly explain the tumor profile of *Asclepios*, we decided to analyze differentially expressed genes located on all chromosomes when comparing the gene expression profiles from *Asclepios* and the NCSC mix. Indeed, few portions of chromosome 7, 9, 10, 12, 13, 14, 17 and 18 were also differentially expressed in *Asclepios* (Fig. 5). Therefore, the 1,544 significant genes obtained with the class comparison were introduced in IPA for biological functions and pathway analyses. The results confirmed the tumor profile of *Asclepios*, as genes associated with cancer functions such as neoplasia and tumorigenesis (Table 2) and cell death showed altered expression in this clone. Moreover, many biological pathways involved in cancer where highlighted. Among them, PI3K/AKT signaling and PTEN signaling pathways as well as cell cycle-related proteins regulating the G1/G checkpoint (Fig. 6). Important tumor suppressor genes are significantly down regulated in *Asclepios* (*p*-value<0.001). p21cip1 is for instance down-regulated 11.9 fold, NRG1, 9.1 fold and p15, 4 fold. The tumor suppressor down-regulations strongly suggest an aberrant cellular proliferation of *Asclepios*, similar to tumor growth. To validate this hypothesis, we compared *Asclepios* cell proliferation with other clones using MTS assay. MTS colorimetric assay is based on the reduction of MTS tetrazolium salt into formazan by intracellular dehydrogenases enzymes found in metabolically active cells. The quantity of produced formazan as measured by 490 nm absorbance is directly proportional to the number of living cells in culture. As showed in the Figure 7, no difference in cell number is observed between any NCSC clones after 24 hours of culture. Conversely, after 48 hours, a highly significant increase in absorbance is detected for Asclepios in comparison with the clones NCSC2 to 6 (p<0,001; repeated measures ANOVA, followed by Tuckey post-test), reflecting an elevated number of cells in the wells and a higher proliferation rate in an interval of 48 hours.

**Table 2:** Cancer is one of the main biological function hit of *Asclepios*.

*Category*	*Functions Annotation*	*p-Value*	*Regulation z-score*
Cancer	tumorigenesis	4.56E−20	0.517
Cancer	neoplasia	1.58E−19	0.596
Cancer	cancer	3.29E−19	0.508
Cancer	carcinoma	1.06E−13	−0.154
Cancer	solid tumor	1.16E−13	−0.197
Cancer	transformation	5.94E−12	−0.425
Cancer	cell transformation	9.26E−12	−0.326
Cancer	digestive organ tumor	2.06E−09	−1.266
Cancer	metastasis	6.27E−09	1.970
Cancer	transformation of fibroblast cell lines	1.17E−08	0.390
Cancer	hematologic cancer	2.21E−08	−1.161
Cancer	benign tumor	2.32E−08	−0.468
Cancer	uterine cancer	2.90E−08	
Cancer	gastrointestinal tract cancer	5.65E−08	
Cancer	hematological neoplasia	9.30E−08	−0.684
Cancer	colorectal tumor	1.09E−07	
Cancer	genital tumor	1.23E−07	1.415
Cancer	colorectal cancer	1.59E−07	
Cancer	Waldenstrom's macroglobulinemia	1.62E−07	
Cancer	head and neck cancer	3.54E−07	0.903
Cancer	lung cancer	7.52E−07	
Cancer	lung tumor	1.12E−06	−0.416
Cancer	non-small cell lung cancer	1.62E−06	
Cancer	leukemia	3.94E−06	−1.340
Cancer	plasma cell dyscrasia	3.97E−06	
Cancer	prostatic tumor	4.05E−06	1.398
Cancer	leiomyomatosis	9.21E−06	
Cancer	mammary tumor	9.81E−06	0.075
Cancer	central nervous system tumor	2.49E−05	0.585
Cancer	uterine leiomyoma	3.99E−05	
Cancer	breast cancer	4.69E−05	
Cancer	prostate cancer	5.48E−05	1.107
Cancer	pancreatic tumor	6.34E−05	
Cancer	brain cancer	7.46E−05	0.585
Cancer	metastatic colorectal cancer	8.40E−05	
Cancer	glioma	9.23E−05	0.646
Cancer	infection of tumor cell lines	1.69E−04	0.683
Cancer	transformation of fibroblasts	2.04E−04	0.128
Cancer	adenocarcinoma	2.56E−04	
Cancer	endocrine gland tumor	4.43E−04	
Cancer	metastasis of mammary tumor	5.52E−04	1.041
Cancer	infection of hepatoma cell lines	6.18E−04	0.849
Cancer	colon tumor	6.79E−04	
Cancer	myeloproliferative disorder	8.45E−04	
Cancer	colon cancer	8.82E−04	
Cancer	tumorigenesis of fibrosarcoma	9.13E−04	
Cancer	glioblastoma	1.14E−03	
Cancer	sarcoma	1.32E−03	−0.413

Microarray comparison between *Asclepios* and neural crest stem cell clones revealed 1,544 significant genes that were differentially expressed. Those genes were introduced in IPA for biological functions and pathway analyses. The results confirmed the tumor profile of *Asclepios* with cancer functions.

### 7. *Asclepios* comparison with other cell types includ*ing tumor* cell lines

**Figure 8:**
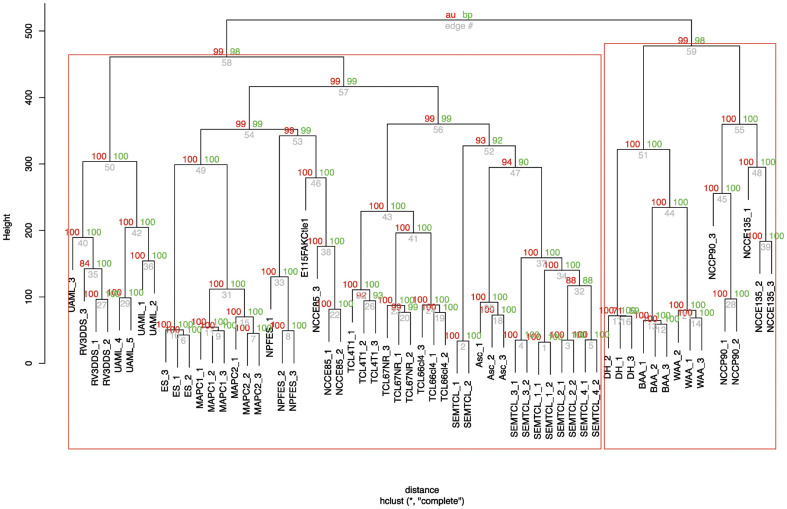
Dendrogram from agglomerative hierarchical clustering of Asclepios and several cell types, including tumor cell lines. Dendrogram generated after agglomerative hierarchical clustering using Euclidean distance, complete linkage and multiscale bootstrap resampling. 61 expression arrays were included in an unsupervised analysis with hierarchical clustering of samples. Spontaneous epithelial mammary tumor cell lines (SEMTCL - GSE13259); Tumor cell lines 67NR, 66cl4 and 4T1 (TCL67NR, TCL66cl4 and TCL4T1 - GSE11259); Embryonal tumor deriving from neural crest cells (NCCE85, NCCE135, NCCP90 - GSE11356); Multipotent adult progenitor cells (MAPC - GSE6291); Developing Heart (DH - GSE7196); Neural precursors obtained from embryonic stem cells (NPFES - GSE8024); White and brown adipose (WAA, BAA - GSE8044); Head Neck Neural Crest Stem Cells (E115FAKCtle1 - GSE11149); Murine acute myeloid leukemia (UAML - GSE30747). Datasets are accessible on GEO datasets/NCBI (http://www.ncbi.nlm.nih.gov/gds). The dendrogram was built with the Euclidean distance as dissimilarity metric and the complete linkage method for definition of the structure. Values on the edges of the clustering are *p*-values (%). Red values are **AU** *p*-values and green values are **BP** values. AU (Approximately Unbiased) *p*-values were computed by multiscale bootstrap resampling. BP (Bootstrap Probability) values were computed by normal bootstrap resampling. R-cran “pvclust” package was used for assessing the uncertainty of this hierarchical cluster analysis for 10,000 permutations of genes. Those values indicated how strongly the cluster was supported by the data.

As chromosome 11 deletions are involved in several cancers including breast cancer [15]–[18], we compared the *Asclepios* transcriptome to several cancer cell lines. Normalized Microarray data from several tumor cell lines, obtained from GEO database, were first organized as clusters using a hierarchical clustering method. The hierarchical clusters were generated, from small clusters of very similar items to large clusters that include more dissimilar items resulting in a dendrogram (Fig. 8). In this study, we compared *Asclepios* to spontaneous epithelial mammary tumor cell lines (SEMTCL - GSE13259), tumor cell lines 67NR, 66cl4 and 4T1 (TCL67NR, TCL66cl4 and TCL4T1 - GSE11259), tumors deriving from neural crest cells (NCCE85, NCCE135, NCCP90 - GSE11356), multipotent adult progenitor cells (MAPC - GSE6291); developing heart (DH - GSE7196), neural precursors obtained from embryonic stem cells (NPFES - GSE8024), white and brown adipose tissue (WAA, BAA - GSE8044), head neck neural crest stem cells (E115FAKCtle1 - GSE11149) and murine acute myeloid leuke*mia* (*UAML* - GSE30747). As observed on Figure 7, *Asclepios* shared numerous similitudes with spontaneous epithelial mammary tumor cell lines (SEMTCL) described by Santisteban et al. [20]. Noteworthy, *Asclepios* and SEMTCL shared similar marker profile as both cell lines were CD24-negative (Fig. 1J), CD34-negative (data not shown), CD44-positive (data not shown), Sca1-negative (data not shown), E-cadherin-negative (data not shown) and N-cadherin-positive (Fig. 2F).

## Discussion

Bone marrow stromal cells (BMSC) are adult multipotent cells that represent an attractive tool in strategies of cellular therapy. Before *in vivo* use, BMSC have to be *in vitro* expanded in order to reach a suitable number of cells for their clinical applications [21]. Several years ago, numerous studies addressed the potential danger of using MSC in cellular therapy. Indeed, it has been shown that the *in vitro* manipulation of both human and murine BMSC may alter the functional and biological properties of the cells, leading to the accumulation of genetic alterations [9], [22]–[26]. However, several laboratories did not confirm the propensity of BMSC to develop morphological or genetic changes [21], [27]–[28]. In light of these discrepant observations, it has been suggested that phenotypic, functional and genetic assays, although known to have limited sensitivity, should be routinely performed on MSC before *in vivo* use to demonstrate whether their biological properties, after *ex vivo* expansion, remain suitable for clinical application.

Similarly, a recent study showed that tumors obtained after human polyomavirus JVC injection into mice bone marrow stromal cells shared mesenchymal and neural crest characteristics [29], suggesting that both cell types could induce tumors. As BMSC are a mixed population containing both mesenchymal stem cells (MSC) and neural crest stem cells (NCSC),, we more specifically analyzed NCSC in this study. Indeed, 6 NCSC clones were characterized in long-term culture process. One of those clones (*Asclepios*) appeared to be tumorigenic as massive tumors were observed after striatal injection. A closer look at the transcriptomic level of *Asclepios* revealed strong modifications of several cell cycle checkpoints. In normal cells, the cell cycle checkpoints are carefully controlled by many factors. These include, among others, the sequential activation and degradation of the cellular cyclins (Cyclin D, A, B, and E), cyclin-dependent kinases (CDKs; serine/threonine kinases) and their inhibitory proteins cyclin-dependent kinase inhibitors (CDKIs, p15, p16 and p21 families) [30]–[31]. Disturbance of cell cycle checkpoints could lead to chromosome instability that can be actively involved in the progression of cancers [32]. Here, we observed a strong down-regulation of tumor suppressors such as p21 (a well-known cyclin-dependant kinase inhibitor), p15 (that normally prevents the activation of the CDK by inhibition of the cyclin D complex) and NRG1, a major anti-proliferative gene [33]. One of the major chromosomal modifications observed in *Asclepios* was located on chromosome 11, as the long arm chromosome 11q was massively down-expressed. Structural aberrations involving 11q are among the most common aberrations in a number of cancers. Indeed, chromosome 11q deletion has been characterized in a number of cancers, including leukemia [18], pancreatic cancer [16], neuroblastoma [17] and breast cancer [34].

In this study, a dendrogram generated after agglomerative hierarchical clustering comparing several transcriptomic data, showed important similitudes between *Asclepios* and mammary tumor cell lines. Transformations of BMSC into epithelial cancers (including breast cancer) have already been reported [35], in that study, they highlighted the fact that BMSC could contribute to breast cancer after Epithelial-Mesenchymal Transition (EMT). In breast cancer EMT is associated with increased aggressiveness, invasiveness and metastasis [36]. However, it is still debated as to whether EMT is an example of transdifferentiation of epithelial cells to mesenchymal cells [37], or an expression of the pluripotency of breast cancer stem cells [38]. In any case, EMT represents a progression of breast cancer to a more malignant phenotype, leading to expression of mesenchymal cells associated-genes and behavior [12]. It is noteworthy that NCSC share many phenotypic traits with classical BMSC [4 retracted in 39], including expression of numerous membrane markers, which in some cases could be associated with EMT process.

One striking observation in this study is the fact that the tumoral character of *Asclepios* was not suspected *in vitro* before grafting the cells. However, a closer look at the proliferation rate of *Asclepios* compared to the other clones revealed a significantly higher level of proliferation after 48 hours, suggesting that proliferative activity should be tested before any clinical use. Likewise, we show here the existence of several modifications in the gene expression profile of a tumorigenic clone that could also be checked before any cellular therapy and thus, be regarded as indicators of a possible tumoral transformation.

In conclusion, this study highlights the fact that NCSC isolated from adult bone marrow may represent a potential danger for cellular therapy, as in some restricted cases, NCSC can adopt a tumorigenic phenotype, producing tumor *in vivo*. We then suggest that phenotypic, functional and genetic assays should be performed on NCSC (as it has already been suggested for MSC) before *in vivo* use to demonstrate whether their biological properties remain suitable for clinical applications.

## Supporting Information

Table S1 **Microarray comparisons between *Asclepios* and NCSC mix reference.** We performed an mRNA expression microarray comparison between *Asclepios* and the NCSC mix. The class comparison revealed 1,544 differentially expressed genes (*p*-value<0.001).
(DOC)
