# Correction: *In Vivo* Tumorigenesis Was Observed after Injection of *In Vitro* Expanded Neural Crest Stem Cells Isolated from Adult Bone Marrow

**DOI:** 10.1371/journal.pone.0256477

**Published:** 2021-09-28

**Authors:** Sabine Wislet-Gendebien, Christophe Poulet, Virginie Neirinckx, Benoit Hennuy, James T. Swingland, Emerence Laudet, Lukas Sommer, Olga Shakova, Vincent Bours, Bernard Rogister

Following the publication of this article [1] concerns were raised regarding similarities between results presented in Fig 1 and results presented in previous and subsequent articles [2 retracted in 6, 3–5] by the same research group. Specifically, the Nestin panel in Fig 1C has also been used to represent Nestin results in Fig 2D of [2 retracted in 6] and Fig 1C of [3], the P75NRT panel in Fig 1D has also been used to represent the P75^NRT^ results in Fig 2D of [2 retracted in 6], and the SOX10 panel in Fig 1E has also been used to represent the SOX10 results in Fig 1D of [3], Fig 2C of [4], and Fig 1F of [5].

The authors have clarified that the study described in this article [1] is a follow up on their research previously published in *Cellular and Molecular Life Sciences*, and that the Fig 1C, 1D and 1E panels in this article [1] represent the same experimental conditions described in their previous articles [2 retracted in 6, 4] which are not offered under a CC-BY license.

**Fig 1:**
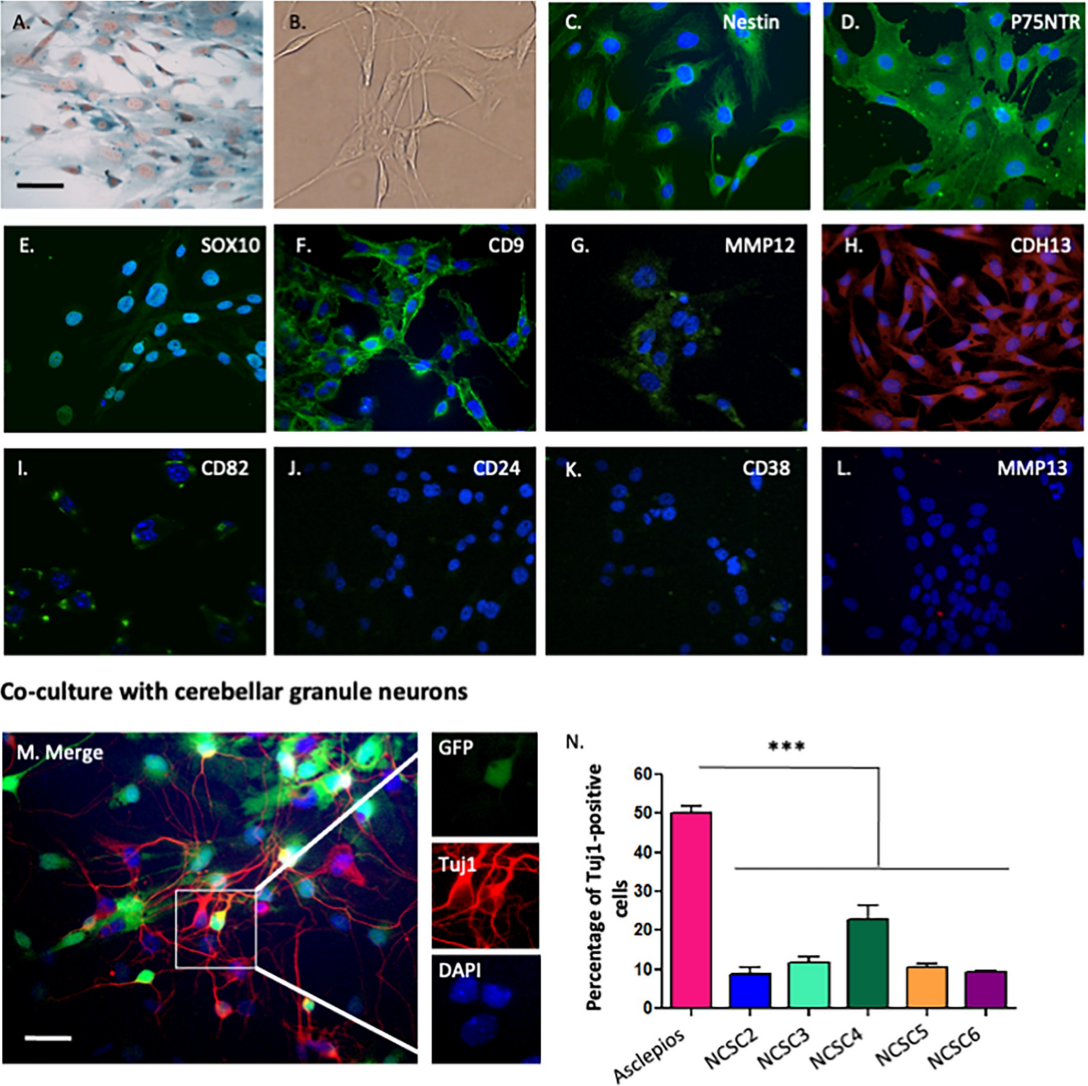
Phenotypic characterization of neural-crest derived cells isolated from adult bone marrow. Neural crest stem cells were isolated from double transgenic Wnt1/Cre-R26R/LacZ mice and cultured under clonal conditions. **A–B.** Neural crest derived clones were morphologically similar to classical BMSC. As clones have been isolated from double transgenic mice Wnt1-CRE/R26R-LacZ, neural crest-derived cells are expressing beta-galactosidase, visualized after an X-gal staining (A). C–L. Immunological characterization revealed that neural crest derived cells were nestin (C), P75NTR (D), Sox10 (E), CD9 (F), MMP12 (G), CDH13 (H), CD82 (I) positives, but CD24 (J), CD38 (K) and MMP13 (L) negatives. **M–N.** A percentage of neural crest stem cells were able to differentiate into beta-III-tubulin-positive cells when co-cultivated with GFP-positive cerebellar granule neurons (M), however, *Asclepios* showed a higher percentage of positive cells as 50.25%±1.70% of cells were beta-III-tubulin-positive, when around 15% of cells were observed with the other clones (N) (mean ± SEM, *n*  =  3, p<0.001, ANOVA followed by Bonferroni *post hoc* test). Nuclei were counterstained with Dapi (blue) on panels C to N. Scale bars  =  30 µm.

The Board of Ethics and Scientific Integrity of University of Liège investigated the overlap between the aforementioned panels and recommended the article be corrected. In addition, a member of *PLOS ONE*’s Editorial Board advised that the updated figures support the results and conclusions reported in the original article. As the original images presented in Fig 1C, 1D and 1E are not licensed for reproduction and distribution under the terms of the Creative Commons Attribution License (or Public Domain License for US gov), this article was republished on September 28, 2021 to remove this content and replace it with alternative relevant immunological characterization images. Please download this article again to view the correct version.

## Supporting information

S1 File Original images underlying Fig 1A–1M. (ZIP)

S2 File Raw data underlying Fig 1N. (PZF)

## References

[1] Wislet-Gendebien S, Poulet C, Neirinckx V, Hennuy B, Swingland JT, Laudet E, et al. (2012) *In Vivo* Tumorigenesis Was Observed after Injection of *In Vitro* Expanded Neural Crest Stem Cells Isolated from Adult Bone Marrow. PLoS ONE 7(10): e46425. 10.1371/journal.pone.0046425 23071568 PMC3465331

[2] Wislet-Gendebien S., Laudet E., Neirinckx V. et al. (2012) RETRACTED ARTICLE: Mesenchymal stem cells and neural crest stem cells from adult bone marrow: characterization of their surprising similarities and differences. Cell. Mol. Life Sci. 69, 2593–2608. 10.1007/s00018-012-0937-1 22349262 PMC11114712

[3] Neirinckx V, Marquet A, Coste C, Rogister B, Wislet-Gendebien S (2013) Adult Bone Marrow Neural Crest Stem Cells and Mesenchymal Stem Cells Are Not Able to Replace Lost Neurons in Acute MPTP-Lesioned Mice. PLoS ONE 8(5): e64723. 10.1371/journal.pone.0064723 23741377 PMC3669410

[4] Glejzer A., Laudet E., Leprince P. et al. (2011) Wnt1 and BMP2: two factors recruiting multipotent neural crest progenitors isolated from adult bone marrow. Cell. Mol. Life Sci. 68, 2101–2114. 10.1007/s00018-010-0558-5 20976520 PMC11114799

[5] Coste C, Neirinckx V, Sharma A, Agirman G, Rogister B, Foguenne J, et al. (2017) Human bone marrow harbors cells with neural crest-associated characteristics like human adipose and dermis tissues. PLoS ONE 12(7): e0177962. 10.1371/journal.pone.0177962 28683107 PMC5500284

[6] Wislet-Gendebien S, Laudet E, Neirinckx V, et al. (2021) Retraction note: Mesenchymal stem cells and neural crest stem cells from adult bone marrow: characterization of their surprising similarities and differences. Cell. Mol. Life Sci. 78, 5041. doi: 10.1007/s00018-012-0937-1 33963879 PMC11072493

