# Supplementary material for: In Vivo Tumorigenesis Was Observed after Injection of In Vitro Expanded Neural Crest Stem Cells Isolated from Adult Bone Marrow
Source: PLoS One. 2012 Oct 5;7(10):e46425. doi: 10.1371/journal.pone.0046425 (PMC3465331; doi:10.1371/journal.pone.0046425)
Supplement: Table S1 — Microarray comparisons between Asclepios and NCSC mix reference. We performed an mRNA expression microarray comparison between Asclepios and the NCSC mix. The class comparison revealed 1,544 differentially expressed genes (p-value<0.001). (DOC) [file journal.pone.0046425.s001.doc]

In vivo tumorigenesis was observed after injection of in vitro expanded neural crest stem cells isolated from adult bone marrow.

Sabine Wislet-Gendebien^1*^, Christophe Poulet^2*^, Virginie Neirinckx^1^, Benoit Hennuy^6^, James T. Swingland^7^, Emerence Laudet^1^, Lukas Sommer^3^, Olga Shakova^3^, Vincent Bours^2^, Bernard Rogister^1,4,5^ .

**1** Groupe Interdisciplinaire de Génoprotéomique appliquée (GIGA), Unit of Neurosciences, University of Liege, Liège, Belgium. **2** GIGA, Unit of Human Genetics, University of Liege, Liège, Belgium. **3** Institute of Anatomy, University of Zurich, Zurich, Switzerland. **4** GIGA, Unit of Development, Stem Cells and Regenerative Medicine, University of Liège, Liège, Belgium. **5** Department of Neurology, Centre Hospitalier Universitaire de Liège, Liège, Belgium. **6** GIGA Genomics Platform, University of Liege, Liège, Belgium. **7** King's College, Department of Clinical Neuroscience, Division of Experimental Medicine, London, UK.

* Sabine Wislet-Gendebien and Christophe Poulet equally contributed to this work.

**Corresponding Author:**

Sabine Wislet-Gendebien

[s.wislet@ulg.ac.be](mailto:s.wislet@ulg.ac.be)

Phone: +32-4-3665956

Fax: +32-4-3665912

**Key words**

Neural crest stem cells; adult bone marrow; microarray; tumorigenesis; cancer; tumor

**SUPPLEMENTARY TABLE LEGENDS**

**TABLE S1. Microarray comparisons between *Asclepios* and NCSC mix reference.** We performed an mRNA expression microarray comparison between *Asclepios* and the NCSC mix. The class comparison revealed 1,544 differentially expressed genes (*p*-value < 0.001).

TABLE S1

|  | **Parametric p-value** | **FDR** | **Geom mean of intensities in Asclepios** | **Geom mean of intensities in NCSC** | **Fold-change** | **ProbeSet** | **Symbol** | **Name** |
| --- | --- | --- | --- | --- | --- | --- | --- | --- |
|
| 1 | < 1e-07 | < 1e-07 | 4,48 | 155,63 | 0,029 | [1422975_at](https://www.affymetrix.com/LinkServlet?probeset=1422975_at) | [Mme](http://www.ncbi.nlm.nih.gov/entrez/query.fcgi?cmd=search&db=gene&term=Mme) | membrane metallo endopeptidase |
| 2 | < 1e-07 | < 1e-07 | 4,77 | 387,37 | 0,012 | [1438169_a_at](https://www.affymetrix.com/LinkServlet?probeset=1438169_a_at) | [Frmd4b](http://www.ncbi.nlm.nih.gov/entrez/query.fcgi?cmd=search&db=gene&term=Frmd4b) | FERM domain containing 4B |
| 3 | < 1e-07 | < 1e-07 | 5,81 | 334,1 | 0,017 | [1456072_at](https://www.affymetrix.com/LinkServlet?probeset=1456072_at) | [Ppp1r9a](http://www.ncbi.nlm.nih.gov/entrez/query.fcgi?cmd=search&db=gene&term=Ppp1r9a) | protein phosphatase 1, regulatory (inhibitor) subunit 9A |
| 4 | 1,00E-07 | 0,000328 | 6,23 | 4300,41 | 0,0014 | [1450047_at](https://www.affymetrix.com/LinkServlet?probeset=1450047_at) | [Hs6st2](http://www.ncbi.nlm.nih.gov/entrez/query.fcgi?cmd=search&db=gene&term=Hs6st2) | heparan sulfate 6-O-sulfotransferase 2 |
| 5 | 1,00E-07 | 0,000328 | 4,89 | 175,51 | 0,028 | [1419476_at](https://www.affymetrix.com/LinkServlet?probeset=1419476_at) | [Adamdec1](http://www.ncbi.nlm.nih.gov/entrez/query.fcgi?cmd=search&db=gene&term=Adamdec1) | ADAM-like, decysin 1 |
| 6 | 1,00E-07 | 0,000328 | 137 | 5,02 | 27,31 | [1418299_at](https://www.affymetrix.com/LinkServlet?probeset=1418299_at) | [Dpysl4](http://www.ncbi.nlm.nih.gov/entrez/query.fcgi?cmd=search&db=gene&term=Dpysl4) | dihydropyrimidinase-like 4 |
| 7 | 3,00E-07 | 0,000656 | 5,98 | 47,16 | 0,13 | [1418569_at](https://www.affymetrix.com/LinkServlet?probeset=1418569_at) | [Fblim1](http://www.ncbi.nlm.nih.gov/entrez/query.fcgi?cmd=search&db=gene&term=Fblim1) | filamin binding LIM protein 1 |
| 8 | 3,00E-07 | 0,000656 | 4,67 | 300,11 | 0,016 | [1440285_at](https://www.affymetrix.com/LinkServlet?probeset=1440285_at) | [Ppp1r9a](http://www.ncbi.nlm.nih.gov/entrez/query.fcgi?cmd=search&db=gene&term=Ppp1r9a) | protein phosphatase 1, regulatory (inhibitor) subunit 9A |
| 9 | 3,00E-07 | 0,000656 | 4,54 | 1286,83 | 0,0035 | [1417210_at](https://www.affymetrix.com/LinkServlet?probeset=1417210_at) | [Eif2s3y](http://www.ncbi.nlm.nih.gov/entrez/query.fcgi?cmd=search&db=gene&term=Eif2s3y) | eukaryotic translation initiation factor 2, subunit 3, structural gene Y-linked |
| 10 | 4,00E-07 | 0,000656 | 4,73 | 284,48 | 0,017 | [1423278_at](https://www.affymetrix.com/LinkServlet?probeset=1423278_at) | [Ptprk](http://www.ncbi.nlm.nih.gov/entrez/query.fcgi?cmd=search&db=gene&term=Ptprk) | protein tyrosine phosphatase, receptor type, K |
| 11 | 4,00E-07 | 0,000656 | 6481,36 | 2701,31 | 2,4 | [1437226_x_at](https://www.affymetrix.com/LinkServlet?probeset=1437226_x_at) | [Marcksl1](http://www.ncbi.nlm.nih.gov/entrez/query.fcgi?cmd=search&db=gene&term=Marcksl1) | MARCKS-like 1 |
| 12 | 4,00E-07 | 0,000656 | 4,62 | 506,52 | 0,0091 | [1416613_at](https://www.affymetrix.com/LinkServlet?probeset=1416613_at) | [Cyp1b1](http://www.ncbi.nlm.nih.gov/entrez/query.fcgi?cmd=search&db=gene&term=Cyp1b1) | cytochrome P450, family 1, subfamily b, polypeptide 1 |
| 13 | 5,00E-07 | 0,000702 | 4,64 | 429,14 | 0,011 | [1447928_at](https://www.affymetrix.com/LinkServlet?probeset=1447928_at) | [Car5b](http://www.ncbi.nlm.nih.gov/entrez/query.fcgi?cmd=search&db=gene&term=Car5b) | carbonic anhydrase 5b, mitochondrial |
| 14 | 5,00E-07 | 0,000702 | 6,55 | 419,87 | 0,016 | [1433653_at](https://www.affymetrix.com/LinkServlet?probeset=1433653_at) | [Fam20a](http://www.ncbi.nlm.nih.gov/entrez/query.fcgi?cmd=search&db=gene&term=Fam20a) | family with sequence similarity 20, member A |
| 15 | 7,00E-07 | 0,000874 | 4,6 | 118,7 | 0,039 | [1434990_at](https://www.affymetrix.com/LinkServlet?probeset=1434990_at) | [Ppm1e](http://www.ncbi.nlm.nih.gov/entrez/query.fcgi?cmd=search&db=gene&term=Ppm1e) | protein phosphatase 1E (PP2C domain containing) |
| 16 | 8,00E-07 | 0,000874 | 4,78 | 213,61 | 0,022 | [1420938_at](https://www.affymetrix.com/LinkServlet?probeset=1420938_at) | [Hs6st2](http://www.ncbi.nlm.nih.gov/entrez/query.fcgi?cmd=search&db=gene&term=Hs6st2) | heparan sulfate 6-O-sulfotransferase 2 |
| 17 | 8,00E-07 | 0,000874 | 5,46 | 123,94 | 0,044 | [1459804_at](https://www.affymetrix.com/LinkServlet?probeset=1459804_at) | [Crebbp](http://www.ncbi.nlm.nih.gov/entrez/query.fcgi?cmd=search&db=gene&term=Crebbp) | CREB binding protein |
| 18 | 8,00E-07 | 0,000874 | 5,07 | 345,71 | 0,015 | [1417772_at](https://www.affymetrix.com/LinkServlet?probeset=1417772_at) | [Grhpr](http://www.ncbi.nlm.nih.gov/entrez/query.fcgi?cmd=search&db=gene&term=Grhpr) | glyoxylate reductase/hydroxypyruvate reductase |
| 19 | 9,00E-07 | 0,000894 | 639,74 | 245 | 2,61 | [1419328_at](https://www.affymetrix.com/LinkServlet?probeset=1419328_at) | [Sema4f](http://www.ncbi.nlm.nih.gov/entrez/query.fcgi?cmd=search&db=gene&term=Sema4f) | sema domain, immunoglobulin domain (Ig), TM domain, and short cytoplasmic domain |
| 20 | 1,00E-06 | 0,000894 | 111,96 | 715,72 | 0,16 | [1438036_x_at](https://www.affymetrix.com/LinkServlet?probeset=1438036_x_at) | [Fam82a1](http://www.ncbi.nlm.nih.gov/entrez/query.fcgi?cmd=search&db=gene&term=Fam82a1) | family with sequence similarity 82, member A1 |
| 21 | 1,00E-06 | 0,000894 | 477,93 | 179,38 | 2,66 | [1423892_at](https://www.affymetrix.com/LinkServlet?probeset=1423892_at) | [Apbb1](http://www.ncbi.nlm.nih.gov/entrez/query.fcgi?cmd=search&db=gene&term=Apbb1) | amyloid beta (A4) precursor protein-binding, family B, member 1 |
| 22 | 1,00E-06 | 0,000894 | 6,89 | 217,81 | 0,032 | [1443219_at](https://www.affymetrix.com/LinkServlet?probeset=1443219_at) | [NA](http://www.ncbi.nlm.nih.gov/entrez/query.fcgi?cmd=search&db=gene&term=NA) | NA |
| 23 | 1,10E-06 | 0,000941 | 841,49 | 211,19 | 3,98 | [1434138_at](https://www.affymetrix.com/LinkServlet?probeset=1434138_at) | [Prune](http://www.ncbi.nlm.nih.gov/entrez/query.fcgi?cmd=search&db=gene&term=Prune) | prune homolog (Drosophila) |
| 24 | 1,20E-06 | 0,000944 | 3154,67 | 8807,16 | 0,36 | [1451827_a_at](https://www.affymetrix.com/LinkServlet?probeset=1451827_a_at) | [Nox4](http://www.ncbi.nlm.nih.gov/entrez/query.fcgi?cmd=search&db=gene&term=Nox4) | NADPH oxidase 4 |
| 25 | 1,20E-06 | 0,000944 | 681,25 | 2257,86 | 0,3 | [1448196_at](https://www.affymetrix.com/LinkServlet?probeset=1448196_at) | [Mat2b](http://www.ncbi.nlm.nih.gov/entrez/query.fcgi?cmd=search&db=gene&term=Mat2b) | methionine adenosyltransferase II, beta |
| 26 | 1,30E-06 | 0,000983 | 4,92 | 1334,46 | 0,0037 | [1418752_at](https://www.affymetrix.com/LinkServlet?probeset=1418752_at) | [Aldh3a1](http://www.ncbi.nlm.nih.gov/entrez/query.fcgi?cmd=search&db=gene&term=Aldh3a1) | aldehyde dehydrogenase family 3, subfamily A1 |
| 27 | 1,40E-06 | 0,00102 | 5,13 | 111,77 | 0,046 | [1443381_at](https://www.affymetrix.com/LinkServlet?probeset=1443381_at) | [Etv4](http://www.ncbi.nlm.nih.gov/entrez/query.fcgi?cmd=search&db=gene&term=Etv4) | ets variant gene 4 (E1A enhancer binding protein, E1AF) |
| 28 | 1,50E-06 | 0,00105 | 4,43 | 234,34 | 0,019 | [1419705_at](https://www.affymetrix.com/LinkServlet?probeset=1419705_at) | [Car5b](http://www.ncbi.nlm.nih.gov/entrez/query.fcgi?cmd=search&db=gene&term=Car5b) | carbonic anhydrase 5b, mitochondrial |
| 29 | 1,60E-06 | 0,00109 | 24,92 | 189,48 | 0,13 | [1435520_at](https://www.affymetrix.com/LinkServlet?probeset=1435520_at) | [Msi2](http://www.ncbi.nlm.nih.gov/entrez/query.fcgi?cmd=search&db=gene&term=Msi2) | Musashi homolog 2 (Drosophila) |
| 30 | 1,80E-06 | 0,00116 | 257,71 | 462,55 | 0,56 | [1441906_x_at](https://www.affymetrix.com/LinkServlet?probeset=1441906_x_at) | [Syap1](http://www.ncbi.nlm.nih.gov/entrez/query.fcgi?cmd=search&db=gene&term=Syap1) | synapse associated protein 1 |
| 31 | 1,90E-06 | 0,00116 | 967,44 | 3404,13 | 0,28 | [1448939_at](https://www.affymetrix.com/LinkServlet?probeset=1448939_at) | [Usp25](http://www.ncbi.nlm.nih.gov/entrez/query.fcgi?cmd=search&db=gene&term=Usp25) | ubiquitin specific peptidase 25 |
| 32 | 2,00E-06 | 0,00116 | 281,51 | 586,48 | 0,48 | [1452166_a_at](https://www.affymetrix.com/LinkServlet?probeset=1452166_a_at) | [Krt10](http://www.ncbi.nlm.nih.gov/entrez/query.fcgi?cmd=search&db=gene&term=Krt10) | keratin 10 |
| 33 | 2,00E-06 | 0,00116 | 3376,76 | 380,94 | 8,86 | [1424985_a_at](https://www.affymetrix.com/LinkServlet?probeset=1424985_a_at) | [Sox10](http://www.ncbi.nlm.nih.gov/entrez/query.fcgi?cmd=search&db=gene&term=Sox10) | SRY-box containing gene 10 |
| 34 | 2,10E-06 | 0,00116 | 5,75 | 255,13 | 0,023 | [1429987_at](https://www.affymetrix.com/LinkServlet?probeset=1429987_at) | [9930013L23Rik](http://www.ncbi.nlm.nih.gov/entrez/query.fcgi?cmd=search&db=gene&term=9930013L23Rik) | RIKEN cDNA 9930013L23 gene |
| 35 | 2,20E-06 | 0,00116 | 4,4 | 74,73 | 0,059 | [1439610_at](https://www.affymetrix.com/LinkServlet?probeset=1439610_at) | [Rab27b](http://www.ncbi.nlm.nih.gov/entrez/query.fcgi?cmd=search&db=gene&term=Rab27b) | RAB27b, member RAS oncogene family |
| 36 | 2,30E-06 | 0,00116 | 336,15 | 121,67 | 2,76 | [1418294_at](https://www.affymetrix.com/LinkServlet?probeset=1418294_at) | [Epb4.1l4b](http://www.ncbi.nlm.nih.gov/entrez/query.fcgi?cmd=search&db=gene&term=Epb4.1l4b) | erythrocyte protein band 4.1-like 4b |
| 37 | 2,30E-06 | 0,00116 | 4,5 | 82,74 | 0,054 | [1419063_at](https://www.affymetrix.com/LinkServlet?probeset=1419063_at) | [Ugt8a](http://www.ncbi.nlm.nih.gov/entrez/query.fcgi?cmd=search&db=gene&term=Ugt8a) | UDP galactosyltransferase 8A |
| 38 | 2,40E-06 | 0,00116 | 900,27 | 4305,15 | 0,21 | [1435554_at](https://www.affymetrix.com/LinkServlet?probeset=1435554_at) | [Tmcc3](http://www.ncbi.nlm.nih.gov/entrez/query.fcgi?cmd=search&db=gene&term=Tmcc3) | transmembrane and coiled coil domains 3 |
| 39 | 2,50E-06 | 0,00116 | 6,92 | 215,98 | 0,032 | [1437467_at](https://www.affymetrix.com/LinkServlet?probeset=1437467_at) | [Alcam](http://www.ncbi.nlm.nih.gov/entrez/query.fcgi?cmd=search&db=gene&term=Alcam) | activated leukocyte cell adhesion molecule |
| 40 | 2,50E-06 | 0,00116 | 4,69 | 543,81 | 0,0086 | [1418357_at](https://www.affymetrix.com/LinkServlet?probeset=1418357_at) | [Foxg1](http://www.ncbi.nlm.nih.gov/entrez/query.fcgi?cmd=search&db=gene&term=Foxg1) | forkhead box G1 |
| 41 | 2,50E-06 | 0,00116 | 4,4 | 452,29 | 0,0097 | [1424903_at](https://www.affymetrix.com/LinkServlet?probeset=1424903_at) | [Kdm5d](http://www.ncbi.nlm.nih.gov/entrez/query.fcgi?cmd=search&db=gene&term=Kdm5d) | lysine (K)-specific demethylase 5D |
| 42 | 2,50E-06 | 0,00116 | 94,53 | 5,15 | 18,35 | [1425840_a_at](https://www.affymetrix.com/LinkServlet?probeset=1425840_a_at) | [Sema3f](http://www.ncbi.nlm.nih.gov/entrez/query.fcgi?cmd=search&db=gene&term=Sema3f) | sema domain, immunoglobulin domain (Ig), short basic domain, secreted, (semaphorin) 3F |
| 43 | 2,60E-06 | 0,00116 | 4,51 | 13,16 | 0,34 | [1440929_at](https://www.affymetrix.com/LinkServlet?probeset=1440929_at) | [Ggnbp2](http://www.ncbi.nlm.nih.gov/entrez/query.fcgi?cmd=search&db=gene&term=Ggnbp2) | gametogenetin binding protein 2 |
| 44 | 2,60E-06 | 0,00116 | 4185,06 | 9521,55 | 0,44 | [1417472_at](https://www.affymetrix.com/LinkServlet?probeset=1417472_at) | [Myh9](http://www.ncbi.nlm.nih.gov/entrez/query.fcgi?cmd=search&db=gene&term=Myh9) | myosin, heavy polypeptide 9, non-muscle |
| 45 | 2,80E-06 | 0,0012 | 2252,9 | 95,02 | 23,71 | [1420901_a_at](https://www.affymetrix.com/LinkServlet?probeset=1420901_a_at) | [Hk1](http://www.ncbi.nlm.nih.gov/entrez/query.fcgi?cmd=search&db=gene&term=Hk1) | hexokinase 1 |
| 46 | 2,80E-06 | 0,0012 | 4,88 | 855,19 | 0,0057 | [1436791_at](https://www.affymetrix.com/LinkServlet?probeset=1436791_at) | [Wnt5a](http://www.ncbi.nlm.nih.gov/entrez/query.fcgi?cmd=search&db=gene&term=Wnt5a) | wingless-related MMTV integration site 5A |
| 47 | 2,90E-06 | 0,0012 | 4,69 | 36,16 | 0,13 | [1423805_at](https://www.affymetrix.com/LinkServlet?probeset=1423805_at) | [Dab2](http://www.ncbi.nlm.nih.gov/entrez/query.fcgi?cmd=search&db=gene&term=Dab2) | disabled homolog 2 (Drosophila) |
| 48 | 3,00E-06 | 0,0012 | 96,03 | 598,96 | 0,16 | [1419035_s_at](https://www.affymetrix.com/LinkServlet?probeset=1419035_s_at) | [NA](http://www.ncbi.nlm.nih.gov/entrez/query.fcgi?cmd=search&db=gene&term=NA) | NA |
| 49 | 3,00E-06 | 0,0012 | 704,56 | 207,16 | 3,4 | [1436306_at](https://www.affymetrix.com/LinkServlet?probeset=1436306_at) | [Ppp6r1](http://www.ncbi.nlm.nih.gov/entrez/query.fcgi?cmd=search&db=gene&term=Ppp6r1) | protein phosphatase 6, regulatory subunit 1 |
| 50 | 3,20E-06 | 0,00122 | 6,94 | 85,4 | 0,081 | [1454224_at](https://www.affymetrix.com/LinkServlet?probeset=1454224_at) | [2010300F17Rik](http://www.ncbi.nlm.nih.gov/entrez/query.fcgi?cmd=search&db=gene&term=2010300F17Rik) | RIKEN cDNA 2010300F17 gene |
| 51 | 3,20E-06 | 0,00122 | 663,27 | 1801,56 | 0,37 | [1435029_at](https://www.affymetrix.com/LinkServlet?probeset=1435029_at) | [B230120H23Rik](http://www.ncbi.nlm.nih.gov/entrez/query.fcgi?cmd=search&db=gene&term=B230120H23Rik) | RIKEN cDNA B230120H23 gene |
| 52 | 3,30E-06 | 0,00122 | 232,19 | 2748,5 | 0,084 | [1417087_at](https://www.affymetrix.com/LinkServlet?probeset=1417087_at) | [Glg1](http://www.ncbi.nlm.nih.gov/entrez/query.fcgi?cmd=search&db=gene&term=Glg1) | golgi apparatus protein 1 |
| 53 | 3,30E-06 | 0,00122 | 58,41 | 854,46 | 0,068 | [1436448_a_at](https://www.affymetrix.com/LinkServlet?probeset=1436448_a_at) | [Ptgs1](http://www.ncbi.nlm.nih.gov/entrez/query.fcgi?cmd=search&db=gene&term=Ptgs1) | prostaglandin-endoperoxide synthase 1 |
| 54 | 3,50E-06 | 0,00123 | 55,76 | 5,46 | 10,2 | [1431326_a_at](https://www.affymetrix.com/LinkServlet?probeset=1431326_a_at) | [Tmod2](http://www.ncbi.nlm.nih.gov/entrez/query.fcgi?cmd=search&db=gene&term=Tmod2) | tropomodulin 2 |
| 55 | 3,50E-06 | 0,00123 | 168,81 | 5,77 | 29,25 | [1449956_at](https://www.affymetrix.com/LinkServlet?probeset=1449956_at) | [Prkce](http://www.ncbi.nlm.nih.gov/entrez/query.fcgi?cmd=search&db=gene&term=Prkce) | protein kinase C, epsilon |
| 56 | 3,50E-06 | 0,00123 | 26,94 | 151,26 | 0,18 | [1431834_a_at](https://www.affymetrix.com/LinkServlet?probeset=1431834_a_at) | [Emilin1](http://www.ncbi.nlm.nih.gov/entrez/query.fcgi?cmd=search&db=gene&term=Emilin1) | elastin microfibril interfacer 1 |
| 57 | 3,70E-06 | 0,00125 | 2947,29 | 1182,09 | 2,49 | [1415713_a_at](https://www.affymetrix.com/LinkServlet?probeset=1415713_a_at) | [Ddx24](http://www.ncbi.nlm.nih.gov/entrez/query.fcgi?cmd=search&db=gene&term=Ddx24) | DEAD (Asp-Glu-Ala-Asp) box polypeptide 24 |
| 58 | 3,70E-06 | 0,00125 | 80,43 | 845,07 | 0,095 | [1452493_s_at](https://www.affymetrix.com/LinkServlet?probeset=1452493_s_at) | [NA](http://www.ncbi.nlm.nih.gov/entrez/query.fcgi?cmd=search&db=gene&term=NA) | NA |
| 59 | 3,90E-06 | 0,00129 | 1330,43 | 389,19 | 3,42 | [1452024_a_at](https://www.affymetrix.com/LinkServlet?probeset=1452024_a_at) | [Ldb1](http://www.ncbi.nlm.nih.gov/entrez/query.fcgi?cmd=search&db=gene&term=Ldb1) | LIM domain binding 1 |
| 60 | 4,00E-06 | 0,00129 | 3053,64 | 959,03 | 3,18 | [1415792_at](https://www.affymetrix.com/LinkServlet?probeset=1415792_at) | [Rbck1](http://www.ncbi.nlm.nih.gov/entrez/query.fcgi?cmd=search&db=gene&term=Rbck1) | RanBP-type and C3HC4-type zinc finger containing 1 |
| 61 | 4,10E-06 | 0,00129 | 4,53 | 429,95 | 0,011 | [1429579_at](https://www.affymetrix.com/LinkServlet?probeset=1429579_at) | [6330407I18Rik](http://www.ncbi.nlm.nih.gov/entrez/query.fcgi?cmd=search&db=gene&term=6330407I18Rik) | RIKEN cDNA 6330407I18 gene |
| 62 | 4,20E-06 | 0,00129 | 8,54 | 222,06 | 0,038 | [1448978_at](https://www.affymetrix.com/LinkServlet?probeset=1448978_at) | [Ngef](http://www.ncbi.nlm.nih.gov/entrez/query.fcgi?cmd=search&db=gene&term=Ngef) | neuronal guanine nucleotide exchange factor |
| 63 | 4,30E-06 | 0,00129 | 435,57 | 1258,81 | 0,35 | [1429690_at](https://www.affymetrix.com/LinkServlet?probeset=1429690_at) | [Zfp946](http://www.ncbi.nlm.nih.gov/entrez/query.fcgi?cmd=search&db=gene&term=Zfp946) | zinc finger protein 946 |
| 64 | 4,30E-06 | 0,00129 | 50,31 | 253,53 | 0,2 | [1455087_at](https://www.affymetrix.com/LinkServlet?probeset=1455087_at) | [D7Ertd715e](http://www.ncbi.nlm.nih.gov/entrez/query.fcgi?cmd=search&db=gene&term=D7Ertd715e) | DNA segment, Chr 7, ERATO Doi 715, expressed |
| 65 | 4,50E-06 | 0,00129 | 909,93 | 115,03 | 7,91 | [1426539_at](https://www.affymetrix.com/LinkServlet?probeset=1426539_at) | [Usp11](http://www.ncbi.nlm.nih.gov/entrez/query.fcgi?cmd=search&db=gene&term=Usp11) | ubiquitin specific peptidase 11 |
| 66 | 4,50E-06 | 0,00129 | 5,1 | 59,9 | 0,085 | [1443975_at](https://www.affymetrix.com/LinkServlet?probeset=1443975_at) | [Trip11](http://www.ncbi.nlm.nih.gov/entrez/query.fcgi?cmd=search&db=gene&term=Trip11) | thyroid hormone receptor interactor 11 |
| 67 | 4,60E-06 | 0,00129 | 8,47 | 565,08 | 0,015 | [1435747_at](https://www.affymetrix.com/LinkServlet?probeset=1435747_at) | [Fgf14](http://www.ncbi.nlm.nih.gov/entrez/query.fcgi?cmd=search&db=gene&term=Fgf14) | fibroblast growth factor 14 |
| 68 | 4,60E-06 | 0,00129 | 5,04 | 98,11 | 0,051 | [1441466_at](https://www.affymetrix.com/LinkServlet?probeset=1441466_at) | [Tra2b](http://www.ncbi.nlm.nih.gov/entrez/query.fcgi?cmd=search&db=gene&term=Tra2b) | transformer 2 beta homolog (Drosophila) |
| 69 | 4,60E-06 | 0,00129 | 2558,16 | 1642,68 | 1,56 | [1450376_at](https://www.affymetrix.com/LinkServlet?probeset=1450376_at) | [Mxi1](http://www.ncbi.nlm.nih.gov/entrez/query.fcgi?cmd=search&db=gene&term=Mxi1) | Max interacting protein 1 |
| 70 | 4,60E-06 | 0,00129 | 8,11 | 679,14 | 0,012 | [1422945_a_at](https://www.affymetrix.com/LinkServlet?probeset=1422945_a_at) | [Kif5c](http://www.ncbi.nlm.nih.gov/entrez/query.fcgi?cmd=search&db=gene&term=Kif5c) | kinesin family member 5C |
| 71 | 4,70E-06 | 0,0013 | 114,18 | 332,93 | 0,34 | [1434192_at](https://www.affymetrix.com/LinkServlet?probeset=1434192_at) | [Zzef1](http://www.ncbi.nlm.nih.gov/entrez/query.fcgi?cmd=search&db=gene&term=Zzef1) | zinc finger, ZZ-type with EF hand domain 1 |
| 72 | 4,80E-06 | 0,00131 | 4,44 | 3657,06 | 0,0012 | [1426438_at](https://www.affymetrix.com/LinkServlet?probeset=1426438_at) | [Ddx3y](http://www.ncbi.nlm.nih.gov/entrez/query.fcgi?cmd=search&db=gene&term=Ddx3y) | DEAD (Asp-Glu-Ala-Asp) box polypeptide 3, Y-linked |
| 73 | 5,20E-06 | 0,00138 | 240,19 | 1934,45 | 0,12 | [1429624_at](https://www.affymetrix.com/LinkServlet?probeset=1429624_at) | [Sltm](http://www.ncbi.nlm.nih.gov/entrez/query.fcgi?cmd=search&db=gene&term=Sltm) | SAFB-like, transcription modulator |
| 74 | 5,20E-06 | 0,00138 | 661,29 | 8,37 | 79 | [1421958_at](https://www.affymetrix.com/LinkServlet?probeset=1421958_at) | [L1cam](http://www.ncbi.nlm.nih.gov/entrez/query.fcgi?cmd=search&db=gene&term=L1cam) | L1 cell adhesion molecule |
| 75 | 5,70E-06 | 0,00145 | 4,54 | 103,36 | 0,044 | [1453125_at](https://www.affymetrix.com/LinkServlet?probeset=1453125_at) | [Sox11](http://www.ncbi.nlm.nih.gov/entrez/query.fcgi?cmd=search&db=gene&term=Sox11) | SRY-box containing gene 11 |
| 76 | 5,70E-06 | 0,00145 | 2515,25 | 777,73 | 3,23 | [1451067_at](https://www.affymetrix.com/LinkServlet?probeset=1451067_at) | [Sgta](http://www.ncbi.nlm.nih.gov/entrez/query.fcgi?cmd=search&db=gene&term=Sgta) | small glutamine-rich tetratricopeptide repeat (TPR)-containing, alpha |
| 77 | 5,80E-06 | 0,00145 | 304,44 | 3423,26 | 0,089 | [1428967_at](https://www.affymetrix.com/LinkServlet?probeset=1428967_at) | [Igf1r](http://www.ncbi.nlm.nih.gov/entrez/query.fcgi?cmd=search&db=gene&term=Igf1r) | insulin-like growth factor I receptor |
| 78 | 5,90E-06 | 0,00145 | 5,2 | 516,28 | 0,01 | [1434354_at](https://www.affymetrix.com/LinkServlet?probeset=1434354_at) | [Maob](http://www.ncbi.nlm.nih.gov/entrez/query.fcgi?cmd=search&db=gene&term=Maob) | monoamine oxidase B |
| 79 | 5,90E-06 | 0,00145 | 19,01 | 381,92 | 0,05 | [1451268_at](https://www.affymetrix.com/LinkServlet?probeset=1451268_at) | [Tram1l1](http://www.ncbi.nlm.nih.gov/entrez/query.fcgi?cmd=search&db=gene&term=Tram1l1) | translocation associated membrane protein 1-like 1 |
| 80 | 6,20E-06 | 0,00145 | 351,28 | 699,36 | 0,5 | [1427028_at](https://www.affymetrix.com/LinkServlet?probeset=1427028_at) | [Lgr6](http://www.ncbi.nlm.nih.gov/entrez/query.fcgi?cmd=search&db=gene&term=Lgr6) | leucine-rich repeat-containing G protein-coupled receptor 6 |
| 81 | 6,20E-06 | 0,00145 | 185,33 | 672,56 | 0,28 | [1437197_at](https://www.affymetrix.com/LinkServlet?probeset=1437197_at) | [Sorbs2](http://www.ncbi.nlm.nih.gov/entrez/query.fcgi?cmd=search&db=gene&term=Sorbs2) | sorbin and SH3 domain containing 2 |
| 82 | 6,20E-06 | 0,00145 | 100,4 | 8,53 | 11,77 | [1450049_a_at](https://www.affymetrix.com/LinkServlet?probeset=1450049_a_at) | [Hira](http://www.ncbi.nlm.nih.gov/entrez/query.fcgi?cmd=search&db=gene&term=Hira) | histone cell cycle regulation defective homolog A (S. cerevisiae) |
| 83 | 6,30E-06 | 0,00145 | 5,31 | 2231,03 | 0,0024 | [1425241_a_at](https://www.affymetrix.com/LinkServlet?probeset=1425241_a_at) | [Wsb1](http://www.ncbi.nlm.nih.gov/entrez/query.fcgi?cmd=search&db=gene&term=Wsb1) | WD repeat and SOCS box-containing 1 |
| 84 | 6,30E-06 | 0,00145 | 4522,7 | 877,92 | 5,15 | [1417240_at](https://www.affymetrix.com/LinkServlet?probeset=1417240_at) | [Zyx](http://www.ncbi.nlm.nih.gov/entrez/query.fcgi?cmd=search&db=gene&term=Zyx) | zyxin |
| 85 | 6,40E-06 | 0,00145 | 550,13 | 8,31 | 66,21 | [AFFX-r2-Ec-bioB-5_at](https://www.affymetrix.com/LinkServlet?probeset=AFFX-r2-Ec-bioB-5_at) | [NA](http://www.ncbi.nlm.nih.gov/entrez/query.fcgi?cmd=search&db=gene&term=NA) | NA |
| 86 | 6,40E-06 | 0,00145 | 481,72 | 261,5 | 1,84 | [1434686_at](https://www.affymetrix.com/LinkServlet?probeset=1434686_at) | [Fam48a](http://www.ncbi.nlm.nih.gov/entrez/query.fcgi?cmd=search&db=gene&term=Fam48a) | family with sequence similarity 48, member A |
| 87 | 6,60E-06 | 0,00145 | 63,34 | 222,83 | 0,28 | [1438771_at](https://www.affymetrix.com/LinkServlet?probeset=1438771_at) | [Brd1](http://www.ncbi.nlm.nih.gov/entrez/query.fcgi?cmd=search&db=gene&term=Brd1) | bromodomain containing 1 |
| 88 | 6,70E-06 | 0,00145 | 295,37 | 1734,48 | 0,17 | [1436983_at](https://www.affymetrix.com/LinkServlet?probeset=1436983_at) | [Crebbp](http://www.ncbi.nlm.nih.gov/entrez/query.fcgi?cmd=search&db=gene&term=Crebbp) | CREB binding protein |
| 89 | 6,70E-06 | 0,00145 | 12,92 | 24,35 | 0,53 | [1429162_at](https://www.affymetrix.com/LinkServlet?probeset=1429162_at) | [1500015A07Rik](http://www.ncbi.nlm.nih.gov/entrez/query.fcgi?cmd=search&db=gene&term=1500015A07Rik) | RIKEN cDNA 1500015A07 gene |
| 90 | 6,70E-06 | 0,00145 | 12551,77 | 1981,11 | 6,34 | [1416240_at](https://www.affymetrix.com/LinkServlet?probeset=1416240_at) | [Psmb7](http://www.ncbi.nlm.nih.gov/entrez/query.fcgi?cmd=search&db=gene&term=Psmb7) | proteasome (prosome, macropain) subunit, beta type 7 |
| 91 | 6,90E-06 | 0,00145 | 1497,66 | 4451,09 | 0,34 | [1420170_at](https://www.affymetrix.com/LinkServlet?probeset=1420170_at) | [Myh9](http://www.ncbi.nlm.nih.gov/entrez/query.fcgi?cmd=search&db=gene&term=Myh9) | myosin, heavy polypeptide 9, non-muscle |
| 92 | 6,90E-06 | 0,00145 | 8,45 | 201,58 | 0,042 | [1420498_a_at](https://www.affymetrix.com/LinkServlet?probeset=1420498_a_at) | [Dab2](http://www.ncbi.nlm.nih.gov/entrez/query.fcgi?cmd=search&db=gene&term=Dab2) | disabled homolog 2 (Drosophila) |
| 93 | 6,90E-06 | 0,00145 | 76,22 | 208,01 | 0,37 | [1417261_at](https://www.affymetrix.com/LinkServlet?probeset=1417261_at) | [Mbtd1](http://www.ncbi.nlm.nih.gov/entrez/query.fcgi?cmd=search&db=gene&term=Mbtd1) | mbt domain containing 1 |
| 94 | 7,00E-06 | 0,00145 | 4,78 | 404,36 | 0,012 | [1440315_at](https://www.affymetrix.com/LinkServlet?probeset=1440315_at) | [Mbnl1](http://www.ncbi.nlm.nih.gov/entrez/query.fcgi?cmd=search&db=gene&term=Mbnl1) | muscleblind-like 1 (Drosophila) |
| 95 | 7,00E-06 | 0,00145 | 5,05 | 298,3 | 0,017 | [1426300_at](https://www.affymetrix.com/LinkServlet?probeset=1426300_at) | [Alcam](http://www.ncbi.nlm.nih.gov/entrez/query.fcgi?cmd=search&db=gene&term=Alcam) | activated leukocyte cell adhesion molecule |
| 96 | 7,20E-06 | 0,00148 | 5,88 | 22,08 | 0,27 | [1420449_at](https://www.affymetrix.com/LinkServlet?probeset=1420449_at) | [Heatr5b](http://www.ncbi.nlm.nih.gov/entrez/query.fcgi?cmd=search&db=gene&term=Heatr5b) | HEAT repeat containing 5B |
| 97 | 7,70E-06 | 0,00155 | 106,21 | 27,2 | 3,9 | [1422969_s_at](https://www.affymetrix.com/LinkServlet?probeset=1422969_s_at) | [Ip6k1](http://www.ncbi.nlm.nih.gov/entrez/query.fcgi?cmd=search&db=gene&term=Ip6k1) | inositol hexaphosphate kinase 1 |
| 98 | 7,80E-06 | 0,00155 | 6,96 | 499,96 | 0,014 | [1449007_at](https://www.affymetrix.com/LinkServlet?probeset=1449007_at) | [NA](http://www.ncbi.nlm.nih.gov/entrez/query.fcgi?cmd=search&db=gene&term=NA) | NA |
| 99 | 7,90E-06 | 0,00155 | 5,17 | 1245,71 | 0,0041 | [1448919_at](https://www.affymetrix.com/LinkServlet?probeset=1448919_at) | [Cd302](http://www.ncbi.nlm.nih.gov/entrez/query.fcgi?cmd=search&db=gene&term=Cd302) | CD302 antigen |
| 100 | 8,20E-06 | 0,00155 | 32,87 | 214,91 | 0,15 | [1458385_at](https://www.affymetrix.com/LinkServlet?probeset=1458385_at) | [Hspa4l](http://www.ncbi.nlm.nih.gov/entrez/query.fcgi?cmd=search&db=gene&term=Hspa4l) | heat shock protein 4 like |
| 101 | 8,20E-06 | 0,00155 | 3379,15 | 412,36 | 8,19 | [1416521_at](https://www.affymetrix.com/LinkServlet?probeset=1416521_at) | [Sepw1](http://www.ncbi.nlm.nih.gov/entrez/query.fcgi?cmd=search&db=gene&term=Sepw1) | selenoprotein W, muscle 1 |
| 102 | 8,30E-06 | 0,00155 | 5,8 | 2069,26 | 0,0028 | [1452077_at](https://www.affymetrix.com/LinkServlet?probeset=1452077_at) | [Ddx3y](http://www.ncbi.nlm.nih.gov/entrez/query.fcgi?cmd=search&db=gene&term=Ddx3y) | DEAD (Asp-Glu-Ala-Asp) box polypeptide 3, Y-linked |
| 103 | 8,30E-06 | 0,00155 | 14,72 | 382,03 | 0,039 | [1426569_a_at](https://www.affymetrix.com/LinkServlet?probeset=1426569_a_at) | [Frk](http://www.ncbi.nlm.nih.gov/entrez/query.fcgi?cmd=search&db=gene&term=Frk) | fyn-related kinase |
| 104 | 8,30E-06 | 0,00155 | 7,6 | 255,18 | 0,03 | [1427630_x_at](https://www.affymetrix.com/LinkServlet?probeset=1427630_x_at) | [Ceacam1](http://www.ncbi.nlm.nih.gov/entrez/query.fcgi?cmd=search&db=gene&term=Ceacam1) | carcinoembryonic antigen-related cell adhesion molecule 1 |
| 105 | 8,30E-06 | 0,00155 | 580,57 | 203,32 | 2,86 | [1425656_a_at](https://www.affymetrix.com/LinkServlet?probeset=1425656_a_at) | [Baiap2](http://www.ncbi.nlm.nih.gov/entrez/query.fcgi?cmd=search&db=gene&term=Baiap2) | brain-specific angiogenesis inhibitor 1-associated protein 2 |
| 106 | 8,60E-06 | 0,0016 | 5,27 | 197,88 | 0,027 | [1439665_at](https://www.affymetrix.com/LinkServlet?probeset=1439665_at) | [Lpar4](http://www.ncbi.nlm.nih.gov/entrez/query.fcgi?cmd=search&db=gene&term=Lpar4) | lysophosphatidic acid receptor 4 |
| 107 | 9,60E-06 | 0,00172 | 1395,94 | 3657,2 | 0,38 | [1434405_at](https://www.affymetrix.com/LinkServlet?probeset=1434405_at) | [Fnip1](http://www.ncbi.nlm.nih.gov/entrez/query.fcgi?cmd=search&db=gene&term=Fnip1) | folliculin interacting protein 1 |
| 108 | 9,70E-06 | 0,00172 | 61,4 | 7,96 | 7,72 | [1417229_at](https://www.affymetrix.com/LinkServlet?probeset=1417229_at) | [Capn1](http://www.ncbi.nlm.nih.gov/entrez/query.fcgi?cmd=search&db=gene&term=Capn1) | calpain 1 |
| 109 | 9,70E-06 | 0,00172 | 6,06 | 811,3 | 0,0075 | [1416612_at](https://www.affymetrix.com/LinkServlet?probeset=1416612_at) | [Cyp1b1](http://www.ncbi.nlm.nih.gov/entrez/query.fcgi?cmd=search&db=gene&term=Cyp1b1) | cytochrome P450, family 1, subfamily b, polypeptide 1 |
| 110 | 9,70E-06 | 0,00172 | 41,92 | 251,12 | 0,17 | [1438433_at](https://www.affymetrix.com/LinkServlet?probeset=1438433_at) | [Whamm](http://www.ncbi.nlm.nih.gov/entrez/query.fcgi?cmd=search&db=gene&term=Whamm) | WAS protein homolog associated with actin, golgi membranes and microtubules |
| 111 | 9,70E-06 | 0,00172 | 3589,01 | 1388,93 | 2,58 | [1453004_at](https://www.affymetrix.com/LinkServlet?probeset=1453004_at) | [Slc22a23](http://www.ncbi.nlm.nih.gov/entrez/query.fcgi?cmd=search&db=gene&term=Slc22a23) | solute carrier family 22, member 23 |
| 112 | 9,80E-06 | 0,00172 | 10,97 | 1102,38 | 0,01 | [1450397_at](https://www.affymetrix.com/LinkServlet?probeset=1450397_at) | [Mtap1b](http://www.ncbi.nlm.nih.gov/entrez/query.fcgi?cmd=search&db=gene&term=Mtap1b) | microtubule-associated protein 1B |
| 113 | 1,00E-05 | 0,00174 | 1159,01 | 39,29 | 29,5 | [AFFX-r2-Ec-bioB-M_at](https://www.affymetrix.com/LinkServlet?probeset=AFFX-r2-Ec-bioB-M_at) | [NA](http://www.ncbi.nlm.nih.gov/entrez/query.fcgi?cmd=search&db=gene&term=NA) | NA |
| 114 | 1,01E-05 | 0,00174 | 4,43 | 152,91 | 0,029 | [1418412_at](https://www.affymetrix.com/LinkServlet?probeset=1418412_at) | [Tpd52l1](http://www.ncbi.nlm.nih.gov/entrez/query.fcgi?cmd=search&db=gene&term=Tpd52l1) | tumor protein D52-like 1 |
| 115 | 1,11E-05 | 0,0019 | 6,25 | 442,55 | 0,014 | [1420679_a_at](https://www.affymetrix.com/LinkServlet?probeset=1420679_a_at) | [Aig1](http://www.ncbi.nlm.nih.gov/entrez/query.fcgi?cmd=search&db=gene&term=Aig1) | androgen-induced 1 |
| 116 | 1,12E-05 | 0,0019 | 48,75 | 341,12 | 0,14 | [1417310_at](https://www.affymetrix.com/LinkServlet?probeset=1417310_at) | [Tob2](http://www.ncbi.nlm.nih.gov/entrez/query.fcgi?cmd=search&db=gene&term=Tob2) | transducer of ERBB2, 2 |
| 117 | 1,14E-05 | 0,00192 | 4,99 | 168,36 | 0,03 | [1439327_at](https://www.affymetrix.com/LinkServlet?probeset=1439327_at) | [Ccbe1](http://www.ncbi.nlm.nih.gov/entrez/query.fcgi?cmd=search&db=gene&term=Ccbe1) | collagen and calcium binding EGF domains 1 |
| 118 | 1,18E-05 | 0,00195 | 1202,86 | 6,61 | 181,85 | [1425369_a_at](https://www.affymetrix.com/LinkServlet?probeset=1425369_a_at) | [Sox10](http://www.ncbi.nlm.nih.gov/entrez/query.fcgi?cmd=search&db=gene&term=Sox10) | SRY-box containing gene 10 |
| 119 | 1,21E-05 | 0,00195 | 351,42 | 1591,25 | 0,22 | [1452814_at](https://www.affymetrix.com/LinkServlet?probeset=1452814_at) | [Cpne3](http://www.ncbi.nlm.nih.gov/entrez/query.fcgi?cmd=search&db=gene&term=Cpne3) | copine III |
| 120 | 1,21E-05 | 0,00195 | 2989,54 | 1282,12 | 2,33 | [1452173_at](https://www.affymetrix.com/LinkServlet?probeset=1452173_at) | [Hadha](http://www.ncbi.nlm.nih.gov/entrez/query.fcgi?cmd=search&db=gene&term=Hadha) | hydroxyacyl-Coenzyme A dehydrogenase/3-ketoacyl-Coenzyme A thiolase/enoyl-Coenzyme A hydratase (trifunctional protein), alpha subunit |
| 121 | 1,23E-05 | 0,00195 | 5229,66 | 634,88 | 8,24 | [1420619_a_at](https://www.affymetrix.com/LinkServlet?probeset=1420619_a_at) | [Aes](http://www.ncbi.nlm.nih.gov/entrez/query.fcgi?cmd=search&db=gene&term=Aes) | amino-terminal enhancer of split |
| 122 | 1,23E-05 | 0,00195 | 715,71 | 5,84 | 122,47 | [1422565_s_at](https://www.affymetrix.com/LinkServlet?probeset=1422565_s_at) | [Nfic](http://www.ncbi.nlm.nih.gov/entrez/query.fcgi?cmd=search&db=gene&term=Nfic) | nuclear factor I/C |
| 123 | 1,24E-05 | 0,00195 | 1353,49 | 56,78 | 23,84 | [1419313_at](https://www.affymetrix.com/LinkServlet?probeset=1419313_at) | [Ccnt1](http://www.ncbi.nlm.nih.gov/entrez/query.fcgi?cmd=search&db=gene&term=Ccnt1) | cyclin T1 |
| 124 | 1,24E-05 | 0,00195 | 7,02 | 87,69 | 0,08 | [1450494_x_at](https://www.affymetrix.com/LinkServlet?probeset=1450494_x_at) | [Ceacam1](http://www.ncbi.nlm.nih.gov/entrez/query.fcgi?cmd=search&db=gene&term=Ceacam1) | carcinoembryonic antigen-related cell adhesion molecule 1 |
| 125 | 1,24E-05 | 0,00195 | 842,02 | 2208,63 | 0,38 | [1447878_s_at](https://www.affymetrix.com/LinkServlet?probeset=1447878_s_at) | [Fgfrl1](http://www.ncbi.nlm.nih.gov/entrez/query.fcgi?cmd=search&db=gene&term=Fgfrl1) | fibroblast growth factor receptor-like 1 |
| 126 | 1,25E-05 | 0,00195 | 41,2 | 2546,15 | 0,016 | [1428572_at](https://www.affymetrix.com/LinkServlet?probeset=1428572_at) | [Basp1](http://www.ncbi.nlm.nih.gov/entrez/query.fcgi?cmd=search&db=gene&term=Basp1) | brain abundant, membrane attached signal protein 1 |
| 127 | 1,26E-05 | 0,00195 | 160,11 | 324,98 | 0,49 | [1452898_at](https://www.affymetrix.com/LinkServlet?probeset=1452898_at) | [Vps36](http://www.ncbi.nlm.nih.gov/entrez/query.fcgi?cmd=search&db=gene&term=Vps36) | vacuolar protein sorting 36 (yeast) |
| 128 | 1,29E-05 | 0,00198 | 66,52 | 4846,98 | 0,014 | [1434891_at](https://www.affymetrix.com/LinkServlet?probeset=1434891_at) | [Ptgfrn](http://www.ncbi.nlm.nih.gov/entrez/query.fcgi?cmd=search&db=gene&term=Ptgfrn) | prostaglandin F2 receptor negative regulator |
| 129 | 1,32E-05 | 0,00201 | 594,87 | 5153,58 | 0,12 | [1417385_at](https://www.affymetrix.com/LinkServlet?probeset=1417385_at) | [Npepps](http://www.ncbi.nlm.nih.gov/entrez/query.fcgi?cmd=search&db=gene&term=Npepps) | aminopeptidase puromycin sensitive |
| 130 | 1,33E-05 | 0,00201 | 2603,16 | 667,46 | 3,9 | [1415818_at](https://www.affymetrix.com/LinkServlet?probeset=1415818_at) | [Anxa6](http://www.ncbi.nlm.nih.gov/entrez/query.fcgi?cmd=search&db=gene&term=Anxa6) | annexin A6 |
| 131 | 1,38E-05 | 0,00206 | 115,9 | 2631,95 | 0,044 | [1448433_a_at](https://www.affymetrix.com/LinkServlet?probeset=1448433_a_at) | [Pcolce](http://www.ncbi.nlm.nih.gov/entrez/query.fcgi?cmd=search&db=gene&term=Pcolce) | procollagen C-endopeptidase enhancer protein |
| 132 | 1,38E-05 | 0,00206 | 5,73 | 450,25 | 0,013 | [1448818_at](https://www.affymetrix.com/LinkServlet?probeset=1448818_at) | [Wnt5a](http://www.ncbi.nlm.nih.gov/entrez/query.fcgi?cmd=search&db=gene&term=Wnt5a) | wingless-related MMTV integration site 5A |
| 133 | 1,40E-05 | 0,00207 | 4,4 | 362,22 | 0,012 | [1426858_at](https://www.affymetrix.com/LinkServlet?probeset=1426858_at) | [Inhbb](http://www.ncbi.nlm.nih.gov/entrez/query.fcgi?cmd=search&db=gene&term=Inhbb) | inhibin beta-B |
| 134 | 1,41E-05 | 0,00207 | 13,65 | 82,29 | 0,17 | [1425675_s_at](https://www.affymetrix.com/LinkServlet?probeset=1425675_s_at) | [Ceacam1](http://www.ncbi.nlm.nih.gov/entrez/query.fcgi?cmd=search&db=gene&term=Ceacam1) | carcinoembryonic antigen-related cell adhesion molecule 1 |
| 135 | 1,45E-05 | 0,0021 | 3393,65 | 1831,2 | 1,85 | [1460170_at](https://www.affymetrix.com/LinkServlet?probeset=1460170_at) | [Ext2](http://www.ncbi.nlm.nih.gov/entrez/query.fcgi?cmd=search&db=gene&term=Ext2) | exostoses (multiple) 2 |
| 136 | 1,45E-05 | 0,0021 | 315,01 | 10,19 | 30,92 | [AFFX-BioB-5_at](https://www.affymetrix.com/LinkServlet?probeset=AFFX-BioB-5_at) | [NA](http://www.ncbi.nlm.nih.gov/entrez/query.fcgi?cmd=search&db=gene&term=NA) | NA |
| 137 | 1,48E-05 | 0,0021 | 6,73 | 185,68 | 0,036 | [1428415_at](https://www.affymetrix.com/LinkServlet?probeset=1428415_at) | [Rnf113a2](http://www.ncbi.nlm.nih.gov/entrez/query.fcgi?cmd=search&db=gene&term=Rnf113a2) | ring finger protein 113A2 |
| 138 | 1,50E-05 | 0,0021 | 117,19 | 1521,07 | 0,077 | [1454877_at](https://www.affymetrix.com/LinkServlet?probeset=1454877_at) | [Sertad4](http://www.ncbi.nlm.nih.gov/entrez/query.fcgi?cmd=search&db=gene&term=Sertad4) | SERTA domain containing 4 |
| 139 | 1,51E-05 | 0,0021 | 418,35 | 1767,32 | 0,24 | [1434252_at](https://www.affymetrix.com/LinkServlet?probeset=1434252_at) | [Tmcc3](http://www.ncbi.nlm.nih.gov/entrez/query.fcgi?cmd=search&db=gene&term=Tmcc3) | transmembrane and coiled coil domains 3 |
| 140 | 1,53E-05 | 0,0021 | 3403,08 | 2312,35 | 1,47 | [1450414_at](https://www.affymetrix.com/LinkServlet?probeset=1450414_at) | [Pdgfb](http://www.ncbi.nlm.nih.gov/entrez/query.fcgi?cmd=search&db=gene&term=Pdgfb) | platelet derived growth factor, B polypeptide |
| 141 | 1,54E-05 | 0,0021 | 8,83 | 36,45 | 0,24 | [1449141_at](https://www.affymetrix.com/LinkServlet?probeset=1449141_at) | [Fblim1](http://www.ncbi.nlm.nih.gov/entrez/query.fcgi?cmd=search&db=gene&term=Fblim1) | filamin binding LIM protein 1 |
| 142 | 1,54E-05 | 0,0021 | 6058,61 | 260,82 | 23,23 | [1418450_at](https://www.affymetrix.com/LinkServlet?probeset=1418450_at) | [Islr](http://www.ncbi.nlm.nih.gov/entrez/query.fcgi?cmd=search&db=gene&term=Islr) | immunoglobulin superfamily containing leucine-rich repeat |
| 143 | 1,54E-05 | 0,0021 | 926,33 | 4782,28 | 0,19 | [1422864_at](https://www.affymetrix.com/LinkServlet?probeset=1422864_at) | [Runx1](http://www.ncbi.nlm.nih.gov/entrez/query.fcgi?cmd=search&db=gene&term=Runx1) | runt related transcription factor 1 |
| 144 | 1,54E-05 | 0,0021 | 74,26 | 9541,18 | 0,0078 | [1443798_at](https://www.affymetrix.com/LinkServlet?probeset=1443798_at) | [Pik3cd](http://www.ncbi.nlm.nih.gov/entrez/query.fcgi?cmd=search&db=gene&term=Pik3cd) | phosphatidylinositol 3-kinase catalytic delta polypeptide |
| 145 | 1,57E-05 | 0,00213 | 6328,28 | 1963,57 | 3,22 | [1460692_at](https://www.affymetrix.com/LinkServlet?probeset=1460692_at) | [Ehmt2](http://www.ncbi.nlm.nih.gov/entrez/query.fcgi?cmd=search&db=gene&term=Ehmt2) | euchromatic histone lysine N-methyltransferase 2 |
| 146 | 1,59E-05 | 0,00213 | 964,62 | 32,3 | 29,86 | [AFFX-r2-Ec-bioB-3_at](https://www.affymetrix.com/LinkServlet?probeset=AFFX-r2-Ec-bioB-3_at) | [NA](http://www.ncbi.nlm.nih.gov/entrez/query.fcgi?cmd=search&db=gene&term=NA) | NA |
| 147 | 1,59E-05 | 0,00213 | 1309,2 | 584,93 | 2,24 | [1428246_at](https://www.affymetrix.com/LinkServlet?probeset=1428246_at) | [Vps26b](http://www.ncbi.nlm.nih.gov/entrez/query.fcgi?cmd=search&db=gene&term=Vps26b) | vacuolar protein sorting 26 homolog B (yeast) |
| 148 | 1,62E-05 | 0,00215 | 4,72 | 54,18 | 0,087 | [1419758_at](https://www.affymetrix.com/LinkServlet?probeset=1419758_at) | [Abcb1a](http://www.ncbi.nlm.nih.gov/entrez/query.fcgi?cmd=search&db=gene&term=Abcb1a) | ATP-binding cassette, sub-family B (MDR/TAP), member 1A |
| 149 | 1,67E-05 | 0,0022 | 999,5 | 246,15 | 4,06 | [1448956_at](https://www.affymetrix.com/LinkServlet?probeset=1448956_at) | [Stard10](http://www.ncbi.nlm.nih.gov/entrez/query.fcgi?cmd=search&db=gene&term=Stard10) | START domain containing 10 |
| 150 | 1,70E-05 | 0,00223 | 6642,94 | 3247,62 | 2,05 | [1419249_at](https://www.affymetrix.com/LinkServlet?probeset=1419249_at) | [Cdk14](http://www.ncbi.nlm.nih.gov/entrez/query.fcgi?cmd=search&db=gene&term=Cdk14) | cyclin-dependent kinase 14 |
| 151 | 1,74E-05 | 0,00223 | 94,16 | 1473,2 | 0,064 | [1417611_at](https://www.affymetrix.com/LinkServlet?probeset=1417611_at) | [Tmem37](http://www.ncbi.nlm.nih.gov/entrez/query.fcgi?cmd=search&db=gene&term=Tmem37) | transmembrane protein 37 |
| 152 | 1,75E-05 | 0,00223 | 4,4 | 330,26 | 0,013 | [1436790_a_at](https://www.affymetrix.com/LinkServlet?probeset=1436790_a_at) | [Sox11](http://www.ncbi.nlm.nih.gov/entrez/query.fcgi?cmd=search&db=gene&term=Sox11) | SRY-box containing gene 11 |
| 153 | 1,78E-05 | 0,00223 | 1282,94 | 570,12 | 2,25 | [1449333_at](https://www.affymetrix.com/LinkServlet?probeset=1449333_at) | [Sf3a1](http://www.ncbi.nlm.nih.gov/entrez/query.fcgi?cmd=search&db=gene&term=Sf3a1) | splicing factor 3a, subunit 1 |
| 154 | 1,80E-05 | 0,00223 | 186,03 | 1126,57 | 0,17 | [1449419_at](https://www.affymetrix.com/LinkServlet?probeset=1449419_at) | [Dock8](http://www.ncbi.nlm.nih.gov/entrez/query.fcgi?cmd=search&db=gene&term=Dock8) | dedicator of cytokinesis 8 |
| 155 | 1,80E-05 | 0,00223 | 5840 | 2638,62 | 2,21 | [1419536_a_at](https://www.affymetrix.com/LinkServlet?probeset=1419536_a_at) | [Rela](http://www.ncbi.nlm.nih.gov/entrez/query.fcgi?cmd=search&db=gene&term=Rela) | v-rel reticuloendotheliosis viral oncogene homolog A (avian) |
| 156 | 1,80E-05 | 0,00223 | 107,88 | 1378,88 | 0,078 | [1440668_at](https://www.affymetrix.com/LinkServlet?probeset=1440668_at) | [Adamtsl3](http://www.ncbi.nlm.nih.gov/entrez/query.fcgi?cmd=search&db=gene&term=Adamtsl3) | ADAMTS-like 3 |
| 157 | 1,82E-05 | 0,00223 | 1444,73 | 27,82 | 51,93 | [1417440_at](https://www.affymetrix.com/LinkServlet?probeset=1417440_at) | [Arid1a](http://www.ncbi.nlm.nih.gov/entrez/query.fcgi?cmd=search&db=gene&term=Arid1a) | AT rich interactive domain 1A (SWI-like) |
| 158 | 1,82E-05 | 0,00223 | 1452,08 | 111,59 | 13,01 | [1418570_at](https://www.affymetrix.com/LinkServlet?probeset=1418570_at) | [Ncstn](http://www.ncbi.nlm.nih.gov/entrez/query.fcgi?cmd=search&db=gene&term=Ncstn) | nicastrin |
| 159 | 1,85E-05 | 0,00223 | 839,91 | 128,15 | 6,55 | [1417637_a_at](https://www.affymetrix.com/LinkServlet?probeset=1417637_a_at) | [Hmg20b](http://www.ncbi.nlm.nih.gov/entrez/query.fcgi?cmd=search&db=gene&term=Hmg20b) | high mobility group 20 B |
| 160 | 1,89E-05 | 0,00223 | 2674,69 | 14683,01 | 0,18 | [1416041_at](https://www.affymetrix.com/LinkServlet?probeset=1416041_at) | [Sgk1](http://www.ncbi.nlm.nih.gov/entrez/query.fcgi?cmd=search&db=gene&term=Sgk1) | serum/glucocorticoid regulated kinase 1 |
| 161 | 1,89E-05 | 0,00223 | 2687,71 | 1721,01 | 1,56 | [1427258_at](https://www.affymetrix.com/LinkServlet?probeset=1427258_at) | [Trim24](http://www.ncbi.nlm.nih.gov/entrez/query.fcgi?cmd=search&db=gene&term=Trim24) | tripartite motif-containing 24 |
| 162 | 1,91E-05 | 0,00223 | 66,21 | 597,17 | 0,11 | [1450391_a_at](https://www.affymetrix.com/LinkServlet?probeset=1450391_a_at) | [Mgll](http://www.ncbi.nlm.nih.gov/entrez/query.fcgi?cmd=search&db=gene&term=Mgll) | monoglyceride lipase |
| 163 | 1,91E-05 | 0,00223 | 26,42 | 499,44 | 0,053 | [1429489_at](https://www.affymetrix.com/LinkServlet?probeset=1429489_at) | [Rexo1](http://www.ncbi.nlm.nih.gov/entrez/query.fcgi?cmd=search&db=gene&term=Rexo1) | REX1, RNA exonuclease 1 homolog (S. cerevisiae) |
| 164 | 1,91E-05 | 0,00223 | 580,92 | 3924,74 | 0,15 | [1426777_a_at](https://www.affymetrix.com/LinkServlet?probeset=1426777_a_at) | [Wasl](http://www.ncbi.nlm.nih.gov/entrez/query.fcgi?cmd=search&db=gene&term=Wasl) | Wiskott-Aldrich syndrome-like (human) |
| 165 | 1,92E-05 | 0,00223 | 712,48 | 330,41 | 2,16 | [1433538_at](https://www.affymetrix.com/LinkServlet?probeset=1433538_at) | [Marveld1](http://www.ncbi.nlm.nih.gov/entrez/query.fcgi?cmd=search&db=gene&term=Marveld1) | MARVEL (membrane-associating) domain containing 1 |
| 166 | 1,92E-05 | 0,00223 | 1329,74 | 414,34 | 3,21 | [1418136_at](https://www.affymetrix.com/LinkServlet?probeset=1418136_at) | [Tgfb1i1](http://www.ncbi.nlm.nih.gov/entrez/query.fcgi?cmd=search&db=gene&term=Tgfb1i1) | transforming growth factor beta 1 induced transcript 1 |
| 167 | 1,93E-05 | 0,00223 | 4671,99 | 1069,42 | 4,37 | [1433460_at](https://www.affymetrix.com/LinkServlet?probeset=1433460_at) | [Ttc7b](http://www.ncbi.nlm.nih.gov/entrez/query.fcgi?cmd=search&db=gene&term=Ttc7b) | tetratricopeptide repeat domain 7B |
| 168 | 1,93E-05 | 0,00223 | 4,4 | 210,94 | 0,021 | [1426598_at](https://www.affymetrix.com/LinkServlet?probeset=1426598_at) | [Uty](http://www.ncbi.nlm.nih.gov/entrez/query.fcgi?cmd=search&db=gene&term=Uty) | ubiquitously transcribed tetratricopeptide repeat gene, Y chromosome |
| 169 | 1,94E-05 | 0,00223 | 36,36 | 558,24 | 0,065 | [1437544_at](https://www.affymetrix.com/LinkServlet?probeset=1437544_at) | [Fubp1](http://www.ncbi.nlm.nih.gov/entrez/query.fcgi?cmd=search&db=gene&term=Fubp1) | far upstream element (FUSE) binding protein 1 |
| 170 | 1,94E-05 | 0,00223 | 2742,79 | 7676,51 | 0,36 | [1434210_s_at](https://www.affymetrix.com/LinkServlet?probeset=1434210_s_at) | [Lrig1](http://www.ncbi.nlm.nih.gov/entrez/query.fcgi?cmd=search&db=gene&term=Lrig1) | leucine-rich repeats and immunoglobulin-like domains 1 |
| 171 | 1,94E-05 | 0,00223 | 5,09 | 336,5 | 0,015 | [1418733_at](https://www.affymetrix.com/LinkServlet?probeset=1418733_at) | [Twist1](http://www.ncbi.nlm.nih.gov/entrez/query.fcgi?cmd=search&db=gene&term=Twist1) | twist homolog 1 (Drosophila) |
| 172 | 1,98E-05 | 0,00225 | 166,44 | 62,14 | 2,68 | [1416753_at](https://www.affymetrix.com/LinkServlet?probeset=1416753_at) | [Prkar1b](http://www.ncbi.nlm.nih.gov/entrez/query.fcgi?cmd=search&db=gene&term=Prkar1b) | protein kinase, cAMP dependent regulatory, type I beta |
| 173 | 1,99E-05 | 0,00225 | 472,84 | 1334,57 | 0,35 | [1429428_at](https://www.affymetrix.com/LinkServlet?probeset=1429428_at) | [Tcf7l2](http://www.ncbi.nlm.nih.gov/entrez/query.fcgi?cmd=search&db=gene&term=Tcf7l2) | transcription factor 7-like 2, T-cell specific, HMG-box |
| 174 | 2,00E-05 | 0,00225 | 386,21 | 935,46 | 0,41 | [1434256_s_at](https://www.affymetrix.com/LinkServlet?probeset=1434256_s_at) | [Cds2](http://www.ncbi.nlm.nih.gov/entrez/query.fcgi?cmd=search&db=gene&term=Cds2) | CDP-diacylglycerol synthase (phosphatidate cytidylyltransferase) 2 |
| 175 | 2,01E-05 | 0,00225 | 12,41 | 106,06 | 0,12 | [1456629_at](https://www.affymetrix.com/LinkServlet?probeset=1456629_at) | [Kank3](http://www.ncbi.nlm.nih.gov/entrez/query.fcgi?cmd=search&db=gene&term=Kank3) | KN motif and ankyrin repeat domains 3 |
| 176 | 2,01E-05 | 0,00225 | 5,46 | 552,51 | 0,0099 | [1426301_at](https://www.affymetrix.com/LinkServlet?probeset=1426301_at) | [Alcam](http://www.ncbi.nlm.nih.gov/entrez/query.fcgi?cmd=search&db=gene&term=Alcam) | activated leukocyte cell adhesion molecule |
| 177 | 2,03E-05 | 0,00225 | 549,07 | 1513,01 | 0,36 | [1431126_a_at](https://www.affymetrix.com/LinkServlet?probeset=1431126_a_at) | [0610011F06Rik](http://www.ncbi.nlm.nih.gov/entrez/query.fcgi?cmd=search&db=gene&term=0610011F06Rik) | RIKEN cDNA 0610011F06 gene |
| 178 | 2,04E-05 | 0,00225 | 5,67 | 601,39 | 0,0094 | [1437502_x_at](https://www.affymetrix.com/LinkServlet?probeset=1437502_x_at) | [Cd24a](http://www.ncbi.nlm.nih.gov/entrez/query.fcgi?cmd=search&db=gene&term=Cd24a) | CD24a antigen |
| 179 | 2,10E-05 | 0,00229 | 8,64 | 504,92 | 0,017 | [1429432_at](https://www.affymetrix.com/LinkServlet?probeset=1429432_at) | [Prrc2c](http://www.ncbi.nlm.nih.gov/entrez/query.fcgi?cmd=search&db=gene&term=Prrc2c) | proline-rich coiled-coil 2C |
| 180 | 2,10E-05 | 0,00229 | 26,28 | 8,15 | 3,22 | [1426620_at](https://www.affymetrix.com/LinkServlet?probeset=1426620_at) | [Chst10](http://www.ncbi.nlm.nih.gov/entrez/query.fcgi?cmd=search&db=gene&term=Chst10) | carbohydrate sulfotransferase 10 |
| 181 | 2,12E-05 | 0,0023 | 6,7 | 207,38 | 0,032 | [1426892_at](https://www.affymetrix.com/LinkServlet?probeset=1426892_at) | [Utrn](http://www.ncbi.nlm.nih.gov/entrez/query.fcgi?cmd=search&db=gene&term=Utrn) | utrophin |
| 182 | 2,20E-05 | 0,00238 | 732,16 | 273,39 | 2,68 | [1426577_a_at](https://www.affymetrix.com/LinkServlet?probeset=1426577_a_at) | [Lin37](http://www.ncbi.nlm.nih.gov/entrez/query.fcgi?cmd=search&db=gene&term=Lin37) | lin-37 homolog (C. elegans) |
| 183 | 2,24E-05 | 0,0024 | 5,61 | 133,12 | 0,042 | [1425775_at](https://www.affymetrix.com/LinkServlet?probeset=1425775_at) | [Zfp820](http://www.ncbi.nlm.nih.gov/entrez/query.fcgi?cmd=search&db=gene&term=Zfp820) | zinc finger protein 820 |
| 184 | 2,26E-05 | 0,0024 | 6,15 | 376,79 | 0,016 | [1416034_at](https://www.affymetrix.com/LinkServlet?probeset=1416034_at) | [Cd24a](http://www.ncbi.nlm.nih.gov/entrez/query.fcgi?cmd=search&db=gene&term=Cd24a) | CD24a antigen |
| 185 | 2,27E-05 | 0,0024 | 3844,39 | 9229,68 | 0,42 | [1428463_a_at](https://www.affymetrix.com/LinkServlet?probeset=1428463_a_at) | [Ppp2r5e](http://www.ncbi.nlm.nih.gov/entrez/query.fcgi?cmd=search&db=gene&term=Ppp2r5e) | protein phosphatase 2, regulatory subunit B (B56), epsilon isoform |
| 186 | 2,27E-05 | 0,0024 | 584,79 | 5,84 | 100,13 | [1430112_at](https://www.affymetrix.com/LinkServlet?probeset=1430112_at) | [Wdr66](http://www.ncbi.nlm.nih.gov/entrez/query.fcgi?cmd=search&db=gene&term=Wdr66) | WD repeat domain 66 |
| 187 | 2,29E-05 | 0,00241 | 987,07 | 1874,7 | 0,53 | [1428945_at](https://www.affymetrix.com/LinkServlet?probeset=1428945_at) | [Uba6](http://www.ncbi.nlm.nih.gov/entrez/query.fcgi?cmd=search&db=gene&term=Uba6) | ubiquitin-like modifier activating enzyme 6 |
| 188 | 2,33E-05 | 0,00243 | 868,76 | 637,78 | 1,36 | [1433733_a_at](https://www.affymetrix.com/LinkServlet?probeset=1433733_a_at) | [Cry1](http://www.ncbi.nlm.nih.gov/entrez/query.fcgi?cmd=search&db=gene&term=Cry1) | cryptochrome 1 (photolyase-like) |
| 189 | 2,34E-05 | 0,00243 | 8,98 | 170,77 | 0,053 | [1458236_at](https://www.affymetrix.com/LinkServlet?probeset=1458236_at) | [NA](http://www.ncbi.nlm.nih.gov/entrez/query.fcgi?cmd=search&db=gene&term=NA) | NA |
| 190 | 2,37E-05 | 0,00244 | 1757,08 | 5368,29 | 0,33 | [1433768_at](https://www.affymetrix.com/LinkServlet?probeset=1433768_at) | [Palld](http://www.ncbi.nlm.nih.gov/entrez/query.fcgi?cmd=search&db=gene&term=Palld) | palladin, cytoskeletal associated protein |
| 191 | 2,37E-05 | 0,00244 | 4,46 | 317,77 | 0,014 | [1424208_at](https://www.affymetrix.com/LinkServlet?probeset=1424208_at) | [Ptger4](http://www.ncbi.nlm.nih.gov/entrez/query.fcgi?cmd=search&db=gene&term=Ptger4) | prostaglandin E receptor 4 (subtype EP4) |
| 192 | 2,39E-05 | 0,00244 | 21154,23 | 5799,3 | 3,65 | [1449110_at](https://www.affymetrix.com/LinkServlet?probeset=1449110_at) | [Rhob](http://www.ncbi.nlm.nih.gov/entrez/query.fcgi?cmd=search&db=gene&term=Rhob) | ras homolog gene family, member B |
| 193 | 2,42E-05 | 0,00244 | 17943,86 | 7180,64 | 2,5 | [1448590_at](https://www.affymetrix.com/LinkServlet?probeset=1448590_at) | [Col6a1](http://www.ncbi.nlm.nih.gov/entrez/query.fcgi?cmd=search&db=gene&term=Col6a1) | collagen, type VI, alpha 1 |
| 194 | 2,42E-05 | 0,00244 | 302,92 | 155,31 | 1,95 | [1424145_at](https://www.affymetrix.com/LinkServlet?probeset=1424145_at) | [Prr3](http://www.ncbi.nlm.nih.gov/entrez/query.fcgi?cmd=search&db=gene&term=Prr3) | proline-rich polypeptide 3 |
| 195 | 2,42E-05 | 0,00244 | 1346,49 | 167,04 | 8,06 | [1423816_at](https://www.affymetrix.com/LinkServlet?probeset=1423816_at) | [NA](http://www.ncbi.nlm.nih.gov/entrez/query.fcgi?cmd=search&db=gene&term=NA) | NA |
| 196 | 2,45E-05 | 0,00246 | 1064,19 | 544,05 | 1,96 | [1451108_at](https://www.affymetrix.com/LinkServlet?probeset=1451108_at) | [Rnf185](http://www.ncbi.nlm.nih.gov/entrez/query.fcgi?cmd=search&db=gene&term=Rnf185) | ring finger protein 185 |
| 197 | 2,47E-05 | 0,00246 | 5642,06 | 2880,02 | 1,96 | [1450054_at](https://www.affymetrix.com/LinkServlet?probeset=1450054_at) | [Add1](http://www.ncbi.nlm.nih.gov/entrez/query.fcgi?cmd=search&db=gene&term=Add1) | adducin 1 (alpha) |
| 198 | 2,48E-05 | 0,00246 | 21,62 | 340,51 | 0,063 | [1419477_at](https://www.affymetrix.com/LinkServlet?probeset=1419477_at) | [Clec2d](http://www.ncbi.nlm.nih.gov/entrez/query.fcgi?cmd=search&db=gene&term=Clec2d) | C-type lectin domain family 2, member d |
| 199 | 2,49E-05 | 0,00246 | 9,1 | 483,16 | 0,019 | [1449270_at](https://www.affymetrix.com/LinkServlet?probeset=1449270_at) | [Plxdc2](http://www.ncbi.nlm.nih.gov/entrez/query.fcgi?cmd=search&db=gene&term=Plxdc2) | plexin domain containing 2 |
| 200 | 2,52E-05 | 0,00248 | 2842,5 | 1532,39 | 1,85 | [1423370_a_at](https://www.affymetrix.com/LinkServlet?probeset=1423370_a_at) | [Csnk1g2](http://www.ncbi.nlm.nih.gov/entrez/query.fcgi?cmd=search&db=gene&term=Csnk1g2) | casein kinase 1, gamma 2 |
| 201 | 2,61E-05 | 0,00254 | 5,48 | 40,37 | 0,14 | [1442496_at](https://www.affymetrix.com/LinkServlet?probeset=1442496_at) | [Cdkl3](http://www.ncbi.nlm.nih.gov/entrez/query.fcgi?cmd=search&db=gene&term=Cdkl3) | cyclin-dependent kinase-like 3 |
| 202 | 2,61E-05 | 0,00254 | 7,46 | 71,06 | 0,1 | [1416154_at](https://www.affymetrix.com/LinkServlet?probeset=1416154_at) | [NA](http://www.ncbi.nlm.nih.gov/entrez/query.fcgi?cmd=search&db=gene&term=NA) | NA |
| 203 | 2,62E-05 | 0,00254 | 177,93 | 352,75 | 0,5 | [1418915_at](https://www.affymetrix.com/LinkServlet?probeset=1418915_at) | [Mmachc](http://www.ncbi.nlm.nih.gov/entrez/query.fcgi?cmd=search&db=gene&term=Mmachc) | methylmalonic aciduria cblC type, with homocystinuria |
| 204 | 2,65E-05 | 0,00255 | 4,57 | 64,66 | 0,071 | [1440290_at](https://www.affymetrix.com/LinkServlet?probeset=1440290_at) | [Gm10010](http://www.ncbi.nlm.nih.gov/entrez/query.fcgi?cmd=search&db=gene&term=Gm10010) | predicted gene 10010 |
| 205 | 2,84E-05 | 0,00272 | 6,51 | 56,31 | 0,12 | [1433909_at](https://www.affymetrix.com/LinkServlet?probeset=1433909_at) | [Syt17](http://www.ncbi.nlm.nih.gov/entrez/query.fcgi?cmd=search&db=gene&term=Syt17) | synaptotagmin XVII |
| 206 | 2,88E-05 | 0,00275 | 95,69 | 560,05 | 0,17 | [1437424_at](https://www.affymetrix.com/LinkServlet?probeset=1437424_at) | [Syde2](http://www.ncbi.nlm.nih.gov/entrez/query.fcgi?cmd=search&db=gene&term=Syde2) | synapse defective 1, Rho GTPase, homolog 2 (C. elegans) |
| 207 | 2,90E-05 | 0,00276 | 15,4 | 485,35 | 0,032 | [1423066_at](https://www.affymetrix.com/LinkServlet?probeset=1423066_at) | [Dnmt3a](http://www.ncbi.nlm.nih.gov/entrez/query.fcgi?cmd=search&db=gene&term=Dnmt3a) | DNA methyltransferase 3A |
| 208 | 2,93E-05 | 0,00277 | 5715,01 | 10053,76 | 0,57 | [1438176_x_at](https://www.affymetrix.com/LinkServlet?probeset=1438176_x_at) | [Snap47](http://www.ncbi.nlm.nih.gov/entrez/query.fcgi?cmd=search&db=gene&term=Snap47) | synaptosomal-associated protein, 47 |
| 209 | 2,99E-05 | 0,00281 | 5,03 | 54,9 | 0,092 | [1440314_at](https://www.affymetrix.com/LinkServlet?probeset=1440314_at) | [NA](http://www.ncbi.nlm.nih.gov/entrez/query.fcgi?cmd=search&db=gene&term=NA) | NA |
| 210 | 3,06E-05 | 0,00285 | 4,4 | 79,68 | 0,055 | [1450684_at](https://www.affymetrix.com/LinkServlet?probeset=1450684_at) | [Etv1](http://www.ncbi.nlm.nih.gov/entrez/query.fcgi?cmd=search&db=gene&term=Etv1) | ets variant gene 1 |
| 211 | 3,07E-05 | 0,00285 | 5,78 | 173,77 | 0,033 | [1422139_at](https://www.affymetrix.com/LinkServlet?probeset=1422139_at) | [Plau](http://www.ncbi.nlm.nih.gov/entrez/query.fcgi?cmd=search&db=gene&term=Plau) | plasminogen activator, urokinase |
| 212 | 3,08E-05 | 0,00285 | 647,06 | 1435,29 | 0,45 | [1428421_a_at](https://www.affymetrix.com/LinkServlet?probeset=1428421_a_at) | [Glod4](http://www.ncbi.nlm.nih.gov/entrez/query.fcgi?cmd=search&db=gene&term=Glod4) | glyoxalase domain containing 4 |
| 213 | 3,10E-05 | 0,00285 | 1821,52 | 882,87 | 2,06 | [1453013_at](https://www.affymetrix.com/LinkServlet?probeset=1453013_at) | [Zfp740](http://www.ncbi.nlm.nih.gov/entrez/query.fcgi?cmd=search&db=gene&term=Zfp740) | zinc finger protein 740 |
| 214 | 3,12E-05 | 0,00285 | 5,84 | 244,25 | 0,024 | [1421358_at](https://www.affymetrix.com/LinkServlet?probeset=1421358_at) | [H2-M3](http://www.ncbi.nlm.nih.gov/entrez/query.fcgi?cmd=search&db=gene&term=H2-M3) | histocompatibility 2, M region locus 3 |
| 215 | 3,12E-05 | 0,00285 | 522,51 | 24,52 | 21,31 | [1437733_at](https://www.affymetrix.com/LinkServlet?probeset=1437733_at) | [Eif4ebp2](http://www.ncbi.nlm.nih.gov/entrez/query.fcgi?cmd=search&db=gene&term=Eif4ebp2) | eukaryotic translation initiation factor 4E binding protein 2 |
| 216 | 3,13E-05 | 0,00285 | 843,83 | 1960,65 | 0,43 | [1449090_a_at](https://www.affymetrix.com/LinkServlet?probeset=1449090_a_at) | [Yes1](http://www.ncbi.nlm.nih.gov/entrez/query.fcgi?cmd=search&db=gene&term=Yes1) | Yamaguchi sarcoma viral (v-yes) oncogene homolog 1 |
| 217 | 3,16E-05 | 0,00286 | 3086,13 | 11170,57 | 0,28 | [1437149_at](https://www.affymetrix.com/LinkServlet?probeset=1437149_at) | [Slc6a6](http://www.ncbi.nlm.nih.gov/entrez/query.fcgi?cmd=search&db=gene&term=Slc6a6) | solute carrier family 6 (neurotransmitter transporter, taurine), member 6 |
| 218 | 3,19E-05 | 0,00287 | 1822,57 | 4348,18 | 0,42 | [1429514_at](https://www.affymetrix.com/LinkServlet?probeset=1429514_at) | [Ppap2b](http://www.ncbi.nlm.nih.gov/entrez/query.fcgi?cmd=search&db=gene&term=Ppap2b) | phosphatidic acid phosphatase type 2B |
| 219 | 3,20E-05 | 0,00287 | 214,07 | 462,01 | 0,46 | [1452310_at](https://www.affymetrix.com/LinkServlet?probeset=1452310_at) | [Tada2a](http://www.ncbi.nlm.nih.gov/entrez/query.fcgi?cmd=search&db=gene&term=Tada2a) | transcriptional adaptor 2A |
| 220 | 3,27E-05 | 0,00292 | 157,72 | 35,1 | 4,49 | [1460433_at](https://www.affymetrix.com/LinkServlet?probeset=1460433_at) | [Entpd6](http://www.ncbi.nlm.nih.gov/entrez/query.fcgi?cmd=search&db=gene&term=Entpd6) | ectonucleoside triphosphate diphosphohydrolase 6 |
| 221 | 3,30E-05 | 0,00294 | 138,55 | 379,02 | 0,37 | [1456904_at](https://www.affymetrix.com/LinkServlet?probeset=1456904_at) | [NA](http://www.ncbi.nlm.nih.gov/entrez/query.fcgi?cmd=search&db=gene&term=NA) | NA |
| 222 | 3,32E-05 | 0,00294 | 67,47 | 7,14 | 9,45 | [1433493_at](https://www.affymetrix.com/LinkServlet?probeset=1433493_at) | [Fam73b](http://www.ncbi.nlm.nih.gov/entrez/query.fcgi?cmd=search&db=gene&term=Fam73b) | family with sequence similarity 73, member B |
| 223 | 3,39E-05 | 0,00299 | 25,66 | 554,3 | 0,046 | [1437104_at](https://www.affymetrix.com/LinkServlet?probeset=1437104_at) | [Arfgef1](http://www.ncbi.nlm.nih.gov/entrez/query.fcgi?cmd=search&db=gene&term=Arfgef1) | ADP-ribosylation factor guanine nucleotide-exchange factor 1(brefeldin A-inhibited) |
| 224 | 3,43E-05 | 0,003 | 578,55 | 1571,01 | 0,37 | [1455820_x_at](https://www.affymetrix.com/LinkServlet?probeset=1455820_x_at) | [Scarb1](http://www.ncbi.nlm.nih.gov/entrez/query.fcgi?cmd=search&db=gene&term=Scarb1) | scavenger receptor class B, member 1 |
| 225 | 3,43E-05 | 0,003 | 6,96 | 201,47 | 0,035 | [1437190_at](https://www.affymetrix.com/LinkServlet?probeset=1437190_at) | [Styk1](http://www.ncbi.nlm.nih.gov/entrez/query.fcgi?cmd=search&db=gene&term=Styk1) | serine/threonine/tyrosine kinase 1 |
| 226 | 3,46E-05 | 0,003 | 1206,05 | 3235,81 | 0,37 | [1416123_at](https://www.affymetrix.com/LinkServlet?probeset=1416123_at) | [Ccnd2](http://www.ncbi.nlm.nih.gov/entrez/query.fcgi?cmd=search&db=gene&term=Ccnd2) | cyclin D2 |
| 227 | 3,46E-05 | 0,003 | 345,7 | 98,05 | 3,53 | [1434994_at](https://www.affymetrix.com/LinkServlet?probeset=1434994_at) | [Dedd](http://www.ncbi.nlm.nih.gov/entrez/query.fcgi?cmd=search&db=gene&term=Dedd) | death effector domain-containing |
| 228 | 3,52E-05 | 0,00302 | 864,21 | 2059,81 | 0,42 | [1437378_x_at](https://www.affymetrix.com/LinkServlet?probeset=1437378_x_at) | [Scarb1](http://www.ncbi.nlm.nih.gov/entrez/query.fcgi?cmd=search&db=gene&term=Scarb1) | scavenger receptor class B, member 1 |
| 229 | 3,52E-05 | 0,00302 | 48,36 | 157,43 | 0,31 | [1435098_at](https://www.affymetrix.com/LinkServlet?probeset=1435098_at) | [NA](http://www.ncbi.nlm.nih.gov/entrez/query.fcgi?cmd=search&db=gene&term=NA) | NA |
| 230 | 3,55E-05 | 0,00303 | 25,69 | 7075,6 | 0,0036 | [1455961_at](https://www.affymetrix.com/LinkServlet?probeset=1455961_at) | [NA](http://www.ncbi.nlm.nih.gov/entrez/query.fcgi?cmd=search&db=gene&term=NA) | NA |
| 231 | 3,56E-05 | 0,00303 | 3615,56 | 1258,3 | 2,87 | [1423128_at](https://www.affymetrix.com/LinkServlet?probeset=1423128_at) | [Aip](http://www.ncbi.nlm.nih.gov/entrez/query.fcgi?cmd=search&db=gene&term=Aip) | aryl-hydrocarbon receptor-interacting protein |
| 232 | 3,58E-05 | 0,00303 | 904,68 | 2064,06 | 0,44 | [1433940_at](https://www.affymetrix.com/LinkServlet?probeset=1433940_at) | [Spag7](http://www.ncbi.nlm.nih.gov/entrez/query.fcgi?cmd=search&db=gene&term=Spag7) | sperm associated antigen 7 |
| 233 | 3,60E-05 | 0,00304 | 13469,47 | 4028,2 | 3,34 | [1417423_at](https://www.affymetrix.com/LinkServlet?probeset=1417423_at) | [Grina](http://www.ncbi.nlm.nih.gov/entrez/query.fcgi?cmd=search&db=gene&term=Grina) | glutamate receptor, ionotropic, N-methyl D-aspartate-associated protein 1 (glutamate binding) |
| 234 | 3,67E-05 | 0,00308 | 4378,31 | 808,06 | 5,42 | [1433541_a_at](https://www.affymetrix.com/LinkServlet?probeset=1433541_a_at) | [Ubap2l](http://www.ncbi.nlm.nih.gov/entrez/query.fcgi?cmd=search&db=gene&term=Ubap2l) | ubiquitin associated protein 2-like |
| 235 | 3,70E-05 | 0,0031 | 6,97 | 89,84 | 0,078 | [1435931_at](https://www.affymetrix.com/LinkServlet?probeset=1435931_at) | [NA](http://www.ncbi.nlm.nih.gov/entrez/query.fcgi?cmd=search&db=gene&term=NA) | NA |
| 236 | 3,72E-05 | 0,0031 | 4,89 | 142,62 | 0,034 | [1434582_at](https://www.affymetrix.com/LinkServlet?probeset=1434582_at) | [Erc2](http://www.ncbi.nlm.nih.gov/entrez/query.fcgi?cmd=search&db=gene&term=Erc2) | ELKS/RAB6-interacting/CAST family member 2 |
| 237 | 3,73E-05 | 0,0031 | 11,38 | 216,98 | 0,052 | [1423277_at](https://www.affymetrix.com/LinkServlet?probeset=1423277_at) | [Ptprk](http://www.ncbi.nlm.nih.gov/entrez/query.fcgi?cmd=search&db=gene&term=Ptprk) | protein tyrosine phosphatase, receptor type, K |
| 238 | 3,75E-05 | 0,0031 | 287,57 | 4,75 | 60,55 | [1456379_x_at](https://www.affymetrix.com/LinkServlet?probeset=1456379_x_at) | [Dner](http://www.ncbi.nlm.nih.gov/entrez/query.fcgi?cmd=search&db=gene&term=Dner) | delta/notch-like EGF-related receptor |
| 239 | 3,84E-05 | 0,00315 | 8,02 | 31,91 | 0,25 | [1440448_at](https://www.affymetrix.com/LinkServlet?probeset=1440448_at) | [NA](http://www.ncbi.nlm.nih.gov/entrez/query.fcgi?cmd=search&db=gene&term=NA) | NA |
| 240 | 3,84E-05 | 0,00315 | 746,29 | 2966,77 | 0,25 | [1425488_at](https://www.affymetrix.com/LinkServlet?probeset=1425488_at) | [Slu7](http://www.ncbi.nlm.nih.gov/entrez/query.fcgi?cmd=search&db=gene&term=Slu7) | SLU7 splicing factor homolog (S. cerevisiae) |
| 241 | 3,86E-05 | 0,00315 | 713,98 | 333,52 | 2,14 | [1433498_at](https://www.affymetrix.com/LinkServlet?probeset=1433498_at) | [Urgcp](http://www.ncbi.nlm.nih.gov/entrez/query.fcgi?cmd=search&db=gene&term=Urgcp) | upregulator of cell proliferation |
| 242 | 3,89E-05 | 0,00315 | 31,69 | 345,63 | 0,092 | [1419289_a_at](https://www.affymetrix.com/LinkServlet?probeset=1419289_a_at) | [Syngr1](http://www.ncbi.nlm.nih.gov/entrez/query.fcgi?cmd=search&db=gene&term=Syngr1) | synaptogyrin 1 |
| 243 | 3,89E-05 | 0,00315 | 371,4 | 1530,31 | 0,24 | [1436247_at](https://www.affymetrix.com/LinkServlet?probeset=1436247_at) | [NA](http://www.ncbi.nlm.nih.gov/entrez/query.fcgi?cmd=search&db=gene&term=NA) | NA |
| 244 | 3,97E-05 | 0,00317 | 506,45 | 6754,07 | 0,075 | [1424967_x_at](https://www.affymetrix.com/LinkServlet?probeset=1424967_x_at) | [Tnnt2](http://www.ncbi.nlm.nih.gov/entrez/query.fcgi?cmd=search&db=gene&term=Tnnt2) | troponin T2, cardiac |
| 245 | 3,97E-05 | 0,00317 | 884,53 | 65,49 | 13,51 | [1451147_x_at](https://www.affymetrix.com/LinkServlet?probeset=1451147_x_at) | [Csdc2](http://www.ncbi.nlm.nih.gov/entrez/query.fcgi?cmd=search&db=gene&term=Csdc2) | cold shock domain containing C2, RNA binding |
| 246 | 3,99E-05 | 0,00317 | 273,02 | 5,74 | 47,55 | [1450576_a_at](https://www.affymetrix.com/LinkServlet?probeset=1450576_a_at) | [Sf3a2](http://www.ncbi.nlm.nih.gov/entrez/query.fcgi?cmd=search&db=gene&term=Sf3a2) | splicing factor 3a, subunit 2 |
| 247 | 3,99E-05 | 0,00317 | 12226,05 | 6172,7 | 1,98 | [1419554_at](https://www.affymetrix.com/LinkServlet?probeset=1419554_at) | [Cd47](http://www.ncbi.nlm.nih.gov/entrez/query.fcgi?cmd=search&db=gene&term=Cd47) | CD47 antigen (Rh-related antigen, integrin-associated signal transducer) |
| 248 | 4,00E-05 | 0,00317 | 7,14 | 158,93 | 0,045 | [1448926_at](https://www.affymetrix.com/LinkServlet?probeset=1448926_at) | [Hoxa5](http://www.ncbi.nlm.nih.gov/entrez/query.fcgi?cmd=search&db=gene&term=Hoxa5) | homeobox A5 |
| 249 | 4,06E-05 | 0,00319 | 1084,38 | 444,5 | 2,44 | [1429473_at](https://www.affymetrix.com/LinkServlet?probeset=1429473_at) | [Zfp869](http://www.ncbi.nlm.nih.gov/entrez/query.fcgi?cmd=search&db=gene&term=Zfp869) | zinc finger protein 869 |
| 250 | 4,09E-05 | 0,00319 | 6,24 | 43,21 | 0,14 | [1455948_x_at](https://www.affymetrix.com/LinkServlet?probeset=1455948_x_at) | [Matn3](http://www.ncbi.nlm.nih.gov/entrez/query.fcgi?cmd=search&db=gene&term=Matn3) | matrilin 3 |
| 251 | 4,09E-05 | 0,00319 | 14,61 | 125,07 | 0,12 | [1426570_a_at](https://www.affymetrix.com/LinkServlet?probeset=1426570_a_at) | [Frk](http://www.ncbi.nlm.nih.gov/entrez/query.fcgi?cmd=search&db=gene&term=Frk) | fyn-related kinase |
| 252 | 4,09E-05 | 0,00319 | 130,18 | 173,91 | 0,75 | [1429317_at](https://www.affymetrix.com/LinkServlet?probeset=1429317_at) | [Qrsl1](http://www.ncbi.nlm.nih.gov/entrez/query.fcgi?cmd=search&db=gene&term=Qrsl1) | glutaminyl-tRNA synthase (glutamine-hydrolyzing)-like 1 |
| 253 | 4,13E-05 | 0,0032 | 26402,63 | 2997,81 | 8,81 | [1423669_at](https://www.affymetrix.com/LinkServlet?probeset=1423669_at) | [Col1a1](http://www.ncbi.nlm.nih.gov/entrez/query.fcgi?cmd=search&db=gene&term=Col1a1) | collagen, type I, alpha 1 |
| 254 | 4,14E-05 | 0,0032 | 1736,91 | 670,65 | 2,59 | [1437532_at](https://www.affymetrix.com/LinkServlet?probeset=1437532_at) | [Rnf216](http://www.ncbi.nlm.nih.gov/entrez/query.fcgi?cmd=search&db=gene&term=Rnf216) | ring finger protein 216 |
| 255 | 4,15E-05 | 0,0032 | 1702,91 | 490,01 | 3,48 | [1415907_at](https://www.affymetrix.com/LinkServlet?probeset=1415907_at) | [Ccnd3](http://www.ncbi.nlm.nih.gov/entrez/query.fcgi?cmd=search&db=gene&term=Ccnd3) | cyclin D3 |
| 256 | 4,22E-05 | 0,00324 | 4,46 | 415,71 | 0,011 | [1436293_x_at](https://www.affymetrix.com/LinkServlet?probeset=1436293_x_at) | [Ildr2](http://www.ncbi.nlm.nih.gov/entrez/query.fcgi?cmd=search&db=gene&term=Ildr2) | immunoglobulin-like domain containing receptor 2 |
| 257 | 4,23E-05 | 0,00324 | 7,35 | 169,13 | 0,043 | [1455796_x_at](https://www.affymetrix.com/LinkServlet?probeset=1455796_x_at) | [Olfm1](http://www.ncbi.nlm.nih.gov/entrez/query.fcgi?cmd=search&db=gene&term=Olfm1) | olfactomedin 1 |
| 258 | 4,31E-05 | 0,00329 | 5,81 | 4,41 | 1,32 | [1450867_at](https://www.affymetrix.com/LinkServlet?probeset=1450867_at) | [Mrpl17](http://www.ncbi.nlm.nih.gov/entrez/query.fcgi?cmd=search&db=gene&term=Mrpl17) | mitochondrial ribosomal protein L17 |
| 259 | 4,35E-05 | 0,0033 | 957,47 | 235,34 | 4,07 | [1452174_at](https://www.affymetrix.com/LinkServlet?probeset=1452174_at) | [Srebf2](http://www.ncbi.nlm.nih.gov/entrez/query.fcgi?cmd=search&db=gene&term=Srebf2) | sterol regulatory element binding factor 2 |
| 260 | 4,37E-05 | 0,00331 | 8,86 | 452,94 | 0,02 | [1437594_x_at](https://www.affymetrix.com/LinkServlet?probeset=1437594_x_at) | [Pigt](http://www.ncbi.nlm.nih.gov/entrez/query.fcgi?cmd=search&db=gene&term=Pigt) | phosphatidylinositol glycan anchor biosynthesis, class T |
| 261 | 4,49E-05 | 0,00338 | 499,36 | 105,24 | 4,75 | [1435083_at](https://www.affymetrix.com/LinkServlet?probeset=1435083_at) | [Ctxn1](http://www.ncbi.nlm.nih.gov/entrez/query.fcgi?cmd=search&db=gene&term=Ctxn1) | cortexin 1 |
| 262 | 4,56E-05 | 0,00342 | 1051,7 | 1660,81 | 0,63 | [1434821_at](https://www.affymetrix.com/LinkServlet?probeset=1434821_at) | [Brd1](http://www.ncbi.nlm.nih.gov/entrez/query.fcgi?cmd=search&db=gene&term=Brd1) | bromodomain containing 1 |
| 263 | 4,61E-05 | 0,00345 | 287,19 | 541,18 | 0,53 | [1437198_at](https://www.affymetrix.com/LinkServlet?probeset=1437198_at) | [Lig3](http://www.ncbi.nlm.nih.gov/entrez/query.fcgi?cmd=search&db=gene&term=Lig3) | ligase III, DNA, ATP-dependent |
| 264 | 4,70E-05 | 0,00349 | 3482,71 | 908,48 | 3,83 | [1416315_at](https://www.affymetrix.com/LinkServlet?probeset=1416315_at) | [Abhd4](http://www.ncbi.nlm.nih.gov/entrez/query.fcgi?cmd=search&db=gene&term=Abhd4) | abhydrolase domain containing 4 |
| 265 | 4,73E-05 | 0,00349 | 56,44 | 388,71 | 0,15 | [1437950_at](https://www.affymetrix.com/LinkServlet?probeset=1437950_at) | [Fam149a](http://www.ncbi.nlm.nih.gov/entrez/query.fcgi?cmd=search&db=gene&term=Fam149a) | family with sequence similarity 149, member A |
| 266 | 4,73E-05 | 0,00349 | 6,78 | 214 | 0,032 | [1442569_at](https://www.affymetrix.com/LinkServlet?probeset=1442569_at) | [NA](http://www.ncbi.nlm.nih.gov/entrez/query.fcgi?cmd=search&db=gene&term=NA) | NA |
| 267 | 4,76E-05 | 0,00349 | 6,12 | 81,26 | 0,075 | [1437129_at](https://www.affymetrix.com/LinkServlet?probeset=1437129_at) | [E330018D03Rik](http://www.ncbi.nlm.nih.gov/entrez/query.fcgi?cmd=search&db=gene&term=E330018D03Rik) | RIKEN cDNA E330018D03 gene |
| 268 | 4,77E-05 | 0,00349 | 80,05 | 26,84 | 2,98 | [1436710_at](https://www.affymetrix.com/LinkServlet?probeset=1436710_at) | [Zswim4](http://www.ncbi.nlm.nih.gov/entrez/query.fcgi?cmd=search&db=gene&term=Zswim4) | zinc finger, SWIM domain containing 4 |
| 269 | 4,78E-05 | 0,00349 | 2211,92 | 3979,09 | 0,56 | [1424106_at](https://www.affymetrix.com/LinkServlet?probeset=1424106_at) | [Jkamp](http://www.ncbi.nlm.nih.gov/entrez/query.fcgi?cmd=search&db=gene&term=Jkamp) | JNK1/MAPK8-associated membrane protein |
| 270 | 4,85E-05 | 0,00353 | 882,79 | 456,09 | 1,94 | [1416206_at](https://www.affymetrix.com/LinkServlet?probeset=1416206_at) | [Sipa1](http://www.ncbi.nlm.nih.gov/entrez/query.fcgi?cmd=search&db=gene&term=Sipa1) | signal-induced proliferation associated gene 1 |
| 271 | 4,90E-05 | 0,00356 | 12082,52 | 5240,45 | 2,31 | [1416406_at](https://www.affymetrix.com/LinkServlet?probeset=1416406_at) | [Pea15a](http://www.ncbi.nlm.nih.gov/entrez/query.fcgi?cmd=search&db=gene&term=Pea15a) | phosphoprotein enriched in astrocytes 15A |
| 272 | 4,95E-05 | 0,00358 | 198,94 | 75,86 | 2,62 | [1424297_at](https://www.affymetrix.com/LinkServlet?probeset=1424297_at) | [Zfp282](http://www.ncbi.nlm.nih.gov/entrez/query.fcgi?cmd=search&db=gene&term=Zfp282) | zinc finger protein 282 |
| 273 | 5,02E-05 | 0,00361 | 847,65 | 110,93 | 7,64 | [1423267_s_at](https://www.affymetrix.com/LinkServlet?probeset=1423267_s_at) | [Itga5](http://www.ncbi.nlm.nih.gov/entrez/query.fcgi?cmd=search&db=gene&term=Itga5) | integrin alpha 5 (fibronectin receptor alpha) |
| 274 | 5,10E-05 | 0,00361 | 1006,13 | 170,54 | 5,9 | [1424064_at](https://www.affymetrix.com/LinkServlet?probeset=1424064_at) | [Rab1b](http://www.ncbi.nlm.nih.gov/entrez/query.fcgi?cmd=search&db=gene&term=Rab1b) | RAB1B, member RAS oncogene family |
| 275 | 5,11E-05 | 0,00361 | 2267,59 | 625,11 | 3,63 | [1438883_at](https://www.affymetrix.com/LinkServlet?probeset=1438883_at) | [Fgf5](http://www.ncbi.nlm.nih.gov/entrez/query.fcgi?cmd=search&db=gene&term=Fgf5) | fibroblast growth factor 5 |
| 276 | 5,11E-05 | 0,00361 | 4,58 | 402,25 | 0,011 | [1438861_at](https://www.affymetrix.com/LinkServlet?probeset=1438861_at) | [Bnc2](http://www.ncbi.nlm.nih.gov/entrez/query.fcgi?cmd=search&db=gene&term=Bnc2) | basonuclin 2 |
| 277 | 5,12E-05 | 0,00361 | 21,97 | 203,56 | 0,11 | [1445723_at](https://www.affymetrix.com/LinkServlet?probeset=1445723_at) | [Plcl1](http://www.ncbi.nlm.nih.gov/entrez/query.fcgi?cmd=search&db=gene&term=Plcl1) | phospholipase C-like 1 |
| 278 | 5,12E-05 | 0,00361 | 329,1 | 1411,61 | 0,23 | [1416046_a_at](https://www.affymetrix.com/LinkServlet?probeset=1416046_a_at) | [Fuca2](http://www.ncbi.nlm.nih.gov/entrez/query.fcgi?cmd=search&db=gene&term=Fuca2) | fucosidase, alpha-L- 2, plasma |
| 279 | 5,16E-05 | 0,00361 | 369,23 | 7,23 | 51,1 | [AFFX-BioB-3_at](https://www.affymetrix.com/LinkServlet?probeset=AFFX-BioB-3_at) | [NA](http://www.ncbi.nlm.nih.gov/entrez/query.fcgi?cmd=search&db=gene&term=NA) | NA |
| 280 | 5,16E-05 | 0,00361 | 530,56 | 1420,27 | 0,37 | [1422859_a_at](https://www.affymetrix.com/LinkServlet?probeset=1422859_a_at) | [Rpl23](http://www.ncbi.nlm.nih.gov/entrez/query.fcgi?cmd=search&db=gene&term=Rpl23) | ribosomal protein L23 |
| 281 | 5,17E-05 | 0,00361 | 6,63 | 47,02 | 0,14 | [1437439_at](https://www.affymetrix.com/LinkServlet?probeset=1437439_at) | [2410089E03Rik](http://www.ncbi.nlm.nih.gov/entrez/query.fcgi?cmd=search&db=gene&term=2410089E03Rik) | RIKEN cDNA 2410089E03 gene |
| 282 | 5,18E-05 | 0,00361 | 148,29 | 441,95 | 0,34 | [1422924_at](https://www.affymetrix.com/LinkServlet?probeset=1422924_at) | [Tnfsf9](http://www.ncbi.nlm.nih.gov/entrez/query.fcgi?cmd=search&db=gene&term=Tnfsf9) | tumor necrosis factor (ligand) superfamily, member 9 |
| 283 | 5,23E-05 | 0,00363 | 387,75 | 6,74 | 57,54 | [1448147_at](https://www.affymetrix.com/LinkServlet?probeset=1448147_at) | [Tnfrsf19](http://www.ncbi.nlm.nih.gov/entrez/query.fcgi?cmd=search&db=gene&term=Tnfrsf19) | tumor necrosis factor receptor superfamily, member 19 |
| 284 | 5,24E-05 | 0,00363 | 4,9 | 85,44 | 0,057 | [1441961_at](https://www.affymetrix.com/LinkServlet?probeset=1441961_at) | [Mtap9](http://www.ncbi.nlm.nih.gov/entrez/query.fcgi?cmd=search&db=gene&term=Mtap9) | microtubule-associated protein 9 |
| 285 | 5,33E-05 | 0,00368 | 22,71 | 5,67 | 4 | [1424032_at](https://www.affymetrix.com/LinkServlet?probeset=1424032_at) | [Hvcn1](http://www.ncbi.nlm.nih.gov/entrez/query.fcgi?cmd=search&db=gene&term=Hvcn1) | hydrogen voltage-gated channel 1 |
| 286 | 5,36E-05 | 0,00369 | 2830,77 | 640,81 | 4,42 | [1436332_at](https://www.affymetrix.com/LinkServlet?probeset=1436332_at) | [Hspb6](http://www.ncbi.nlm.nih.gov/entrez/query.fcgi?cmd=search&db=gene&term=Hspb6) | heat shock protein, alpha-crystallin-related, B6 |
| 287 | 5,43E-05 | 0,00369 | 3054,11 | 948,02 | 3,22 | [1415755_a_at](https://www.affymetrix.com/LinkServlet?probeset=1415755_a_at) | [Ube2v1](http://www.ncbi.nlm.nih.gov/entrez/query.fcgi?cmd=search&db=gene&term=Ube2v1) | ubiquitin-conjugating enzyme E2 variant 1 |
| 288 | 5,44E-05 | 0,00369 | 166,09 | 8,3 | 20,02 | [1416853_at](https://www.affymetrix.com/LinkServlet?probeset=1416853_at) | [Ncdn](http://www.ncbi.nlm.nih.gov/entrez/query.fcgi?cmd=search&db=gene&term=Ncdn) | neurochondrin |
| 289 | 5,45E-05 | 0,00369 | 99,98 | 28,99 | 3,45 | [1452376_at](https://www.affymetrix.com/LinkServlet?probeset=1452376_at) | [Zfp444](http://www.ncbi.nlm.nih.gov/entrez/query.fcgi?cmd=search&db=gene&term=Zfp444) | zinc finger protein 444 |
| 290 | 5,47E-05 | 0,00369 | 3616,63 | 2300,87 | 1,57 | [1452675_at](https://www.affymetrix.com/LinkServlet?probeset=1452675_at) | [Rbm22](http://www.ncbi.nlm.nih.gov/entrez/query.fcgi?cmd=search&db=gene&term=Rbm22) | RNA binding motif protein 22 |
| 291 | 5,49E-05 | 0,00369 | 769,55 | 10230,71 | 0,075 | [1418726_a_at](https://www.affymetrix.com/LinkServlet?probeset=1418726_a_at) | [Tnnt2](http://www.ncbi.nlm.nih.gov/entrez/query.fcgi?cmd=search&db=gene&term=Tnnt2) | troponin T2, cardiac |
| 292 | 5,50E-05 | 0,00369 | 300,74 | 3055,17 | 0,098 | [1436505_at](https://www.affymetrix.com/LinkServlet?probeset=1436505_at) | [Ppig](http://www.ncbi.nlm.nih.gov/entrez/query.fcgi?cmd=search&db=gene&term=Ppig) | peptidyl-prolyl isomerase G (cyclophilin G) |
| 293 | 5,54E-05 | 0,00369 | 59,46 | 329,09 | 0,18 | [1422852_at](https://www.affymetrix.com/LinkServlet?probeset=1422852_at) | [Cib2](http://www.ncbi.nlm.nih.gov/entrez/query.fcgi?cmd=search&db=gene&term=Cib2) | calcium and integrin binding family member 2 |
| 294 | 5,54E-05 | 0,00369 | 6,26 | 125,61 | 0,05 | [1449306_at](https://www.affymetrix.com/LinkServlet?probeset=1449306_at) | [Hsf2](http://www.ncbi.nlm.nih.gov/entrez/query.fcgi?cmd=search&db=gene&term=Hsf2) | heat shock factor 2 |
| 295 | 5,54E-05 | 0,00369 | 4,87 | 78,49 | 0,062 | [1418500_at](https://www.affymetrix.com/LinkServlet?probeset=1418500_at) | [Nap1l3](http://www.ncbi.nlm.nih.gov/entrez/query.fcgi?cmd=search&db=gene&term=Nap1l3) | nucleosome assembly protein 1-like 3 |
| 296 | 5,55E-05 | 0,00369 | 843,47 | 2445,13 | 0,34 | [1435589_at](https://www.affymetrix.com/LinkServlet?probeset=1435589_at) | [Ccdc85b](http://www.ncbi.nlm.nih.gov/entrez/query.fcgi?cmd=search&db=gene&term=Ccdc85b) | coiled-coil domain containing 85B |
| 297 | 5,57E-05 | 0,00369 | 135,34 | 5,5 | 24,63 | [1417163_at](https://www.affymetrix.com/LinkServlet?probeset=1417163_at) | [Dusp10](http://www.ncbi.nlm.nih.gov/entrez/query.fcgi?cmd=search&db=gene&term=Dusp10) | dual specificity phosphatase 10 |
| 298 | 5,59E-05 | 0,00369 | 26 | 250,55 | 0,1 | [1451860_a_at](https://www.affymetrix.com/LinkServlet?probeset=1451860_a_at) | [Trim30a](http://www.ncbi.nlm.nih.gov/entrez/query.fcgi?cmd=search&db=gene&term=Trim30a) | tripartite motif-containing 30A |
| 299 | 5,61E-05 | 0,00369 | 13,14 | 301,75 | 0,044 | [1421471_at](https://www.affymetrix.com/LinkServlet?probeset=1421471_at) | [Npy1r](http://www.ncbi.nlm.nih.gov/entrez/query.fcgi?cmd=search&db=gene&term=Npy1r) | neuropeptide Y receptor Y1 |
| 300 | 5,65E-05 | 0,00369 | 2933,66 | 859,11 | 3,41 | [1449069_at](https://www.affymetrix.com/LinkServlet?probeset=1449069_at) | [Zfp148](http://www.ncbi.nlm.nih.gov/entrez/query.fcgi?cmd=search&db=gene&term=Zfp148) | zinc finger protein 148 |
| 301 | 5,66E-05 | 0,00369 | 1249,15 | 311,93 | 4 | [1422597_at](https://www.affymetrix.com/LinkServlet?probeset=1422597_at) | [Mmp15](http://www.ncbi.nlm.nih.gov/entrez/query.fcgi?cmd=search&db=gene&term=Mmp15) | matrix metallopeptidase 15 |
| 302 | 5,66E-05 | 0,00369 | 332,22 | 1031,19 | 0,32 | [1424634_at](https://www.affymetrix.com/LinkServlet?probeset=1424634_at) | [Tceal1](http://www.ncbi.nlm.nih.gov/entrez/query.fcgi?cmd=search&db=gene&term=Tceal1) | transcription elongation factor A (SII)-like 1 |
| 303 | 5,77E-05 | 0,00375 | 1059,92 | 283,47 | 3,74 | [1451465_at](https://www.affymetrix.com/LinkServlet?probeset=1451465_at) | [Ubl7](http://www.ncbi.nlm.nih.gov/entrez/query.fcgi?cmd=search&db=gene&term=Ubl7) | ubiquitin-like 7 (bone marrow stromal cell-derived) |
| 304 | 5,79E-05 | 0,00375 | 69,16 | 13,06 | 5,3 | [1418341_at](https://www.affymetrix.com/LinkServlet?probeset=1418341_at) | [Rab4a](http://www.ncbi.nlm.nih.gov/entrez/query.fcgi?cmd=search&db=gene&term=Rab4a) | RAB4A, member RAS oncogene family |
| 305 | 5,84E-05 | 0,00377 | 236,35 | 443,51 | 0,53 | [1421077_at](https://www.affymetrix.com/LinkServlet?probeset=1421077_at) | [Sertad3](http://www.ncbi.nlm.nih.gov/entrez/query.fcgi?cmd=search&db=gene&term=Sertad3) | SERTA domain containing 3 |
| 306 | 5,91E-05 | 0,0038 | 15284,1 | 32029,5 | 0,48 | [1437457_a_at](https://www.affymetrix.com/LinkServlet?probeset=1437457_a_at) | [Mtpn](http://www.ncbi.nlm.nih.gov/entrez/query.fcgi?cmd=search&db=gene&term=Mtpn) | myotrophin |
| 307 | 5,97E-05 | 0,00382 | 4002,17 | 459,67 | 8,71 | [1416769_s_at](https://www.affymetrix.com/LinkServlet?probeset=1416769_s_at) | [Atp6v0b](http://www.ncbi.nlm.nih.gov/entrez/query.fcgi?cmd=search&db=gene&term=Atp6v0b) | ATPase, H+ transporting, lysosomal V0 subunit B |
| 308 | 6,13E-05 | 0,00391 | 5,88 | 24,32 | 0,24 | [1421016_at](https://www.affymetrix.com/LinkServlet?probeset=1421016_at) | [Ighmbp2](http://www.ncbi.nlm.nih.gov/entrez/query.fcgi?cmd=search&db=gene&term=Ighmbp2) | immunoglobulin mu binding protein 2 |
| 309 | 6,20E-05 | 0,00395 | 386,27 | 71,33 | 5,42 | [1422268_a_at](https://www.affymetrix.com/LinkServlet?probeset=1422268_a_at) | [Rps6kb2](http://www.ncbi.nlm.nih.gov/entrez/query.fcgi?cmd=search&db=gene&term=Rps6kb2) | ribosomal protein S6 kinase, polypeptide 2 |
| 310 | 6,24E-05 | 0,00396 | 6,01 | 30,57 | 0,2 | [1425464_at](https://www.affymetrix.com/LinkServlet?probeset=1425464_at) | [Gata6](http://www.ncbi.nlm.nih.gov/entrez/query.fcgi?cmd=search&db=gene&term=Gata6) | GATA binding protein 6 |
| 311 | 6,30E-05 | 0,00398 | 665,79 | 269,01 | 2,47 | [1424054_at](https://www.affymetrix.com/LinkServlet?probeset=1424054_at) | [Btbd2](http://www.ncbi.nlm.nih.gov/entrez/query.fcgi?cmd=search&db=gene&term=Btbd2) | BTB (POZ) domain containing 2 |
| 312 | 6,33E-05 | 0,00398 | 10027,18 | 4989,53 | 2,01 | [1416438_at](https://www.affymetrix.com/LinkServlet?probeset=1416438_at) | [Puf60](http://www.ncbi.nlm.nih.gov/entrez/query.fcgi?cmd=search&db=gene&term=Puf60) | poly-U binding splicing factor 60 |
| 313 | 6,36E-05 | 0,00398 | 1258,87 | 1845,17 | 0,68 | [1451714_a_at](https://www.affymetrix.com/LinkServlet?probeset=1451714_a_at) | [Map2k3](http://www.ncbi.nlm.nih.gov/entrez/query.fcgi?cmd=search&db=gene&term=Map2k3) | mitogen-activated protein kinase kinase 3 |
| 314 | 6,39E-05 | 0,00398 | 2169,34 | 816,01 | 2,66 | [1416914_s_at](https://www.affymetrix.com/LinkServlet?probeset=1416914_s_at) | [Fam89b](http://www.ncbi.nlm.nih.gov/entrez/query.fcgi?cmd=search&db=gene&term=Fam89b) | family with sequence similarity 89, member B |
| 315 | 6,41E-05 | 0,00398 | 269,13 | 598,82 | 0,45 | [1438396_at](https://www.affymetrix.com/LinkServlet?probeset=1438396_at) | [Ocrl](http://www.ncbi.nlm.nih.gov/entrez/query.fcgi?cmd=search&db=gene&term=Ocrl) | oculocerebrorenal syndrome of Lowe |
| 316 | 6,41E-05 | 0,00398 | 1554,29 | 2857,05 | 0,54 | [1448564_at](https://www.affymetrix.com/LinkServlet?probeset=1448564_at) | [Cib1](http://www.ncbi.nlm.nih.gov/entrez/query.fcgi?cmd=search&db=gene&term=Cib1) | calcium and integrin binding 1 (calmyrin) |
| 317 | 6,41E-05 | 0,00398 | 8,61 | 63,28 | 0,14 | [1425556_at](https://www.affymetrix.com/LinkServlet?probeset=1425556_at) | [Cdk12](http://www.ncbi.nlm.nih.gov/entrez/query.fcgi?cmd=search&db=gene&term=Cdk12) | cyclin-dependent kinase 12 |
| 318 | 6,46E-05 | 0,004 | 6,43 | 1867,74 | 0,0034 | [1426439_at](https://www.affymetrix.com/LinkServlet?probeset=1426439_at) | [Ddx3y](http://www.ncbi.nlm.nih.gov/entrez/query.fcgi?cmd=search&db=gene&term=Ddx3y) | DEAD (Asp-Glu-Ala-Asp) box polypeptide 3, Y-linked |
| 319 | 6,53E-05 | 0,00401 | 463,42 | 106,04 | 4,37 | [1425356_at](https://www.affymetrix.com/LinkServlet?probeset=1425356_at) | [Zfp142](http://www.ncbi.nlm.nih.gov/entrez/query.fcgi?cmd=search&db=gene&term=Zfp142) | zinc finger protein 142 |
| 320 | 6,53E-05 | 0,00401 | 1779,91 | 614,27 | 2,9 | [1450106_a_at](https://www.affymetrix.com/LinkServlet?probeset=1450106_a_at) | [Evl](http://www.ncbi.nlm.nih.gov/entrez/query.fcgi?cmd=search&db=gene&term=Evl) | Ena-vasodilator stimulated phosphoprotein |
| 321 | 6,56E-05 | 0,00402 | 1258,59 | 3210,74 | 0,39 | [1446560_at](https://www.affymetrix.com/LinkServlet?probeset=1446560_at) | [Prss23](http://www.ncbi.nlm.nih.gov/entrez/query.fcgi?cmd=search&db=gene&term=Prss23) | protease, serine, 23 |
| 322 | 6,60E-05 | 0,00402 | 571,22 | 59,22 | 9,65 | [1448913_at](https://www.affymetrix.com/LinkServlet?probeset=1448913_at) | [Smarcd1](http://www.ncbi.nlm.nih.gov/entrez/query.fcgi?cmd=search&db=gene&term=Smarcd1) | SWI/SNF related, matrix associated, actin dependent regulator of chromatin, subfamily d, member 1 |
| 323 | 6,62E-05 | 0,00402 | 1747,03 | 239,51 | 7,29 | [1448164_at](https://www.affymetrix.com/LinkServlet?probeset=1448164_at) | [Klhdc3](http://www.ncbi.nlm.nih.gov/entrez/query.fcgi?cmd=search&db=gene&term=Klhdc3) | kelch domain containing 3 |
| 324 | 6,63E-05 | 0,00402 | 3431,71 | 308,47 | 11,12 | [1416644_a_at](https://www.affymetrix.com/LinkServlet?probeset=1416644_a_at) | [Sema3b](http://www.ncbi.nlm.nih.gov/entrez/query.fcgi?cmd=search&db=gene&term=Sema3b) | sema domain, immunoglobulin domain (Ig), short basic domain, secreted, (semaphorin) 3B |
| 325 | 6,67E-05 | 0,00404 | 2517,18 | 964,8 | 2,61 | [1422854_at](https://www.affymetrix.com/LinkServlet?probeset=1422854_at) | [Shc1](http://www.ncbi.nlm.nih.gov/entrez/query.fcgi?cmd=search&db=gene&term=Shc1) | src homology 2 domain-containing transforming protein C1 |
| 326 | 6,70E-05 | 0,00404 | 1061,98 | 1803,15 | 0,59 | [1416410_at](https://www.affymetrix.com/LinkServlet?probeset=1416410_at) | [Pafah1b3](http://www.ncbi.nlm.nih.gov/entrez/query.fcgi?cmd=search&db=gene&term=Pafah1b3) | platelet-activating factor acetylhydrolase, isoform 1b, subunit 3 |
| 327 | 6,75E-05 | 0,00405 | 996,15 | 1810,14 | 0,55 | [1436019_a_at](https://www.affymetrix.com/LinkServlet?probeset=1436019_a_at) | [Trappc1](http://www.ncbi.nlm.nih.gov/entrez/query.fcgi?cmd=search&db=gene&term=Trappc1) | trafficking protein particle complex 1 |
| 328 | 6,77E-05 | 0,00405 | 521,24 | 2961,49 | 0,18 | [1455381_at](https://www.affymetrix.com/LinkServlet?probeset=1455381_at) | [4921513D23Rik](http://www.ncbi.nlm.nih.gov/entrez/query.fcgi?cmd=search&db=gene&term=4921513D23Rik) | RIKEN cDNA 4921513D23 gene |
| 329 | 6,78E-05 | 0,00405 | 107,44 | 745,43 | 0,14 | [1447364_x_at](https://www.affymetrix.com/LinkServlet?probeset=1447364_x_at) | [Myo1b](http://www.ncbi.nlm.nih.gov/entrez/query.fcgi?cmd=search&db=gene&term=Myo1b) | myosin IB |
| 330 | 6,86E-05 | 0,00406 | 4,7 | 95,24 | 0,049 | [1417214_at](https://www.affymetrix.com/LinkServlet?probeset=1417214_at) | [Rab27b](http://www.ncbi.nlm.nih.gov/entrez/query.fcgi?cmd=search&db=gene&term=Rab27b) | RAB27b, member RAS oncogene family |
| 331 | 6,87E-05 | 0,00406 | 105,57 | 34,56 | 3,05 | [1452372_at](https://www.affymetrix.com/LinkServlet?probeset=1452372_at) | [Bsdc1](http://www.ncbi.nlm.nih.gov/entrez/query.fcgi?cmd=search&db=gene&term=Bsdc1) | BSD domain containing 1 |
| 332 | 6,89E-05 | 0,00406 | 9,75 | 79,74 | 0,12 | [1440649_at](https://www.affymetrix.com/LinkServlet?probeset=1440649_at) | [Gm4876](http://www.ncbi.nlm.nih.gov/entrez/query.fcgi?cmd=search&db=gene&term=Gm4876) | predicted gene 4876 |
| 333 | 6,91E-05 | 0,00406 | 30,32 | 5,59 | 5,42 | [1453190_at](https://www.affymetrix.com/LinkServlet?probeset=1453190_at) | [Usp19](http://www.ncbi.nlm.nih.gov/entrez/query.fcgi?cmd=search&db=gene&term=Usp19) | ubiquitin specific peptidase 19 |
| 334 | 6,92E-05 | 0,00406 | 22,31 | 4,98 | 4,48 | [1451905_a_at](https://www.affymetrix.com/LinkServlet?probeset=1451905_a_at) | [Mx1](http://www.ncbi.nlm.nih.gov/entrez/query.fcgi?cmd=search&db=gene&term=Mx1) | myxovirus (influenza virus) resistance 1 |
| 335 | 6,92E-05 | 0,00406 | 16711,32 | 10450,45 | 1,6 | [1416958_at](https://www.affymetrix.com/LinkServlet?probeset=1416958_at) | [Nr1d2](http://www.ncbi.nlm.nih.gov/entrez/query.fcgi?cmd=search&db=gene&term=Nr1d2) | nuclear receptor subfamily 1, group D, member 2 |
| 336 | 7,03E-05 | 0,0041 | 74,32 | 643,9 | 0,12 | [1459679_s_at](https://www.affymetrix.com/LinkServlet?probeset=1459679_s_at) | [Myo1b](http://www.ncbi.nlm.nih.gov/entrez/query.fcgi?cmd=search&db=gene&term=Myo1b) | myosin IB |
| 337 | 7,04E-05 | 0,0041 | 31,01 | 4,47 | 6,94 | [1449165_at](https://www.affymetrix.com/LinkServlet?probeset=1449165_at) | [Capn5](http://www.ncbi.nlm.nih.gov/entrez/query.fcgi?cmd=search&db=gene&term=Capn5) | calpain 5 |
| 338 | 7,07E-05 | 0,0041 | 85,07 | 202,55 | 0,42 | [1435089_at](https://www.affymetrix.com/LinkServlet?probeset=1435089_at) | [2010111I01Rik](http://www.ncbi.nlm.nih.gov/entrez/query.fcgi?cmd=search&db=gene&term=2010111I01Rik) | RIKEN cDNA 2010111I01 gene |
| 339 | 7,07E-05 | 0,0041 | 4,4 | 11,54 | 0,38 | [1458823_at](https://www.affymetrix.com/LinkServlet?probeset=1458823_at) | [NA](http://www.ncbi.nlm.nih.gov/entrez/query.fcgi?cmd=search&db=gene&term=NA) | NA |
| 340 | 7,12E-05 | 0,00411 | 605,41 | 12,2 | 49,62 | [1419416_a_at](https://www.affymetrix.com/LinkServlet?probeset=1419416_a_at) | [Rarg](http://www.ncbi.nlm.nih.gov/entrez/query.fcgi?cmd=search&db=gene&term=Rarg) | retinoic acid receptor, gamma |
| 341 | 7,14E-05 | 0,00411 | 17,31 | 455,93 | 0,038 | [1435521_at](https://www.affymetrix.com/LinkServlet?probeset=1435521_at) | [Msi2](http://www.ncbi.nlm.nih.gov/entrez/query.fcgi?cmd=search&db=gene&term=Msi2) | Musashi homolog 2 (Drosophila) |
| 342 | 7,15E-05 | 0,00411 | 354,35 | 14,33 | 24,72 | [1423007_a_at](https://www.affymetrix.com/LinkServlet?probeset=1423007_a_at) | [Gfra2](http://www.ncbi.nlm.nih.gov/entrez/query.fcgi?cmd=search&db=gene&term=Gfra2) | glial cell line derived neurotrophic factor family receptor alpha 2 |
| 343 | 7,23E-05 | 0,00413 | 1016,29 | 228,93 | 4,44 | [1424667_a_at](https://www.affymetrix.com/LinkServlet?probeset=1424667_a_at) | [Cux1](http://www.ncbi.nlm.nih.gov/entrez/query.fcgi?cmd=search&db=gene&term=Cux1) | cut-like homeobox 1 |
| 344 | 7,25E-05 | 0,00413 | 2478,43 | 1356,63 | 1,83 | [1454616_at](https://www.affymetrix.com/LinkServlet?probeset=1454616_at) | [Ubr7](http://www.ncbi.nlm.nih.gov/entrez/query.fcgi?cmd=search&db=gene&term=Ubr7) | ubiquitin protein ligase E3 component n-recognin 7 (putative) |
| 345 | 7,26E-05 | 0,00413 | 4789,69 | 555,92 | 8,62 | [1449054_a_at](https://www.affymetrix.com/LinkServlet?probeset=1449054_a_at) | [Pcbp4](http://www.ncbi.nlm.nih.gov/entrez/query.fcgi?cmd=search&db=gene&term=Pcbp4) | poly(rC) binding protein 4 |
| 346 | 7,27E-05 | 0,00413 | 175,62 | 373,54 | 0,47 | [1427939_s_at](https://www.affymetrix.com/LinkServlet?probeset=1427939_s_at) | [Mycbp](http://www.ncbi.nlm.nih.gov/entrez/query.fcgi?cmd=search&db=gene&term=Mycbp) | c-myc binding protein |
| 347 | 7,37E-05 | 0,00418 | 1681,24 | 336,3 | 5 | [1435965_at](https://www.affymetrix.com/LinkServlet?probeset=1435965_at) | [Cnot3](http://www.ncbi.nlm.nih.gov/entrez/query.fcgi?cmd=search&db=gene&term=Cnot3) | CCR4-NOT transcription complex, subunit 3 |
| 348 | 7,40E-05 | 0,00418 | 7,8 | 76,51 | 0,1 | [1452309_at](https://www.affymetrix.com/LinkServlet?probeset=1452309_at) | [Cgnl1](http://www.ncbi.nlm.nih.gov/entrez/query.fcgi?cmd=search&db=gene&term=Cgnl1) | cingulin-like 1 |
| 349 | 7,43E-05 | 0,00419 | 27,29 | 699,54 | 0,039 | [1439787_at](https://www.affymetrix.com/LinkServlet?probeset=1439787_at) | [P2rx7](http://www.ncbi.nlm.nih.gov/entrez/query.fcgi?cmd=search&db=gene&term=P2rx7) | purinergic receptor P2X, ligand-gated ion channel, 7 |
| 350 | 7,51E-05 | 0,00421 | 1595,31 | 2748,31 | 0,58 | [1437437_x_at](https://www.affymetrix.com/LinkServlet?probeset=1437437_x_at) | [Dnpep](http://www.ncbi.nlm.nih.gov/entrez/query.fcgi?cmd=search&db=gene&term=Dnpep) | aspartyl aminopeptidase |
| 351 | 7,51E-05 | 0,00421 | 70,42 | 677,11 | 0,1 | [1452741_s_at](https://www.affymetrix.com/LinkServlet?probeset=1452741_s_at) | [Gpd2](http://www.ncbi.nlm.nih.gov/entrez/query.fcgi?cmd=search&db=gene&term=Gpd2) | glycerol phosphate dehydrogenase 2, mitochondrial |
| 352 | 7,55E-05 | 0,00422 | 1809,5 | 3801,18 | 0,48 | [1454947_a_at](https://www.affymetrix.com/LinkServlet?probeset=1454947_a_at) | [Ublcp1](http://www.ncbi.nlm.nih.gov/entrez/query.fcgi?cmd=search&db=gene&term=Ublcp1) | ubiquitin-like domain containing CTD phosphatase 1 |
| 353 | 7,59E-05 | 0,00423 | 5,18 | 26,66 | 0,19 | [1431936_a_at](https://www.affymetrix.com/LinkServlet?probeset=1431936_a_at) | [Neu2](http://www.ncbi.nlm.nih.gov/entrez/query.fcgi?cmd=search&db=gene&term=Neu2) | neuraminidase 2 |
| 354 | 7,68E-05 | 0,00427 | 32,8 | 293,5 | 0,11 | [1422803_at](https://www.affymetrix.com/LinkServlet?probeset=1422803_at) | [Fstl3](http://www.ncbi.nlm.nih.gov/entrez/query.fcgi?cmd=search&db=gene&term=Fstl3) | follistatin-like 3 |
| 355 | 7,72E-05 | 0,00428 | 8,01 | 461,61 | 0,017 | [1435283_s_at](https://www.affymetrix.com/LinkServlet?probeset=1435283_s_at) | [Fam189a2](http://www.ncbi.nlm.nih.gov/entrez/query.fcgi?cmd=search&db=gene&term=Fam189a2) | family with sequence similarity 189, member A2 |
| 356 | 7,79E-05 | 0,0043 | 1317,77 | 4690,5 | 0,28 | [1418024_at](https://www.affymetrix.com/LinkServlet?probeset=1418024_at) | [Naa15](http://www.ncbi.nlm.nih.gov/entrez/query.fcgi?cmd=search&db=gene&term=Naa15) | N(alpha)-acetyltransferase 15, NatA auxiliary subunit |
| 357 | 7,89E-05 | 0,00434 | 570,07 | 234,81 | 2,43 | [1456098_a_at](https://www.affymetrix.com/LinkServlet?probeset=1456098_a_at) | [Elmo2](http://www.ncbi.nlm.nih.gov/entrez/query.fcgi?cmd=search&db=gene&term=Elmo2) | engulfment and cell motility 2, ced-12 homolog (C. elegans) |
| 358 | 7,90E-05 | 0,00434 | 1877,37 | 3076,01 | 0,61 | [1443881_at](https://www.affymetrix.com/LinkServlet?probeset=1443881_at) | [Pofut1](http://www.ncbi.nlm.nih.gov/entrez/query.fcgi?cmd=search&db=gene&term=Pofut1) | protein O-fucosyltransferase 1 |
| 359 | 7,92E-05 | 0,00434 | 835,02 | 57,46 | 14,53 | [1423845_at](https://www.affymetrix.com/LinkServlet?probeset=1423845_at) | [Csdc2](http://www.ncbi.nlm.nih.gov/entrez/query.fcgi?cmd=search&db=gene&term=Csdc2) | cold shock domain containing C2, RNA binding |
| 360 | 7,97E-05 | 0,00434 | 620,95 | 1197,58 | 0,52 | [1447780_x_at](https://www.affymetrix.com/LinkServlet?probeset=1447780_x_at) | [Tufm](http://www.ncbi.nlm.nih.gov/entrez/query.fcgi?cmd=search&db=gene&term=Tufm) | Tu translation elongation factor, mitochondrial |
| 361 | 7,98E-05 | 0,00434 | 3229,66 | 2111,31 | 1,53 | [1418693_at](https://www.affymetrix.com/LinkServlet?probeset=1418693_at) | [Hnrnpc](http://www.ncbi.nlm.nih.gov/entrez/query.fcgi?cmd=search&db=gene&term=Hnrnpc) | heterogeneous nuclear ribonucleoprotein C |
| 362 | 7,98E-05 | 0,00434 | 376,17 | 72,72 | 5,17 | [1433931_at](https://www.affymetrix.com/LinkServlet?probeset=1433931_at) | [C030046I01Rik](http://www.ncbi.nlm.nih.gov/entrez/query.fcgi?cmd=search&db=gene&term=C030046I01Rik) | RIKEN cDNA C030046I01 gene |
| 363 | 8,04E-05 | 0,00436 | 477,45 | 2260,74 | 0,21 | [1451501_a_at](https://www.affymetrix.com/LinkServlet?probeset=1451501_a_at) | [Ghr](http://www.ncbi.nlm.nih.gov/entrez/query.fcgi?cmd=search&db=gene&term=Ghr) | growth hormone receptor |
| 364 | 8,07E-05 | 0,00436 | 967,77 | 45,76 | 21,15 | [1427103_at](https://www.affymetrix.com/LinkServlet?probeset=1427103_at) | [Plekho2](http://www.ncbi.nlm.nih.gov/entrez/query.fcgi?cmd=search&db=gene&term=Plekho2) | pleckstrin homology domain containing, family O member 2 |
| 365 | 8,17E-05 | 0,0044 | 14981,24 | 19661,72 | 0,76 | [1417418_s_at](https://www.affymetrix.com/LinkServlet?probeset=1417418_s_at) | [Cox6a1](http://www.ncbi.nlm.nih.gov/entrez/query.fcgi?cmd=search&db=gene&term=Cox6a1) | cytochrome c oxidase, subunit VI a, polypeptide 1 |
| 366 | 8,24E-05 | 0,00442 | 7,32 | 342,43 | 0,021 | [1434909_at](https://www.affymetrix.com/LinkServlet?probeset=1434909_at) | [Rragd](http://www.ncbi.nlm.nih.gov/entrez/query.fcgi?cmd=search&db=gene&term=Rragd) | Ras-related GTP binding D |
| 367 | 8,25E-05 | 0,00442 | 4355,6 | 9857,2 | 0,44 | [1448279_at](https://www.affymetrix.com/LinkServlet?probeset=1448279_at) | [Arpc3](http://www.ncbi.nlm.nih.gov/entrez/query.fcgi?cmd=search&db=gene&term=Arpc3) | actin related protein 2/3 complex, subunit 3 |
| 368 | 8,28E-05 | 0,00442 | 7,41 | 65,39 | 0,11 | [1456524_at](https://www.affymetrix.com/LinkServlet?probeset=1456524_at) | [Nrg1](http://www.ncbi.nlm.nih.gov/entrez/query.fcgi?cmd=search&db=gene&term=Nrg1) | neuregulin 1 |
| 369 | 8,29E-05 | 0,00442 | 510,09 | 250,51 | 2,04 | [1419272_at](https://www.affymetrix.com/LinkServlet?probeset=1419272_at) | [Myd88](http://www.ncbi.nlm.nih.gov/entrez/query.fcgi?cmd=search&db=gene&term=Myd88) | myeloid differentiation primary response gene 88 |
| 370 | 8,35E-05 | 0,00443 | 77,27 | 261,33 | 0,3 | [1424698_s_at](https://www.affymetrix.com/LinkServlet?probeset=1424698_s_at) | [Gca](http://www.ncbi.nlm.nih.gov/entrez/query.fcgi?cmd=search&db=gene&term=Gca) | grancalcin |
| 371 | 8,35E-05 | 0,00443 | 4,4 | 110,27 | 0,04 | [1429372_at](https://www.affymetrix.com/LinkServlet?probeset=1429372_at) | [Sox11](http://www.ncbi.nlm.nih.gov/entrez/query.fcgi?cmd=search&db=gene&term=Sox11) | SRY-box containing gene 11 |
| 372 | 8,42E-05 | 0,00445 | 7,16 | 37,52 | 0,19 | [1444250_at](https://www.affymetrix.com/LinkServlet?probeset=1444250_at) | [NA](http://www.ncbi.nlm.nih.gov/entrez/query.fcgi?cmd=search&db=gene&term=NA) | NA |
| 373 | 8,50E-05 | 0,00446 | 526,86 | 38,16 | 13,81 | [1425680_a_at](https://www.affymetrix.com/LinkServlet?probeset=1425680_a_at) | [Btrc](http://www.ncbi.nlm.nih.gov/entrez/query.fcgi?cmd=search&db=gene&term=Btrc) | beta-transducin repeat containing protein |
| 374 | 8,50E-05 | 0,00446 | 11,85 | 188,41 | 0,063 | [1421354_at](https://www.affymetrix.com/LinkServlet?probeset=1421354_at) | [Prkg2](http://www.ncbi.nlm.nih.gov/entrez/query.fcgi?cmd=search&db=gene&term=Prkg2) | protein kinase, cGMP-dependent, type II |
| 375 | 8,50E-05 | 0,00446 | 465,96 | 66,47 | 7,01 | [1428147_at](https://www.affymetrix.com/LinkServlet?probeset=1428147_at) | [Coro7](http://www.ncbi.nlm.nih.gov/entrez/query.fcgi?cmd=search&db=gene&term=Coro7) | coronin 7 |
| 376 | 8,53E-05 | 0,00446 | 1105,59 | 277,57 | 3,98 | [1425265_a_at](https://www.affymetrix.com/LinkServlet?probeset=1425265_a_at) | [Eri3](http://www.ncbi.nlm.nih.gov/entrez/query.fcgi?cmd=search&db=gene&term=Eri3) | exoribonuclease 3 |
| 377 | 8,69E-05 | 0,00453 | 29141,45 | 12946,16 | 2,25 | [1417490_at](https://www.affymetrix.com/LinkServlet?probeset=1417490_at) | [Ctsb](http://www.ncbi.nlm.nih.gov/entrez/query.fcgi?cmd=search&db=gene&term=Ctsb) | cathepsin B |
| 378 | 8,79E-05 | 0,00457 | 261,18 | 998,67 | 0,26 | [1454734_at](https://www.affymetrix.com/LinkServlet?probeset=1454734_at) | [Lef1](http://www.ncbi.nlm.nih.gov/entrez/query.fcgi?cmd=search&db=gene&term=Lef1) | lymphoid enhancer binding factor 1 |
| 379 | 8,82E-05 | 0,00458 | 1297,22 | 434,78 | 2,98 | [1452777_a_at](https://www.affymetrix.com/LinkServlet?probeset=1452777_a_at) | [Nub1](http://www.ncbi.nlm.nih.gov/entrez/query.fcgi?cmd=search&db=gene&term=Nub1) | negative regulator of ubiquitin-like proteins 1 |
| 380 | 8,84E-05 | 0,00458 | 5,59 | 14,7 | 0,38 | [1434526_at](https://www.affymetrix.com/LinkServlet?probeset=1434526_at) | [Ephx4](http://www.ncbi.nlm.nih.gov/entrez/query.fcgi?cmd=search&db=gene&term=Ephx4) | epoxide hydrolase 4 |
| 381 | 8,93E-05 | 0,00461 | 466,2 | 1066,9 | 0,44 | [1452190_at](https://www.affymetrix.com/LinkServlet?probeset=1452190_at) | [Prcp](http://www.ncbi.nlm.nih.gov/entrez/query.fcgi?cmd=search&db=gene&term=Prcp) | prolylcarboxypeptidase (angiotensinase C) |
| 382 | 9,01E-05 | 0,00464 | 5,07 | 15,69 | 0,32 | [1446127_at](https://www.affymetrix.com/LinkServlet?probeset=1446127_at) | [Zeb1](http://www.ncbi.nlm.nih.gov/entrez/query.fcgi?cmd=search&db=gene&term=Zeb1) | zinc finger E-box binding homeobox 1 |
| 383 | 9,03E-05 | 0,00464 | 5,17 | 38,78 | 0,13 | [1444080_at](https://www.affymetrix.com/LinkServlet?probeset=1444080_at) | [Nav2](http://www.ncbi.nlm.nih.gov/entrez/query.fcgi?cmd=search&db=gene&term=Nav2) | neuron navigator 2 |
| 384 | 9,08E-05 | 0,00464 | 393,56 | 4049,65 | 0,097 | [1448870_at](https://www.affymetrix.com/LinkServlet?probeset=1448870_at) | [Ltbp1](http://www.ncbi.nlm.nih.gov/entrez/query.fcgi?cmd=search&db=gene&term=Ltbp1) | latent transforming growth factor beta binding protein 1 |
| 385 | 9,12E-05 | 0,00464 | 8,87 | 218,61 | 0,041 | [1428209_at](https://www.affymetrix.com/LinkServlet?probeset=1428209_at) | [Bex4](http://www.ncbi.nlm.nih.gov/entrez/query.fcgi?cmd=search&db=gene&term=Bex4) | brain expressed gene 4 |
| 386 | 9,12E-05 | 0,00464 | 5,86 | 31,66 | 0,19 | [1457908_at](https://www.affymetrix.com/LinkServlet?probeset=1457908_at) | [NA](http://www.ncbi.nlm.nih.gov/entrez/query.fcgi?cmd=search&db=gene&term=NA) | NA |
| 387 | 9,13E-05 | 0,00464 | 108,37 | 6,17 | 17,56 | [1428211_at](https://www.affymetrix.com/LinkServlet?probeset=1428211_at) | [P4htm](http://www.ncbi.nlm.nih.gov/entrez/query.fcgi?cmd=search&db=gene&term=P4htm) | prolyl 4-hydroxylase, transmembrane (endoplasmic reticulum) |
| 388 | 9,20E-05 | 0,00466 | 676,96 | 1331,61 | 0,51 | [1431802_a_at](https://www.affymetrix.com/LinkServlet?probeset=1431802_a_at) | [Ept1](http://www.ncbi.nlm.nih.gov/entrez/query.fcgi?cmd=search&db=gene&term=Ept1) | ethanolaminephosphotransferase 1 (CDP-ethanolamine-specific) |
| 389 | 9,23E-05 | 0,00467 | 91,53 | 134,16 | 0,68 | [1450337_a_at](https://www.affymetrix.com/LinkServlet?probeset=1450337_a_at) | [Nek8](http://www.ncbi.nlm.nih.gov/entrez/query.fcgi?cmd=search&db=gene&term=Nek8) | NIMA (never in mitosis gene a)-related expressed kinase 8 |
| 390 | 9,27E-05 | 0,00467 | 5,68 | 43,36 | 0,13 | [1435957_at](https://www.affymetrix.com/LinkServlet?probeset=1435957_at) | [B830032F12](http://www.ncbi.nlm.nih.gov/entrez/query.fcgi?cmd=search&db=gene&term=B830032F12) | hypothetical protein B830032F12 |
| 391 | 9,50E-05 | 0,00475 | 431,05 | 724,83 | 0,59 | [1417739_at](https://www.affymetrix.com/LinkServlet?probeset=1417739_at) | [Med11](http://www.ncbi.nlm.nih.gov/entrez/query.fcgi?cmd=search&db=gene&term=Med11) | mediator of RNA polymerase II transcription, subunit 11 homolog (S. cerevisiae) |
| 392 | 9,51E-05 | 0,00475 | 307,73 | 1054,87 | 0,29 | [1416872_at](https://www.affymetrix.com/LinkServlet?probeset=1416872_at) | [Tspan6](http://www.ncbi.nlm.nih.gov/entrez/query.fcgi?cmd=search&db=gene&term=Tspan6) | tetraspanin 6 |
| 393 | 9,51E-05 | 0,00475 | 1098,68 | 109,78 | 10,01 | [1425679_a_at](https://www.affymetrix.com/LinkServlet?probeset=1425679_a_at) | [Mapk8ip1](http://www.ncbi.nlm.nih.gov/entrez/query.fcgi?cmd=search&db=gene&term=Mapk8ip1) | mitogen-activated protein kinase 8 interacting protein 1 |
| 394 | 9,53E-05 | 0,00475 | 3318,47 | 995,79 | 3,33 | [1460334_at](https://www.affymetrix.com/LinkServlet?probeset=1460334_at) | [Dbnl](http://www.ncbi.nlm.nih.gov/entrez/query.fcgi?cmd=search&db=gene&term=Dbnl) | drebrin-like |
| 395 | 9,56E-05 | 0,00475 | 6,94 | 187,8 | 0,037 | [1441955_s_at](https://www.affymetrix.com/LinkServlet?probeset=1441955_s_at) | [Paip1](http://www.ncbi.nlm.nih.gov/entrez/query.fcgi?cmd=search&db=gene&term=Paip1) | polyadenylate binding protein-interacting protein 1 |
| 396 | 9,65E-05 | 0,00475 | 379,03 | 1143,09 | 0,33 | [1436507_at](https://www.affymetrix.com/LinkServlet?probeset=1436507_at) | [Irak2](http://www.ncbi.nlm.nih.gov/entrez/query.fcgi?cmd=search&db=gene&term=Irak2) | interleukin-1 receptor-associated kinase 2 |
| 397 | 9,67E-05 | 0,00475 | 7,49 | 309,71 | 0,024 | [1452123_s_at](https://www.affymetrix.com/LinkServlet?probeset=1452123_s_at) | [Frmd4b](http://www.ncbi.nlm.nih.gov/entrez/query.fcgi?cmd=search&db=gene&term=Frmd4b) | FERM domain containing 4B |
| 398 | 9,67E-05 | 0,00475 | 58,28 | 20,36 | 2,86 | [1435131_at](https://www.affymetrix.com/LinkServlet?probeset=1435131_at) | [Zfp13](http://www.ncbi.nlm.nih.gov/entrez/query.fcgi?cmd=search&db=gene&term=Zfp13) | zinc finger protein 13 |
| 399 | 9,70E-05 | 0,00475 | 4,4 | 247,01 | 0,018 | [1434096_at](https://www.affymetrix.com/LinkServlet?probeset=1434096_at) | [Slc4a4](http://www.ncbi.nlm.nih.gov/entrez/query.fcgi?cmd=search&db=gene&term=Slc4a4) | solute carrier family 4 (anion exchanger), member 4 |
| 400 | 9,72E-05 | 0,00475 | 19852,65 | 5543,69 | 3,58 | [1422444_at](https://www.affymetrix.com/LinkServlet?probeset=1422444_at) | [Itga6](http://www.ncbi.nlm.nih.gov/entrez/query.fcgi?cmd=search&db=gene&term=Itga6) | integrin alpha 6 |
| 401 | 9,73E-05 | 0,00475 | 1098,89 | 4239,9 | 0,26 | [1455288_at](https://www.affymetrix.com/LinkServlet?probeset=1455288_at) | [Leprel4](http://www.ncbi.nlm.nih.gov/entrez/query.fcgi?cmd=search&db=gene&term=Leprel4) | leprecan-like 4 |
| 402 | 9,74E-05 | 0,00475 | 7357,13 | 2664,77 | 2,76 | [1450894_a_at](https://www.affymetrix.com/LinkServlet?probeset=1450894_a_at) | [Ap2m1](http://www.ncbi.nlm.nih.gov/entrez/query.fcgi?cmd=search&db=gene&term=Ap2m1) | adaptor protein complex AP-2, mu1 |
| 403 | 9,75E-05 | 0,00475 | 6,2 | 102,09 | 0,061 | [1427433_s_at](https://www.affymetrix.com/LinkServlet?probeset=1427433_s_at) | [Hoxa3](http://www.ncbi.nlm.nih.gov/entrez/query.fcgi?cmd=search&db=gene&term=Hoxa3) | homeobox A3 |
| 404 | 9,77E-05 | 0,00475 | 64,92 | 155,55 | 0,42 | [1441986_at](https://www.affymetrix.com/LinkServlet?probeset=1441986_at) | [Zcchc6](http://www.ncbi.nlm.nih.gov/entrez/query.fcgi?cmd=search&db=gene&term=Zcchc6) | zinc finger, CCHC domain containing 6 |
| 405 | 9,82E-05 | 0,00475 | 236,44 | 27,54 | 8,58 | [1435872_at](https://www.affymetrix.com/LinkServlet?probeset=1435872_at) | [NA](http://www.ncbi.nlm.nih.gov/entrez/query.fcgi?cmd=search&db=gene&term=NA) | NA |
| 406 | 9,82E-05 | 0,00475 | 621,22 | 1013,45 | 0,61 | [1448414_at](https://www.affymetrix.com/LinkServlet?probeset=1448414_at) | [Rad1](http://www.ncbi.nlm.nih.gov/entrez/query.fcgi?cmd=search&db=gene&term=Rad1) | RAD1 homolog (S. pombe) |
| 407 | 9,85E-05 | 0,00475 | 380,97 | 43,14 | 8,83 | [1424179_at](https://www.affymetrix.com/LinkServlet?probeset=1424179_at) | [Plekhj1](http://www.ncbi.nlm.nih.gov/entrez/query.fcgi?cmd=search&db=gene&term=Plekhj1) | pleckstrin homology domain containing, family J member 1 |
| 408 | 9,86E-05 | 0,00475 | 4,74 | 97,45 | 0,049 | [1456307_s_at](https://www.affymetrix.com/LinkServlet?probeset=1456307_s_at) | [Adcy7](http://www.ncbi.nlm.nih.gov/entrez/query.fcgi?cmd=search&db=gene&term=Adcy7) | adenylate cyclase 7 |
| 409 | 9,87E-05 | 0,00475 | 1283,7 | 247,46 | 5,19 | [1449151_at](https://www.affymetrix.com/LinkServlet?probeset=1449151_at) | [Cdk18](http://www.ncbi.nlm.nih.gov/entrez/query.fcgi?cmd=search&db=gene&term=Cdk18) | cyclin-dependent kinase 18 |
| 410 | 9,92E-05 | 0,00476 | 7445,22 | 46133 | 0,16 | [1421811_at](https://www.affymetrix.com/LinkServlet?probeset=1421811_at) | [Thbs1](http://www.ncbi.nlm.nih.gov/entrez/query.fcgi?cmd=search&db=gene&term=Thbs1) | thrombospondin 1 |
| 411 | 0,0001001 | 0,00479 | 1212,31 | 374,24 | 3,24 | [1426944_at](https://www.affymetrix.com/LinkServlet?probeset=1426944_at) | [Fbxw8](http://www.ncbi.nlm.nih.gov/entrez/query.fcgi?cmd=search&db=gene&term=Fbxw8) | F-box and WD-40 domain protein 8 |
| 412 | 0,0001005 | 0,0048 | 5398,63 | 716,82 | 7,53 | [1415819_a_at](https://www.affymetrix.com/LinkServlet?probeset=1415819_a_at) | [Ppp2r1a](http://www.ncbi.nlm.nih.gov/entrez/query.fcgi?cmd=search&db=gene&term=Ppp2r1a) | protein phosphatase 2 (formerly 2A), regulatory subunit A (PR 65), alpha isoform |
| 413 | 0,0001007 | 0,0048 | 247,1 | 78,73 | 3,14 | [1447929_at](https://www.affymetrix.com/LinkServlet?probeset=1447929_at) | [Ssh3](http://www.ncbi.nlm.nih.gov/entrez/query.fcgi?cmd=search&db=gene&term=Ssh3) | slingshot homolog 3 (Drosophila) |
| 414 | 0,0001031 | 0,0049 | 108,24 | 552,71 | 0,2 | [1456419_at](https://www.affymetrix.com/LinkServlet?probeset=1456419_at) | [5730455P16Rik](http://www.ncbi.nlm.nih.gov/entrez/query.fcgi?cmd=search&db=gene&term=5730455P16Rik) | RIKEN cDNA 5730455P16 gene |
| 415 | 0,0001034 | 0,0049 | 11,22 | 317,18 | 0,035 | [1430568_at](https://www.affymetrix.com/LinkServlet?probeset=1430568_at) | [Zc3h13](http://www.ncbi.nlm.nih.gov/entrez/query.fcgi?cmd=search&db=gene&term=Zc3h13) | zinc finger CCCH type containing 13 |
| 416 | 0,000104 | 0,00492 | 6501,79 | 3697,86 | 1,76 | [1422887_a_at](https://www.affymetrix.com/LinkServlet?probeset=1422887_a_at) | [Ctbp2](http://www.ncbi.nlm.nih.gov/entrez/query.fcgi?cmd=search&db=gene&term=Ctbp2) | C-terminal binding protein 2 |
| 417 | 0,0001045 | 0,00493 | 2038,18 | 3257,33 | 0,63 | [1437289_at](https://www.affymetrix.com/LinkServlet?probeset=1437289_at) | [Impad1](http://www.ncbi.nlm.nih.gov/entrez/query.fcgi?cmd=search&db=gene&term=Impad1) | inositol monophosphatase domain containing 1 |
| 418 | 0,0001048 | 0,00493 | 17,09 | 64,72 | 0,26 | [1447271_at](https://www.affymetrix.com/LinkServlet?probeset=1447271_at) | [Nck1](http://www.ncbi.nlm.nih.gov/entrez/query.fcgi?cmd=search&db=gene&term=Nck1) | non-catalytic region of tyrosine kinase adaptor protein 1 |
| 419 | 0,000106 | 0,00498 | 3936,08 | 6950,08 | 0,57 | [1435616_at](https://www.affymetrix.com/LinkServlet?probeset=1435616_at) | [Cyp20a1](http://www.ncbi.nlm.nih.gov/entrez/query.fcgi?cmd=search&db=gene&term=Cyp20a1) | cytochrome P450, family 20, subfamily A, polypeptide 1 |
| 420 | 0,0001065 | 0,00499 | 950,72 | 1830,55 | 0,52 | [1428652_at](https://www.affymetrix.com/LinkServlet?probeset=1428652_at) | [0610010F05Rik](http://www.ncbi.nlm.nih.gov/entrez/query.fcgi?cmd=search&db=gene&term=0610010F05Rik) | RIKEN cDNA 0610010F05 gene |
| 421 | 0,0001083 | 0,00505 | 4,84 | 73,84 | 0,066 | [1418604_at](https://www.affymetrix.com/LinkServlet?probeset=1418604_at) | [Avpr1a](http://www.ncbi.nlm.nih.gov/entrez/query.fcgi?cmd=search&db=gene&term=Avpr1a) | arginine vasopressin receptor 1A |
| 422 | 0,0001084 | 0,00505 | 532,37 | 94,89 | 5,61 | [1460724_at](https://www.affymetrix.com/LinkServlet?probeset=1460724_at) | [Ap2a1](http://www.ncbi.nlm.nih.gov/entrez/query.fcgi?cmd=search&db=gene&term=Ap2a1) | adaptor protein complex AP-2, alpha 1 subunit |
| 423 | 0,0001087 | 0,00505 | 14,36 | 94,51 | 0,15 | [1427209_at](https://www.affymetrix.com/LinkServlet?probeset=1427209_at) | [Baz2a](http://www.ncbi.nlm.nih.gov/entrez/query.fcgi?cmd=search&db=gene&term=Baz2a) | bromodomain adjacent to zinc finger domain, 2A |
| 424 | 0,0001088 | 0,00505 | 62,57 | 23,01 | 2,72 | [1444773_at](https://www.affymetrix.com/LinkServlet?probeset=1444773_at) | [D430040D24Rik](http://www.ncbi.nlm.nih.gov/entrez/query.fcgi?cmd=search&db=gene&term=D430040D24Rik) | RIKEN cDNA D430040D24 gene |
| 425 | 0,0001095 | 0,00505 | 4,48 | 344,04 | 0,013 | [1428331_at](https://www.affymetrix.com/LinkServlet?probeset=1428331_at) | [2210016F16Rik](http://www.ncbi.nlm.nih.gov/entrez/query.fcgi?cmd=search&db=gene&term=2210016F16Rik) | RIKEN cDNA 2210016F16 gene |
| 426 | 0,0001098 | 0,00505 | 4,4 | 24,15 | 0,18 | [1443022_at](https://www.affymetrix.com/LinkServlet?probeset=1443022_at) | [Tctn3](http://www.ncbi.nlm.nih.gov/entrez/query.fcgi?cmd=search&db=gene&term=Tctn3) | tectonic family member 3 |
| 427 | 0,0001099 | 0,00505 | 4149,34 | 1823,03 | 2,28 | [1423102_a_at](https://www.affymetrix.com/LinkServlet?probeset=1423102_a_at) | [Rnf10](http://www.ncbi.nlm.nih.gov/entrez/query.fcgi?cmd=search&db=gene&term=Rnf10) | ring finger protein 10 |
| 428 | 0,0001101 | 0,00505 | 64,68 | 379,01 | 0,17 | [1439827_at](https://www.affymetrix.com/LinkServlet?probeset=1439827_at) | [Adamts12](http://www.ncbi.nlm.nih.gov/entrez/query.fcgi?cmd=search&db=gene&term=Adamts12) | a disintegrin-like and metallopeptidase (reprolysin type) with thrombospondin type 1 motif, 12 |
| 429 | 0,0001102 | 0,00505 | 118,66 | 293,69 | 0,4 | [1436118_at](https://www.affymetrix.com/LinkServlet?probeset=1436118_at) | [Vangl2](http://www.ncbi.nlm.nih.gov/entrez/query.fcgi?cmd=search&db=gene&term=Vangl2) | vang-like 2 (van gogh, Drosophila) |
| 430 | 0,0001107 | 0,00506 | 15,8 | 64,33 | 0,25 | [1438301_at](https://www.affymetrix.com/LinkServlet?probeset=1438301_at) | [NA](http://www.ncbi.nlm.nih.gov/entrez/query.fcgi?cmd=search&db=gene&term=NA) | NA |
| 431 | 0,0001113 | 0,00506 | 1109,61 | 677,72 | 1,64 | [1426347_at](https://www.affymetrix.com/LinkServlet?probeset=1426347_at) | [2010321M09Rik](http://www.ncbi.nlm.nih.gov/entrez/query.fcgi?cmd=search&db=gene&term=2010321M09Rik) | RIKEN cDNA 2010321M09 gene |
| 432 | 0,0001113 | 0,00506 | 498,04 | 173,35 | 2,87 | [1433448_at](https://www.affymetrix.com/LinkServlet?probeset=1433448_at) | [Slc25a44](http://www.ncbi.nlm.nih.gov/entrez/query.fcgi?cmd=search&db=gene&term=Slc25a44) | solute carrier family 25, member 44 |
| 433 | 0,0001114 | 0,00506 | 31,84 | 57,12 | 0,56 | [1459975_at](https://www.affymetrix.com/LinkServlet?probeset=1459975_at) | [Myst2](http://www.ncbi.nlm.nih.gov/entrez/query.fcgi?cmd=search&db=gene&term=Myst2) | MYST histone acetyltransferase 2 |
| 434 | 0,0001126 | 0,00507 | 225,79 | 856,13 | 0,26 | [1455057_at](https://www.affymetrix.com/LinkServlet?probeset=1455057_at) | [Gmps](http://www.ncbi.nlm.nih.gov/entrez/query.fcgi?cmd=search&db=gene&term=Gmps) | guanine monophosphate synthetase |
| 435 | 0,0001131 | 0,00507 | 4,4 | 6,3 | 0,7 | [1445882_at](https://www.affymetrix.com/LinkServlet?probeset=1445882_at) | [Cd300lb](http://www.ncbi.nlm.nih.gov/entrez/query.fcgi?cmd=search&db=gene&term=Cd300lb) | CD300 antigen like family member B |
| 436 | 0,0001133 | 0,00507 | 3397,15 | 6033,62 | 0,56 | [1416655_at](https://www.affymetrix.com/LinkServlet?probeset=1416655_at) | [C1galt1c1](http://www.ncbi.nlm.nih.gov/entrez/query.fcgi?cmd=search&db=gene&term=C1galt1c1) | C1GALT1-specific chaperone 1 |
| 437 | 0,0001134 | 0,00507 | 484,02 | 1678,34 | 0,29 | [1422906_at](https://www.affymetrix.com/LinkServlet?probeset=1422906_at) | [Abcg2](http://www.ncbi.nlm.nih.gov/entrez/query.fcgi?cmd=search&db=gene&term=Abcg2) | ATP-binding cassette, sub-family G (WHITE), member 2 |
| 438 | 0,0001136 | 0,00507 | 63,28 | 358,84 | 0,18 | [1433915_s_at](https://www.affymetrix.com/LinkServlet?probeset=1433915_s_at) | [Epn2](http://www.ncbi.nlm.nih.gov/entrez/query.fcgi?cmd=search&db=gene&term=Epn2) | epsin 2 |
| 439 | 0,0001137 | 0,00507 | 7,27 | 241,93 | 0,03 | [1441845_at](https://www.affymetrix.com/LinkServlet?probeset=1441845_at) | [Caps2](http://www.ncbi.nlm.nih.gov/entrez/query.fcgi?cmd=search&db=gene&term=Caps2) | calcyphosphine 2 |
| 440 | 0,0001139 | 0,00507 | 14502,04 | 5415,69 | 2,68 | [1455827_at](https://www.affymetrix.com/LinkServlet?probeset=1455827_at) | [Mbnl2](http://www.ncbi.nlm.nih.gov/entrez/query.fcgi?cmd=search&db=gene&term=Mbnl2) | muscleblind-like 2 |
| 441 | 0,0001139 | 0,00507 | 2261,83 | 3444,64 | 0,66 | [1449668_s_at](https://www.affymetrix.com/LinkServlet?probeset=1449668_s_at) | [Fnip1](http://www.ncbi.nlm.nih.gov/entrez/query.fcgi?cmd=search&db=gene&term=Fnip1) | folliculin interacting protein 1 |
| 442 | 0,000114 | 0,00507 | 1041,51 | 4145,91 | 0,25 | [1460639_a_at](https://www.affymetrix.com/LinkServlet?probeset=1460639_a_at) | [Atox1](http://www.ncbi.nlm.nih.gov/entrez/query.fcgi?cmd=search&db=gene&term=Atox1) | ATX1 (antioxidant protein 1) homolog 1 (yeast) |
| 443 | 0,0001148 | 0,00508 | 140,42 | 15,43 | 9,1 | [1420833_at](https://www.affymetrix.com/LinkServlet?probeset=1420833_at) | [Vamp2](http://www.ncbi.nlm.nih.gov/entrez/query.fcgi?cmd=search&db=gene&term=Vamp2) | vesicle-associated membrane protein 2 |
| 444 | 0,0001149 | 0,00508 | 826,12 | 239,5 | 3,45 | [1434290_at](https://www.affymetrix.com/LinkServlet?probeset=1434290_at) | [Gtdc1](http://www.ncbi.nlm.nih.gov/entrez/query.fcgi?cmd=search&db=gene&term=Gtdc1) | glycosyltransferase-like domain containing 1 |
| 445 | 0,0001149 | 0,00508 | 510,25 | 48,89 | 10,44 | [1417476_at](https://www.affymetrix.com/LinkServlet?probeset=1417476_at) | [Fbxw5](http://www.ncbi.nlm.nih.gov/entrez/query.fcgi?cmd=search&db=gene&term=Fbxw5) | F-box and WD-40 domain protein 5 |
| 446 | 0,0001151 | 0,00508 | 119,71 | 9,79 | 12,23 | [1447625_at](https://www.affymetrix.com/LinkServlet?probeset=1447625_at) | [E2f5](http://www.ncbi.nlm.nih.gov/entrez/query.fcgi?cmd=search&db=gene&term=E2f5) | E2F transcription factor 5 |
| 447 | 0,0001157 | 0,00509 | 265,24 | 1568,54 | 0,17 | [1444524_at](https://www.affymetrix.com/LinkServlet?probeset=1444524_at) | [Gm14005](http://www.ncbi.nlm.nih.gov/entrez/query.fcgi?cmd=search&db=gene&term=Gm14005) | predicted gene 14005 |
| 448 | 0,0001159 | 0,00509 | 1695,11 | 3805,9 | 0,45 | [1422470_at](https://www.affymetrix.com/LinkServlet?probeset=1422470_at) | [Bnip3](http://www.ncbi.nlm.nih.gov/entrez/query.fcgi?cmd=search&db=gene&term=Bnip3) | BCL2/adenovirus E1B interacting protein 3 |
| 449 | 0,0001169 | 0,00509 | 10669,49 | 20188,68 | 0,53 | [1451071_a_at](https://www.affymetrix.com/LinkServlet?probeset=1451071_a_at) | [Atp1a1](http://www.ncbi.nlm.nih.gov/entrez/query.fcgi?cmd=search&db=gene&term=Atp1a1) | ATPase, Na+/K+ transporting, alpha 1 polypeptide |
| 450 | 0,0001172 | 0,00509 | 7,8 | 111,88 | 0,07 | [1442427_at](https://www.affymetrix.com/LinkServlet?probeset=1442427_at) | [NA](http://www.ncbi.nlm.nih.gov/entrez/query.fcgi?cmd=search&db=gene&term=NA) | NA |
| 451 | 0,0001179 | 0,00509 | 61,12 | 5,97 | 10,23 | [1422341_s_at](https://www.affymetrix.com/LinkServlet?probeset=1422341_s_at) | [Pla2g15](http://www.ncbi.nlm.nih.gov/entrez/query.fcgi?cmd=search&db=gene&term=Pla2g15) | phospholipase A2, group XV |
| 452 | 0,000118 | 0,00509 | 92,07 | 324,4 | 0,28 | [1428828_at](https://www.affymetrix.com/LinkServlet?probeset=1428828_at) | [Rpain](http://www.ncbi.nlm.nih.gov/entrez/query.fcgi?cmd=search&db=gene&term=Rpain) | RPA interacting protein |
| 453 | 0,0001184 | 0,00509 | 5659,19 | 2924,99 | 1,93 | [1421870_at](https://www.affymetrix.com/LinkServlet?probeset=1421870_at) | [Trim44](http://www.ncbi.nlm.nih.gov/entrez/query.fcgi?cmd=search&db=gene&term=Trim44) | tripartite motif-containing 44 |
| 454 | 0,0001187 | 0,00509 | 1596,19 | 3396,41 | 0,47 | [1428193_at](https://www.affymetrix.com/LinkServlet?probeset=1428193_at) | [Usp9x](http://www.ncbi.nlm.nih.gov/entrez/query.fcgi?cmd=search&db=gene&term=Usp9x) | ubiquitin specific peptidase 9, X chromosome |
| 455 | 0,0001187 | 0,00509 | 8,76 | 157,14 | 0,056 | [1437864_at](https://www.affymetrix.com/LinkServlet?probeset=1437864_at) | [Adipor2](http://www.ncbi.nlm.nih.gov/entrez/query.fcgi?cmd=search&db=gene&term=Adipor2) | adiponectin receptor 2 |
| 456 | 0,0001188 | 0,00509 | 805,84 | 258,89 | 3,11 | [1460703_at](https://www.affymetrix.com/LinkServlet?probeset=1460703_at) | [Ascc1](http://www.ncbi.nlm.nih.gov/entrez/query.fcgi?cmd=search&db=gene&term=Ascc1) | activating signal cointegrator 1 complex subunit 1 |
| 457 | 0,0001192 | 0,00509 | 92,92 | 5,83 | 15,94 | [1449554_at](https://www.affymetrix.com/LinkServlet?probeset=1449554_at) | [Tle3](http://www.ncbi.nlm.nih.gov/entrez/query.fcgi?cmd=search&db=gene&term=Tle3) | transducin-like enhancer of split 3, homolog of Drosophila E(spl) |
| 458 | 0,0001193 | 0,00509 | 3322,43 | 1006,23 | 3,3 | [1437155_a_at](https://www.affymetrix.com/LinkServlet?probeset=1437155_a_at) | [Wwtr1](http://www.ncbi.nlm.nih.gov/entrez/query.fcgi?cmd=search&db=gene&term=Wwtr1) | WW domain containing transcription regulator 1 |
| 459 | 0,0001198 | 0,00509 | 2226,15 | 862,5 | 2,58 | [1454645_at](https://www.affymetrix.com/LinkServlet?probeset=1454645_at) | [Mgrn1](http://www.ncbi.nlm.nih.gov/entrez/query.fcgi?cmd=search&db=gene&term=Mgrn1) | mahogunin, ring finger 1 |
| 460 | 0,0001198 | 0,00509 | 898,11 | 1911,66 | 0,47 | [1418458_at](https://www.affymetrix.com/LinkServlet?probeset=1418458_at) | [Anapc7](http://www.ncbi.nlm.nih.gov/entrez/query.fcgi?cmd=search&db=gene&term=Anapc7) | anaphase promoting complex subunit 7 |
| 461 | 0,0001202 | 0,00509 | 189,9 | 59,25 | 3,21 | [1452139_at](https://www.affymetrix.com/LinkServlet?probeset=1452139_at) | [Slc35c1](http://www.ncbi.nlm.nih.gov/entrez/query.fcgi?cmd=search&db=gene&term=Slc35c1) | solute carrier family 35, member C1 |
| 462 | 0,0001205 | 0,00509 | 6,02 | 37,28 | 0,16 | [1437302_at](https://www.affymetrix.com/LinkServlet?probeset=1437302_at) | [Adrb2](http://www.ncbi.nlm.nih.gov/entrez/query.fcgi?cmd=search&db=gene&term=Adrb2) | adrenergic receptor, beta 2 |
| 463 | 0,0001207 | 0,00509 | 538,36 | 9,13 | 58,97 | [1416007_at](https://www.affymetrix.com/LinkServlet?probeset=1416007_at) | [Satb1](http://www.ncbi.nlm.nih.gov/entrez/query.fcgi?cmd=search&db=gene&term=Satb1) | special AT-rich sequence binding protein 1 |
| 464 | 0,0001209 | 0,00509 | 277,38 | 28,4 | 9,77 | [1450151_at](https://www.affymetrix.com/LinkServlet?probeset=1450151_at) | [Zfp316](http://www.ncbi.nlm.nih.gov/entrez/query.fcgi?cmd=search&db=gene&term=Zfp316) | zinc finger protein 316 |
| 465 | 0,000121 | 0,00509 | 68,84 | 209,08 | 0,33 | [1456339_at](https://www.affymetrix.com/LinkServlet?probeset=1456339_at) | [2810410D24Rik](http://www.ncbi.nlm.nih.gov/entrez/query.fcgi?cmd=search&db=gene&term=2810410D24Rik) | RIKEN cDNA 2810410D24 gene |
| 466 | 0,0001214 | 0,00509 | 20,99 | 200,41 | 0,1 | [1425749_at](https://www.affymetrix.com/LinkServlet?probeset=1425749_at) | [Stxbp6](http://www.ncbi.nlm.nih.gov/entrez/query.fcgi?cmd=search&db=gene&term=Stxbp6) | syntaxin binding protein 6 (amisyn) |
| 467 | 0,0001217 | 0,00509 | 3605,09 | 1368,68 | 2,63 | [1421754_at](https://www.affymetrix.com/LinkServlet?probeset=1421754_at) | [AY036118](http://www.ncbi.nlm.nih.gov/entrez/query.fcgi?cmd=search&db=gene&term=AY036118) | cDNA sequence AY036118 |
| 468 | 0,0001219 | 0,00509 | 3645,72 | 6438,16 | 0,57 | [1454724_x_at](https://www.affymetrix.com/LinkServlet?probeset=1454724_x_at) | [Fam108b](http://www.ncbi.nlm.nih.gov/entrez/query.fcgi?cmd=search&db=gene&term=Fam108b) | family with sequence similarity 108, member B |
| 469 | 0,0001221 | 0,00509 | 335,73 | 82,39 | 4,07 | [1426828_at](https://www.affymetrix.com/LinkServlet?probeset=1426828_at) | [1300018I17Rik](http://www.ncbi.nlm.nih.gov/entrez/query.fcgi?cmd=search&db=gene&term=1300018I17Rik) | RIKEN cDNA 1300018I17 gene |
| 470 | 0,0001222 | 0,00509 | 6450,82 | 14068,15 | 0,46 | [1417660_s_at](https://www.affymetrix.com/LinkServlet?probeset=1417660_s_at) | [Vps29](http://www.ncbi.nlm.nih.gov/entrez/query.fcgi?cmd=search&db=gene&term=Vps29) | vacuolar protein sorting 29 (S. pombe) |
| 471 | 0,0001224 | 0,00509 | 73,51 | 11,27 | 6,52 | [1447566_at](https://www.affymetrix.com/LinkServlet?probeset=1447566_at) | [Hdac4](http://www.ncbi.nlm.nih.gov/entrez/query.fcgi?cmd=search&db=gene&term=Hdac4) | histone deacetylase 4 |
| 472 | 0,0001224 | 0,00509 | 901,51 | 352,33 | 2,56 | [1416358_at](https://www.affymetrix.com/LinkServlet?probeset=1416358_at) | [Mfsd10](http://www.ncbi.nlm.nih.gov/entrez/query.fcgi?cmd=search&db=gene&term=Mfsd10) | major facilitator superfamily domain containing 10 |
| 473 | 0,0001229 | 0,00509 | 911,91 | 1850,12 | 0,49 | [1429461_at](https://www.affymetrix.com/LinkServlet?probeset=1429461_at) | [Ints2](http://www.ncbi.nlm.nih.gov/entrez/query.fcgi?cmd=search&db=gene&term=Ints2) | integrator complex subunit 2 |
| 474 | 0,000123 | 0,00509 | 3913 | 1480,26 | 2,64 | [1422550_a_at](https://www.affymetrix.com/LinkServlet?probeset=1422550_a_at) | [Mtap6](http://www.ncbi.nlm.nih.gov/entrez/query.fcgi?cmd=search&db=gene&term=Mtap6) | microtubule-associated protein 6 |
| 475 | 0,0001232 | 0,00509 | 4,42 | 39,22 | 0,11 | [1447227_at](https://www.affymetrix.com/LinkServlet?probeset=1447227_at) | [NA](http://www.ncbi.nlm.nih.gov/entrez/query.fcgi?cmd=search&db=gene&term=NA) | NA |
| 476 | 0,0001233 | 0,00509 | 693,91 | 35,77 | 19,4 | [1460587_at](https://www.affymetrix.com/LinkServlet?probeset=1460587_at) | [Sox2ot](http://www.ncbi.nlm.nih.gov/entrez/query.fcgi?cmd=search&db=gene&term=Sox2ot) | SOX2 overlapping transcript (non-protein coding) |
| 477 | 0,000124 | 0,0051 | 282,13 | 9,8 | 28,79 | [1423537_at](https://www.affymetrix.com/LinkServlet?probeset=1423537_at) | [Gap43](http://www.ncbi.nlm.nih.gov/entrez/query.fcgi?cmd=search&db=gene&term=Gap43) | growth associated protein 43 |
| 478 | 0,000124 | 0,0051 | 199,19 | 43,26 | 4,6 | [1433499_at](https://www.affymetrix.com/LinkServlet?probeset=1433499_at) | [Urgcp](http://www.ncbi.nlm.nih.gov/entrez/query.fcgi?cmd=search&db=gene&term=Urgcp) | upregulator of cell proliferation |
| 479 | 0,0001245 | 0,00511 | 6,81 | 94,55 | 0,072 | [1445617_at](https://www.affymetrix.com/LinkServlet?probeset=1445617_at) | [NA](http://www.ncbi.nlm.nih.gov/entrez/query.fcgi?cmd=search&db=gene&term=NA) | NA |
| 480 | 0,0001247 | 0,00511 | 806,33 | 194,21 | 4,15 | [1419415_a_at](https://www.affymetrix.com/LinkServlet?probeset=1419415_a_at) | [Rarg](http://www.ncbi.nlm.nih.gov/entrez/query.fcgi?cmd=search&db=gene&term=Rarg) | retinoic acid receptor, gamma |
| 481 | 0,000125 | 0,00511 | 210,89 | 9,26 | 22,77 | [1456532_at](https://www.affymetrix.com/LinkServlet?probeset=1456532_at) | [Pdgfd](http://www.ncbi.nlm.nih.gov/entrez/query.fcgi?cmd=search&db=gene&term=Pdgfd) | platelet-derived growth factor, D polypeptide |
| 482 | 0,0001258 | 0,00511 | 1054,57 | 45,46 | 23,2 | [AFFX-BioB-M_at](https://www.affymetrix.com/LinkServlet?probeset=AFFX-BioB-M_at) | [NA](http://www.ncbi.nlm.nih.gov/entrez/query.fcgi?cmd=search&db=gene&term=NA) | NA |
| 483 | 0,0001259 | 0,00511 | 34,74 | 112,32 | 0,31 | [1438740_at](https://www.affymetrix.com/LinkServlet?probeset=1438740_at) | [Nmt2](http://www.ncbi.nlm.nih.gov/entrez/query.fcgi?cmd=search&db=gene&term=Nmt2) | N-myristoyltransferase 2 |
| 484 | 0,0001261 | 0,00511 | 896,24 | 428,43 | 2,09 | [1460189_at](https://www.affymetrix.com/LinkServlet?probeset=1460189_at) | [Dcaf11](http://www.ncbi.nlm.nih.gov/entrez/query.fcgi?cmd=search&db=gene&term=Dcaf11) | DDB1 and CUL4 associated factor 11 |
| 485 | 0,0001261 | 0,00511 | 113,14 | 62,3 | 1,82 | [1451522_s_at](https://www.affymetrix.com/LinkServlet?probeset=1451522_s_at) | [NA](http://www.ncbi.nlm.nih.gov/entrez/query.fcgi?cmd=search&db=gene&term=NA) | NA |
| 486 | 0,0001267 | 0,00513 | 793,39 | 213,26 | 3,72 | [1453865_a_at](https://www.affymetrix.com/LinkServlet?probeset=1453865_a_at) | [Otud5](http://www.ncbi.nlm.nih.gov/entrez/query.fcgi?cmd=search&db=gene&term=Otud5) | OTU domain containing 5 |
| 487 | 0,0001282 | 0,00518 | 10652,81 | 1846,14 | 5,77 | [1429088_at](https://www.affymetrix.com/LinkServlet?probeset=1429088_at) | [Lbh](http://www.ncbi.nlm.nih.gov/entrez/query.fcgi?cmd=search&db=gene&term=Lbh) | limb-bud and heart |
| 488 | 0,0001295 | 0,00522 | 68,84 | 1173,75 | 0,059 | [1453855_at](https://www.affymetrix.com/LinkServlet?probeset=1453855_at) | [Mxra7](http://www.ncbi.nlm.nih.gov/entrez/query.fcgi?cmd=search&db=gene&term=Mxra7) | matrix-remodelling associated 7 |
| 489 | 0,00013 | 0,00523 | 1664,75 | 856,7 | 1,94 | [1428395_at](https://www.affymetrix.com/LinkServlet?probeset=1428395_at) | [Smurf1](http://www.ncbi.nlm.nih.gov/entrez/query.fcgi?cmd=search&db=gene&term=Smurf1) | SMAD specific E3 ubiquitin protein ligase 1 |
| 490 | 0,0001304 | 0,00523 | 8,25 | 32,53 | 0,25 | [1447853_x_at](https://www.affymetrix.com/LinkServlet?probeset=1447853_x_at) | [Kif13a](http://www.ncbi.nlm.nih.gov/entrez/query.fcgi?cmd=search&db=gene&term=Kif13a) | kinesin family member 13A |
| 491 | 0,0001306 | 0,00523 | 12,76 | 204,64 | 0,062 | [1426981_at](https://www.affymetrix.com/LinkServlet?probeset=1426981_at) | [Pcsk6](http://www.ncbi.nlm.nih.gov/entrez/query.fcgi?cmd=search&db=gene&term=Pcsk6) | proprotein convertase subtilisin/kexin type 6 |
| 492 | 0,000131 | 0,00524 | 86,34 | 378,73 | 0,23 | [1452132_at](https://www.affymetrix.com/LinkServlet?probeset=1452132_at) | [Tlcd1](http://www.ncbi.nlm.nih.gov/entrez/query.fcgi?cmd=search&db=gene&term=Tlcd1) | TLC domain containing 1 |
| 493 | 0,0001318 | 0,00526 | 96,31 | 4,68 | 20,59 | [1449887_at](https://www.affymetrix.com/LinkServlet?probeset=1449887_at) | [Chmp4c](http://www.ncbi.nlm.nih.gov/entrez/query.fcgi?cmd=search&db=gene&term=Chmp4c) | chromatin modifying protein 4C |
| 494 | 0,0001323 | 0,00526 | 28,85 | 285,12 | 0,1 | [1449999_a_at](https://www.affymetrix.com/LinkServlet?probeset=1449999_a_at) | [Cacna2d1](http://www.ncbi.nlm.nih.gov/entrez/query.fcgi?cmd=search&db=gene&term=Cacna2d1) | calcium channel, voltage-dependent, alpha2/delta subunit 1 |
| 495 | 0,0001326 | 0,00526 | 1124,41 | 23,51 | 47,82 | [1429148_at](https://www.affymetrix.com/LinkServlet?probeset=1429148_at) | [Nfic](http://www.ncbi.nlm.nih.gov/entrez/query.fcgi?cmd=search&db=gene&term=Nfic) | nuclear factor I/C |
| 496 | 0,0001327 | 0,00526 | 17,71 | 310,14 | 0,057 | [1448352_at](https://www.affymetrix.com/LinkServlet?probeset=1448352_at) | [Luzp1](http://www.ncbi.nlm.nih.gov/entrez/query.fcgi?cmd=search&db=gene&term=Luzp1) | leucine zipper protein 1 |
| 497 | 0,0001332 | 0,00527 | 13,94 | 10,47 | 1,33 | [1453338_at](https://www.affymetrix.com/LinkServlet?probeset=1453338_at) | [Stam2](http://www.ncbi.nlm.nih.gov/entrez/query.fcgi?cmd=search&db=gene&term=Stam2) | signal transducing adaptor molecule (SH3 domain and ITAM motif) 2 |
| 498 | 0,0001339 | 0,00528 | 742,26 | 116,64 | 6,36 | [1423967_at](https://www.affymetrix.com/LinkServlet?probeset=1423967_at) | [Palm](http://www.ncbi.nlm.nih.gov/entrez/query.fcgi?cmd=search&db=gene&term=Palm) | paralemmin |
| 499 | 0,0001339 | 0,00528 | 408,69 | 34,75 | 11,76 | [1427695_a_at](https://www.affymetrix.com/LinkServlet?probeset=1427695_a_at) | [Pou2f1](http://www.ncbi.nlm.nih.gov/entrez/query.fcgi?cmd=search&db=gene&term=Pou2f1) | POU domain, class 2, transcription factor 1 |
| 500 | 0,0001344 | 0,00528 | 620,86 | 2490,82 | 0,25 | [1417252_at](https://www.affymetrix.com/LinkServlet?probeset=1417252_at) | [Nt5c](http://www.ncbi.nlm.nih.gov/entrez/query.fcgi?cmd=search&db=gene&term=Nt5c) | 5',3'-nucleotidase, cytosolic |
| 501 | 0,0001345 | 0,00528 | 208,11 | 546,53 | 0,38 | [1439515_at](https://www.affymetrix.com/LinkServlet?probeset=1439515_at) | [Setd5](http://www.ncbi.nlm.nih.gov/entrez/query.fcgi?cmd=search&db=gene&term=Setd5) | SET domain containing 5 |
| 502 | 0,0001347 | 0,00528 | 1176,71 | 358,53 | 3,28 | [1418890_a_at](https://www.affymetrix.com/LinkServlet?probeset=1418890_a_at) | [Rab3d](http://www.ncbi.nlm.nih.gov/entrez/query.fcgi?cmd=search&db=gene&term=Rab3d) | RAB3D, member RAS oncogene family |
| 503 | 0,0001352 | 0,00529 | 1181,38 | 680,63 | 1,74 | [1451267_at](https://www.affymetrix.com/LinkServlet?probeset=1451267_at) | [Sharpin](http://www.ncbi.nlm.nih.gov/entrez/query.fcgi?cmd=search&db=gene&term=Sharpin) | SHANK-associated RH domain interacting protein |
| 504 | 0,0001356 | 0,00529 | 16,62 | 719,06 | 0,023 | [1456489_at](https://www.affymetrix.com/LinkServlet?probeset=1456489_at) | [Pcf11](http://www.ncbi.nlm.nih.gov/entrez/query.fcgi?cmd=search&db=gene&term=Pcf11) | cleavage and polyadenylation factor subunit homolog (S. cerevisiae) |
| 505 | 0,0001362 | 0,0053 | 95,52 | 11,2 | 8,52 | [1417613_at](https://www.affymetrix.com/LinkServlet?probeset=1417613_at) | [Ier5](http://www.ncbi.nlm.nih.gov/entrez/query.fcgi?cmd=search&db=gene&term=Ier5) | immediate early response 5 |
| 506 | 0,0001369 | 0,00532 | 540,16 | 1456,88 | 0,37 | [1437152_at](https://www.affymetrix.com/LinkServlet?probeset=1437152_at) | [Mex3b](http://www.ncbi.nlm.nih.gov/entrez/query.fcgi?cmd=search&db=gene&term=Mex3b) | mex3 homolog B (C. elegans) |
| 507 | 0,0001373 | 0,00533 | 49,19 | 457,79 | 0,11 | [1426597_s_at](https://www.affymetrix.com/LinkServlet?probeset=1426597_s_at) | [Iffo2](http://www.ncbi.nlm.nih.gov/entrez/query.fcgi?cmd=search&db=gene&term=Iffo2) | intermediate filament family orphan 2 |
| 508 | 0,0001379 | 0,00534 | 4,4 | 37,55 | 0,12 | [1453807_at](https://www.affymetrix.com/LinkServlet?probeset=1453807_at) | [6330563C09Rik](http://www.ncbi.nlm.nih.gov/entrez/query.fcgi?cmd=search&db=gene&term=6330563C09Rik) | RIKEN cDNA 6330563C09 gene |
| 509 | 0,0001387 | 0,00534 | 20,29 | 42,35 | 0,48 | [1447312_at](https://www.affymetrix.com/LinkServlet?probeset=1447312_at) | [NA](http://www.ncbi.nlm.nih.gov/entrez/query.fcgi?cmd=search&db=gene&term=NA) | NA |
| 510 | 0,0001388 | 0,00534 | 607,99 | 2342,59 | 0,26 | [1420021_s_at](https://www.affymetrix.com/LinkServlet?probeset=1420021_s_at) | [Suz12](http://www.ncbi.nlm.nih.gov/entrez/query.fcgi?cmd=search&db=gene&term=Suz12) | suppressor of zeste 12 homolog (Drosophila) |
| 511 | 0,000139 | 0,00534 | 296,54 | 2177,22 | 0,14 | [1417623_at](https://www.affymetrix.com/LinkServlet?probeset=1417623_at) | [Slc12a2](http://www.ncbi.nlm.nih.gov/entrez/query.fcgi?cmd=search&db=gene&term=Slc12a2) | solute carrier family 12, member 2 |
| 512 | 0,0001392 | 0,00534 | 177,57 | 1200,49 | 0,15 | [1428794_at](https://www.affymetrix.com/LinkServlet?probeset=1428794_at) | [Specc1](http://www.ncbi.nlm.nih.gov/entrez/query.fcgi?cmd=search&db=gene&term=Specc1) | sperm antigen with calponin homology and coiled-coil domains 1 |
| 513 | 0,0001394 | 0,00534 | 4848 | 10688,56 | 0,45 | [1449893_a_at](https://www.affymetrix.com/LinkServlet?probeset=1449893_a_at) | [Lrig1](http://www.ncbi.nlm.nih.gov/entrez/query.fcgi?cmd=search&db=gene&term=Lrig1) | leucine-rich repeats and immunoglobulin-like domains 1 |
| 514 | 0,0001395 | 0,00534 | 209,12 | 20,99 | 9,96 | [1449029_at](https://www.affymetrix.com/LinkServlet?probeset=1449029_at) | [Mknk2](http://www.ncbi.nlm.nih.gov/entrez/query.fcgi?cmd=search&db=gene&term=Mknk2) | MAP kinase-interacting serine/threonine kinase 2 |
| 515 | 0,0001404 | 0,00536 | 637,2 | 196,16 | 3,25 | [1426811_at](https://www.affymetrix.com/LinkServlet?probeset=1426811_at) | [Ppp2r5b](http://www.ncbi.nlm.nih.gov/entrez/query.fcgi?cmd=search&db=gene&term=Ppp2r5b) | protein phosphatase 2, regulatory subunit B (B56), beta isoform |
| 516 | 0,0001414 | 0,00539 | 5,45 | 82,65 | 0,066 | [1417215_at](https://www.affymetrix.com/LinkServlet?probeset=1417215_at) | [Rab27b](http://www.ncbi.nlm.nih.gov/entrez/query.fcgi?cmd=search&db=gene&term=Rab27b) | RAB27b, member RAS oncogene family |
| 517 | 0,0001419 | 0,0054 | 35483,74 | 62140,3 | 0,57 | [1421375_a_at](https://www.affymetrix.com/LinkServlet?probeset=1421375_a_at) | [S100a6](http://www.ncbi.nlm.nih.gov/entrez/query.fcgi?cmd=search&db=gene&term=S100a6) | S100 calcium binding protein A6 (calcyclin) |
| 518 | 0,000143 | 0,00543 | 140,61 | 872,38 | 0,16 | [1438258_at](https://www.affymetrix.com/LinkServlet?probeset=1438258_at) | [Vldlr](http://www.ncbi.nlm.nih.gov/entrez/query.fcgi?cmd=search&db=gene&term=Vldlr) | very low density lipoprotein receptor |
| 519 | 0,0001441 | 0,00546 | 4,65 | 11,23 | 0,41 | [1436552_at](https://www.affymetrix.com/LinkServlet?probeset=1436552_at) | [Jakmip2](http://www.ncbi.nlm.nih.gov/entrez/query.fcgi?cmd=search&db=gene&term=Jakmip2) | janus kinase and microtubule interacting protein 2 |
| 520 | 0,0001443 | 0,00546 | 5,09 | 402,31 | 0,013 | [1418203_at](https://www.affymetrix.com/LinkServlet?probeset=1418203_at) | [Pmaip1](http://www.ncbi.nlm.nih.gov/entrez/query.fcgi?cmd=search&db=gene&term=Pmaip1) | phorbol-12-myristate-13-acetate-induced protein 1 |
| 521 | 0,0001447 | 0,00546 | 16,32 | 165,26 | 0,099 | [1457027_at](https://www.affymetrix.com/LinkServlet?probeset=1457027_at) | [Dhtkd1](http://www.ncbi.nlm.nih.gov/entrez/query.fcgi?cmd=search&db=gene&term=Dhtkd1) | dehydrogenase E1 and transketolase domain containing 1 |
| 522 | 0,0001454 | 0,00548 | 974,56 | 497 | 1,96 | [1426656_at](https://www.affymetrix.com/LinkServlet?probeset=1426656_at) | [Fam63a](http://www.ncbi.nlm.nih.gov/entrez/query.fcgi?cmd=search&db=gene&term=Fam63a) | family with sequence similarity 63, member A |
| 523 | 0,0001463 | 0,00548 | 476,04 | 103,69 | 4,59 | [1436910_at](https://www.affymetrix.com/LinkServlet?probeset=1436910_at) | [Rasal2](http://www.ncbi.nlm.nih.gov/entrez/query.fcgi?cmd=search&db=gene&term=Rasal2) | RAS protein activator like 2 |
| 524 | 0,0001465 | 0,00548 | 19,81 | 235,31 | 0,084 | [1437794_at](https://www.affymetrix.com/LinkServlet?probeset=1437794_at) | [Cabin1](http://www.ncbi.nlm.nih.gov/entrez/query.fcgi?cmd=search&db=gene&term=Cabin1) | calcineurin binding protein 1 |
| 525 | 0,0001465 | 0,00548 | 4,4 | 49,23 | 0,089 | [1417336_a_at](https://www.affymetrix.com/LinkServlet?probeset=1417336_a_at) | [Sytl4](http://www.ncbi.nlm.nih.gov/entrez/query.fcgi?cmd=search&db=gene&term=Sytl4) | synaptotagmin-like 4 |
| 526 | 0,0001467 | 0,00548 | 288,33 | 1279,49 | 0,23 | [1425991_a_at](https://www.affymetrix.com/LinkServlet?probeset=1425991_a_at) | [Kank2](http://www.ncbi.nlm.nih.gov/entrez/query.fcgi?cmd=search&db=gene&term=Kank2) | KN motif and ankyrin repeat domains 2 |
| 527 | 0,0001468 | 0,00548 | 6934,88 | 1085,93 | 6,39 | [1423916_s_at](https://www.affymetrix.com/LinkServlet?probeset=1423916_s_at) | [Mlf2](http://www.ncbi.nlm.nih.gov/entrez/query.fcgi?cmd=search&db=gene&term=Mlf2) | myeloid leukemia factor 2 |
| 528 | 0,0001473 | 0,00549 | 532,72 | 70,56 | 7,55 | [1431829_a_at](https://www.affymetrix.com/LinkServlet?probeset=1431829_a_at) | [Rgl3](http://www.ncbi.nlm.nih.gov/entrez/query.fcgi?cmd=search&db=gene&term=Rgl3) | ral guanine nucleotide dissociation stimulator-like 3 |
| 529 | 0,0001488 | 0,00553 | 9,4 | 38,61 | 0,24 | [1440392_at](https://www.affymetrix.com/LinkServlet?probeset=1440392_at) | [Akap13](http://www.ncbi.nlm.nih.gov/entrez/query.fcgi?cmd=search&db=gene&term=Akap13) | A kinase (PRKA) anchor protein 13 |
| 530 | 0,000149 | 0,00553 | 197,75 | 26,44 | 7,48 | [1418632_at](https://www.affymetrix.com/LinkServlet?probeset=1418632_at) | [Ube2h](http://www.ncbi.nlm.nih.gov/entrez/query.fcgi?cmd=search&db=gene&term=Ube2h) | ubiquitin-conjugating enzyme E2H |
| 531 | 0,0001499 | 0,00554 | 56,05 | 765,84 | 0,073 | [1420935_a_at](https://www.affymetrix.com/LinkServlet?probeset=1420935_a_at) | [Srrm1](http://www.ncbi.nlm.nih.gov/entrez/query.fcgi?cmd=search&db=gene&term=Srrm1) | serine/arginine repetitive matrix 1 |
| 532 | 0,0001499 | 0,00554 | 6,58 | 128,4 | 0,051 | [1452470_at](https://www.affymetrix.com/LinkServlet?probeset=1452470_at) | [Cep350](http://www.ncbi.nlm.nih.gov/entrez/query.fcgi?cmd=search&db=gene&term=Cep350) | centrosomal protein 350 |
| 533 | 0,0001508 | 0,00556 | 5,4 | 78,62 | 0,069 | [1426276_at](https://www.affymetrix.com/LinkServlet?probeset=1426276_at) | [Ifih1](http://www.ncbi.nlm.nih.gov/entrez/query.fcgi?cmd=search&db=gene&term=Ifih1) | interferon induced with helicase C domain 1 |
| 534 | 0,0001512 | 0,00557 | 71,2 | 1662,52 | 0,043 | [1420947_at](https://www.affymetrix.com/LinkServlet?probeset=1420947_at) | [Atrx](http://www.ncbi.nlm.nih.gov/entrez/query.fcgi?cmd=search&db=gene&term=Atrx) | alpha thalassemia/mental retardation syndrome X-linked homolog (human) |
| 535 | 0,0001528 | 0,00561 | 2650,73 | 9082,04 | 0,29 | [1420827_a_at](https://www.affymetrix.com/LinkServlet?probeset=1420827_a_at) | [Ccng1](http://www.ncbi.nlm.nih.gov/entrez/query.fcgi?cmd=search&db=gene&term=Ccng1) | cyclin G1 |
| 536 | 0,000153 | 0,00561 | 9,34 | 116,59 | 0,08 | [1417903_at](https://www.affymetrix.com/LinkServlet?probeset=1417903_at) | [Dfna5](http://www.ncbi.nlm.nih.gov/entrez/query.fcgi?cmd=search&db=gene&term=Dfna5) | deafness, autosomal dominant 5 (human) |
| 537 | 0,0001539 | 0,00564 | 1045,85 | 204,49 | 5,11 | [1423101_at](https://www.affymetrix.com/LinkServlet?probeset=1423101_at) | [Paqr4](http://www.ncbi.nlm.nih.gov/entrez/query.fcgi?cmd=search&db=gene&term=Paqr4) | progestin and adipoQ receptor family member IV |
| 538 | 0,000156 | 0,00568 | 310,71 | 45,91 | 6,77 | [1425940_a_at](https://www.affymetrix.com/LinkServlet?probeset=1425940_a_at) | [Ssbp3](http://www.ncbi.nlm.nih.gov/entrez/query.fcgi?cmd=search&db=gene&term=Ssbp3) | single-stranded DNA binding protein 3 |
| 539 | 0,0001562 | 0,00568 | 6,15 | 209,08 | 0,029 | [1427488_a_at](https://www.affymetrix.com/LinkServlet?probeset=1427488_a_at) | [Birc6](http://www.ncbi.nlm.nih.gov/entrez/query.fcgi?cmd=search&db=gene&term=Birc6) | baculoviral IAP repeat-containing 6 |
| 540 | 0,0001563 | 0,00568 | 399,51 | 567,02 | 0,7 | [1454994_at](https://www.affymetrix.com/LinkServlet?probeset=1454994_at) | [Klhl20](http://www.ncbi.nlm.nih.gov/entrez/query.fcgi?cmd=search&db=gene&term=Klhl20) | kelch-like 20 (Drosophila) |
| 541 | 0,0001575 | 0,00568 | 4093,78 | 7931,95 | 0,52 | [1415760_s_at](https://www.affymetrix.com/LinkServlet?probeset=1415760_s_at) | [Atox1](http://www.ncbi.nlm.nih.gov/entrez/query.fcgi?cmd=search&db=gene&term=Atox1) | ATX1 (antioxidant protein 1) homolog 1 (yeast) |
| 542 | 0,0001578 | 0,00568 | 4421,9 | 1047,86 | 4,22 | [1417209_at](https://www.affymetrix.com/LinkServlet?probeset=1417209_at) | [Sertad2](http://www.ncbi.nlm.nih.gov/entrez/query.fcgi?cmd=search&db=gene&term=Sertad2) | SERTA domain containing 2 |
| 543 | 0,0001579 | 0,00568 | 1494,99 | 696 | 2,15 | [1424268_at](https://www.affymetrix.com/LinkServlet?probeset=1424268_at) | [Smox](http://www.ncbi.nlm.nih.gov/entrez/query.fcgi?cmd=search&db=gene&term=Smox) | spermine oxidase |
| 544 | 0,000158 | 0,00568 | 121,17 | 7,78 | 15,57 | [1425368_a_at](https://www.affymetrix.com/LinkServlet?probeset=1425368_a_at) | [Numb](http://www.ncbi.nlm.nih.gov/entrez/query.fcgi?cmd=search&db=gene&term=Numb) | numb gene homolog (Drosophila) |
| 545 | 0,0001584 | 0,00568 | 2859,59 | 2202,88 | 1,3 | [1426535_at](https://www.affymetrix.com/LinkServlet?probeset=1426535_at) | [Poglut1](http://www.ncbi.nlm.nih.gov/entrez/query.fcgi?cmd=search&db=gene&term=Poglut1) | protein O-glucosyltransferase 1 |
| 546 | 0,0001586 | 0,00568 | 702,03 | 140,73 | 4,99 | [1421118_a_at](https://www.affymetrix.com/LinkServlet?probeset=1421118_a_at) | [Gpr56](http://www.ncbi.nlm.nih.gov/entrez/query.fcgi?cmd=search&db=gene&term=Gpr56) | G protein-coupled receptor 56 |
| 547 | 0,0001588 | 0,00568 | 615,57 | 254,87 | 2,42 | [1418145_at](https://www.affymetrix.com/LinkServlet?probeset=1418145_at) | [Tfip11](http://www.ncbi.nlm.nih.gov/entrez/query.fcgi?cmd=search&db=gene&term=Tfip11) | tuftelin interacting protein 11 |
| 548 | 0,0001589 | 0,00568 | 1018,61 | 3233,46 | 0,32 | [1451238_at](https://www.affymetrix.com/LinkServlet?probeset=1451238_at) | [Jkamp](http://www.ncbi.nlm.nih.gov/entrez/query.fcgi?cmd=search&db=gene&term=Jkamp) | JNK1/MAPK8-associated membrane protein |
| 549 | 0,000159 | 0,00568 | 2465,28 | 626,49 | 3,94 | [1417817_a_at](https://www.affymetrix.com/LinkServlet?probeset=1417817_a_at) | [Wwtr1](http://www.ncbi.nlm.nih.gov/entrez/query.fcgi?cmd=search&db=gene&term=Wwtr1) | WW domain containing transcription regulator 1 |
| 550 | 0,0001592 | 0,00568 | 4366,65 | 844,61 | 5,17 | [1417326_a_at](https://www.affymetrix.com/LinkServlet?probeset=1417326_a_at) | [Anapc11](http://www.ncbi.nlm.nih.gov/entrez/query.fcgi?cmd=search&db=gene&term=Anapc11) | anaphase promoting complex subunit 11 |
| 551 | 0,0001593 | 0,00568 | 5,35 | 59,11 | 0,09 | [1460417_at](https://www.affymetrix.com/LinkServlet?probeset=1460417_at) | [AB041803](http://www.ncbi.nlm.nih.gov/entrez/query.fcgi?cmd=search&db=gene&term=AB041803) | cDNA sequence AB041803 |
| 552 | 0,0001594 | 0,00568 | 16,76 | 58,65 | 0,29 | [1430610_at](https://www.affymetrix.com/LinkServlet?probeset=1430610_at) | [Mrpl38](http://www.ncbi.nlm.nih.gov/entrez/query.fcgi?cmd=search&db=gene&term=Mrpl38) | mitochondrial ribosomal protein L38 |
| 553 | 0,0001597 | 0,00568 | 915,63 | 421,02 | 2,17 | [1424066_at](https://www.affymetrix.com/LinkServlet?probeset=1424066_at) | [Dus3l](http://www.ncbi.nlm.nih.gov/entrez/query.fcgi?cmd=search&db=gene&term=Dus3l) | dihydrouridine synthase 3-like (S. cerevisiae) |
| 554 | 0,0001616 | 0,00572 | 5,7 | 145,65 | 0,039 | [1446512_at](https://www.affymetrix.com/LinkServlet?probeset=1446512_at) | [Zc3h15](http://www.ncbi.nlm.nih.gov/entrez/query.fcgi?cmd=search&db=gene&term=Zc3h15) | zinc finger CCCH-type containing 15 |
| 555 | 0,0001617 | 0,00572 | 3749,09 | 1620,81 | 2,31 | [1424376_at](https://www.affymetrix.com/LinkServlet?probeset=1424376_at) | [Cdc42ep1](http://www.ncbi.nlm.nih.gov/entrez/query.fcgi?cmd=search&db=gene&term=Cdc42ep1) | CDC42 effector protein (Rho GTPase binding) 1 |
| 556 | 0,0001618 | 0,00572 | 118,14 | 1477,55 | 0,08 | [1418289_at](https://www.affymetrix.com/LinkServlet?probeset=1418289_at) | [Nes](http://www.ncbi.nlm.nih.gov/entrez/query.fcgi?cmd=search&db=gene&term=Nes) | nestin |
| 557 | 0,0001624 | 0,00572 | 476,07 | 1880,91 | 0,25 | [1436266_x_at](https://www.affymetrix.com/LinkServlet?probeset=1436266_x_at) | [Cbx1](http://www.ncbi.nlm.nih.gov/entrez/query.fcgi?cmd=search&db=gene&term=Cbx1) | chromobox homolog 1 (Drosophila HP1 beta) |
| 558 | 0,0001626 | 0,00572 | 72,06 | 3091,35 | 0,023 | [1438476_a_at](https://www.affymetrix.com/LinkServlet?probeset=1438476_a_at) | [Chd4](http://www.ncbi.nlm.nih.gov/entrez/query.fcgi?cmd=search&db=gene&term=Chd4) | chromodomain helicase DNA binding protein 4 |
| 559 | 0,0001627 | 0,00572 | 13260,31 | 8821 | 1,5 | [1426037_a_at](https://www.affymetrix.com/LinkServlet?probeset=1426037_a_at) | [Rgs16](http://www.ncbi.nlm.nih.gov/entrez/query.fcgi?cmd=search&db=gene&term=Rgs16) | regulator of G-protein signaling 16 |
| 560 | 0,0001633 | 0,00574 | 309,66 | 15,95 | 19,41 | [1420887_a_at](https://www.affymetrix.com/LinkServlet?probeset=1420887_a_at) | [Bcl2l1](http://www.ncbi.nlm.nih.gov/entrez/query.fcgi?cmd=search&db=gene&term=Bcl2l1) | BCL2-like 1 |
| 561 | 0,0001637 | 0,00574 | 7,12 | 98 | 0,073 | [1434191_at](https://www.affymetrix.com/LinkServlet?probeset=1434191_at) | [Tmem195](http://www.ncbi.nlm.nih.gov/entrez/query.fcgi?cmd=search&db=gene&term=Tmem195) | transmembrane protein 195 |
| 562 | 0,0001651 | 0,00575 | 1136,29 | 303,53 | 3,74 | [1428777_at](https://www.affymetrix.com/LinkServlet?probeset=1428777_at) | [Spred1](http://www.ncbi.nlm.nih.gov/entrez/query.fcgi?cmd=search&db=gene&term=Spred1) | sprouty protein with EVH-1 domain 1, related sequence |
| 563 | 0,0001656 | 0,00575 | 4,74 | 28,71 | 0,16 | [1422836_at](https://www.affymetrix.com/LinkServlet?probeset=1422836_at) | [Mbnl3](http://www.ncbi.nlm.nih.gov/entrez/query.fcgi?cmd=search&db=gene&term=Mbnl3) | muscleblind-like 3 (Drosophila) |
| 564 | 0,0001656 | 0,00575 | 66,63 | 499,05 | 0,13 | [1442169_at](https://www.affymetrix.com/LinkServlet?probeset=1442169_at) | [Vldlr](http://www.ncbi.nlm.nih.gov/entrez/query.fcgi?cmd=search&db=gene&term=Vldlr) | very low density lipoprotein receptor |
| 565 | 0,0001656 | 0,00575 | 4,64 | 46,94 | 0,099 | [1417230_at](https://www.affymetrix.com/LinkServlet?probeset=1417230_at) | [Ralgps2](http://www.ncbi.nlm.nih.gov/entrez/query.fcgi?cmd=search&db=gene&term=Ralgps2) | Ral GEF with PH domain and SH3 binding motif 2 |
| 566 | 0,0001659 | 0,00575 | 25,69 | 1400,73 | 0,018 | [1448182_a_at](https://www.affymetrix.com/LinkServlet?probeset=1448182_a_at) | [Cd24a](http://www.ncbi.nlm.nih.gov/entrez/query.fcgi?cmd=search&db=gene&term=Cd24a) | CD24a antigen |
| 567 | 0,0001661 | 0,00575 | 255,71 | 575,91 | 0,44 | [1424617_at](https://www.affymetrix.com/LinkServlet?probeset=1424617_at) | [Ifi35](http://www.ncbi.nlm.nih.gov/entrez/query.fcgi?cmd=search&db=gene&term=Ifi35) | interferon-induced protein 35 |
| 568 | 0,0001661 | 0,00575 | 256,47 | 14,34 | 17,88 | [1425208_at](https://www.affymetrix.com/LinkServlet?probeset=1425208_at) | [Lbh](http://www.ncbi.nlm.nih.gov/entrez/query.fcgi?cmd=search&db=gene&term=Lbh) | limb-bud and heart |
| 569 | 0,0001663 | 0,00575 | 70,46 | 119,58 | 0,59 | [1421784_a_at](https://www.affymetrix.com/LinkServlet?probeset=1421784_a_at) | [Efna4](http://www.ncbi.nlm.nih.gov/entrez/query.fcgi?cmd=search&db=gene&term=Efna4) | ephrin A4 |
| 570 | 0,0001671 | 0,00577 | 10,15 | 527,57 | 0,019 | [1425521_at](https://www.affymetrix.com/LinkServlet?probeset=1425521_at) | [Paip1](http://www.ncbi.nlm.nih.gov/entrez/query.fcgi?cmd=search&db=gene&term=Paip1) | polyadenylate binding protein-interacting protein 1 |
| 571 | 0,0001674 | 0,00577 | 2666,03 | 15116,63 | 0,18 | [1448823_at](https://www.affymetrix.com/LinkServlet?probeset=1448823_at) | [Cxcl12](http://www.ncbi.nlm.nih.gov/entrez/query.fcgi?cmd=search&db=gene&term=Cxcl12) | chemokine (C-X-C motif) ligand 12 |
| 572 | 0,000168 | 0,00577 | 155,31 | 8,34 | 18,63 | [1422034_a_at](https://www.affymetrix.com/LinkServlet?probeset=1422034_a_at) | [Palm](http://www.ncbi.nlm.nih.gov/entrez/query.fcgi?cmd=search&db=gene&term=Palm) | paralemmin |
| 573 | 0,0001682 | 0,00577 | 246,11 | 1078,69 | 0,23 | [1422528_a_at](https://www.affymetrix.com/LinkServlet?probeset=1422528_a_at) | [Zfp36l1](http://www.ncbi.nlm.nih.gov/entrez/query.fcgi?cmd=search&db=gene&term=Zfp36l1) | zinc finger protein 36, C3H type-like 1 |
| 574 | 0,0001689 | 0,00578 | 162,06 | 19,91 | 8,14 | [1450183_a_at](https://www.affymetrix.com/LinkServlet?probeset=1450183_a_at) | [Sh2b3](http://www.ncbi.nlm.nih.gov/entrez/query.fcgi?cmd=search&db=gene&term=Sh2b3) | SH2B adaptor protein 3 |
| 575 | 0,000169 | 0,00578 | 475,39 | 279,42 | 1,7 | [1436360_at](https://www.affymetrix.com/LinkServlet?probeset=1436360_at) | [Zscan22](http://www.ncbi.nlm.nih.gov/entrez/query.fcgi?cmd=search&db=gene&term=Zscan22) | zinc finger and SCAN domain containing 22 |
| 576 | 0,0001702 | 0,00581 | 1826,85 | 562,2 | 3,25 | [1417179_at](https://www.affymetrix.com/LinkServlet?probeset=1417179_at) | [Tspan5](http://www.ncbi.nlm.nih.gov/entrez/query.fcgi?cmd=search&db=gene&term=Tspan5) | tetraspanin 5 |
| 577 | 0,0001706 | 0,00581 | 4,69 | 44,19 | 0,11 | [1452532_x_at](https://www.affymetrix.com/LinkServlet?probeset=1452532_x_at) | [Ceacam1](http://www.ncbi.nlm.nih.gov/entrez/query.fcgi?cmd=search&db=gene&term=Ceacam1) | carcinoembryonic antigen-related cell adhesion molecule 1 |
| 578 | 0,0001716 | 0,00584 | 674,62 | 2563,23 | 0,26 | [1458440_at](https://www.affymetrix.com/LinkServlet?probeset=1458440_at) | [Specc1](http://www.ncbi.nlm.nih.gov/entrez/query.fcgi?cmd=search&db=gene&term=Specc1) | sperm antigen with calponin homology and coiled-coil domains 1 |
| 579 | 0,0001719 | 0,00584 | 9,51 | 268,74 | 0,035 | [1434957_at](https://www.affymetrix.com/LinkServlet?probeset=1434957_at) | [Cdon](http://www.ncbi.nlm.nih.gov/entrez/query.fcgi?cmd=search&db=gene&term=Cdon) | cell adhesion molecule-related/down-regulated by oncogenes |
| 580 | 0,0001726 | 0,00584 | 124,36 | 55,61 | 2,24 | [1439008_at](https://www.affymetrix.com/LinkServlet?probeset=1439008_at) | [Zfp319](http://www.ncbi.nlm.nih.gov/entrez/query.fcgi?cmd=search&db=gene&term=Zfp319) | zinc finger protein 319 |
| 581 | 0,0001729 | 0,00584 | 36,43 | 752,19 | 0,048 | [1450093_s_at](https://www.affymetrix.com/LinkServlet?probeset=1450093_s_at) | [Zbtb7a](http://www.ncbi.nlm.nih.gov/entrez/query.fcgi?cmd=search&db=gene&term=Zbtb7a) | zinc finger and BTB domain containing 7a |
| 582 | 0,0001731 | 0,00584 | 383,31 | 3268,74 | 0,12 | [1434303_at](https://www.affymetrix.com/LinkServlet?probeset=1434303_at) | [Raph1](http://www.ncbi.nlm.nih.gov/entrez/query.fcgi?cmd=search&db=gene&term=Raph1) | Ras association (RalGDS/AF-6) and pleckstrin homology domains 1 |
| 583 | 0,0001735 | 0,00584 | 2234,32 | 195,06 | 11,45 | [1435602_at](https://www.affymetrix.com/LinkServlet?probeset=1435602_at) | [Sephs2](http://www.ncbi.nlm.nih.gov/entrez/query.fcgi?cmd=search&db=gene&term=Sephs2) | selenophosphate synthetase 2 |
| 584 | 0,0001738 | 0,00584 | 2461,28 | 1134,15 | 2,17 | [1455448_at](https://www.affymetrix.com/LinkServlet?probeset=1455448_at) | [Dagla](http://www.ncbi.nlm.nih.gov/entrez/query.fcgi?cmd=search&db=gene&term=Dagla) | diacylglycerol lipase, alpha |
| 585 | 0,0001742 | 0,00584 | 185,74 | 289,03 | 0,64 | [1428482_at](https://www.affymetrix.com/LinkServlet?probeset=1428482_at) | [Akap10](http://www.ncbi.nlm.nih.gov/entrez/query.fcgi?cmd=search&db=gene&term=Akap10) | A kinase (PRKA) anchor protein 10 |
| 586 | 0,0001744 | 0,00584 | 1635,75 | 2823,49 | 0,58 | [1426476_at](https://www.affymetrix.com/LinkServlet?probeset=1426476_at) | [Rasa1](http://www.ncbi.nlm.nih.gov/entrez/query.fcgi?cmd=search&db=gene&term=Rasa1) | RAS p21 protein activator 1 |
| 587 | 0,0001748 | 0,00584 | 6,41 | 56 | 0,11 | [1440658_at](https://www.affymetrix.com/LinkServlet?probeset=1440658_at) | [Ammecr1l](http://www.ncbi.nlm.nih.gov/entrez/query.fcgi?cmd=search&db=gene&term=Ammecr1l) | AMME chromosomal region gene 1-like |
| 588 | 0,0001752 | 0,00584 | 570,1 | 139,34 | 4,09 | [1424117_at](https://www.affymetrix.com/LinkServlet?probeset=1424117_at) | [BC056474](http://www.ncbi.nlm.nih.gov/entrez/query.fcgi?cmd=search&db=gene&term=BC056474) | cDNA sequence BC056474 |
| 589 | 0,0001754 | 0,00584 | 1717,02 | 619,5 | 2,77 | [1452149_at](https://www.affymetrix.com/LinkServlet?probeset=1452149_at) | [Ube3b](http://www.ncbi.nlm.nih.gov/entrez/query.fcgi?cmd=search&db=gene&term=Ube3b) | ubiquitin protein ligase E3B |
| 590 | 0,0001755 | 0,00584 | 181,25 | 19,74 | 9,18 | [1452979_at](https://www.affymetrix.com/LinkServlet?probeset=1452979_at) | [Atat1](http://www.ncbi.nlm.nih.gov/entrez/query.fcgi?cmd=search&db=gene&term=Atat1) | alpha tubulin acetyltransferase 1 |
| 591 | 0,000176 | 0,00584 | 3284,19 | 1187,53 | 2,77 | [1448525_a_at](https://www.affymetrix.com/LinkServlet?probeset=1448525_a_at) | [Bnip3l](http://www.ncbi.nlm.nih.gov/entrez/query.fcgi?cmd=search&db=gene&term=Bnip3l) | BCL2/adenovirus E1B interacting protein 3-like |
| 592 | 0,000176 | 0,00584 | 80,57 | 1192,6 | 0,068 | [1424529_s_at](https://www.affymetrix.com/LinkServlet?probeset=1424529_s_at) | [Cgref1](http://www.ncbi.nlm.nih.gov/entrez/query.fcgi?cmd=search&db=gene&term=Cgref1) | cell growth regulator with EF hand domain 1 |
| 593 | 0,0001761 | 0,00584 | 6507,41 | 4562,14 | 1,43 | [1426400_a_at](https://www.affymetrix.com/LinkServlet?probeset=1426400_a_at) | [Capns1](http://www.ncbi.nlm.nih.gov/entrez/query.fcgi?cmd=search&db=gene&term=Capns1) | calpain, small subunit 1 |
| 594 | 0,0001764 | 0,00584 | 2796,63 | 4123,24 | 0,68 | [1428385_at](https://www.affymetrix.com/LinkServlet?probeset=1428385_at) | [8-mars](http://www.ncbi.nlm.nih.gov/entrez/query.fcgi?cmd=search&db=gene&term=March8) | membrane-associated ring finger (C3HC4) 8 |
| 595 | 0,0001775 | 0,00587 | 2474,42 | 357,54 | 6,92 | [1418396_at](https://www.affymetrix.com/LinkServlet?probeset=1418396_at) | [Gpsm3](http://www.ncbi.nlm.nih.gov/entrez/query.fcgi?cmd=search&db=gene&term=Gpsm3) | G-protein signalling modulator 3 (AGS3-like, C. elegans) |
| 596 | 0,0001787 | 0,00589 | 403,79 | 11036 | 0,037 | [1437165_a_at](https://www.affymetrix.com/LinkServlet?probeset=1437165_a_at) | [Pcolce](http://www.ncbi.nlm.nih.gov/entrez/query.fcgi?cmd=search&db=gene&term=Pcolce) | procollagen C-endopeptidase enhancer protein |
| 597 | 0,0001788 | 0,00589 | 255,22 | 564,97 | 0,45 | [1435836_at](https://www.affymetrix.com/LinkServlet?probeset=1435836_at) | [Pdk1](http://www.ncbi.nlm.nih.gov/entrez/query.fcgi?cmd=search&db=gene&term=Pdk1) | pyruvate dehydrogenase kinase, isoenzyme 1 |
| 598 | 0,0001794 | 0,00589 | 12,58 | 272,15 | 0,046 | [1444589_at](https://www.affymetrix.com/LinkServlet?probeset=1444589_at) | [Gm4944](http://www.ncbi.nlm.nih.gov/entrez/query.fcgi?cmd=search&db=gene&term=Gm4944) | predicted gene 4944 |
| 599 | 0,0001797 | 0,00589 | 1183,21 | 648,7 | 1,82 | [1448114_a_at](https://www.affymetrix.com/LinkServlet?probeset=1448114_a_at) | [Trmt2a](http://www.ncbi.nlm.nih.gov/entrez/query.fcgi?cmd=search&db=gene&term=Trmt2a) | TRM2 tRNA methyltransferase 2 homolog A (S. cerevisiae) |
| 600 | 0,0001798 | 0,00589 | 1042,56 | 422,37 | 2,47 | [1433436_s_at](https://www.affymetrix.com/LinkServlet?probeset=1433436_s_at) | [Thtpa](http://www.ncbi.nlm.nih.gov/entrez/query.fcgi?cmd=search&db=gene&term=Thtpa) | thiamine triphosphatase |
| 601 | 0,000181 | 0,00589 | 241,44 | 35,55 | 6,79 | [1435202_at](https://www.affymetrix.com/LinkServlet?probeset=1435202_at) | [Zfp574](http://www.ncbi.nlm.nih.gov/entrez/query.fcgi?cmd=search&db=gene&term=Zfp574) | zinc finger protein 574 |
| 602 | 0,0001811 | 0,00589 | 2799,14 | 8566,17 | 0,33 | [1445597_s_at](https://www.affymetrix.com/LinkServlet?probeset=1445597_s_at) | [Pla2g16](http://www.ncbi.nlm.nih.gov/entrez/query.fcgi?cmd=search&db=gene&term=Pla2g16) | phospholipase A2, group XVI |
| 603 | 0,0001815 | 0,00589 | 1446,57 | 396,03 | 3,65 | [1416353_at](https://www.affymetrix.com/LinkServlet?probeset=1416353_at) | [Nr1h2](http://www.ncbi.nlm.nih.gov/entrez/query.fcgi?cmd=search&db=gene&term=Nr1h2) | nuclear receptor subfamily 1, group H, member 2 |
| 604 | 0,0001815 | 0,00589 | 20,85 | 93,34 | 0,22 | [1442358_at](https://www.affymetrix.com/LinkServlet?probeset=1442358_at) | [AA409587](http://www.ncbi.nlm.nih.gov/entrez/query.fcgi?cmd=search&db=gene&term=AA409587) | expressed sequence AA409587 |
| 605 | 0,0001816 | 0,00589 | 3594,7 | 1967,87 | 1,83 | [1415806_at](https://www.affymetrix.com/LinkServlet?probeset=1415806_at) | [Plat](http://www.ncbi.nlm.nih.gov/entrez/query.fcgi?cmd=search&db=gene&term=Plat) | plasminogen activator, tissue |
| 606 | 0,0001816 | 0,00589 | 78,7 | 814,97 | 0,097 | [1452203_at](https://www.affymetrix.com/LinkServlet?probeset=1452203_at) | [Obfc2a](http://www.ncbi.nlm.nih.gov/entrez/query.fcgi?cmd=search&db=gene&term=Obfc2a) | oligonucleotide/oligosaccharide-binding fold containing 2A |
| 607 | 0,0001823 | 0,0059 | 127,67 | 17,82 | 7,16 | [1428150_at](https://www.affymetrix.com/LinkServlet?probeset=1428150_at) | [Coro7](http://www.ncbi.nlm.nih.gov/entrez/query.fcgi?cmd=search&db=gene&term=Coro7) | coronin 7 |
| 608 | 0,0001825 | 0,0059 | 1138,22 | 269,05 | 4,23 | [1453119_at](https://www.affymetrix.com/LinkServlet?probeset=1453119_at) | [Otud1](http://www.ncbi.nlm.nih.gov/entrez/query.fcgi?cmd=search&db=gene&term=Otud1) | OTU domain containing 1 |
| 609 | 0,000183 | 0,0059 | 178,27 | 20,99 | 8,49 | [1427565_a_at](https://www.affymetrix.com/LinkServlet?probeset=1427565_a_at) | [Abcc5](http://www.ncbi.nlm.nih.gov/entrez/query.fcgi?cmd=search&db=gene&term=Abcc5) | ATP-binding cassette, sub-family C (CFTR/MRP), member 5 |
| 610 | 0,0001831 | 0,0059 | 1292,31 | 447,79 | 2,89 | [1442006_at](https://www.affymetrix.com/LinkServlet?probeset=1442006_at) | [NA](http://www.ncbi.nlm.nih.gov/entrez/query.fcgi?cmd=search&db=gene&term=NA) | NA |
| 611 | 0,0001841 | 0,00593 | 422,33 | 150,41 | 2,81 | [1428148_s_at](https://www.affymetrix.com/LinkServlet?probeset=1428148_s_at) | [Coro7](http://www.ncbi.nlm.nih.gov/entrez/query.fcgi?cmd=search&db=gene&term=Coro7) | coronin 7 |
| 612 | 0,0001845 | 0,00593 | 329,94 | 52,29 | 6,31 | [1429325_at](https://www.affymetrix.com/LinkServlet?probeset=1429325_at) | [Poc1b](http://www.ncbi.nlm.nih.gov/entrez/query.fcgi?cmd=search&db=gene&term=Poc1b) | POC1 centriolar protein homolog B (Chlamydomonas) |
| 613 | 0,0001847 | 0,00593 | 2024,79 | 5410,25 | 0,37 | [1438957_x_at](https://www.affymetrix.com/LinkServlet?probeset=1438957_x_at) | [Cds2](http://www.ncbi.nlm.nih.gov/entrez/query.fcgi?cmd=search&db=gene&term=Cds2) | CDP-diacylglycerol synthase (phosphatidate cytidylyltransferase) 2 |
| 614 | 0,0001855 | 0,00594 | 361,25 | 159,73 | 2,26 | [1416307_at](https://www.affymetrix.com/LinkServlet?probeset=1416307_at) | [Ap1m1](http://www.ncbi.nlm.nih.gov/entrez/query.fcgi?cmd=search&db=gene&term=Ap1m1) | adaptor-related protein complex AP-1, mu subunit 1 |
| 615 | 0,0001858 | 0,00594 | 111,83 | 586,98 | 0,19 | [1431076_at](https://www.affymetrix.com/LinkServlet?probeset=1431076_at) | [Add2](http://www.ncbi.nlm.nih.gov/entrez/query.fcgi?cmd=search&db=gene&term=Add2) | adducin 2 (beta) |
| 616 | 0,0001859 | 0,00594 | 8,46 | 82,87 | 0,1 | [1416879_at](https://www.affymetrix.com/LinkServlet?probeset=1416879_at) | [Mrpl51](http://www.ncbi.nlm.nih.gov/entrez/query.fcgi?cmd=search&db=gene&term=Mrpl51) | mitochondrial ribosomal protein L51 |
| 617 | 0,0001873 | 0,00597 | 19,3 | 204,78 | 0,094 | [1417961_a_at](https://www.affymetrix.com/LinkServlet?probeset=1417961_a_at) | [Trim30a](http://www.ncbi.nlm.nih.gov/entrez/query.fcgi?cmd=search&db=gene&term=Trim30a) | tripartite motif-containing 30A |
| 618 | 0,0001884 | 0,006 | 730,87 | 357,73 | 2,04 | [1451545_at](https://www.affymetrix.com/LinkServlet?probeset=1451545_at) | [Tdrd3](http://www.ncbi.nlm.nih.gov/entrez/query.fcgi?cmd=search&db=gene&term=Tdrd3) | tudor domain containing 3 |
| 619 | 0,0001892 | 0,00601 | 1050,84 | 222,26 | 4,73 | [1416863_at](https://www.affymetrix.com/LinkServlet?probeset=1416863_at) | [Abhd8](http://www.ncbi.nlm.nih.gov/entrez/query.fcgi?cmd=search&db=gene&term=Abhd8) | abhydrolase domain containing 8 |
| 620 | 0,0001901 | 0,00603 | 107,79 | 228,61 | 0,47 | [1437077_at](https://www.affymetrix.com/LinkServlet?probeset=1437077_at) | [Dcun1d2](http://www.ncbi.nlm.nih.gov/entrez/query.fcgi?cmd=search&db=gene&term=Dcun1d2) | DCN1, defective in cullin neddylation 1, domain containing 2 (S. cerevisiae) |
| 621 | 0,0001912 | 0,00606 | 1030,07 | 514,73 | 2 | [1426747_at](https://www.affymetrix.com/LinkServlet?probeset=1426747_at) | [Abcf3](http://www.ncbi.nlm.nih.gov/entrez/query.fcgi?cmd=search&db=gene&term=Abcf3) | ATP-binding cassette, sub-family F (GCN20), member 3 |
| 622 | 0,0001922 | 0,00606 | 568,66 | 124,16 | 4,58 | [1448477_at](https://www.affymetrix.com/LinkServlet?probeset=1448477_at) | [Chst12](http://www.ncbi.nlm.nih.gov/entrez/query.fcgi?cmd=search&db=gene&term=Chst12) | carbohydrate sulfotransferase 12 |
| 623 | 0,0001923 | 0,00606 | 24,69 | 629,47 | 0,039 | [1427311_at](https://www.affymetrix.com/LinkServlet?probeset=1427311_at) | [Bptf](http://www.ncbi.nlm.nih.gov/entrez/query.fcgi?cmd=search&db=gene&term=Bptf) | bromodomain PHD finger transcription factor |
| 624 | 0,0001927 | 0,00606 | 5,53 | 121,57 | 0,045 | [1439906_at](https://www.affymetrix.com/LinkServlet?probeset=1439906_at) | [NA](http://www.ncbi.nlm.nih.gov/entrez/query.fcgi?cmd=search&db=gene&term=NA) | NA |
| 625 | 0,0001928 | 0,00606 | 24,61 | 92,24 | 0,27 | [1422148_at](https://www.affymetrix.com/LinkServlet?probeset=1422148_at) | [Matn3](http://www.ncbi.nlm.nih.gov/entrez/query.fcgi?cmd=search&db=gene&term=Matn3) | matrilin 3 |
| 626 | 0,0001929 | 0,00606 | 3230,13 | 1184,19 | 2,73 | [1425615_a_at](https://www.affymetrix.com/LinkServlet?probeset=1425615_a_at) | [Pck2](http://www.ncbi.nlm.nih.gov/entrez/query.fcgi?cmd=search&db=gene&term=Pck2) | phosphoenolpyruvate carboxykinase 2 (mitochondrial) |
| 627 | 0,0001933 | 0,00606 | 955,78 | 2175,06 | 0,44 | [1427310_at](https://www.affymetrix.com/LinkServlet?probeset=1427310_at) | [Bptf](http://www.ncbi.nlm.nih.gov/entrez/query.fcgi?cmd=search&db=gene&term=Bptf) | bromodomain PHD finger transcription factor |
| 628 | 0,0001934 | 0,00606 | 1862,19 | 988,12 | 1,88 | [1425830_a_at](https://www.affymetrix.com/LinkServlet?probeset=1425830_a_at) | [Cinp](http://www.ncbi.nlm.nih.gov/entrez/query.fcgi?cmd=search&db=gene&term=Cinp) | cyclin-dependent kinase 2 interacting protein |
| 629 | 0,0001945 | 0,00607 | 164,12 | 549,23 | 0,3 | [1455801_x_at](https://www.affymetrix.com/LinkServlet?probeset=1455801_x_at) | [Tbcd](http://www.ncbi.nlm.nih.gov/entrez/query.fcgi?cmd=search&db=gene&term=Tbcd) | tubulin-specific chaperone d |
| 630 | 0,000195 | 0,00607 | 9831,39 | 1032,48 | 9,52 | [1423053_at](https://www.affymetrix.com/LinkServlet?probeset=1423053_at) | [Arf4](http://www.ncbi.nlm.nih.gov/entrez/query.fcgi?cmd=search&db=gene&term=Arf4) | ADP-ribosylation factor 4 |
| 631 | 0,0001951 | 0,00607 | 2582,24 | 839,98 | 3,07 | [1448138_at](https://www.affymetrix.com/LinkServlet?probeset=1448138_at) | [Ppp2r4](http://www.ncbi.nlm.nih.gov/entrez/query.fcgi?cmd=search&db=gene&term=Ppp2r4) | protein phosphatase 2A, regulatory subunit B (PR 53) |
| 632 | 0,0001952 | 0,00607 | 19,02 | 893,05 | 0,021 | [1423249_at](https://www.affymetrix.com/LinkServlet?probeset=1423249_at) | [Nktr](http://www.ncbi.nlm.nih.gov/entrez/query.fcgi?cmd=search&db=gene&term=Nktr) | natural killer tumor recognition sequence |
| 633 | 0,0001954 | 0,00607 | 2104,98 | 3899,3 | 0,54 | [1426782_at](https://www.affymetrix.com/LinkServlet?probeset=1426782_at) | [Gpr125](http://www.ncbi.nlm.nih.gov/entrez/query.fcgi?cmd=search&db=gene&term=Gpr125) | G protein-coupled receptor 125 |
| 634 | 0,0001959 | 0,00607 | 904,32 | 2314,55 | 0,39 | [1426745_at](https://www.affymetrix.com/LinkServlet?probeset=1426745_at) | [Rnasek](http://www.ncbi.nlm.nih.gov/entrez/query.fcgi?cmd=search&db=gene&term=Rnasek) | ribonuclease, RNase K |
| 635 | 0,0001959 | 0,00607 | 33,62 | 1014,66 | 0,033 | [1440282_at](https://www.affymetrix.com/LinkServlet?probeset=1440282_at) | [Tulp4](http://www.ncbi.nlm.nih.gov/entrez/query.fcgi?cmd=search&db=gene&term=Tulp4) | tubby like protein 4 |
| 636 | 0,0001966 | 0,00608 | 104,35 | 19,97 | 5,23 | [1424849_at](https://www.affymetrix.com/LinkServlet?probeset=1424849_at) | [Wdr62](http://www.ncbi.nlm.nih.gov/entrez/query.fcgi?cmd=search&db=gene&term=Wdr62) | WD repeat domain 62 |
| 637 | 0,0001974 | 0,00609 | 665,56 | 438,4 | 1,52 | [1416173_at](https://www.affymetrix.com/LinkServlet?probeset=1416173_at) | [Pes1](http://www.ncbi.nlm.nih.gov/entrez/query.fcgi?cmd=search&db=gene&term=Pes1) | pescadillo homolog 1, containing BRCT domain (zebrafish) |
| 638 | 0,0001981 | 0,00609 | 100,6 | 220,4 | 0,46 | [1434576_at](https://www.affymetrix.com/LinkServlet?probeset=1434576_at) | [Tsga14](http://www.ncbi.nlm.nih.gov/entrez/query.fcgi?cmd=search&db=gene&term=Tsga14) | testis specific gene A14 |
| 639 | 0,0001981 | 0,00609 | 17,81 | 216,44 | 0,082 | [1427905_at](https://www.affymetrix.com/LinkServlet?probeset=1427905_at) | [1810063B07Rik](http://www.ncbi.nlm.nih.gov/entrez/query.fcgi?cmd=search&db=gene&term=1810063B07Rik) | RIKEN cDNA 1810063B07 gene |
| 640 | 0,0001982 | 0,00609 | 1763,69 | 920,39 | 1,92 | [1418467_at](https://www.affymetrix.com/LinkServlet?probeset=1418467_at) | [Smarcd3](http://www.ncbi.nlm.nih.gov/entrez/query.fcgi?cmd=search&db=gene&term=Smarcd3) | SWI/SNF related, matrix associated, actin dependent regulator of chromatin, subfamily d, member 3 |
| 641 | 0,0001991 | 0,0061 | 548,98 | 2566,91 | 0,21 | [1460338_a_at](https://www.affymetrix.com/LinkServlet?probeset=1460338_a_at) | [Crlf3](http://www.ncbi.nlm.nih.gov/entrez/query.fcgi?cmd=search&db=gene&term=Crlf3) | cytokine receptor-like factor 3 |
| 642 | 0,0001991 | 0,0061 | 671,79 | 1469,38 | 0,46 | [1423162_s_at](https://www.affymetrix.com/LinkServlet?probeset=1423162_s_at) | [Spred1](http://www.ncbi.nlm.nih.gov/entrez/query.fcgi?cmd=search&db=gene&term=Spred1) | sprouty protein with EVH-1 domain 1, related sequence |
| 643 | 0,0001998 | 0,0061 | 506,6 | 209,84 | 2,41 | [1416960_at](https://www.affymetrix.com/LinkServlet?probeset=1416960_at) | [B3gat3](http://www.ncbi.nlm.nih.gov/entrez/query.fcgi?cmd=search&db=gene&term=B3gat3) | beta-1,3-glucuronyltransferase 3 (glucuronosyltransferase I) |
| 644 | 0,0001998 | 0,0061 | 396,51 | 22,3 | 17,78 | [1428513_at](https://www.affymetrix.com/LinkServlet?probeset=1428513_at) | [Calcoco1](http://www.ncbi.nlm.nih.gov/entrez/query.fcgi?cmd=search&db=gene&term=Calcoco1) | calcium binding and coiled coil domain 1 |
| 645 | 0,0002008 | 0,00612 | 3322,53 | 1388,98 | 2,39 | [1415738_at](https://www.affymetrix.com/LinkServlet?probeset=1415738_at) | [Txndc12](http://www.ncbi.nlm.nih.gov/entrez/query.fcgi?cmd=search&db=gene&term=Txndc12) | thioredoxin domain containing 12 (endoplasmic reticulum) |
| 646 | 0,0002015 | 0,00613 | 951,55 | 419,46 | 2,27 | [1418401_a_at](https://www.affymetrix.com/LinkServlet?probeset=1418401_a_at) | [Dusp16](http://www.ncbi.nlm.nih.gov/entrez/query.fcgi?cmd=search&db=gene&term=Dusp16) | dual specificity phosphatase 16 |
| 647 | 0,0002032 | 0,00617 | 215,55 | 42,5 | 5,07 | [1436241_s_at](https://www.affymetrix.com/LinkServlet?probeset=1436241_s_at) | [Hira](http://www.ncbi.nlm.nih.gov/entrez/query.fcgi?cmd=search&db=gene&term=Hira) | histone cell cycle regulation defective homolog A (S. cerevisiae) |
| 648 | 0,0002046 | 0,00617 | 499,09 | 174,53 | 2,86 | [1435886_at](https://www.affymetrix.com/LinkServlet?probeset=1435886_at) | [Dnajb12](http://www.ncbi.nlm.nih.gov/entrez/query.fcgi?cmd=search&db=gene&term=Dnajb12) | DnaJ (Hsp40) homolog, subfamily B, member 12 |
| 649 | 0,0002053 | 0,00617 | 494,55 | 8,33 | 59,38 | [1434755_at](https://www.affymetrix.com/LinkServlet?probeset=1434755_at) | [Coro2b](http://www.ncbi.nlm.nih.gov/entrez/query.fcgi?cmd=search&db=gene&term=Coro2b) | coronin, actin binding protein, 2B |
| 650 | 0,0002054 | 0,00617 | 134,52 | 41,03 | 3,28 | [1443976_at](https://www.affymetrix.com/LinkServlet?probeset=1443976_at) | [Cdk5rap2](http://www.ncbi.nlm.nih.gov/entrez/query.fcgi?cmd=search&db=gene&term=Cdk5rap2) | CDK5 regulatory subunit associated protein 2 |
| 651 | 0,0002059 | 0,00617 | 689,71 | 244,45 | 2,82 | [1460617_s_at](https://www.affymetrix.com/LinkServlet?probeset=1460617_s_at) | [Rab6b](http://www.ncbi.nlm.nih.gov/entrez/query.fcgi?cmd=search&db=gene&term=Rab6b) | RAB6B, member RAS oncogene family |
| 652 | 0,0002061 | 0,00617 | 439,75 | 1080,55 | 0,41 | [1422431_at](https://www.affymetrix.com/LinkServlet?probeset=1422431_at) | [Magee1](http://www.ncbi.nlm.nih.gov/entrez/query.fcgi?cmd=search&db=gene&term=Magee1) | melanoma antigen, family E, 1 |
| 653 | 0,0002063 | 0,00617 | 4363,89 | 3088,35 | 1,41 | [1448100_at](https://www.affymetrix.com/LinkServlet?probeset=1448100_at) | [4833439L19Rik](http://www.ncbi.nlm.nih.gov/entrez/query.fcgi?cmd=search&db=gene&term=4833439L19Rik) | RIKEN cDNA 4833439L19 gene |
| 654 | 0,0002066 | 0,00617 | 431,83 | 4082,02 | 0,11 | [1451485_at](https://www.affymetrix.com/LinkServlet?probeset=1451485_at) | [Luc7l3](http://www.ncbi.nlm.nih.gov/entrez/query.fcgi?cmd=search&db=gene&term=Luc7l3) | LUC7-like 3 (S. cerevisiae) |
| 655 | 0,000207 | 0,00617 | 6853,59 | 2435,31 | 2,81 | [1449041_a_at](https://www.affymetrix.com/LinkServlet?probeset=1449041_a_at) | [Trip6](http://www.ncbi.nlm.nih.gov/entrez/query.fcgi?cmd=search&db=gene&term=Trip6) | thyroid hormone receptor interactor 6 |
| 656 | 0,000207 | 0,00617 | 10,98 | 162,72 | 0,067 | [1418060_a_at](https://www.affymetrix.com/LinkServlet?probeset=1418060_a_at) | [Mapk7](http://www.ncbi.nlm.nih.gov/entrez/query.fcgi?cmd=search&db=gene&term=Mapk7) | mitogen-activated protein kinase 7 |
| 657 | 0,0002071 | 0,00617 | 5,08 | 147,7 | 0,034 | [1437673_at](https://www.affymetrix.com/LinkServlet?probeset=1437673_at) | [Wnt5a](http://www.ncbi.nlm.nih.gov/entrez/query.fcgi?cmd=search&db=gene&term=Wnt5a) | wingless-related MMTV integration site 5A |
| 658 | 0,0002072 | 0,00617 | 1283,45 | 682,23 | 1,88 | [1426643_at](https://www.affymetrix.com/LinkServlet?probeset=1426643_at) | [Elp3](http://www.ncbi.nlm.nih.gov/entrez/query.fcgi?cmd=search&db=gene&term=Elp3) | elongation protein 3 homolog (S. cerevisiae) |
| 659 | 0,0002073 | 0,00617 | 83,47 | 6,84 | 12,21 | [1446072_at](https://www.affymetrix.com/LinkServlet?probeset=1446072_at) | [4632415L05Rik](http://www.ncbi.nlm.nih.gov/entrez/query.fcgi?cmd=search&db=gene&term=4632415L05Rik) | RRS1 ribosome biogenesis regulator homolog pseudogene |
| 660 | 0,0002074 | 0,00617 | 713,52 | 518,33 | 1,38 | [1422692_at](https://www.affymetrix.com/LinkServlet?probeset=1422692_at) | [Sub1](http://www.ncbi.nlm.nih.gov/entrez/query.fcgi?cmd=search&db=gene&term=Sub1) | SUB1 homolog (S. cerevisiae) |
| 661 | 0,0002075 | 0,00617 | 3939,61 | 1508,52 | 2,61 | [1426963_at](https://www.affymetrix.com/LinkServlet?probeset=1426963_at) | [Pacs2](http://www.ncbi.nlm.nih.gov/entrez/query.fcgi?cmd=search&db=gene&term=Pacs2) | phosphofurin acidic cluster sorting protein 2 |
| 662 | 0,0002078 | 0,00617 | 4213,18 | 1538,54 | 2,74 | [1423448_at](https://www.affymetrix.com/LinkServlet?probeset=1423448_at) | [Rab11b](http://www.ncbi.nlm.nih.gov/entrez/query.fcgi?cmd=search&db=gene&term=Rab11b) | RAB11B, member RAS oncogene family |
| 663 | 0,0002079 | 0,00617 | 1010,14 | 601,67 | 1,68 | [1451309_at](https://www.affymetrix.com/LinkServlet?probeset=1451309_at) | [Arhgap1](http://www.ncbi.nlm.nih.gov/entrez/query.fcgi?cmd=search&db=gene&term=Arhgap1) | Rho GTPase activating protein 1 |
| 664 | 0,0002087 | 0,00618 | 27,11 | 133,57 | 0,2 | [1455799_at](https://www.affymetrix.com/LinkServlet?probeset=1455799_at) | [Rorb](http://www.ncbi.nlm.nih.gov/entrez/query.fcgi?cmd=search&db=gene&term=Rorb) | RAR-related orphan receptor beta |
| 665 | 0,0002088 | 0,00618 | 13892,44 | 3801,22 | 3,65 | [1423628_s_at](https://www.affymetrix.com/LinkServlet?probeset=1423628_s_at) | [NA](http://www.ncbi.nlm.nih.gov/entrez/query.fcgi?cmd=search&db=gene&term=NA) | NA |
| 666 | 0,0002096 | 0,00619 | 15535,6 | 11585,74 | 1,34 | [1437802_x_at](https://www.affymetrix.com/LinkServlet?probeset=1437802_x_at) | [Morf4l1](http://www.ncbi.nlm.nih.gov/entrez/query.fcgi?cmd=search&db=gene&term=Morf4l1) | mortality factor 4 like 1 |
| 667 | 0,0002108 | 0,00621 | 65,09 | 14,13 | 4,61 | [1455924_at](https://www.affymetrix.com/LinkServlet?probeset=1455924_at) | [Rab6b](http://www.ncbi.nlm.nih.gov/entrez/query.fcgi?cmd=search&db=gene&term=Rab6b) | RAB6B, member RAS oncogene family |
| 668 | 0,0002108 | 0,00621 | 370,4 | 33,27 | 11,13 | [1424668_a_at](https://www.affymetrix.com/LinkServlet?probeset=1424668_a_at) | [Cux1](http://www.ncbi.nlm.nih.gov/entrez/query.fcgi?cmd=search&db=gene&term=Cux1) | cut-like homeobox 1 |
| 669 | 0,0002131 | 0,00625 | 2661,03 | 1218,23 | 2,18 | [1423698_at](https://www.affymetrix.com/LinkServlet?probeset=1423698_at) | [Ncaph2](http://www.ncbi.nlm.nih.gov/entrez/query.fcgi?cmd=search&db=gene&term=Ncaph2) | non-SMC condensin II complex, subunit H2 |
| 670 | 0,0002136 | 0,00625 | 1542,09 | 732,04 | 2,11 | [1434563_at](https://www.affymetrix.com/LinkServlet?probeset=1434563_at) | [Rps6kc1](http://www.ncbi.nlm.nih.gov/entrez/query.fcgi?cmd=search&db=gene&term=Rps6kc1) | ribosomal protein S6 kinase polypeptide 1 |
| 671 | 0,0002136 | 0,00625 | 29,67 | 439,06 | 0,068 | [1455876_at](https://www.affymetrix.com/LinkServlet?probeset=1455876_at) | [Slc4a7](http://www.ncbi.nlm.nih.gov/entrez/query.fcgi?cmd=search&db=gene&term=Slc4a7) | solute carrier family 4, sodium bicarbonate cotransporter, member 7 |
| 672 | 0,0002137 | 0,00625 | 646,76 | 247,33 | 2,61 | [1450844_at](https://www.affymetrix.com/LinkServlet?probeset=1450844_at) | [Stx6](http://www.ncbi.nlm.nih.gov/entrez/query.fcgi?cmd=search&db=gene&term=Stx6) | syntaxin 6 |
| 673 | 0,0002147 | 0,00627 | 5,93 | 65,51 | 0,091 | [1458706_at](https://www.affymetrix.com/LinkServlet?probeset=1458706_at) | [NA](http://www.ncbi.nlm.nih.gov/entrez/query.fcgi?cmd=search&db=gene&term=NA) | NA |
| 674 | 0,000215 | 0,00627 | 27,62 | 374,18 | 0,074 | [1445843_at](https://www.affymetrix.com/LinkServlet?probeset=1445843_at) | [Chd2](http://www.ncbi.nlm.nih.gov/entrez/query.fcgi?cmd=search&db=gene&term=Chd2) | chromodomain helicase DNA binding protein 2 |
| 675 | 0,0002159 | 0,00629 | 489,46 | 173,89 | 2,81 | [1443935_at](https://www.affymetrix.com/LinkServlet?probeset=1443935_at) | [BC032203](http://www.ncbi.nlm.nih.gov/entrez/query.fcgi?cmd=search&db=gene&term=BC032203) | cDNA sequence BC032203 |
| 676 | 0,0002171 | 0,00631 | 725,23 | 2091,55 | 0,35 | [1449249_at](https://www.affymetrix.com/LinkServlet?probeset=1449249_at) | [Pcdh7](http://www.ncbi.nlm.nih.gov/entrez/query.fcgi?cmd=search&db=gene&term=Pcdh7) | protocadherin 7 |
| 677 | 0,0002173 | 0,00631 | 774,7 | 303,12 | 2,56 | [1422683_at](https://www.affymetrix.com/LinkServlet?probeset=1422683_at) | [Irak1bp1](http://www.ncbi.nlm.nih.gov/entrez/query.fcgi?cmd=search&db=gene&term=Irak1bp1) | interleukin-1 receptor-associated kinase 1 binding protein 1 |
| 678 | 0,0002185 | 0,00634 | 32,44 | 166,12 | 0,2 | [1417288_at](https://www.affymetrix.com/LinkServlet?probeset=1417288_at) | [Plekha2](http://www.ncbi.nlm.nih.gov/entrez/query.fcgi?cmd=search&db=gene&term=Plekha2) | pleckstrin homology domain-containing, family A (phosphoinositide binding specific) member 2 |
| 679 | 0,0002199 | 0,00636 | 723,61 | 64,16 | 11,28 | [1422853_at](https://www.affymetrix.com/LinkServlet?probeset=1422853_at) | [Shc1](http://www.ncbi.nlm.nih.gov/entrez/query.fcgi?cmd=search&db=gene&term=Shc1) | src homology 2 domain-containing transforming protein C1 |
| 680 | 0,0002201 | 0,00636 | 1418,77 | 794,23 | 1,79 | [1418387_at](https://www.affymetrix.com/LinkServlet?probeset=1418387_at) | [Mphosph8](http://www.ncbi.nlm.nih.gov/entrez/query.fcgi?cmd=search&db=gene&term=Mphosph8) | M-phase phosphoprotein 8 |
| 681 | 0,0002204 | 0,00636 | 421,15 | 73,69 | 5,72 | [1450519_a_at](https://www.affymetrix.com/LinkServlet?probeset=1450519_a_at) | [Prkaca](http://www.ncbi.nlm.nih.gov/entrez/query.fcgi?cmd=search&db=gene&term=Prkaca) | protein kinase, cAMP dependent, catalytic, alpha |
| 682 | 0,0002207 | 0,00636 | 7,62 | 123,57 | 0,062 | [1439972_at](https://www.affymetrix.com/LinkServlet?probeset=1439972_at) | [Etnk1](http://www.ncbi.nlm.nih.gov/entrez/query.fcgi?cmd=search&db=gene&term=Etnk1) | ethanolamine kinase 1 |
| 683 | 0,0002217 | 0,00637 | 8,16 | 169,09 | 0,048 | [1453002_at](https://www.affymetrix.com/LinkServlet?probeset=1453002_at) | [Sox11](http://www.ncbi.nlm.nih.gov/entrez/query.fcgi?cmd=search&db=gene&term=Sox11) | SRY-box containing gene 11 |
| 684 | 0,000222 | 0,00637 | 977,78 | 557,56 | 1,75 | [1452097_a_at](https://www.affymetrix.com/LinkServlet?probeset=1452097_a_at) | [Dusp7](http://www.ncbi.nlm.nih.gov/entrez/query.fcgi?cmd=search&db=gene&term=Dusp7) | dual specificity phosphatase 7 |
| 685 | 0,000222 | 0,00637 | 181,17 | 677,33 | 0,27 | [1424008_a_at](https://www.affymetrix.com/LinkServlet?probeset=1424008_a_at) | [Rbpms2](http://www.ncbi.nlm.nih.gov/entrez/query.fcgi?cmd=search&db=gene&term=Rbpms2) | RNA binding protein with multiple splicing 2 |
| 686 | 0,0002223 | 0,00637 | 1339,9 | 3658,77 | 0,37 | [1456778_at](https://www.affymetrix.com/LinkServlet?probeset=1456778_at) | [NA](http://www.ncbi.nlm.nih.gov/entrez/query.fcgi?cmd=search&db=gene&term=NA) | NA |
| 687 | 0,0002235 | 0,00638 | 82,3 | 808,54 | 0,1 | [1427225_at](https://www.affymetrix.com/LinkServlet?probeset=1427225_at) | [Epn2](http://www.ncbi.nlm.nih.gov/entrez/query.fcgi?cmd=search&db=gene&term=Epn2) | epsin 2 |
| 688 | 0,0002236 | 0,00638 | 6,63 | 229,06 | 0,029 | [1431362_a_at](https://www.affymetrix.com/LinkServlet?probeset=1431362_a_at) | [Smoc2](http://www.ncbi.nlm.nih.gov/entrez/query.fcgi?cmd=search&db=gene&term=Smoc2) | SPARC related modular calcium binding 2 |
| 689 | 0,0002236 | 0,00638 | 34,89 | 238,36 | 0,15 | [1420682_at](https://www.affymetrix.com/LinkServlet?probeset=1420682_at) | [Chrnb1](http://www.ncbi.nlm.nih.gov/entrez/query.fcgi?cmd=search&db=gene&term=Chrnb1) | cholinergic receptor, nicotinic, beta polypeptide 1 (muscle) |
| 690 | 0,0002244 | 0,00639 | 11816,87 | 3668,8 | 3,22 | [1416657_at](https://www.affymetrix.com/LinkServlet?probeset=1416657_at) | [Akt1](http://www.ncbi.nlm.nih.gov/entrez/query.fcgi?cmd=search&db=gene&term=Akt1) | thymoma viral proto-oncogene 1 |
| 691 | 0,0002251 | 0,00639 | 59,2 | 9,36 | 6,32 | [1435481_at](https://www.affymetrix.com/LinkServlet?probeset=1435481_at) | [Zfp653](http://www.ncbi.nlm.nih.gov/entrez/query.fcgi?cmd=search&db=gene&term=Zfp653) | zinc finger protein 653 |
| 692 | 0,0002253 | 0,00639 | 118,27 | 251,42 | 0,47 | [1456520_at](https://www.affymetrix.com/LinkServlet?probeset=1456520_at) | [Zfp652](http://www.ncbi.nlm.nih.gov/entrez/query.fcgi?cmd=search&db=gene&term=Zfp652) | zinc finger protein 652 |
| 693 | 0,0002253 | 0,00639 | 2866,38 | 1262,07 | 2,27 | [1431299_a_at](https://www.affymetrix.com/LinkServlet?probeset=1431299_a_at) | [2310014H01Rik](http://www.ncbi.nlm.nih.gov/entrez/query.fcgi?cmd=search&db=gene&term=2310014H01Rik) | RIKEN cDNA 2310014H01 gene |
| 694 | 0,0002295 | 0,0065 | 10,3 | 34,01 | 0,3 | [1436943_at](https://www.affymetrix.com/LinkServlet?probeset=1436943_at) | [Cyb5d2](http://www.ncbi.nlm.nih.gov/entrez/query.fcgi?cmd=search&db=gene&term=Cyb5d2) | cytochrome b5 domain containing 2 |
| 695 | 0,0002307 | 0,00653 | 16530,34 | 7620,24 | 2,17 | [1460561_x_at](https://www.affymetrix.com/LinkServlet?probeset=1460561_x_at) | [Sepw1](http://www.ncbi.nlm.nih.gov/entrez/query.fcgi?cmd=search&db=gene&term=Sepw1) | selenoprotein W, muscle 1 |
| 696 | 0,0002323 | 0,00656 | 12988,77 | 17585,9 | 0,74 | [1455036_s_at](https://www.affymetrix.com/LinkServlet?probeset=1455036_s_at) | [Ndufc2](http://www.ncbi.nlm.nih.gov/entrez/query.fcgi?cmd=search&db=gene&term=Ndufc2) | NADH dehydrogenase (ubiquinone) 1, subcomplex unknown, 2 |
| 697 | 0,0002324 | 0,00656 | 6,1 | 14,9 | 0,41 | [1421960_at](https://www.affymetrix.com/LinkServlet?probeset=1421960_at) | [Adcy3](http://www.ncbi.nlm.nih.gov/entrez/query.fcgi?cmd=search&db=gene&term=Adcy3) | adenylate cyclase 3 |
| 698 | 0,000233 | 0,00657 | 421,2 | 7,09 | 59,44 | [1422726_x_at](https://www.affymetrix.com/LinkServlet?probeset=1422726_x_at) | [Speer4a](http://www.ncbi.nlm.nih.gov/entrez/query.fcgi?cmd=search&db=gene&term=Speer4a) | spermatogenesis associated glutamate (E)-rich protein 4a |
| 699 | 0,000235 | 0,00661 | 406,56 | 1157,66 | 0,35 | [1457751_at](https://www.affymetrix.com/LinkServlet?probeset=1457751_at) | [Rsf1](http://www.ncbi.nlm.nih.gov/entrez/query.fcgi?cmd=search&db=gene&term=Rsf1) | remodeling and spacing factor 1 |
| 700 | 0,0002354 | 0,00661 | 12,26 | 342,57 | 0,036 | [1425538_x_at](https://www.affymetrix.com/LinkServlet?probeset=1425538_x_at) | [Ceacam1](http://www.ncbi.nlm.nih.gov/entrez/query.fcgi?cmd=search&db=gene&term=Ceacam1) | carcinoembryonic antigen-related cell adhesion molecule 1 |
| 701 | 0,0002356 | 0,00661 | 31,94 | 247,49 | 0,13 | [1452050_at](https://www.affymetrix.com/LinkServlet?probeset=1452050_at) | [Camk1d](http://www.ncbi.nlm.nih.gov/entrez/query.fcgi?cmd=search&db=gene&term=Camk1d) | calcium/calmodulin-dependent protein kinase ID |
| 702 | 0,0002363 | 0,00662 | 7218,93 | 15476,56 | 0,47 | [1423722_at](https://www.affymetrix.com/LinkServlet?probeset=1423722_at) | [Tmem49](http://www.ncbi.nlm.nih.gov/entrez/query.fcgi?cmd=search&db=gene&term=Tmem49) | transmembrane protein 49 |
| 703 | 0,0002365 | 0,00662 | 816,83 | 45,19 | 18,08 | [1454686_at](https://www.affymetrix.com/LinkServlet?probeset=1454686_at) | [NA](http://www.ncbi.nlm.nih.gov/entrez/query.fcgi?cmd=search&db=gene&term=NA) | NA |
| 704 | 0,0002372 | 0,00663 | 4,51 | 108,1 | 0,042 | [1422607_at](https://www.affymetrix.com/LinkServlet?probeset=1422607_at) | [Etv1](http://www.ncbi.nlm.nih.gov/entrez/query.fcgi?cmd=search&db=gene&term=Etv1) | ets variant gene 1 |
| 705 | 0,0002381 | 0,00664 | 453,67 | 679,24 | 0,67 | [1423118_at](https://www.affymetrix.com/LinkServlet?probeset=1423118_at) | [1200014J11Rik](http://www.ncbi.nlm.nih.gov/entrez/query.fcgi?cmd=search&db=gene&term=1200014J11Rik) | RIKEN cDNA 1200014J11 gene |
| 706 | 0,0002385 | 0,00664 | 572,27 | 11,83 | 48,37 | [1421026_at](https://www.affymetrix.com/LinkServlet?probeset=1421026_at) | [Gna12](http://www.ncbi.nlm.nih.gov/entrez/query.fcgi?cmd=search&db=gene&term=Gna12) | guanine nucleotide binding protein, alpha 12 |
| 707 | 0,0002386 | 0,00664 | 5,68 | 37,05 | 0,15 | [1444435_at](https://www.affymetrix.com/LinkServlet?probeset=1444435_at) | [Vps13b](http://www.ncbi.nlm.nih.gov/entrez/query.fcgi?cmd=search&db=gene&term=Vps13b) | vacuolar protein sorting 13B (yeast) |
| 708 | 0,0002396 | 0,00666 | 20,68 | 4,44 | 4,66 | [1432229_a_at](https://www.affymetrix.com/LinkServlet?probeset=1432229_a_at) | [Cdyl2](http://www.ncbi.nlm.nih.gov/entrez/query.fcgi?cmd=search&db=gene&term=Cdyl2) | chromodomain protein, Y chromosome-like 2 |
| 709 | 0,0002404 | 0,00666 | 1715,69 | 169,5 | 10,12 | [1417963_at](https://www.affymetrix.com/LinkServlet?probeset=1417963_at) | [Pltp](http://www.ncbi.nlm.nih.gov/entrez/query.fcgi?cmd=search&db=gene&term=Pltp) | phospholipid transfer protein |
| 710 | 0,0002405 | 0,00666 | 1083,47 | 462,15 | 2,34 | [1419883_s_at](https://www.affymetrix.com/LinkServlet?probeset=1419883_s_at) | [Atp6v1b2](http://www.ncbi.nlm.nih.gov/entrez/query.fcgi?cmd=search&db=gene&term=Atp6v1b2) | ATPase, H+ transporting, lysosomal V1 subunit B2 |
| 711 | 0,0002409 | 0,00666 | 953,48 | 241,05 | 3,96 | [1431188_a_at](https://www.affymetrix.com/LinkServlet?probeset=1431188_a_at) | [Tom1](http://www.ncbi.nlm.nih.gov/entrez/query.fcgi?cmd=search&db=gene&term=Tom1) | target of myb1 homolog (chicken) |
| 712 | 0,0002414 | 0,00667 | 5,93 | 71,58 | 0,083 | [1457701_at](https://www.affymetrix.com/LinkServlet?probeset=1457701_at) | [Tmem136](http://www.ncbi.nlm.nih.gov/entrez/query.fcgi?cmd=search&db=gene&term=Tmem136) | transmembrane protein 136 |
| 713 | 0,0002439 | 0,00673 | 812,82 | 268,56 | 3,03 | [1426492_at](https://www.affymetrix.com/LinkServlet?probeset=1426492_at) | [Tdp1](http://www.ncbi.nlm.nih.gov/entrez/query.fcgi?cmd=search&db=gene&term=Tdp1) | tyrosyl-DNA phosphodiesterase 1 |
| 714 | 0,0002442 | 0,00673 | 82,12 | 5,07 | 16,21 | [1422520_at](https://www.affymetrix.com/LinkServlet?probeset=1422520_at) | [Nefm](http://www.ncbi.nlm.nih.gov/entrez/query.fcgi?cmd=search&db=gene&term=Nefm) | neurofilament, medium polypeptide |
| 715 | 0,0002445 | 0,00673 | 1254,89 | 282,49 | 4,44 | [1419640_at](https://www.affymetrix.com/LinkServlet?probeset=1419640_at) | [Purb](http://www.ncbi.nlm.nih.gov/entrez/query.fcgi?cmd=search&db=gene&term=Purb) | purine rich element binding protein B |
| 716 | 0,0002452 | 0,00674 | 916,86 | 204,33 | 4,49 | [1434361_at](https://www.affymetrix.com/LinkServlet?probeset=1434361_at) | [Snx33](http://www.ncbi.nlm.nih.gov/entrez/query.fcgi?cmd=search&db=gene&term=Snx33) | sorting nexin 33 |
| 717 | 0,0002457 | 0,00674 | 1500,19 | 559,51 | 2,68 | [1418888_a_at](https://www.affymetrix.com/LinkServlet?probeset=1418888_a_at) | [Sepx1](http://www.ncbi.nlm.nih.gov/entrez/query.fcgi?cmd=search&db=gene&term=Sepx1) | selenoprotein X 1 |
| 718 | 0,0002466 | 0,00675 | 1313,94 | 575,42 | 2,28 | [1434318_a_at](https://www.affymetrix.com/LinkServlet?probeset=1434318_a_at) | [Tcfe3](http://www.ncbi.nlm.nih.gov/entrez/query.fcgi?cmd=search&db=gene&term=Tcfe3) | transcription factor E3 |
| 719 | 0,0002469 | 0,00675 | 3227,17 | 822,69 | 3,92 | [1428065_at](https://www.affymetrix.com/LinkServlet?probeset=1428065_at) | [Slc44a2](http://www.ncbi.nlm.nih.gov/entrez/query.fcgi?cmd=search&db=gene&term=Slc44a2) | solute carrier family 44, member 2 |
| 720 | 0,0002479 | 0,00677 | 835,41 | 429,29 | 1,95 | [1427132_at](https://www.affymetrix.com/LinkServlet?probeset=1427132_at) | [Sbf2](http://www.ncbi.nlm.nih.gov/entrez/query.fcgi?cmd=search&db=gene&term=Sbf2) | SET binding factor 2 |
| 721 | 0,0002492 | 0,0068 | 217,32 | 629,04 | 0,35 | [1457266_at](https://www.affymetrix.com/LinkServlet?probeset=1457266_at) | [NA](http://www.ncbi.nlm.nih.gov/entrez/query.fcgi?cmd=search&db=gene&term=NA) | NA |
| 722 | 0,0002497 | 0,0068 | 18118,4 | 3363,46 | 5,39 | [1448732_at](https://www.affymetrix.com/LinkServlet?probeset=1448732_at) | [Ctsb](http://www.ncbi.nlm.nih.gov/entrez/query.fcgi?cmd=search&db=gene&term=Ctsb) | cathepsin B |
| 723 | 0,0002509 | 0,00681 | 7,87 | 416,84 | 0,019 | [1417262_at](https://www.affymetrix.com/LinkServlet?probeset=1417262_at) | [Ptgs2](http://www.ncbi.nlm.nih.gov/entrez/query.fcgi?cmd=search&db=gene&term=Ptgs2) | prostaglandin-endoperoxide synthase 2 |
| 724 | 0,000251 | 0,00681 | 6,31 | 33,85 | 0,19 | [1440729_at](https://www.affymetrix.com/LinkServlet?probeset=1440729_at) | [Eps15](http://www.ncbi.nlm.nih.gov/entrez/query.fcgi?cmd=search&db=gene&term=Eps15) | epidermal growth factor receptor pathway substrate 15 |
| 725 | 0,0002511 | 0,00681 | 12,69 | 44,96 | 0,28 | [1433465_a_at](https://www.affymetrix.com/LinkServlet?probeset=1433465_a_at) | [AI467606](http://www.ncbi.nlm.nih.gov/entrez/query.fcgi?cmd=search&db=gene&term=AI467606) | expressed sequence AI467606 |
| 726 | 0,0002521 | 0,00681 | 4232,17 | 7073,6 | 0,6 | [1423211_at](https://www.affymetrix.com/LinkServlet?probeset=1423211_at) | [Nop10](http://www.ncbi.nlm.nih.gov/entrez/query.fcgi?cmd=search&db=gene&term=Nop10) | NOP10 ribonucleoprotein homolog (yeast) |
| 727 | 0,0002525 | 0,00681 | 124,37 | 919,86 | 0,14 | [1426785_s_at](https://www.affymetrix.com/LinkServlet?probeset=1426785_s_at) | [Mgll](http://www.ncbi.nlm.nih.gov/entrez/query.fcgi?cmd=search&db=gene&term=Mgll) | monoglyceride lipase |
| 728 | 0,0002527 | 0,00681 | 923,79 | 284,94 | 3,24 | [1452685_at](https://www.affymetrix.com/LinkServlet?probeset=1452685_at) | [0610009O20Rik](http://www.ncbi.nlm.nih.gov/entrez/query.fcgi?cmd=search&db=gene&term=0610009O20Rik) | RIKEN cDNA 0610009O20 gene |
| 729 | 0,0002532 | 0,00681 | 798,6 | 1994,22 | 0,4 | [1417045_at](https://www.affymetrix.com/LinkServlet?probeset=1417045_at) | [Bid](http://www.ncbi.nlm.nih.gov/entrez/query.fcgi?cmd=search&db=gene&term=Bid) | BH3 interacting domain death agonist |
| 730 | 0,0002535 | 0,00681 | 77,87 | 523,73 | 0,15 | [1436978_at](https://www.affymetrix.com/LinkServlet?probeset=1436978_at) | [Wnt9a](http://www.ncbi.nlm.nih.gov/entrez/query.fcgi?cmd=search&db=gene&term=Wnt9a) | wingless-type MMTV integration site 9A |
| 731 | 0,0002535 | 0,00681 | 605,29 | 152,62 | 3,97 | [1433932_x_at](https://www.affymetrix.com/LinkServlet?probeset=1433932_x_at) | [C030046I01Rik](http://www.ncbi.nlm.nih.gov/entrez/query.fcgi?cmd=search&db=gene&term=C030046I01Rik) | RIKEN cDNA C030046I01 gene |
| 732 | 0,0002537 | 0,00681 | 2987,68 | 746,28 | 4 | [1416933_at](https://www.affymetrix.com/LinkServlet?probeset=1416933_at) | [Por](http://www.ncbi.nlm.nih.gov/entrez/query.fcgi?cmd=search&db=gene&term=Por) | P450 (cytochrome) oxidoreductase |
| 733 | 0,0002538 | 0,00681 | 4,77 | 13,54 | 0,35 | [1422955_at](https://www.affymetrix.com/LinkServlet?probeset=1422955_at) | [Syt17](http://www.ncbi.nlm.nih.gov/entrez/query.fcgi?cmd=search&db=gene&term=Syt17) | synaptotagmin XVII |
| 734 | 0,0002541 | 0,00681 | 4598 | 1195,01 | 3,85 | [1450976_at](https://www.affymetrix.com/LinkServlet?probeset=1450976_at) | [Ndrg1](http://www.ncbi.nlm.nih.gov/entrez/query.fcgi?cmd=search&db=gene&term=Ndrg1) | N-myc downstream regulated gene 1 |
| 735 | 0,0002557 | 0,00684 | 3121,88 | 303,56 | 10,28 | [1416572_at](https://www.affymetrix.com/LinkServlet?probeset=1416572_at) | [Mmp14](http://www.ncbi.nlm.nih.gov/entrez/query.fcgi?cmd=search&db=gene&term=Mmp14) | matrix metallopeptidase 14 (membrane-inserted) |
| 736 | 0,000256 | 0,00684 | 1273,81 | 129,44 | 9,84 | [1427165_at](https://www.affymetrix.com/LinkServlet?probeset=1427165_at) | [Il13ra1](http://www.ncbi.nlm.nih.gov/entrez/query.fcgi?cmd=search&db=gene&term=Il13ra1) | interleukin 13 receptor, alpha 1 |
| 737 | 0,0002563 | 0,00684 | 1048,16 | 621,42 | 1,69 | [1435093_at](https://www.affymetrix.com/LinkServlet?probeset=1435093_at) | [Zfyve20](http://www.ncbi.nlm.nih.gov/entrez/query.fcgi?cmd=search&db=gene&term=Zfyve20) | zinc finger, FYVE domain containing 20 |
| 738 | 0,0002575 | 0,00686 | 1477,92 | 2331,36 | 0,63 | [1424184_at](https://www.affymetrix.com/LinkServlet?probeset=1424184_at) | [Acadvl](http://www.ncbi.nlm.nih.gov/entrez/query.fcgi?cmd=search&db=gene&term=Acadvl) | acyl-Coenzyme A dehydrogenase, very long chain |
| 739 | 0,0002601 | 0,00692 | 145,15 | 442,52 | 0,33 | [1417279_at](https://www.affymetrix.com/LinkServlet?probeset=1417279_at) | [Itpr1](http://www.ncbi.nlm.nih.gov/entrez/query.fcgi?cmd=search&db=gene&term=Itpr1) | inositol 1,4,5-triphosphate receptor 1 |
| 740 | 0,0002604 | 0,00692 | 325,03 | 78,57 | 4,14 | [1434455_at](https://www.affymetrix.com/LinkServlet?probeset=1434455_at) | [Fbxo44](http://www.ncbi.nlm.nih.gov/entrez/query.fcgi?cmd=search&db=gene&term=Fbxo44) | F-box protein 44 |
| 741 | 0,0002607 | 0,00692 | 3019,3 | 872,6 | 3,46 | [1429290_at](https://www.affymetrix.com/LinkServlet?probeset=1429290_at) | [NA](http://www.ncbi.nlm.nih.gov/entrez/query.fcgi?cmd=search&db=gene&term=NA) | NA |
| 742 | 0,0002612 | 0,00692 | 3147,66 | 8256,48 | 0,38 | [1415964_at](https://www.affymetrix.com/LinkServlet?probeset=1415964_at) | [Scd1](http://www.ncbi.nlm.nih.gov/entrez/query.fcgi?cmd=search&db=gene&term=Scd1) | stearoyl-Coenzyme A desaturase 1 |
| 743 | 0,0002624 | 0,00695 | 3856,48 | 1291,69 | 2,99 | [1433890_a_at](https://www.affymetrix.com/LinkServlet?probeset=1433890_a_at) | [Bag6](http://www.ncbi.nlm.nih.gov/entrez/query.fcgi?cmd=search&db=gene&term=Bag6) | BCL2-associated athanogene 6 |
| 744 | 0,0002633 | 0,00695 | 324,81 | 5382,28 | 0,06 | [1450035_a_at](https://www.affymetrix.com/LinkServlet?probeset=1450035_a_at) | [Prpf40a](http://www.ncbi.nlm.nih.gov/entrez/query.fcgi?cmd=search&db=gene&term=Prpf40a) | PRP40 pre-mRNA processing factor 40 homolog A (yeast) |
| 745 | 0,0002644 | 0,00695 | 2845,1 | 894,2 | 3,18 | [1423693_at](https://www.affymetrix.com/LinkServlet?probeset=1423693_at) | [Cela1](http://www.ncbi.nlm.nih.gov/entrez/query.fcgi?cmd=search&db=gene&term=Cela1) | chymotrypsin-like elastase family, member 1 |
| 746 | 0,0002646 | 0,00695 | 26,15 | 1400,4 | 0,019 | [1415935_at](https://www.affymetrix.com/LinkServlet?probeset=1415935_at) | [Smoc2](http://www.ncbi.nlm.nih.gov/entrez/query.fcgi?cmd=search&db=gene&term=Smoc2) | SPARC related modular calcium binding 2 |
| 747 | 0,0002646 | 0,00695 | 9,02 | 213,47 | 0,042 | [1418603_at](https://www.affymetrix.com/LinkServlet?probeset=1418603_at) | [Avpr1a](http://www.ncbi.nlm.nih.gov/entrez/query.fcgi?cmd=search&db=gene&term=Avpr1a) | arginine vasopressin receptor 1A |
| 748 | 0,0002646 | 0,00695 | 1664,44 | 883,68 | 1,88 | [1448877_at](https://www.affymetrix.com/LinkServlet?probeset=1448877_at) | [Dlx2](http://www.ncbi.nlm.nih.gov/entrez/query.fcgi?cmd=search&db=gene&term=Dlx2) | distal-less homeobox 2 |
| 749 | 0,0002649 | 0,00695 | 8857,81 | 2405,12 | 3,68 | [1448415_a_at](https://www.affymetrix.com/LinkServlet?probeset=1448415_a_at) | [Sema3b](http://www.ncbi.nlm.nih.gov/entrez/query.fcgi?cmd=search&db=gene&term=Sema3b) | sema domain, immunoglobulin domain (Ig), short basic domain, secreted, (semaphorin) 3B |
| 750 | 0,000265 | 0,00695 | 1602,06 | 4505,13 | 0,36 | [1418083_at](https://www.affymetrix.com/LinkServlet?probeset=1418083_at) | [0610009B22Rik](http://www.ncbi.nlm.nih.gov/entrez/query.fcgi?cmd=search&db=gene&term=0610009B22Rik) | RIKEN cDNA 0610009B22 gene |
| 751 | 0,0002659 | 0,00695 | 408,89 | 132,37 | 3,09 | [1428425_at](https://www.affymetrix.com/LinkServlet?probeset=1428425_at) | [Tgfbrap1](http://www.ncbi.nlm.nih.gov/entrez/query.fcgi?cmd=search&db=gene&term=Tgfbrap1) | transforming growth factor, beta receptor associated protein 1 |
| 752 | 0,000266 | 0,00695 | 1497,67 | 372,09 | 4,03 | [1415978_at](https://www.affymetrix.com/LinkServlet?probeset=1415978_at) | [Tubb3](http://www.ncbi.nlm.nih.gov/entrez/query.fcgi?cmd=search&db=gene&term=Tubb3) | tubulin, beta 3 |
| 753 | 0,0002661 | 0,00695 | 315,44 | 149,37 | 2,11 | [1428202_at](https://www.affymetrix.com/LinkServlet?probeset=1428202_at) | [Fam3a](http://www.ncbi.nlm.nih.gov/entrez/query.fcgi?cmd=search&db=gene&term=Fam3a) | family with sequence similarity 3, member A |
| 754 | 0,0002676 | 0,00695 | 8,35 | 823,59 | 0,01 | [1437385_at](https://www.affymetrix.com/LinkServlet?probeset=1437385_at) | [Ccbe1](http://www.ncbi.nlm.nih.gov/entrez/query.fcgi?cmd=search&db=gene&term=Ccbe1) | collagen and calcium binding EGF domains 1 |
| 755 | 0,0002677 | 0,00695 | 250,97 | 66,31 | 3,78 | [1418982_at](https://www.affymetrix.com/LinkServlet?probeset=1418982_at) | [Cebpa](http://www.ncbi.nlm.nih.gov/entrez/query.fcgi?cmd=search&db=gene&term=Cebpa) | CCAAT/enhancer binding protein (C/EBP), alpha |
| 756 | 0,0002679 | 0,00695 | 367,53 | 30,49 | 12,06 | [1416388_at](https://www.affymetrix.com/LinkServlet?probeset=1416388_at) | [Pip4k2c](http://www.ncbi.nlm.nih.gov/entrez/query.fcgi?cmd=search&db=gene&term=Pip4k2c) | phosphatidylinositol-5-phosphate 4-kinase, type II, gamma |
| 757 | 0,0002679 | 0,00695 | 31,78 | 68,72 | 0,46 | [1436404_at](https://www.affymetrix.com/LinkServlet?probeset=1436404_at) | [Tlcd1](http://www.ncbi.nlm.nih.gov/entrez/query.fcgi?cmd=search&db=gene&term=Tlcd1) | TLC domain containing 1 |
| 758 | 0,0002681 | 0,00695 | 248,15 | 43,08 | 5,76 | [1423984_a_at](https://www.affymetrix.com/LinkServlet?probeset=1423984_a_at) | [Meis3](http://www.ncbi.nlm.nih.gov/entrez/query.fcgi?cmd=search&db=gene&term=Meis3) | Meis homeobox 3 |
| 759 | 0,0002681 | 0,00695 | 2469,61 | 1242,88 | 1,99 | [1437448_s_at](https://www.affymetrix.com/LinkServlet?probeset=1437448_s_at) | [Ctnnd1](http://www.ncbi.nlm.nih.gov/entrez/query.fcgi?cmd=search&db=gene&term=Ctnnd1) | catenin (cadherin associated protein), delta 1 |
| 760 | 0,0002687 | 0,00695 | 2247,2 | 3923 | 0,57 | [1450866_a_at](https://www.affymetrix.com/LinkServlet?probeset=1450866_a_at) | [Mrpl17](http://www.ncbi.nlm.nih.gov/entrez/query.fcgi?cmd=search&db=gene&term=Mrpl17) | mitochondrial ribosomal protein L17 |
| 761 | 0,0002693 | 0,00696 | 4786,08 | 3523,16 | 1,36 | [1448422_at](https://www.affymetrix.com/LinkServlet?probeset=1448422_at) | [Tmed4](http://www.ncbi.nlm.nih.gov/entrez/query.fcgi?cmd=search&db=gene&term=Tmed4) | transmembrane emp24 protein transport domain containing 4 |
| 762 | 0,0002698 | 0,00696 | 8,07 | 152,19 | 0,053 | [1428789_at](https://www.affymetrix.com/LinkServlet?probeset=1428789_at) | [Ralgps2](http://www.ncbi.nlm.nih.gov/entrez/query.fcgi?cmd=search&db=gene&term=Ralgps2) | Ral GEF with PH domain and SH3 binding motif 2 |
| 763 | 0,0002712 | 0,00699 | 77 | 5,21 | 14,77 | [1453222_at](https://www.affymetrix.com/LinkServlet?probeset=1453222_at) | [NA](http://www.ncbi.nlm.nih.gov/entrez/query.fcgi?cmd=search&db=gene&term=NA) | NA |
| 764 | 0,000272 | 0,00699 | 8708,9 | 12282,97 | 0,71 | [1429227_x_at](https://www.affymetrix.com/LinkServlet?probeset=1429227_x_at) | [Nap1l1](http://www.ncbi.nlm.nih.gov/entrez/query.fcgi?cmd=search&db=gene&term=Nap1l1) | nucleosome assembly protein 1-like 1 |
| 765 | 0,0002721 | 0,00699 | 834,09 | 129,72 | 6,43 | [1460634_at](https://www.affymetrix.com/LinkServlet?probeset=1460634_at) | [Ralgds](http://www.ncbi.nlm.nih.gov/entrez/query.fcgi?cmd=search&db=gene&term=Ralgds) | ral guanine nucleotide dissociation stimulator |
| 766 | 0,0002726 | 0,00699 | 2848,91 | 1137,11 | 2,51 | [1434044_at](https://www.affymetrix.com/LinkServlet?probeset=1434044_at) | [Repin1](http://www.ncbi.nlm.nih.gov/entrez/query.fcgi?cmd=search&db=gene&term=Repin1) | replication initiator 1 |
| 767 | 0,0002728 | 0,00699 | 8047,2 | 991,66 | 8,11 | [1448346_at](https://www.affymetrix.com/LinkServlet?probeset=1448346_at) | [Cfl1](http://www.ncbi.nlm.nih.gov/entrez/query.fcgi?cmd=search&db=gene&term=Cfl1) | cofilin 1, non-muscle |
| 768 | 0,000273 | 0,00699 | 7,03 | 233,37 | 0,03 | [1417292_at](https://www.affymetrix.com/LinkServlet?probeset=1417292_at) | [Ifi47](http://www.ncbi.nlm.nih.gov/entrez/query.fcgi?cmd=search&db=gene&term=Ifi47) | interferon gamma inducible protein 47 |
| 769 | 0,0002737 | 0,007 | 3382,12 | 9715,15 | 0,35 | [1426964_at](https://www.affymetrix.com/LinkServlet?probeset=1426964_at) | [3110003A17Rik](http://www.ncbi.nlm.nih.gov/entrez/query.fcgi?cmd=search&db=gene&term=3110003A17Rik) | RIKEN cDNA 3110003A17 gene |
| 770 | 0,000274 | 0,007 | 3058,92 | 1455,26 | 2,1 | [1419155_a_at](https://www.affymetrix.com/LinkServlet?probeset=1419155_a_at) | [Sox4](http://www.ncbi.nlm.nih.gov/entrez/query.fcgi?cmd=search&db=gene&term=Sox4) | SRY-box containing gene 4 |
| 771 | 0,0002759 | 0,00704 | 25,36 | 352,54 | 0,072 | [1448468_a_at](https://www.affymetrix.com/LinkServlet?probeset=1448468_a_at) | [Kcnab1](http://www.ncbi.nlm.nih.gov/entrez/query.fcgi?cmd=search&db=gene&term=Kcnab1) | potassium voltage-gated channel, shaker-related subfamily, beta member 1 |
| 772 | 0,0002764 | 0,00704 | 911,82 | 1232,13 | 0,74 | [1428413_at](https://www.affymetrix.com/LinkServlet?probeset=1428413_at) | [Ccny](http://www.ncbi.nlm.nih.gov/entrez/query.fcgi?cmd=search&db=gene&term=Ccny) | cyclin Y |
| 773 | 0,0002768 | 0,00704 | 4,86 | 17,76 | 0,27 | [1446612_at](https://www.affymetrix.com/LinkServlet?probeset=1446612_at) | [9330118A15Rik](http://www.ncbi.nlm.nih.gov/entrez/query.fcgi?cmd=search&db=gene&term=9330118A15Rik) | RIKEN cDNA 9330118A15 gene |
| 774 | 0,0002775 | 0,00705 | 2506,07 | 3946,98 | 0,63 | [1423106_at](https://www.affymetrix.com/LinkServlet?probeset=1423106_at) | [Ube2b](http://www.ncbi.nlm.nih.gov/entrez/query.fcgi?cmd=search&db=gene&term=Ube2b) | ubiquitin-conjugating enzyme E2B, RAD6 homology (S. cerevisiae) |
| 775 | 0,0002785 | 0,00707 | 92,28 | 765,81 | 0,12 | [1452639_at](https://www.affymetrix.com/LinkServlet?probeset=1452639_at) | [Enpp4](http://www.ncbi.nlm.nih.gov/entrez/query.fcgi?cmd=search&db=gene&term=Enpp4) | ectonucleotide pyrophosphatase/phosphodiesterase 4 |
| 776 | 0,0002794 | 0,00708 | 2168,62 | 838,28 | 2,59 | [1450927_at](https://www.affymetrix.com/LinkServlet?probeset=1450927_at) | [Lztr1](http://www.ncbi.nlm.nih.gov/entrez/query.fcgi?cmd=search&db=gene&term=Lztr1) | leucine-zipper-like transcriptional regulator, 1 |
| 777 | 0,0002797 | 0,00708 | 7235,52 | 16906,27 | 0,43 | [1417379_at](https://www.affymetrix.com/LinkServlet?probeset=1417379_at) | [Iqgap1](http://www.ncbi.nlm.nih.gov/entrez/query.fcgi?cmd=search&db=gene&term=Iqgap1) | IQ motif containing GTPase activating protein 1 |
| 778 | 0,00028 | 0,00708 | 186,28 | 652,96 | 0,29 | [1423840_at](https://www.affymetrix.com/LinkServlet?probeset=1423840_at) | [Ccdc56](http://www.ncbi.nlm.nih.gov/entrez/query.fcgi?cmd=search&db=gene&term=Ccdc56) | coiled-coil domain containing 56 |
| 779 | 0,0002811 | 0,00708 | 149,56 | 11,49 | 13,02 | [1436414_at](https://www.affymetrix.com/LinkServlet?probeset=1436414_at) | [Obsl1](http://www.ncbi.nlm.nih.gov/entrez/query.fcgi?cmd=search&db=gene&term=Obsl1) | obscurin-like 1 |
| 780 | 0,0002812 | 0,00708 | 5,11 | 184,04 | 0,028 | [1436221_at](https://www.affymetrix.com/LinkServlet?probeset=1436221_at) | [Ildr2](http://www.ncbi.nlm.nih.gov/entrez/query.fcgi?cmd=search&db=gene&term=Ildr2) | immunoglobulin-like domain containing receptor 2 |
| 781 | 0,0002812 | 0,00708 | 615,39 | 2380,95 | 0,26 | [1417323_at](https://www.affymetrix.com/LinkServlet?probeset=1417323_at) | [Psrc1](http://www.ncbi.nlm.nih.gov/entrez/query.fcgi?cmd=search&db=gene&term=Psrc1) | proline/serine-rich coiled-coil 1 |
| 782 | 0,000282 | 0,00709 | 2356,07 | 6873,62 | 0,34 | [1417659_at](https://www.affymetrix.com/LinkServlet?probeset=1417659_at) | [Vps29](http://www.ncbi.nlm.nih.gov/entrez/query.fcgi?cmd=search&db=gene&term=Vps29) | vacuolar protein sorting 29 (S. pombe) |
| 783 | 0,0002825 | 0,0071 | 256,55 | 756,18 | 0,34 | [1424350_s_at](https://www.affymetrix.com/LinkServlet?probeset=1424350_s_at) | [Lpgat1](http://www.ncbi.nlm.nih.gov/entrez/query.fcgi?cmd=search&db=gene&term=Lpgat1) | lysophosphatidylglycerol acyltransferase 1 |
| 784 | 0,0002844 | 0,00713 | 4104,69 | 2042,86 | 2,01 | [1428270_at](https://www.affymetrix.com/LinkServlet?probeset=1428270_at) | [Glt8d1](http://www.ncbi.nlm.nih.gov/entrez/query.fcgi?cmd=search&db=gene&term=Glt8d1) | glycosyltransferase 8 domain containing 1 |
| 785 | 0,0002844 | 0,00713 | 1570,77 | 161,23 | 9,74 | [1415956_a_at](https://www.affymetrix.com/LinkServlet?probeset=1415956_a_at) | [Cdk16](http://www.ncbi.nlm.nih.gov/entrez/query.fcgi?cmd=search&db=gene&term=Cdk16) | cyclin-dependent kinase 16 |
| 786 | 0,0002853 | 0,00714 | 2514,79 | 3552,65 | 0,71 | [1460401_at](https://www.affymetrix.com/LinkServlet?probeset=1460401_at) | [Edem3](http://www.ncbi.nlm.nih.gov/entrez/query.fcgi?cmd=search&db=gene&term=Edem3) | ER degradation enhancer, mannosidase alpha-like 3 |
| 787 | 0,000287 | 0,00717 | 1398,66 | 535,69 | 2,61 | [1449066_a_at](https://www.affymetrix.com/LinkServlet?probeset=1449066_a_at) | [Arhgef7](http://www.ncbi.nlm.nih.gov/entrez/query.fcgi?cmd=search&db=gene&term=Arhgef7) | Rho guanine nucleotide exchange factor (GEF7) |
| 788 | 0,0002877 | 0,00718 | 2947,37 | 5769,31 | 0,51 | [1439452_x_at](https://www.affymetrix.com/LinkServlet?probeset=1439452_x_at) | [Dnpep](http://www.ncbi.nlm.nih.gov/entrez/query.fcgi?cmd=search&db=gene&term=Dnpep) | aspartyl aminopeptidase |
| 789 | 0,0002889 | 0,0072 | 2862,8 | 1716,55 | 1,67 | [1427027_a_at](https://www.affymetrix.com/LinkServlet?probeset=1427027_a_at) | [Gtf3a](http://www.ncbi.nlm.nih.gov/entrez/query.fcgi?cmd=search&db=gene&term=Gtf3a) | general transcription factor III A |
| 790 | 0,0002893 | 0,0072 | 443,11 | 3400,55 | 0,13 | [1456603_at](https://www.affymetrix.com/LinkServlet?probeset=1456603_at) | [Fam101b](http://www.ncbi.nlm.nih.gov/entrez/query.fcgi?cmd=search&db=gene&term=Fam101b) | family with sequence similarity 101, member B |
| 791 | 0,0002894 | 0,0072 | 6,93 | 322,05 | 0,022 | [1419692_a_at](https://www.affymetrix.com/LinkServlet?probeset=1419692_a_at) | [Ltc4s](http://www.ncbi.nlm.nih.gov/entrez/query.fcgi?cmd=search&db=gene&term=Ltc4s) | leukotriene C4 synthase |
| 792 | 0,0002899 | 0,0072 | 263,88 | 30,85 | 8,55 | [1454222_a_at](https://www.affymetrix.com/LinkServlet?probeset=1454222_a_at) | [Ccdc13](http://www.ncbi.nlm.nih.gov/entrez/query.fcgi?cmd=search&db=gene&term=Ccdc13) | coiled-coil domain containing 13 |
| 793 | 0,0002918 | 0,00724 | 5052,03 | 7825,12 | 0,65 | [1449187_at](https://www.affymetrix.com/LinkServlet?probeset=1449187_at) | [Pdgfa](http://www.ncbi.nlm.nih.gov/entrez/query.fcgi?cmd=search&db=gene&term=Pdgfa) | platelet derived growth factor, alpha |
| 794 | 0,0002927 | 0,00724 | 4236,05 | 824,06 | 5,14 | [1426947_x_at](https://www.affymetrix.com/LinkServlet?probeset=1426947_x_at) | [Col6a2](http://www.ncbi.nlm.nih.gov/entrez/query.fcgi?cmd=search&db=gene&term=Col6a2) | collagen, type VI, alpha 2 |
| 795 | 0,0002928 | 0,00724 | 453,49 | 1814,08 | 0,25 | [1424711_at](https://www.affymetrix.com/LinkServlet?probeset=1424711_at) | [Tmem2](http://www.ncbi.nlm.nih.gov/entrez/query.fcgi?cmd=search&db=gene&term=Tmem2) | transmembrane protein 2 |
| 796 | 0,0002934 | 0,00724 | 399,33 | 122,29 | 3,27 | [1455592_at](https://www.affymetrix.com/LinkServlet?probeset=1455592_at) | [Vangl2](http://www.ncbi.nlm.nih.gov/entrez/query.fcgi?cmd=search&db=gene&term=Vangl2) | vang-like 2 (van gogh, Drosophila) |
| 797 | 0,0002936 | 0,00724 | 1076,54 | 1840,31 | 0,58 | [1452908_at](https://www.affymetrix.com/LinkServlet?probeset=1452908_at) | [Dip2a](http://www.ncbi.nlm.nih.gov/entrez/query.fcgi?cmd=search&db=gene&term=Dip2a) | DIP2 disco-interacting protein 2 homolog A (Drosophila) |
| 798 | 0,0002965 | 0,0073 | 188,8 | 603,96 | 0,31 | [1435633_at](https://www.affymetrix.com/LinkServlet?probeset=1435633_at) | [Pwwp2a](http://www.ncbi.nlm.nih.gov/entrez/query.fcgi?cmd=search&db=gene&term=Pwwp2a) | PWWP domain containing 2A |
| 799 | 0,0002965 | 0,0073 | 9097,84 | 5723,61 | 1,59 | [1448221_at](https://www.affymetrix.com/LinkServlet?probeset=1448221_at) | [Ddx39b](http://www.ncbi.nlm.nih.gov/entrez/query.fcgi?cmd=search&db=gene&term=Ddx39b) | DEAD (Asp-Glu-Ala-Asp) box polypeptide 39B |
| 800 | 0,0002991 | 0,00735 | 5,91 | 271,49 | 0,022 | [1434203_at](https://www.affymetrix.com/LinkServlet?probeset=1434203_at) | [Fam107a](http://www.ncbi.nlm.nih.gov/entrez/query.fcgi?cmd=search&db=gene&term=Fam107a) | family with sequence similarity 107, member A |
| 801 | 0,0003008 | 0,00738 | 4340,08 | 460,85 | 9,42 | [1425711_a_at](https://www.affymetrix.com/LinkServlet?probeset=1425711_a_at) | [Akt1](http://www.ncbi.nlm.nih.gov/entrez/query.fcgi?cmd=search&db=gene&term=Akt1) | thymoma viral proto-oncogene 1 |
| 802 | 0,0003013 | 0,00738 | 193,77 | 55,77 | 3,47 | [1418313_at](https://www.affymetrix.com/LinkServlet?probeset=1418313_at) | [Zfp276](http://www.ncbi.nlm.nih.gov/entrez/query.fcgi?cmd=search&db=gene&term=Zfp276) | zinc finger protein (C2H2 type) 276 |
| 803 | 0,0003014 | 0,00738 | 2638,62 | 3474,4 | 0,76 | [1419252_at](https://www.affymetrix.com/LinkServlet?probeset=1419252_at) | [Eps15](http://www.ncbi.nlm.nih.gov/entrez/query.fcgi?cmd=search&db=gene&term=Eps15) | epidermal growth factor receptor pathway substrate 15 |
| 804 | 0,0003021 | 0,00739 | 20738,66 | 9054,34 | 2,29 | [1416455_a_at](https://www.affymetrix.com/LinkServlet?probeset=1416455_a_at) | [Cryab](http://www.ncbi.nlm.nih.gov/entrez/query.fcgi?cmd=search&db=gene&term=Cryab) | crystallin, alpha B |
| 805 | 0,0003038 | 0,00742 | 2033,08 | 137,14 | 14,82 | [1448755_at](https://www.affymetrix.com/LinkServlet?probeset=1448755_at) | [Col15a1](http://www.ncbi.nlm.nih.gov/entrez/query.fcgi?cmd=search&db=gene&term=Col15a1) | collagen, type XV, alpha 1 |
| 806 | 0,0003044 | 0,00743 | 476,57 | 861,36 | 0,55 | [1428683_at](https://www.affymetrix.com/LinkServlet?probeset=1428683_at) | [Fam173b](http://www.ncbi.nlm.nih.gov/entrez/query.fcgi?cmd=search&db=gene&term=Fam173b) | family with sequence similarity 173, member B |
| 807 | 0,0003057 | 0,00745 | 1196,46 | 1730,06 | 0,69 | [1434373_at](https://www.affymetrix.com/LinkServlet?probeset=1434373_at) | [Fam168a](http://www.ncbi.nlm.nih.gov/entrez/query.fcgi?cmd=search&db=gene&term=Fam168a) | family with sequence similarity 168, member A |
| 808 | 0,0003061 | 0,00745 | 265,93 | 1778,32 | 0,15 | [1428562_at](https://www.affymetrix.com/LinkServlet?probeset=1428562_at) | [2210403K04Rik](http://www.ncbi.nlm.nih.gov/entrez/query.fcgi?cmd=search&db=gene&term=2210403K04Rik) | RIKEN cDNA 2210403K04 gene |
| 809 | 0,0003066 | 0,00745 | 6193,18 | 3573,34 | 1,73 | [1452712_at](https://www.affymetrix.com/LinkServlet?probeset=1452712_at) | [Hnrnpa3](http://www.ncbi.nlm.nih.gov/entrez/query.fcgi?cmd=search&db=gene&term=Hnrnpa3) | heterogeneous nuclear ribonucleoprotein A3 |
| 810 | 0,0003072 | 0,00745 | 833,53 | 312,2 | 2,67 | [1449406_at](https://www.affymetrix.com/LinkServlet?probeset=1449406_at) | [Cyhr1](http://www.ncbi.nlm.nih.gov/entrez/query.fcgi?cmd=search&db=gene&term=Cyhr1) | cysteine and histidine rich 1 |
| 811 | 0,0003078 | 0,00745 | 51,58 | 250,73 | 0,21 | [1426316_at](https://www.affymetrix.com/LinkServlet?probeset=1426316_at) | [6330416G13Rik](http://www.ncbi.nlm.nih.gov/entrez/query.fcgi?cmd=search&db=gene&term=6330416G13Rik) | RIKEN cDNA 6330416G13 gene |
| 812 | 0,0003078 | 0,00745 | 5,12 | 184,92 | 0,028 | [1421225_a_at](https://www.affymetrix.com/LinkServlet?probeset=1421225_a_at) | [Slc4a4](http://www.ncbi.nlm.nih.gov/entrez/query.fcgi?cmd=search&db=gene&term=Slc4a4) | solute carrier family 4 (anion exchanger), member 4 |
| 813 | 0,0003081 | 0,00745 | 17,23 | 855,25 | 0,02 | [1449033_at](https://www.affymetrix.com/LinkServlet?probeset=1449033_at) | [Tnfrsf11b](http://www.ncbi.nlm.nih.gov/entrez/query.fcgi?cmd=search&db=gene&term=Tnfrsf11b) | tumor necrosis factor receptor superfamily, member 11b (osteoprotegerin) |
| 814 | 0,0003095 | 0,00748 | 223,7 | 539,73 | 0,41 | [1417661_at](https://www.affymetrix.com/LinkServlet?probeset=1417661_at) | [Rdm1](http://www.ncbi.nlm.nih.gov/entrez/query.fcgi?cmd=search&db=gene&term=Rdm1) | RAD52 motif 1 |
| 815 | 0,0003112 | 0,00751 | 1574,5 | 548,68 | 2,87 | [1418241_at](https://www.affymetrix.com/LinkServlet?probeset=1418241_at) | [Usf2](http://www.ncbi.nlm.nih.gov/entrez/query.fcgi?cmd=search&db=gene&term=Usf2) | upstream transcription factor 2 |
| 816 | 0,0003116 | 0,00751 | 3998,98 | 2677,31 | 1,49 | [1428293_at](https://www.affymetrix.com/LinkServlet?probeset=1428293_at) | [Bod1](http://www.ncbi.nlm.nih.gov/entrez/query.fcgi?cmd=search&db=gene&term=Bod1) | biorientation of chromosomes in cell division 1 |
| 817 | 0,0003122 | 0,00751 | 539,92 | 1768,83 | 0,31 | [1423801_a_at](https://www.affymetrix.com/LinkServlet?probeset=1423801_a_at) | [Aprt](http://www.ncbi.nlm.nih.gov/entrez/query.fcgi?cmd=search&db=gene&term=Aprt) | adenine phosphoribosyl transferase |
| 818 | 0,000313 | 0,00751 | 3917,16 | 2212,06 | 1,77 | [1439768_x_at](https://www.affymetrix.com/LinkServlet?probeset=1439768_x_at) | [Sema4f](http://www.ncbi.nlm.nih.gov/entrez/query.fcgi?cmd=search&db=gene&term=Sema4f) | sema domain, immunoglobulin domain (Ig), TM domain, and short cytoplasmic domain |
| 819 | 0,0003131 | 0,00751 | 6,71 | 82,35 | 0,081 | [1434109_at](https://www.affymetrix.com/LinkServlet?probeset=1434109_at) | [Sh3bgrl2](http://www.ncbi.nlm.nih.gov/entrez/query.fcgi?cmd=search&db=gene&term=Sh3bgrl2) | SH3 domain binding glutamic acid-rich protein like 2 |
| 820 | 0,0003134 | 0,00751 | 7,81 | 320,7 | 0,024 | [1425784_a_at](https://www.affymetrix.com/LinkServlet?probeset=1425784_a_at) | [Olfm1](http://www.ncbi.nlm.nih.gov/entrez/query.fcgi?cmd=search&db=gene&term=Olfm1) | olfactomedin 1 |
| 821 | 0,0003138 | 0,00751 | 8,28 | 1369,47 | 0,006 | [1434202_a_at](https://www.affymetrix.com/LinkServlet?probeset=1434202_a_at) | [Fam107a](http://www.ncbi.nlm.nih.gov/entrez/query.fcgi?cmd=search&db=gene&term=Fam107a) | family with sequence similarity 107, member A |
| 822 | 0,0003141 | 0,00751 | 1165,22 | 3375,72 | 0,35 | [1438039_at](https://www.affymetrix.com/LinkServlet?probeset=1438039_at) | [Hectd1](http://www.ncbi.nlm.nih.gov/entrez/query.fcgi?cmd=search&db=gene&term=Hectd1) | HECT domain containing 1 |
| 823 | 0,0003144 | 0,00751 | 1665,43 | 6378,93 | 0,26 | [1417513_at](https://www.affymetrix.com/LinkServlet?probeset=1417513_at) | [Evi5](http://www.ncbi.nlm.nih.gov/entrez/query.fcgi?cmd=search&db=gene&term=Evi5) | ecotropic viral integration site 5 |
| 824 | 0,0003148 | 0,00751 | 1122,99 | 810,48 | 1,39 | [1428337_at](https://www.affymetrix.com/LinkServlet?probeset=1428337_at) | [Mdp1](http://www.ncbi.nlm.nih.gov/entrez/query.fcgi?cmd=search&db=gene&term=Mdp1) | magnesium-dependent phosphatase 1 |
| 825 | 0,0003181 | 0,00757 | 405,65 | 61,94 | 6,55 | [1437938_x_at](https://www.affymetrix.com/LinkServlet?probeset=1437938_x_at) | [Dnm2](http://www.ncbi.nlm.nih.gov/entrez/query.fcgi?cmd=search&db=gene&term=Dnm2) | dynamin 2 |
| 826 | 0,0003189 | 0,00757 | 1359,16 | 3092,34 | 0,44 | [1422418_s_at](https://www.affymetrix.com/LinkServlet?probeset=1422418_s_at) | [NA](http://www.ncbi.nlm.nih.gov/entrez/query.fcgi?cmd=search&db=gene&term=NA) | NA |
| 827 | 0,0003193 | 0,00757 | 50,78 | 1699,01 | 0,03 | [1437106_at](https://www.affymetrix.com/LinkServlet?probeset=1437106_at) | [Kdm5a](http://www.ncbi.nlm.nih.gov/entrez/query.fcgi?cmd=search&db=gene&term=Kdm5a) | lysine (K)-specific demethylase 5A |
| 828 | 0,0003195 | 0,00757 | 975,62 | 15182,96 | 0,064 | [1460302_at](https://www.affymetrix.com/LinkServlet?probeset=1460302_at) | [Thbs1](http://www.ncbi.nlm.nih.gov/entrez/query.fcgi?cmd=search&db=gene&term=Thbs1) | thrombospondin 1 |
| 829 | 0,0003196 | 0,00757 | 126,47 | 978,4 | 0,13 | [1456022_at](https://www.affymetrix.com/LinkServlet?probeset=1456022_at) | [Hipk2](http://www.ncbi.nlm.nih.gov/entrez/query.fcgi?cmd=search&db=gene&term=Hipk2) | homeodomain interacting protein kinase 2 |
| 830 | 0,0003196 | 0,00757 | 3200,48 | 9691,8 | 0,33 | [1452011_a_at](https://www.affymetrix.com/LinkServlet?probeset=1452011_a_at) | [Uxs1](http://www.ncbi.nlm.nih.gov/entrez/query.fcgi?cmd=search&db=gene&term=Uxs1) | UDP-glucuronate decarboxylase 1 |
| 831 | 0,0003204 | 0,00758 | 1618,84 | 523,2 | 3,09 | [1433464_at](https://www.affymetrix.com/LinkServlet?probeset=1433464_at) | [Ipo13](http://www.ncbi.nlm.nih.gov/entrez/query.fcgi?cmd=search&db=gene&term=Ipo13) | importin 13 |
| 832 | 0,0003207 | 0,00758 | 6566,9 | 3677,98 | 1,79 | [1448630_a_at](https://www.affymetrix.com/LinkServlet?probeset=1448630_a_at) | [Sdhc](http://www.ncbi.nlm.nih.gov/entrez/query.fcgi?cmd=search&db=gene&term=Sdhc) | succinate dehydrogenase complex, subunit C, integral membrane protein |
| 833 | 0,0003215 | 0,00759 | 15,97 | 213,94 | 0,075 | [1435665_at](https://www.affymetrix.com/LinkServlet?probeset=1435665_at) | [Trim30d](http://www.ncbi.nlm.nih.gov/entrez/query.fcgi?cmd=search&db=gene&term=Trim30d) | tripartite motif-containing 30D |
| 834 | 0,0003222 | 0,00759 | 58,62 | 7,68 | 7,63 | [1423778_at](https://www.affymetrix.com/LinkServlet?probeset=1423778_at) | [Usp20](http://www.ncbi.nlm.nih.gov/entrez/query.fcgi?cmd=search&db=gene&term=Usp20) | ubiquitin specific peptidase 20 |
| 835 | 0,0003223 | 0,00759 | 1273,77 | 3957,2 | 0,32 | [1426640_s_at](https://www.affymetrix.com/LinkServlet?probeset=1426640_s_at) | [Trib2](http://www.ncbi.nlm.nih.gov/entrez/query.fcgi?cmd=search&db=gene&term=Trib2) | tribbles homolog 2 (Drosophila) |
| 836 | 0,0003235 | 0,00761 | 31,54 | 203,61 | 0,15 | [1460215_at](https://www.affymetrix.com/LinkServlet?probeset=1460215_at) | [Polr1a](http://www.ncbi.nlm.nih.gov/entrez/query.fcgi?cmd=search&db=gene&term=Polr1a) | polymerase (RNA) I polypeptide A |
| 837 | 0,0003258 | 0,00766 | 4,63 | 40,51 | 0,11 | [1433615_at](https://www.affymetrix.com/LinkServlet?probeset=1433615_at) | [Tmem117](http://www.ncbi.nlm.nih.gov/entrez/query.fcgi?cmd=search&db=gene&term=Tmem117) | transmembrane protein 117 |
| 838 | 0,0003266 | 0,00766 | 516,82 | 234,29 | 2,21 | [1433797_at](https://www.affymetrix.com/LinkServlet?probeset=1433797_at) | [E130309D02Rik](http://www.ncbi.nlm.nih.gov/entrez/query.fcgi?cmd=search&db=gene&term=E130309D02Rik) | RIKEN cDNA E130309D02 gene |
| 839 | 0,0003286 | 0,0077 | 1672,19 | 939,09 | 1,78 | [1455147_at](https://www.affymetrix.com/LinkServlet?probeset=1455147_at) | [NA](http://www.ncbi.nlm.nih.gov/entrez/query.fcgi?cmd=search&db=gene&term=NA) | NA |
| 840 | 0,0003289 | 0,0077 | 24,88 | 216 | 0,12 | [1420673_a_at](https://www.affymetrix.com/LinkServlet?probeset=1420673_a_at) | [Acox2](http://www.ncbi.nlm.nih.gov/entrez/query.fcgi?cmd=search&db=gene&term=Acox2) | acyl-Coenzyme A oxidase 2, branched chain |
| 841 | 0,0003297 | 0,0077 | 601,67 | 113,46 | 5,3 | [1460205_at](https://www.affymetrix.com/LinkServlet?probeset=1460205_at) | [Dcakd](http://www.ncbi.nlm.nih.gov/entrez/query.fcgi?cmd=search&db=gene&term=Dcakd) | dephospho-CoA kinase domain containing |
| 842 | 0,0003297 | 0,0077 | 4,71 | 13,01 | 0,36 | [1422125_at](https://www.affymetrix.com/LinkServlet?probeset=1422125_at) | [Htr2b](http://www.ncbi.nlm.nih.gov/entrez/query.fcgi?cmd=search&db=gene&term=Htr2b) | 5-hydroxytryptamine (serotonin) receptor 2B |
| 843 | 0,0003302 | 0,0077 | 6,85 | 99,89 | 0,069 | [1459929_at](https://www.affymetrix.com/LinkServlet?probeset=1459929_at) | [Zfp568](http://www.ncbi.nlm.nih.gov/entrez/query.fcgi?cmd=search&db=gene&term=Zfp568) | zinc finger protein 568 |
| 844 | 0,0003315 | 0,00772 | 12782,39 | 1708,39 | 7,48 | [1423087_a_at](https://www.affymetrix.com/LinkServlet?probeset=1423087_a_at) | [Tomm6](http://www.ncbi.nlm.nih.gov/entrez/query.fcgi?cmd=search&db=gene&term=Tomm6) | translocase of outer mitochondrial membrane 6 homolog (yeast) |
| 845 | 0,0003327 | 0,00774 | 4,68 | 13,98 | 0,33 | [1445720_at](https://www.affymetrix.com/LinkServlet?probeset=1445720_at) | [Sltm](http://www.ncbi.nlm.nih.gov/entrez/query.fcgi?cmd=search&db=gene&term=Sltm) | SAFB-like, transcription modulator |
| 846 | 0,0003331 | 0,00774 | 755,37 | 327,54 | 2,31 | [1448587_at](https://www.affymetrix.com/LinkServlet?probeset=1448587_at) | [Tbc1d10a](http://www.ncbi.nlm.nih.gov/entrez/query.fcgi?cmd=search&db=gene&term=Tbc1d10a) | TBC1 domain family, member 10a |
| 847 | 0,0003339 | 0,00775 | 6,2 | 72,81 | 0,085 | [1418486_at](https://www.affymetrix.com/LinkServlet?probeset=1418486_at) | [Vnn1](http://www.ncbi.nlm.nih.gov/entrez/query.fcgi?cmd=search&db=gene&term=Vnn1) | vanin 1 |
| 848 | 0,0003346 | 0,00775 | 13,99 | 30,27 | 0,46 | [1426363_x_at](https://www.affymetrix.com/LinkServlet?probeset=1426363_x_at) | [H2afy2](http://www.ncbi.nlm.nih.gov/entrez/query.fcgi?cmd=search&db=gene&term=H2afy2) | H2A histone family, member Y2 |
| 849 | 0,0003347 | 0,00775 | 820,72 | 288,39 | 2,85 | [1428143_a_at](https://www.affymetrix.com/LinkServlet?probeset=1428143_a_at) | [Pnpla2](http://www.ncbi.nlm.nih.gov/entrez/query.fcgi?cmd=search&db=gene&term=Pnpla2) | patatin-like phospholipase domain containing 2 |
| 850 | 0,0003355 | 0,00776 | 235,57 | 96,28 | 2,45 | [1455935_at](https://www.affymetrix.com/LinkServlet?probeset=1455935_at) | [2410131K14Rik](http://www.ncbi.nlm.nih.gov/entrez/query.fcgi?cmd=search&db=gene&term=2410131K14Rik) | RIKEN cDNA 2410131K14 gene |
| 851 | 0,0003365 | 0,00777 | 810,41 | 3208,35 | 0,25 | [1448501_at](https://www.affymetrix.com/LinkServlet?probeset=1448501_at) | [Tspan6](http://www.ncbi.nlm.nih.gov/entrez/query.fcgi?cmd=search&db=gene&term=Tspan6) | tetraspanin 6 |
| 852 | 0,0003369 | 0,00777 | 79,89 | 211,66 | 0,38 | [1421834_at](https://www.affymetrix.com/LinkServlet?probeset=1421834_at) | [Pip5k1b](http://www.ncbi.nlm.nih.gov/entrez/query.fcgi?cmd=search&db=gene&term=Pip5k1b) | phosphatidylinositol-4-phosphate 5-kinase, type 1 beta |
| 853 | 0,0003373 | 0,00777 | 2289,49 | 3741 | 0,61 | [1429219_at](https://www.affymetrix.com/LinkServlet?probeset=1429219_at) | [Ikbip](http://www.ncbi.nlm.nih.gov/entrez/query.fcgi?cmd=search&db=gene&term=Ikbip) | IKBKB interacting protein |
| 854 | 0,0003374 | 0,00777 | 4,5 | 15,33 | 0,29 | [1440809_at](https://www.affymetrix.com/LinkServlet?probeset=1440809_at) | [NA](http://www.ncbi.nlm.nih.gov/entrez/query.fcgi?cmd=search&db=gene&term=NA) | NA |
| 855 | 0,0003378 | 0,00777 | 983,33 | 33,51 | 29,34 | [1436665_a_at](https://www.affymetrix.com/LinkServlet?probeset=1436665_a_at) | [Ltbp4](http://www.ncbi.nlm.nih.gov/entrez/query.fcgi?cmd=search&db=gene&term=Ltbp4) | latent transforming growth factor beta binding protein 4 |
| 856 | 0,000339 | 0,00777 | 3082,52 | 1127,92 | 2,73 | [1428791_at](https://www.affymetrix.com/LinkServlet?probeset=1428791_at) | [Ube2h](http://www.ncbi.nlm.nih.gov/entrez/query.fcgi?cmd=search&db=gene&term=Ube2h) | ubiquitin-conjugating enzyme E2H |
| 857 | 0,0003395 | 0,00777 | 1384,75 | 2880,21 | 0,48 | [1428210_s_at](https://www.affymetrix.com/LinkServlet?probeset=1428210_s_at) | [Chuk](http://www.ncbi.nlm.nih.gov/entrez/query.fcgi?cmd=search&db=gene&term=Chuk) | conserved helix-loop-helix ubiquitous kinase |
| 858 | 0,00034 | 0,00777 | 357,7 | 148,38 | 2,41 | [1416888_at](https://www.affymetrix.com/LinkServlet?probeset=1416888_at) | [Fadd](http://www.ncbi.nlm.nih.gov/entrez/query.fcgi?cmd=search&db=gene&term=Fadd) | Fas (TNFRSF6)-associated via death domain |
| 859 | 0,0003404 | 0,00777 | 173,56 | 491,94 | 0,35 | [1429613_at](https://www.affymetrix.com/LinkServlet?probeset=1429613_at) | [Dusp28](http://www.ncbi.nlm.nih.gov/entrez/query.fcgi?cmd=search&db=gene&term=Dusp28) | dual specificity phosphatase 28 |
| 860 | 0,0003406 | 0,00777 | 149,46 | 797,71 | 0,19 | [1438035_at](https://www.affymetrix.com/LinkServlet?probeset=1438035_at) | [Fam82a1](http://www.ncbi.nlm.nih.gov/entrez/query.fcgi?cmd=search&db=gene&term=Fam82a1) | family with sequence similarity 82, member A1 |
| 861 | 0,0003408 | 0,00777 | 1178,17 | 6127,43 | 0,19 | [1423488_at](https://www.affymetrix.com/LinkServlet?probeset=1423488_at) | [Mmd](http://www.ncbi.nlm.nih.gov/entrez/query.fcgi?cmd=search&db=gene&term=Mmd) | monocyte to macrophage differentiation-associated |
| 862 | 0,0003417 | 0,00777 | 2100,59 | 3566,47 | 0,59 | [1448673_at](https://www.affymetrix.com/LinkServlet?probeset=1448673_at) | [Pvrl3](http://www.ncbi.nlm.nih.gov/entrez/query.fcgi?cmd=search&db=gene&term=Pvrl3) | poliovirus receptor-related 3 |
| 863 | 0,0003421 | 0,00777 | 9,15 | 72,55 | 0,13 | [1442542_at](https://www.affymetrix.com/LinkServlet?probeset=1442542_at) | [Eya4](http://www.ncbi.nlm.nih.gov/entrez/query.fcgi?cmd=search&db=gene&term=Eya4) | eyes absent 4 homolog (Drosophila) |
| 864 | 0,0003421 | 0,00777 | 32,33 | 83,23 | 0,39 | [1440095_at](https://www.affymetrix.com/LinkServlet?probeset=1440095_at) | [NA](http://www.ncbi.nlm.nih.gov/entrez/query.fcgi?cmd=search&db=gene&term=NA) | NA |
| 865 | 0,0003425 | 0,00777 | 3288,9 | 1665,04 | 1,98 | [1437545_at](https://www.affymetrix.com/LinkServlet?probeset=1437545_at) | [Rcor1](http://www.ncbi.nlm.nih.gov/entrez/query.fcgi?cmd=search&db=gene&term=Rcor1) | REST corepressor 1 |
| 866 | 0,0003429 | 0,00777 | 17,21 | 430,13 | 0,04 | [1423414_at](https://www.affymetrix.com/LinkServlet?probeset=1423414_at) | [Ptgs1](http://www.ncbi.nlm.nih.gov/entrez/query.fcgi?cmd=search&db=gene&term=Ptgs1) | prostaglandin-endoperoxide synthase 1 |
| 867 | 0,0003433 | 0,00777 | 63,2 | 12,76 | 4,95 | [1432579_at](https://www.affymetrix.com/LinkServlet?probeset=1432579_at) | [Rsph3a](http://www.ncbi.nlm.nih.gov/entrez/query.fcgi?cmd=search&db=gene&term=Rsph3a) | radial spoke 3A homolog (Chlamydomonas) |
| 868 | 0,0003441 | 0,00777 | 576,69 | 1365,98 | 0,42 | [1449168_a_at](https://www.affymetrix.com/LinkServlet?probeset=1449168_a_at) | [Akap2](http://www.ncbi.nlm.nih.gov/entrez/query.fcgi?cmd=search&db=gene&term=Akap2) | A kinase (PRKA) anchor protein 2 |
| 869 | 0,0003441 | 0,00777 | 1473,44 | 682,73 | 2,16 | [1433817_at](https://www.affymetrix.com/LinkServlet?probeset=1433817_at) | [Agpat3](http://www.ncbi.nlm.nih.gov/entrez/query.fcgi?cmd=search&db=gene&term=Agpat3) | 1-acylglycerol-3-phosphate O-acyltransferase 3 |
| 870 | 0,0003442 | 0,00777 | 33,71 | 123,7 | 0,27 | [1439857_at](https://www.affymetrix.com/LinkServlet?probeset=1439857_at) | [NA](http://www.ncbi.nlm.nih.gov/entrez/query.fcgi?cmd=search&db=gene&term=NA) | NA |
| 871 | 0,0003443 | 0,00777 | 648,24 | 1794,48 | 0,36 | [1418895_at](https://www.affymetrix.com/LinkServlet?probeset=1418895_at) | [Skap2](http://www.ncbi.nlm.nih.gov/entrez/query.fcgi?cmd=search&db=gene&term=Skap2) | src family associated phosphoprotein 2 |
| 872 | 0,0003445 | 0,00777 | 248,68 | 467,98 | 0,53 | [1415731_at](https://www.affymetrix.com/LinkServlet?probeset=1415731_at) | [Angel2](http://www.ncbi.nlm.nih.gov/entrez/query.fcgi?cmd=search&db=gene&term=Angel2) | angel homolog 2 (Drosophila) |
| 873 | 0,0003457 | 0,00779 | 5,74 | 24,69 | 0,23 | [1445235_at](https://www.affymetrix.com/LinkServlet?probeset=1445235_at) | [NA](http://www.ncbi.nlm.nih.gov/entrez/query.fcgi?cmd=search&db=gene&term=NA) | NA |
| 874 | 0,0003479 | 0,00782 | 29745,14 | 12900,19 | 2,31 | [1438839_a_at](https://www.affymetrix.com/LinkServlet?probeset=1438839_a_at) | [Ywhae](http://www.ncbi.nlm.nih.gov/entrez/query.fcgi?cmd=search&db=gene&term=Ywhae) | tyrosine 3-monooxygenase/tryptophan 5-monooxygenase activation protein, epsilon polypeptide |
| 875 | 0,0003481 | 0,00782 | 4,57 | 92,53 | 0,049 | [1437466_at](https://www.affymetrix.com/LinkServlet?probeset=1437466_at) | [Alcam](http://www.ncbi.nlm.nih.gov/entrez/query.fcgi?cmd=search&db=gene&term=Alcam) | activated leukocyte cell adhesion molecule |
| 876 | 0,0003498 | 0,00784 | 1287,86 | 334,63 | 3,85 | [1428243_at](https://www.affymetrix.com/LinkServlet?probeset=1428243_at) | [1700021K19Rik](http://www.ncbi.nlm.nih.gov/entrez/query.fcgi?cmd=search&db=gene&term=1700021K19Rik) | RIKEN cDNA 1700021K19 gene |
| 877 | 0,0003499 | 0,00784 | 5703,76 | 8860,93 | 0,64 | [1425568_a_at](https://www.affymetrix.com/LinkServlet?probeset=1425568_a_at) | [Tmem33](http://www.ncbi.nlm.nih.gov/entrez/query.fcgi?cmd=search&db=gene&term=Tmem33) | transmembrane protein 33 |
| 878 | 0,0003502 | 0,00784 | 2609,21 | 1604,26 | 1,63 | [1433805_at](https://www.affymetrix.com/LinkServlet?probeset=1433805_at) | [Jak1](http://www.ncbi.nlm.nih.gov/entrez/query.fcgi?cmd=search&db=gene&term=Jak1) | Janus kinase 1 |
| 879 | 0,0003505 | 0,00784 | 1440,52 | 712,87 | 2,02 | [1448672_a_at](https://www.affymetrix.com/LinkServlet?probeset=1448672_a_at) | [Arfgap2](http://www.ncbi.nlm.nih.gov/entrez/query.fcgi?cmd=search&db=gene&term=Arfgap2) | ADP-ribosylation factor GTPase activating protein 2 |
| 880 | 0,0003512 | 0,00784 | 436,01 | 251,12 | 1,74 | [1425296_a_at](https://www.affymetrix.com/LinkServlet?probeset=1425296_a_at) | [Rgs3](http://www.ncbi.nlm.nih.gov/entrez/query.fcgi?cmd=search&db=gene&term=Rgs3) | regulator of G-protein signaling 3 |
| 881 | 0,0003512 | 0,00784 | 15889,51 | 21439,13 | 0,74 | [1426392_a_at](https://www.affymetrix.com/LinkServlet?probeset=1426392_a_at) | [Actr3](http://www.ncbi.nlm.nih.gov/entrez/query.fcgi?cmd=search&db=gene&term=Actr3) | ARP3 actin-related protein 3 homolog (yeast) |
| 882 | 0,000352 | 0,00785 | 6,88 | 104,77 | 0,066 | [1440146_at](https://www.affymetrix.com/LinkServlet?probeset=1440146_at) | [Vps13a](http://www.ncbi.nlm.nih.gov/entrez/query.fcgi?cmd=search&db=gene&term=Vps13a) | vacuolar protein sorting 13A (yeast) |
| 883 | 0,0003538 | 0,00787 | 8,27 | 76,79 | 0,11 | [1441975_at](https://www.affymetrix.com/LinkServlet?probeset=1441975_at) | [Acpp](http://www.ncbi.nlm.nih.gov/entrez/query.fcgi?cmd=search&db=gene&term=Acpp) | acid phosphatase, prostate |
| 884 | 0,0003539 | 0,00787 | 6,8 | 120,02 | 0,057 | [1420563_at](https://www.affymetrix.com/LinkServlet?probeset=1420563_at) | [Gria3](http://www.ncbi.nlm.nih.gov/entrez/query.fcgi?cmd=search&db=gene&term=Gria3) | glutamate receptor, ionotropic, AMPA3 (alpha 3) |
| 885 | 0,0003553 | 0,00789 | 2010,63 | 4553,38 | 0,44 | [1454739_at](https://www.affymetrix.com/LinkServlet?probeset=1454739_at) | [Cdc27](http://www.ncbi.nlm.nih.gov/entrez/query.fcgi?cmd=search&db=gene&term=Cdc27) | cell division cycle 27 homolog (S. cerevisiae) |
| 886 | 0,0003556 | 0,00789 | 131,93 | 72,7 | 1,81 | [1424878_at](https://www.affymetrix.com/LinkServlet?probeset=1424878_at) | [NA](http://www.ncbi.nlm.nih.gov/entrez/query.fcgi?cmd=search&db=gene&term=NA) | NA |
| 887 | 0,0003573 | 0,00791 | 1368,04 | 739,57 | 1,85 | [1424320_a_at](https://www.affymetrix.com/LinkServlet?probeset=1424320_a_at) | [Traf7](http://www.ncbi.nlm.nih.gov/entrez/query.fcgi?cmd=search&db=gene&term=Traf7) | TNF receptor-associated factor 7 |
| 888 | 0,0003575 | 0,00791 | 2430,67 | 8351,7 | 0,29 | [1434302_at](https://www.affymetrix.com/LinkServlet?probeset=1434302_at) | [Raph1](http://www.ncbi.nlm.nih.gov/entrez/query.fcgi?cmd=search&db=gene&term=Raph1) | Ras association (RalGDS/AF-6) and pleckstrin homology domains 1 |
| 889 | 0,0003576 | 0,00791 | 566,83 | 74,41 | 7,62 | [1425276_at](https://www.affymetrix.com/LinkServlet?probeset=1425276_at) | [Fbrs](http://www.ncbi.nlm.nih.gov/entrez/query.fcgi?cmd=search&db=gene&term=Fbrs) | fibrosin |
| 890 | 0,0003579 | 0,00791 | 218,84 | 82,36 | 2,66 | [1428271_at](https://www.affymetrix.com/LinkServlet?probeset=1428271_at) | [Acbd4](http://www.ncbi.nlm.nih.gov/entrez/query.fcgi?cmd=search&db=gene&term=Acbd4) | acyl-Coenzyme A binding domain containing 4 |
| 891 | 0,0003585 | 0,00791 | 3755,71 | 581,44 | 6,46 | [1422799_at](https://www.affymetrix.com/LinkServlet?probeset=1422799_at) | [Prrc2a](http://www.ncbi.nlm.nih.gov/entrez/query.fcgi?cmd=search&db=gene&term=Prrc2a) | proline-rich coiled-coil 2A |
| 892 | 0,0003587 | 0,00791 | 4,4 | 9,82 | 0,45 | [1445965_at](https://www.affymetrix.com/LinkServlet?probeset=1445965_at) | [NA](http://www.ncbi.nlm.nih.gov/entrez/query.fcgi?cmd=search&db=gene&term=NA) | NA |
| 893 | 0,0003595 | 0,00791 | 8,51 | 433,9 | 0,02 | [1455905_at](https://www.affymetrix.com/LinkServlet?probeset=1455905_at) | [2610507B11Rik](http://www.ncbi.nlm.nih.gov/entrez/query.fcgi?cmd=search&db=gene&term=2610507B11Rik) | RIKEN cDNA 2610507B11 gene |
| 894 | 0,0003603 | 0,00791 | 56,88 | 1148,26 | 0,05 | [1441170_a_at](https://www.affymetrix.com/LinkServlet?probeset=1441170_a_at) | [Dab2ip](http://www.ncbi.nlm.nih.gov/entrez/query.fcgi?cmd=search&db=gene&term=Dab2ip) | disabled homolog 2 (Drosophila) interacting protein |
| 895 | 0,0003608 | 0,00791 | 6817,92 | 3020,36 | 2,26 | [1460718_s_at](https://www.affymetrix.com/LinkServlet?probeset=1460718_s_at) | [Mtch1](http://www.ncbi.nlm.nih.gov/entrez/query.fcgi?cmd=search&db=gene&term=Mtch1) | mitochondrial carrier homolog 1 (C. elegans) |
| 896 | 0,0003613 | 0,00791 | 173,45 | 904,58 | 0,19 | [1426315_a_at](https://www.affymetrix.com/LinkServlet?probeset=1426315_a_at) | [6330416G13Rik](http://www.ncbi.nlm.nih.gov/entrez/query.fcgi?cmd=search&db=gene&term=6330416G13Rik) | RIKEN cDNA 6330416G13 gene |
| 897 | 0,0003615 | 0,00791 | 1672,86 | 698,6 | 2,39 | [1423518_at](https://www.affymetrix.com/LinkServlet?probeset=1423518_at) | [Csk](http://www.ncbi.nlm.nih.gov/entrez/query.fcgi?cmd=search&db=gene&term=Csk) | c-src tyrosine kinase |
| 898 | 0,0003626 | 0,00791 | 336,42 | 548,62 | 0,61 | [1423948_at](https://www.affymetrix.com/LinkServlet?probeset=1423948_at) | [Bag2](http://www.ncbi.nlm.nih.gov/entrez/query.fcgi?cmd=search&db=gene&term=Bag2) | BCL2-associated athanogene 2 |
| 899 | 0,0003627 | 0,00791 | 2931,58 | 999,32 | 2,93 | [1451099_at](https://www.affymetrix.com/LinkServlet?probeset=1451099_at) | [Esyt1](http://www.ncbi.nlm.nih.gov/entrez/query.fcgi?cmd=search&db=gene&term=Esyt1) | extended synaptotagmin-like protein 1 |
| 900 | 0,0003628 | 0,00791 | 365,81 | 72,35 | 5,06 | [1423978_at](https://www.affymetrix.com/LinkServlet?probeset=1423978_at) | [Sbk1](http://www.ncbi.nlm.nih.gov/entrez/query.fcgi?cmd=search&db=gene&term=Sbk1) | SH3-binding kinase 1 |
| 901 | 0,000363 | 0,00791 | 1020,32 | 377,67 | 2,7 | [1428708_x_at](https://www.affymetrix.com/LinkServlet?probeset=1428708_x_at) | [Ptms](http://www.ncbi.nlm.nih.gov/entrez/query.fcgi?cmd=search&db=gene&term=Ptms) | parathymosin |
| 902 | 0,0003631 | 0,00791 | 1561,64 | 2318,95 | 0,67 | [1434238_at](https://www.affymetrix.com/LinkServlet?probeset=1434238_at) | [Taf2](http://www.ncbi.nlm.nih.gov/entrez/query.fcgi?cmd=search&db=gene&term=Taf2) | TAF2 RNA polymerase II, TATA box binding protein (TBP)-associated factor |
| 903 | 0,0003632 | 0,00791 | 6,67 | 27,51 | 0,24 | [1452421_at](https://www.affymetrix.com/LinkServlet?probeset=1452421_at) | [Hoxa3](http://www.ncbi.nlm.nih.gov/entrez/query.fcgi?cmd=search&db=gene&term=Hoxa3) | homeobox A3 |
| 904 | 0,0003634 | 0,00791 | 308,2 | 33,16 | 9,29 | [1449302_at](https://www.affymetrix.com/LinkServlet?probeset=1449302_at) | [Abca2](http://www.ncbi.nlm.nih.gov/entrez/query.fcgi?cmd=search&db=gene&term=Abca2) | ATP-binding cassette, sub-family A (ABC1), member 2 |
| 905 | 0,0003638 | 0,00791 | 10,39 | 47,51 | 0,22 | [1447875_x_at](https://www.affymetrix.com/LinkServlet?probeset=1447875_x_at) | [Zfp800](http://www.ncbi.nlm.nih.gov/entrez/query.fcgi?cmd=search&db=gene&term=Zfp800) | zinc finger protein 800 |
| 906 | 0,0003646 | 0,00791 | 1744,61 | 505,55 | 3,45 | [1436732_s_at](https://www.affymetrix.com/LinkServlet?probeset=1436732_s_at) | [Fbxw8](http://www.ncbi.nlm.nih.gov/entrez/query.fcgi?cmd=search&db=gene&term=Fbxw8) | F-box and WD-40 domain protein 8 |
| 907 | 0,000365 | 0,00791 | 1192,17 | 2009,73 | 0,59 | [1417195_at](https://www.affymetrix.com/LinkServlet?probeset=1417195_at) | [Wwc2](http://www.ncbi.nlm.nih.gov/entrez/query.fcgi?cmd=search&db=gene&term=Wwc2) | WW, C2 and coiled-coil domain containing 2 |
| 908 | 0,0003651 | 0,00791 | 3931,96 | 837,3 | 4,7 | [1449055_x_at](https://www.affymetrix.com/LinkServlet?probeset=1449055_x_at) | [Pcbp4](http://www.ncbi.nlm.nih.gov/entrez/query.fcgi?cmd=search&db=gene&term=Pcbp4) | poly(rC) binding protein 4 |
| 909 | 0,0003664 | 0,00792 | 212,16 | 861,45 | 0,25 | [1460203_at](https://www.affymetrix.com/LinkServlet?probeset=1460203_at) | [Itpr1](http://www.ncbi.nlm.nih.gov/entrez/query.fcgi?cmd=search&db=gene&term=Itpr1) | inositol 1,4,5-triphosphate receptor 1 |
| 910 | 0,0003666 | 0,00792 | 8,56 | 151,11 | 0,057 | [1422871_at](https://www.affymetrix.com/LinkServlet?probeset=1422871_at) | [Kcnj12](http://www.ncbi.nlm.nih.gov/entrez/query.fcgi?cmd=search&db=gene&term=Kcnj12) | potassium inwardly-rectifying channel, subfamily J, member 12 |
| 911 | 0,0003667 | 0,00792 | 6274,31 | 3377,41 | 1,86 | [1418703_at](https://www.affymetrix.com/LinkServlet?probeset=1418703_at) | [Rbms1](http://www.ncbi.nlm.nih.gov/entrez/query.fcgi?cmd=search&db=gene&term=Rbms1) | RNA binding motif, single stranded interacting protein 1 |
| 912 | 0,0003673 | 0,00792 | 17,74 | 59,69 | 0,3 | [1456950_at](https://www.affymetrix.com/LinkServlet?probeset=1456950_at) | [Alms1](http://www.ncbi.nlm.nih.gov/entrez/query.fcgi?cmd=search&db=gene&term=Alms1) | Alstrom syndrome 1 homolog (human) |
| 913 | 0,0003685 | 0,00794 | 1083,48 | 1907,66 | 0,57 | [1460241_a_at](https://www.affymetrix.com/LinkServlet?probeset=1460241_a_at) | [St3gal5](http://www.ncbi.nlm.nih.gov/entrez/query.fcgi?cmd=search&db=gene&term=St3gal5) | ST3 beta-galactoside alpha-2,3-sialyltransferase 5 |
| 914 | 0,0003695 | 0,00795 | 44 | 716,29 | 0,061 | [1429273_at](https://www.affymetrix.com/LinkServlet?probeset=1429273_at) | [Bmper](http://www.ncbi.nlm.nih.gov/entrez/query.fcgi?cmd=search&db=gene&term=Bmper) | BMP-binding endothelial regulator |
| 915 | 0,0003719 | 0,00798 | 65,49 | 16,74 | 3,91 | [1454629_at](https://www.affymetrix.com/LinkServlet?probeset=1454629_at) | [Iffo1](http://www.ncbi.nlm.nih.gov/entrez/query.fcgi?cmd=search&db=gene&term=Iffo1) | intermediate filament family orphan 1 |
| 916 | 0,0003719 | 0,00798 | 757,34 | 303,16 | 2,5 | [1423733_a_at](https://www.affymetrix.com/LinkServlet?probeset=1423733_a_at) | [Fiz1](http://www.ncbi.nlm.nih.gov/entrez/query.fcgi?cmd=search&db=gene&term=Fiz1) | Flt3 interacting zinc finger protein 1 |
| 917 | 0,0003725 | 0,00799 | 782,1 | 234,27 | 3,34 | [1417153_at](https://www.affymetrix.com/LinkServlet?probeset=1417153_at) | [Nacc2](http://www.ncbi.nlm.nih.gov/entrez/query.fcgi?cmd=search&db=gene&term=Nacc2) | nucleus accumbens associated 2, BEN and BTB (POZ) domain containing |
| 918 | 0,0003731 | 0,00799 | 1307,75 | 590,21 | 2,22 | [1416722_at](https://www.affymetrix.com/LinkServlet?probeset=1416722_at) | [Hmg20a](http://www.ncbi.nlm.nih.gov/entrez/query.fcgi?cmd=search&db=gene&term=Hmg20a) | high mobility group 20A |
| 919 | 0,0003739 | 0,008 | 689,65 | 348,62 | 1,98 | [1422847_a_at](https://www.affymetrix.com/LinkServlet?probeset=1422847_a_at) | [Prkcd](http://www.ncbi.nlm.nih.gov/entrez/query.fcgi?cmd=search&db=gene&term=Prkcd) | protein kinase C, delta |
| 920 | 0,0003746 | 0,00801 | 12715,89 | 8923,15 | 1,43 | [1450714_at](https://www.affymetrix.com/LinkServlet?probeset=1450714_at) | [Azin1](http://www.ncbi.nlm.nih.gov/entrez/query.fcgi?cmd=search&db=gene&term=Azin1) | antizyme inhibitor 1 |
| 921 | 0,0003757 | 0,00802 | 1941,66 | 780,7 | 2,49 | [1434591_at](https://www.affymetrix.com/LinkServlet?probeset=1434591_at) | [Zfp862](http://www.ncbi.nlm.nih.gov/entrez/query.fcgi?cmd=search&db=gene&term=Zfp862) | zinc finger protein 862 |
| 922 | 0,0003772 | 0,00805 | 2233,15 | 8078,62 | 0,28 | [1424770_at](https://www.affymetrix.com/LinkServlet?probeset=1424770_at) | [Cald1](http://www.ncbi.nlm.nih.gov/entrez/query.fcgi?cmd=search&db=gene&term=Cald1) | caldesmon 1 |
| 923 | 0,0003776 | 0,00805 | 211,26 | 530,44 | 0,4 | [1452232_at](https://www.affymetrix.com/LinkServlet?probeset=1452232_at) | [Galnt7](http://www.ncbi.nlm.nih.gov/entrez/query.fcgi?cmd=search&db=gene&term=Galnt7) | UDP-N-acetyl-alpha-D-galactosamine: polypeptide N-acetylgalactosaminyltransferase 7 |
| 924 | 0,0003816 | 0,00812 | 23,19 | 935,28 | 0,025 | [1416414_at](https://www.affymetrix.com/LinkServlet?probeset=1416414_at) | [Emilin1](http://www.ncbi.nlm.nih.gov/entrez/query.fcgi?cmd=search&db=gene&term=Emilin1) | elastin microfibril interfacer 1 |
| 925 | 0,000382 | 0,00812 | 1437,18 | 710,52 | 2,02 | [1424459_at](https://www.affymetrix.com/LinkServlet?probeset=1424459_at) | [Lpcat1](http://www.ncbi.nlm.nih.gov/entrez/query.fcgi?cmd=search&db=gene&term=Lpcat1) | lysophosphatidylcholine acyltransferase 1 |
| 926 | 0,0003831 | 0,00814 | 387,41 | 29,65 | 13,07 | [1442408_at](https://www.affymetrix.com/LinkServlet?probeset=1442408_at) | [Sulf2](http://www.ncbi.nlm.nih.gov/entrez/query.fcgi?cmd=search&db=gene&term=Sulf2) | sulfatase 2 |
| 927 | 0,0003845 | 0,00816 | 5605,61 | 2526,21 | 2,22 | [1454696_at](https://www.affymetrix.com/LinkServlet?probeset=1454696_at) | [Gnb1](http://www.ncbi.nlm.nih.gov/entrez/query.fcgi?cmd=search&db=gene&term=Gnb1) | guanine nucleotide binding protein (G protein), beta 1 |
| 928 | 0,0003852 | 0,00816 | 701,65 | 331,75 | 2,11 | [1451118_a_at](https://www.affymetrix.com/LinkServlet?probeset=1451118_a_at) | [Fam53a](http://www.ncbi.nlm.nih.gov/entrez/query.fcgi?cmd=search&db=gene&term=Fam53a) | family with sequence similarity 53, member A |
| 929 | 0,0003855 | 0,00816 | 3210,94 | 1878 | 1,71 | [1416782_s_at](https://www.affymetrix.com/LinkServlet?probeset=1416782_s_at) | [Praf2](http://www.ncbi.nlm.nih.gov/entrez/query.fcgi?cmd=search&db=gene&term=Praf2) | PRA1 domain family 2 |
| 930 | 0,0003862 | 0,00817 | 1438,4 | 585,65 | 2,46 | [1442883_s_at](https://www.affymetrix.com/LinkServlet?probeset=1442883_s_at) | [Fam108a](http://www.ncbi.nlm.nih.gov/entrez/query.fcgi?cmd=search&db=gene&term=Fam108a) | family with sequence similarity 108, member A |
| 931 | 0,000387 | 0,00818 | 929,13 | 5,01 | 185,29 | [1427797_s_at](https://www.affymetrix.com/LinkServlet?probeset=1427797_s_at) | [NA](http://www.ncbi.nlm.nih.gov/entrez/query.fcgi?cmd=search&db=gene&term=NA) | NA |
| 932 | 0,0003887 | 0,0082 | 80,06 | 316,06 | 0,25 | [1456091_at](https://www.affymetrix.com/LinkServlet?probeset=1456091_at) | [Sec22c](http://www.ncbi.nlm.nih.gov/entrez/query.fcgi?cmd=search&db=gene&term=Sec22c) | SEC22 vesicle trafficking protein homolog C (S. cerevisiae) |
| 933 | 0,0003891 | 0,0082 | 45,19 | 65,86 | 0,69 | [1423401_at](https://www.affymetrix.com/LinkServlet?probeset=1423401_at) | [Etv6](http://www.ncbi.nlm.nih.gov/entrez/query.fcgi?cmd=search&db=gene&term=Etv6) | ets variant gene 6 (TEL oncogene) |
| 934 | 0,0003892 | 0,0082 | 8,06 | 97,71 | 0,082 | [1429051_s_at](https://www.affymetrix.com/LinkServlet?probeset=1429051_s_at) | [Sox11](http://www.ncbi.nlm.nih.gov/entrez/query.fcgi?cmd=search&db=gene&term=Sox11) | SRY-box containing gene 11 |
| 935 | 0,0003906 | 0,0082 | 24,26 | 11,11 | 2,18 | [1459497_at](https://www.affymetrix.com/LinkServlet?probeset=1459497_at) | [NA](http://www.ncbi.nlm.nih.gov/entrez/query.fcgi?cmd=search&db=gene&term=NA) | NA |
| 936 | 0,0003907 | 0,0082 | 715,59 | 189,28 | 3,78 | [1417664_a_at](https://www.affymetrix.com/LinkServlet?probeset=1417664_a_at) | [Ndrg3](http://www.ncbi.nlm.nih.gov/entrez/query.fcgi?cmd=search&db=gene&term=Ndrg3) | N-myc downstream regulated gene 3 |
| 937 | 0,0003908 | 0,0082 | 393,21 | 226,83 | 1,73 | [1436089_at](https://www.affymetrix.com/LinkServlet?probeset=1436089_at) | [Ints6](http://www.ncbi.nlm.nih.gov/entrez/query.fcgi?cmd=search&db=gene&term=Ints6) | integrator complex subunit 6 |
| 938 | 0,0003917 | 0,00821 | 971,67 | 2193,52 | 0,44 | [1424488_a_at](https://www.affymetrix.com/LinkServlet?probeset=1424488_a_at) | [Ppa2](http://www.ncbi.nlm.nih.gov/entrez/query.fcgi?cmd=search&db=gene&term=Ppa2) | pyrophosphatase (inorganic) 2 |
| 939 | 0,000392 | 0,00821 | 393,15 | 51,32 | 7,66 | [1416348_at](https://www.affymetrix.com/LinkServlet?probeset=1416348_at) | [Men1](http://www.ncbi.nlm.nih.gov/entrez/query.fcgi?cmd=search&db=gene&term=Men1) | multiple endocrine neoplasia 1 |
| 940 | 0,0003928 | 0,00822 | 39,94 | 306,46 | 0,13 | [1434728_at](https://www.affymetrix.com/LinkServlet?probeset=1434728_at) | [Gria3](http://www.ncbi.nlm.nih.gov/entrez/query.fcgi?cmd=search&db=gene&term=Gria3) | glutamate receptor, ionotropic, AMPA3 (alpha 3) |
| 941 | 0,0003937 | 0,00823 | 15,72 | 111,65 | 0,14 | [1443183_at](https://www.affymetrix.com/LinkServlet?probeset=1443183_at) | [Huwe1](http://www.ncbi.nlm.nih.gov/entrez/query.fcgi?cmd=search&db=gene&term=Huwe1) | HECT, UBA and WWE domain containing 1 |
| 942 | 0,0003955 | 0,00825 | 4,7 | 14,19 | 0,33 | [1451229_at](https://www.affymetrix.com/LinkServlet?probeset=1451229_at) | [Hdac11](http://www.ncbi.nlm.nih.gov/entrez/query.fcgi?cmd=search&db=gene&term=Hdac11) | histone deacetylase 11 |
| 943 | 0,0003958 | 0,00825 | 1299,65 | 847,88 | 1,53 | [1435328_at](https://www.affymetrix.com/LinkServlet?probeset=1435328_at) | [Cyhr1](http://www.ncbi.nlm.nih.gov/entrez/query.fcgi?cmd=search&db=gene&term=Cyhr1) | cysteine and histidine rich 1 |
| 944 | 0,0003961 | 0,00825 | 9,08 | 800,52 | 0,011 | [1424800_at](https://www.affymetrix.com/LinkServlet?probeset=1424800_at) | [Enah](http://www.ncbi.nlm.nih.gov/entrez/query.fcgi?cmd=search&db=gene&term=Enah) | enabled homolog (Drosophila) |
| 945 | 0,0003971 | 0,00826 | 67,57 | 265,75 | 0,25 | [1457552_at](https://www.affymetrix.com/LinkServlet?probeset=1457552_at) | [Zfp295](http://www.ncbi.nlm.nih.gov/entrez/query.fcgi?cmd=search&db=gene&term=Zfp295) | zinc finger protein 295 |
| 946 | 0,0003982 | 0,00828 | 12291,5 | 18098,65 | 0,68 | [1448425_at](https://www.affymetrix.com/LinkServlet?probeset=1448425_at) | [Eif3a](http://www.ncbi.nlm.nih.gov/entrez/query.fcgi?cmd=search&db=gene&term=Eif3a) | eukaryotic translation initiation factor 3, subunit A |
| 947 | 0,0003987 | 0,00828 | 5988,78 | 7842,76 | 0,76 | [1423758_at](https://www.affymetrix.com/LinkServlet?probeset=1423758_at) | [G3bp2](http://www.ncbi.nlm.nih.gov/entrez/query.fcgi?cmd=search&db=gene&term=G3bp2) | GTPase activating protein (SH3 domain) binding protein 2 |
| 948 | 0,0003991 | 0,00828 | 591,64 | 334,85 | 1,77 | [1422591_at](https://www.affymetrix.com/LinkServlet?probeset=1422591_at) | [Tceb3](http://www.ncbi.nlm.nih.gov/entrez/query.fcgi?cmd=search&db=gene&term=Tceb3) | transcription elongation factor B (SIII), polypeptide 3 |
| 949 | 0,0003996 | 0,00828 | 901,61 | 187,15 | 4,82 | [1424428_at](https://www.affymetrix.com/LinkServlet?probeset=1424428_at) | [Ino80e](http://www.ncbi.nlm.nih.gov/entrez/query.fcgi?cmd=search&db=gene&term=Ino80e) | INO80 complex subunit E |
| 950 | 0,0004001 | 0,00828 | 311,23 | 170,04 | 1,83 | [1424550_at](https://www.affymetrix.com/LinkServlet?probeset=1424550_at) | [Zfyve27](http://www.ncbi.nlm.nih.gov/entrez/query.fcgi?cmd=search&db=gene&term=Zfyve27) | zinc finger, FYVE domain containing 27 |
| 951 | 0,0004004 | 0,00828 | 210,42 | 1409,07 | 0,15 | [1421106_at](https://www.affymetrix.com/LinkServlet?probeset=1421106_at) | [Jag1](http://www.ncbi.nlm.nih.gov/entrez/query.fcgi?cmd=search&db=gene&term=Jag1) | jagged 1 |
| 952 | 0,0004006 | 0,00828 | 1305,61 | 3776,88 | 0,35 | [1433536_at](https://www.affymetrix.com/LinkServlet?probeset=1433536_at) | [Lrp11](http://www.ncbi.nlm.nih.gov/entrez/query.fcgi?cmd=search&db=gene&term=Lrp11) | low density lipoprotein receptor-related protein 11 |
| 953 | 0,0004021 | 0,0083 | 9,82 | 118,58 | 0,083 | [1460086_at](https://www.affymetrix.com/LinkServlet?probeset=1460086_at) | [Rc3h2](http://www.ncbi.nlm.nih.gov/entrez/query.fcgi?cmd=search&db=gene&term=Rc3h2) | ring finger and CCCH-type zinc finger domains 2 |
| 954 | 0,0004027 | 0,0083 | 9,13 | 136,81 | 0,067 | [1453427_at](https://www.affymetrix.com/LinkServlet?probeset=1453427_at) | [Csnk2a1](http://www.ncbi.nlm.nih.gov/entrez/query.fcgi?cmd=search&db=gene&term=Csnk2a1) | casein kinase 2, alpha 1 polypeptide |
| 955 | 0,000403 | 0,0083 | 5,14 | 42,75 | 0,12 | [1442735_at](https://www.affymetrix.com/LinkServlet?probeset=1442735_at) | [NA](http://www.ncbi.nlm.nih.gov/entrez/query.fcgi?cmd=search&db=gene&term=NA) | NA |
| 956 | 0,0004041 | 0,00831 | 4,61 | 14,65 | 0,31 | [1438731_at](https://www.affymetrix.com/LinkServlet?probeset=1438731_at) | [Sgsh](http://www.ncbi.nlm.nih.gov/entrez/query.fcgi?cmd=search&db=gene&term=Sgsh) | N-sulfoglucosamine sulfohydrolase (sulfamidase) |
| 957 | 0,0004048 | 0,00832 | 802,15 | 1507,53 | 0,53 | [1423605_a_at](https://www.affymetrix.com/LinkServlet?probeset=1423605_a_at) | [Mdm2](http://www.ncbi.nlm.nih.gov/entrez/query.fcgi?cmd=search&db=gene&term=Mdm2) | transformed mouse 3T3 cell double minute 2 |
| 958 | 0,0004056 | 0,00833 | 7,06 | 35,29 | 0,2 | [1442489_at](https://www.affymetrix.com/LinkServlet?probeset=1442489_at) | [D1Ertd564e](http://www.ncbi.nlm.nih.gov/entrez/query.fcgi?cmd=search&db=gene&term=D1Ertd564e) | DNA segment, Chr 1, ERATO Doi 564, expressed |
| 959 | 0,0004073 | 0,00835 | 1081,03 | 1541,64 | 0,7 | [1434842_s_at](https://www.affymetrix.com/LinkServlet?probeset=1434842_s_at) | [Upf3b](http://www.ncbi.nlm.nih.gov/entrez/query.fcgi?cmd=search&db=gene&term=Upf3b) | UPF3 regulator of nonsense transcripts homolog B (yeast) |
| 960 | 0,0004079 | 0,00836 | 112,15 | 43,83 | 2,56 | [1455071_at](https://www.affymetrix.com/LinkServlet?probeset=1455071_at) | [Zbtb7b](http://www.ncbi.nlm.nih.gov/entrez/query.fcgi?cmd=search&db=gene&term=Zbtb7b) | zinc finger and BTB domain containing 7B |
| 961 | 0,0004092 | 0,00837 | 25,43 | 802,35 | 0,032 | [1424077_at](https://www.affymetrix.com/LinkServlet?probeset=1424077_at) | [Gdpd1](http://www.ncbi.nlm.nih.gov/entrez/query.fcgi?cmd=search&db=gene&term=Gdpd1) | glycerophosphodiester phosphodiesterase domain containing 1 |
| 962 | 0,0004093 | 0,00837 | 4,56 | 19,13 | 0,24 | [1418175_at](https://www.affymetrix.com/LinkServlet?probeset=1418175_at) | [Vdr](http://www.ncbi.nlm.nih.gov/entrez/query.fcgi?cmd=search&db=gene&term=Vdr) | vitamin D receptor |
| 963 | 0,0004098 | 0,00837 | 964,89 | 181,48 | 5,32 | [1415757_at](https://www.affymetrix.com/LinkServlet?probeset=1415757_at) | [Gbf1](http://www.ncbi.nlm.nih.gov/entrez/query.fcgi?cmd=search&db=gene&term=Gbf1) | golgi-specific brefeldin A-resistance factor 1 |
| 964 | 0,0004105 | 0,00837 | 235,01 | 4,7 | 49,95 | [1423671_at](https://www.affymetrix.com/LinkServlet?probeset=1423671_at) | [Dner](http://www.ncbi.nlm.nih.gov/entrez/query.fcgi?cmd=search&db=gene&term=Dner) | delta/notch-like EGF-related receptor |
| 965 | 0,0004128 | 0,0084 | 282,99 | 52,52 | 5,39 | [1439348_at](https://www.affymetrix.com/LinkServlet?probeset=1439348_at) | [S100a10](http://www.ncbi.nlm.nih.gov/entrez/query.fcgi?cmd=search&db=gene&term=S100a10) | S100 calcium binding protein A10 (calpactin) |
| 966 | 0,0004128 | 0,0084 | 4081,69 | 2201,76 | 1,85 | [1426756_at](https://www.affymetrix.com/LinkServlet?probeset=1426756_at) | [Galnt2](http://www.ncbi.nlm.nih.gov/entrez/query.fcgi?cmd=search&db=gene&term=Galnt2) | UDP-N-acetyl-alpha-D-galactosamine:polypeptide N-acetylgalactosaminyltransferase 2 |
| 967 | 0,0004131 | 0,0084 | 115,25 | 1089,86 | 0,11 | [1452592_at](https://www.affymetrix.com/LinkServlet?probeset=1452592_at) | [Mgst2](http://www.ncbi.nlm.nih.gov/entrez/query.fcgi?cmd=search&db=gene&term=Mgst2) | microsomal glutathione S-transferase 2 |
| 968 | 0,0004132 | 0,0084 | 1084,13 | 20,28 | 53,47 | [1416322_at](https://www.affymetrix.com/LinkServlet?probeset=1416322_at) | [Prelp](http://www.ncbi.nlm.nih.gov/entrez/query.fcgi?cmd=search&db=gene&term=Prelp) | proline arginine-rich end leucine-rich repeat |
| 969 | 0,0004143 | 0,00841 | 171,54 | 288,47 | 0,59 | [1437856_at](https://www.affymetrix.com/LinkServlet?probeset=1437856_at) | [Ipmk](http://www.ncbi.nlm.nih.gov/entrez/query.fcgi?cmd=search&db=gene&term=Ipmk) | inositol polyphosphate multikinase |
| 970 | 0,0004149 | 0,00841 | 528,16 | 2655,48 | 0,2 | [1452236_at](https://www.affymetrix.com/LinkServlet?probeset=1452236_at) | [Abcf1](http://www.ncbi.nlm.nih.gov/entrez/query.fcgi?cmd=search&db=gene&term=Abcf1) | ATP-binding cassette, sub-family F (GCN20), member 1 |
| 971 | 0,0004151 | 0,00841 | 31,84 | 170,09 | 0,19 | [1436002_at](https://www.affymetrix.com/LinkServlet?probeset=1436002_at) | [Scube3](http://www.ncbi.nlm.nih.gov/entrez/query.fcgi?cmd=search&db=gene&term=Scube3) | signal peptide, CUB domain, EGF-like 3 |
| 972 | 0,0004174 | 0,00843 | 52,43 | 983,66 | 0,053 | [1417596_at](https://www.affymetrix.com/LinkServlet?probeset=1417596_at) | [B9d1](http://www.ncbi.nlm.nih.gov/entrez/query.fcgi?cmd=search&db=gene&term=B9d1) | B9 protein domain 1 |
| 973 | 0,0004174 | 0,00843 | 14,86 | 47,05 | 0,32 | [1439655_at](https://www.affymetrix.com/LinkServlet?probeset=1439655_at) | [NA](http://www.ncbi.nlm.nih.gov/entrez/query.fcgi?cmd=search&db=gene&term=NA) | NA |
| 974 | 0,0004184 | 0,00843 | 5495,71 | 7431,71 | 0,74 | [1416663_at](https://www.affymetrix.com/LinkServlet?probeset=1416663_at) | [Ndufa9](http://www.ncbi.nlm.nih.gov/entrez/query.fcgi?cmd=search&db=gene&term=Ndufa9) | NADH dehydrogenase (ubiquinone) 1 alpha subcomplex, 9 |
| 975 | 0,0004184 | 0,00843 | 2015,31 | 408,87 | 4,93 | [1448378_at](https://www.affymetrix.com/LinkServlet?probeset=1448378_at) | [Fscn1](http://www.ncbi.nlm.nih.gov/entrez/query.fcgi?cmd=search&db=gene&term=Fscn1) | fascin homolog 1, actin bundling protein (Strongylocentrotus purpuratus) |
| 976 | 0,0004191 | 0,00843 | 47,62 | 2018,85 | 0,024 | [1449036_at](https://www.affymetrix.com/LinkServlet?probeset=1449036_at) | [Rnf128](http://www.ncbi.nlm.nih.gov/entrez/query.fcgi?cmd=search&db=gene&term=Rnf128) | ring finger protein 128 |
| 977 | 0,0004198 | 0,00843 | 13,35 | 81,3 | 0,16 | [1436311_at](https://www.affymetrix.com/LinkServlet?probeset=1436311_at) | [Gemin5](http://www.ncbi.nlm.nih.gov/entrez/query.fcgi?cmd=search&db=gene&term=Gemin5) | gem (nuclear organelle) associated protein 5 |
| 978 | 0,0004199 | 0,00843 | 34,84 | 99,46 | 0,35 | [1441331_at](https://www.affymetrix.com/LinkServlet?probeset=1441331_at) | [A230061C15Rik](http://www.ncbi.nlm.nih.gov/entrez/query.fcgi?cmd=search&db=gene&term=A230061C15Rik) | RIKEN cDNA A230061C15 gene |
| 979 | 0,0004201 | 0,00843 | 193,4 | 7,12 | 27,16 | [1421890_at](https://www.affymetrix.com/LinkServlet?probeset=1421890_at) | [St3gal2](http://www.ncbi.nlm.nih.gov/entrez/query.fcgi?cmd=search&db=gene&term=St3gal2) | ST3 beta-galactoside alpha-2,3-sialyltransferase 2 |
| 980 | 0,0004205 | 0,00843 | 578,73 | 1926,8 | 0,3 | [1415728_at](https://www.affymetrix.com/LinkServlet?probeset=1415728_at) | [Pabpn1](http://www.ncbi.nlm.nih.gov/entrez/query.fcgi?cmd=search&db=gene&term=Pabpn1) | poly(A) binding protein, nuclear 1 |
| 981 | 0,0004209 | 0,00843 | 8,65 | 57,46 | 0,15 | [1436324_at](https://www.affymetrix.com/LinkServlet?probeset=1436324_at) | [Stard9](http://www.ncbi.nlm.nih.gov/entrez/query.fcgi?cmd=search&db=gene&term=Stard9) | START domain containing 9 |
| 982 | 0,0004212 | 0,00843 | 727,67 | 1197,91 | 0,61 | [1417786_a_at](https://www.affymetrix.com/LinkServlet?probeset=1417786_a_at) | [Rgs19](http://www.ncbi.nlm.nih.gov/entrez/query.fcgi?cmd=search&db=gene&term=Rgs19) | regulator of G-protein signaling 19 |
| 983 | 0,0004226 | 0,00843 | 848,05 | 88,87 | 9,54 | [1452057_at](https://www.affymetrix.com/LinkServlet?probeset=1452057_at) | [Actr1b](http://www.ncbi.nlm.nih.gov/entrez/query.fcgi?cmd=search&db=gene&term=Actr1b) | ARP1 actin-related protein 1 homolog B, centractin beta (yeast) |
| 984 | 0,0004228 | 0,00843 | 1138,78 | 107,29 | 10,61 | [1460034_at](https://www.affymetrix.com/LinkServlet?probeset=1460034_at) | [Samd4b](http://www.ncbi.nlm.nih.gov/entrez/query.fcgi?cmd=search&db=gene&term=Samd4b) | sterile alpha motif domain containing 4B |
| 985 | 0,0004229 | 0,00843 | 1899,71 | 3344,34 | 0,57 | [1436121_a_at](https://www.affymetrix.com/LinkServlet?probeset=1436121_a_at) | [Nsmce1](http://www.ncbi.nlm.nih.gov/entrez/query.fcgi?cmd=search&db=gene&term=Nsmce1) | non-SMC element 1 homolog (S. cerevisiae) |
| 986 | 0,000423 | 0,00843 | 31,75 | 89,95 | 0,35 | [1430145_at](https://www.affymetrix.com/LinkServlet?probeset=1430145_at) | [Lrrc28](http://www.ncbi.nlm.nih.gov/entrez/query.fcgi?cmd=search&db=gene&term=Lrrc28) | leucine rich repeat containing 28 |
| 987 | 0,0004233 | 0,00843 | 12,44 | 70,96 | 0,18 | [1450961_a_at](https://www.affymetrix.com/LinkServlet?probeset=1450961_a_at) | [NA](http://www.ncbi.nlm.nih.gov/entrez/query.fcgi?cmd=search&db=gene&term=NA) | NA |
| 988 | 0,0004237 | 0,00843 | 1338,63 | 584,95 | 2,29 | [1422210_at](https://www.affymetrix.com/LinkServlet?probeset=1422210_at) | [Foxd3](http://www.ncbi.nlm.nih.gov/entrez/query.fcgi?cmd=search&db=gene&term=Foxd3) | forkhead box D3 |
| 989 | 0,0004238 | 0,00843 | 21,25 | 89,8 | 0,24 | [1446838_at](https://www.affymetrix.com/LinkServlet?probeset=1446838_at) | [Atad1](http://www.ncbi.nlm.nih.gov/entrez/query.fcgi?cmd=search&db=gene&term=Atad1) | ATPase family, AAA domain containing 1 |
| 990 | 0,0004252 | 0,00844 | 33,99 | 10,35 | 3,29 | [1416016_at](https://www.affymetrix.com/LinkServlet?probeset=1416016_at) | [NA](http://www.ncbi.nlm.nih.gov/entrez/query.fcgi?cmd=search&db=gene&term=NA) | NA |
| 991 | 0,0004253 | 0,00844 | 593,58 | 101,95 | 5,82 | [1455295_at](https://www.affymetrix.com/LinkServlet?probeset=1455295_at) | [Slc38a7](http://www.ncbi.nlm.nih.gov/entrez/query.fcgi?cmd=search&db=gene&term=Slc38a7) | solute carrier family 38, member 7 |
| 992 | 0,0004266 | 0,00845 | 1670,16 | 2665,35 | 0,63 | [1417091_at](https://www.affymetrix.com/LinkServlet?probeset=1417091_at) | [Chuk](http://www.ncbi.nlm.nih.gov/entrez/query.fcgi?cmd=search&db=gene&term=Chuk) | conserved helix-loop-helix ubiquitous kinase |
| 993 | 0,0004266 | 0,00845 | 448,96 | 1290,25 | 0,35 | [1434666_at](https://www.affymetrix.com/LinkServlet?probeset=1434666_at) | [NA](http://www.ncbi.nlm.nih.gov/entrez/query.fcgi?cmd=search&db=gene&term=NA) | NA |
| 994 | 0,00043 | 0,00851 | 1193,31 | 266,62 | 4,48 | [1419066_at](https://www.affymetrix.com/LinkServlet?probeset=1419066_at) | [Ier5l](http://www.ncbi.nlm.nih.gov/entrez/query.fcgi?cmd=search&db=gene&term=Ier5l) | immediate early response 5-like |
| 995 | 0,0004314 | 0,00853 | 11,36 | 238,61 | 0,048 | [1458813_at](https://www.affymetrix.com/LinkServlet?probeset=1458813_at) | [Scn5a](http://www.ncbi.nlm.nih.gov/entrez/query.fcgi?cmd=search&db=gene&term=Scn5a) | sodium channel, voltage-gated, type V, alpha |
| 996 | 0,000434 | 0,00855 | 1174,44 | 2306,39 | 0,51 | [1430133_at](https://www.affymetrix.com/LinkServlet?probeset=1430133_at) | [Tbc1d8b](http://www.ncbi.nlm.nih.gov/entrez/query.fcgi?cmd=search&db=gene&term=Tbc1d8b) | TBC1 domain family, member 8B |
| 997 | 0,0004345 | 0,00855 | 576,04 | 1092,56 | 0,53 | [1428320_at](https://www.affymetrix.com/LinkServlet?probeset=1428320_at) | [Kdm3b](http://www.ncbi.nlm.nih.gov/entrez/query.fcgi?cmd=search&db=gene&term=Kdm3b) | KDM3B lysine (K)-specific demethylase 3B |
| 998 | 0,0004345 | 0,00855 | 447,41 | 1734,37 | 0,26 | [1454889_x_at](https://www.affymetrix.com/LinkServlet?probeset=1454889_x_at) | [Tmcc3](http://www.ncbi.nlm.nih.gov/entrez/query.fcgi?cmd=search&db=gene&term=Tmcc3) | transmembrane and coiled coil domains 3 |
| 999 | 0,0004345 | 0,00855 | 1476,48 | 2212,42 | 0,67 | [1454899_at](https://www.affymetrix.com/LinkServlet?probeset=1454899_at) | [Lpp](http://www.ncbi.nlm.nih.gov/entrez/query.fcgi?cmd=search&db=gene&term=Lpp) | LIM domain containing preferred translocation partner in lipoma |
| 1000 | 0,0004367 | 0,00859 | 9,21 | 58,77 | 0,16 | [1426389_at](https://www.affymetrix.com/LinkServlet?probeset=1426389_at) | [Camk1d](http://www.ncbi.nlm.nih.gov/entrez/query.fcgi?cmd=search&db=gene&term=Camk1d) | calcium/calmodulin-dependent protein kinase ID |
| 1001 | 0,0004377 | 0,00859 | 44,9 | 110,68 | 0,41 | [1428149_at](https://www.affymetrix.com/LinkServlet?probeset=1428149_at) | [Coro7](http://www.ncbi.nlm.nih.gov/entrez/query.fcgi?cmd=search&db=gene&term=Coro7) | coronin 7 |
| 1002 | 0,0004377 | 0,00859 | 2085,73 | 3962,9 | 0,53 | [1424141_at](https://www.affymetrix.com/LinkServlet?probeset=1424141_at) | [Hectd1](http://www.ncbi.nlm.nih.gov/entrez/query.fcgi?cmd=search&db=gene&term=Hectd1) | HECT domain containing 1 |
| 1003 | 0,0004383 | 0,00859 | 104,71 | 356,11 | 0,29 | [1451779_at](https://www.affymetrix.com/LinkServlet?probeset=1451779_at) | [Pyroxd1](http://www.ncbi.nlm.nih.gov/entrez/query.fcgi?cmd=search&db=gene&term=Pyroxd1) | pyridine nucleotide-disulphide oxidoreductase domain 1 |
| 1004 | 0,000439 | 0,0086 | 242,56 | 1643,21 | 0,15 | [1425019_at](https://www.affymetrix.com/LinkServlet?probeset=1425019_at) | [Ubxn2a](http://www.ncbi.nlm.nih.gov/entrez/query.fcgi?cmd=search&db=gene&term=Ubxn2a) | UBX domain protein 2A |
| 1005 | 0,000444 | 0,00869 | 41,45 | 168,04 | 0,25 | [1423357_at](https://www.affymetrix.com/LinkServlet?probeset=1423357_at) | [Lipt2](http://www.ncbi.nlm.nih.gov/entrez/query.fcgi?cmd=search&db=gene&term=Lipt2) | lipoyl(octanoyl) transferase 2 (putative) |
| 1006 | 0,0004461 | 0,00872 | 11582,54 | 7524,53 | 1,54 | [1437984_x_at](https://www.affymetrix.com/LinkServlet?probeset=1437984_x_at) | [Ddx39b](http://www.ncbi.nlm.nih.gov/entrez/query.fcgi?cmd=search&db=gene&term=Ddx39b) | DEAD (Asp-Glu-Ala-Asp) box polypeptide 39B |
| 1007 | 0,000447 | 0,00873 | 3027,37 | 5459,09 | 0,55 | [1424099_at](https://www.affymetrix.com/LinkServlet?probeset=1424099_at) | [Gpx8](http://www.ncbi.nlm.nih.gov/entrez/query.fcgi?cmd=search&db=gene&term=Gpx8) | glutathione peroxidase 8 (putative) |
| 1008 | 0,0004498 | 0,00874 | 3361,81 | 2005,14 | 1,68 | [1421867_at](https://www.affymetrix.com/LinkServlet?probeset=1421867_at) | [Nr3c1](http://www.ncbi.nlm.nih.gov/entrez/query.fcgi?cmd=search&db=gene&term=Nr3c1) | nuclear receptor subfamily 3, group C, member 1 |
| 1009 | 0,0004504 | 0,00874 | 7,59 | 30,41 | 0,25 | [1418595_at](https://www.affymetrix.com/LinkServlet?probeset=1418595_at) | [Plin4](http://www.ncbi.nlm.nih.gov/entrez/query.fcgi?cmd=search&db=gene&term=Plin4) | perilipin 4 |
| 1010 | 0,0004505 | 0,00874 | 3942,2 | 2024,08 | 1,95 | [1434604_at](https://www.affymetrix.com/LinkServlet?probeset=1434604_at) | [Eif5b](http://www.ncbi.nlm.nih.gov/entrez/query.fcgi?cmd=search&db=gene&term=Eif5b) | eukaryotic translation initiation factor 5B |
| 1011 | 0,0004508 | 0,00874 | 478,65 | 3719,78 | 0,13 | [1427742_a_at](https://www.affymetrix.com/LinkServlet?probeset=1427742_a_at) | [Klf6](http://www.ncbi.nlm.nih.gov/entrez/query.fcgi?cmd=search&db=gene&term=Klf6) | Kruppel-like factor 6 |
| 1012 | 0,0004509 | 0,00874 | 283,63 | 37,16 | 7,63 | [1454906_at](https://www.affymetrix.com/LinkServlet?probeset=1454906_at) | [Rarb](http://www.ncbi.nlm.nih.gov/entrez/query.fcgi?cmd=search&db=gene&term=Rarb) | retinoic acid receptor, beta |
| 1013 | 0,000451 | 0,00874 | 603,51 | 189,34 | 3,19 | [1436596_at](https://www.affymetrix.com/LinkServlet?probeset=1436596_at) | [H2afv](http://www.ncbi.nlm.nih.gov/entrez/query.fcgi?cmd=search&db=gene&term=H2afv) | H2A histone family, member V |
| 1014 | 0,000452 | 0,00874 | 16,48 | 939,26 | 0,018 | [1428670_at](https://www.affymetrix.com/LinkServlet?probeset=1428670_at) | [2610305J24Rik](http://www.ncbi.nlm.nih.gov/entrez/query.fcgi?cmd=search&db=gene&term=2610305J24Rik) | RIKEN cDNA 2610305J24 gene |
| 1015 | 0,0004522 | 0,00874 | 1434,01 | 495,22 | 2,9 | [1430514_a_at](https://www.affymetrix.com/LinkServlet?probeset=1430514_a_at) | [Cd99](http://www.ncbi.nlm.nih.gov/entrez/query.fcgi?cmd=search&db=gene&term=Cd99) | CD99 antigen |
| 1016 | 0,0004523 | 0,00874 | 327,68 | 29,97 | 10,93 | [1426596_a_at](https://www.affymetrix.com/LinkServlet?probeset=1426596_a_at) | [Smn1](http://www.ncbi.nlm.nih.gov/entrez/query.fcgi?cmd=search&db=gene&term=Smn1) | survival motor neuron 1 |
| 1017 | 0,0004528 | 0,00874 | 453,16 | 1039,69 | 0,44 | [1416568_a_at](https://www.affymetrix.com/LinkServlet?probeset=1416568_a_at) | [Acin1](http://www.ncbi.nlm.nih.gov/entrez/query.fcgi?cmd=search&db=gene&term=Acin1) | apoptotic chromatin condensation inducer 1 |
| 1018 | 0,0004531 | 0,00874 | 4300,88 | 9184,47 | 0,47 | [1454862_at](https://www.affymetrix.com/LinkServlet?probeset=1454862_at) | [Phldb2](http://www.ncbi.nlm.nih.gov/entrez/query.fcgi?cmd=search&db=gene&term=Phldb2) | pleckstrin homology-like domain, family B, member 2 |
| 1019 | 0,0004531 | 0,00874 | 721,53 | 2390 | 0,3 | [1419568_at](https://www.affymetrix.com/LinkServlet?probeset=1419568_at) | [Mapk1](http://www.ncbi.nlm.nih.gov/entrez/query.fcgi?cmd=search&db=gene&term=Mapk1) | mitogen-activated protein kinase 1 |
| 1020 | 0,0004536 | 0,00875 | 202,69 | 768,77 | 0,26 | [1448780_at](https://www.affymetrix.com/LinkServlet?probeset=1448780_at) | [Slc12a2](http://www.ncbi.nlm.nih.gov/entrez/query.fcgi?cmd=search&db=gene&term=Slc12a2) | solute carrier family 12, member 2 |
| 1021 | 0,0004543 | 0,00875 | 6,02 | 143,86 | 0,042 | [1443924_at](https://www.affymetrix.com/LinkServlet?probeset=1443924_at) | [Wnk3-ps](http://www.ncbi.nlm.nih.gov/entrez/query.fcgi?cmd=search&db=gene&term=Wnk3-ps) | WNK lysine deficient protein kinase 3, pseudogene |
| 1022 | 0,0004548 | 0,00875 | 4,75 | 18,93 | 0,25 | [1443527_at](https://www.affymetrix.com/LinkServlet?probeset=1443527_at) | [Terf1](http://www.ncbi.nlm.nih.gov/entrez/query.fcgi?cmd=search&db=gene&term=Terf1) | telomeric repeat binding factor 1 |
| 1023 | 0,0004563 | 0,00877 | 347,76 | 877,64 | 0,4 | [1451040_at](https://www.affymetrix.com/LinkServlet?probeset=1451040_at) | [Dtd1](http://www.ncbi.nlm.nih.gov/entrez/query.fcgi?cmd=search&db=gene&term=Dtd1) | D-tyrosyl-tRNA deacylase 1 homolog (S. cerevisiae) |
| 1024 | 0,0004569 | 0,00878 | 300,94 | 1162,47 | 0,26 | [1434657_at](https://www.affymetrix.com/LinkServlet?probeset=1434657_at) | [Gls](http://www.ncbi.nlm.nih.gov/entrez/query.fcgi?cmd=search&db=gene&term=Gls) | glutaminase |
| 1025 | 0,0004579 | 0,00879 | 20,7 | 146,02 | 0,14 | [1444244_at](https://www.affymetrix.com/LinkServlet?probeset=1444244_at) | [NA](http://www.ncbi.nlm.nih.gov/entrez/query.fcgi?cmd=search&db=gene&term=NA) | NA |
| 1026 | 0,0004604 | 0,00882 | 26,79 | 7,58 | 3,54 | [1426508_at](https://www.affymetrix.com/LinkServlet?probeset=1426508_at) | [Gfap](http://www.ncbi.nlm.nih.gov/entrez/query.fcgi?cmd=search&db=gene&term=Gfap) | glial fibrillary acidic protein |
| 1027 | 0,0004605 | 0,00882 | 673,94 | 286,79 | 2,35 | [1428110_x_at](https://www.affymetrix.com/LinkServlet?probeset=1428110_x_at) | [Vps11](http://www.ncbi.nlm.nih.gov/entrez/query.fcgi?cmd=search&db=gene&term=Vps11) | vacuolar protein sorting 11 (yeast) |
| 1028 | 0,000461 | 0,00882 | 62,08 | 205,26 | 0,3 | [1418744_s_at](https://www.affymetrix.com/LinkServlet?probeset=1418744_s_at) | [Tesc](http://www.ncbi.nlm.nih.gov/entrez/query.fcgi?cmd=search&db=gene&term=Tesc) | tescalcin |
| 1029 | 0,0004623 | 0,00884 | 120,1 | 549,03 | 0,22 | [1426288_at](https://www.affymetrix.com/LinkServlet?probeset=1426288_at) | [Lrp4](http://www.ncbi.nlm.nih.gov/entrez/query.fcgi?cmd=search&db=gene&term=Lrp4) | low density lipoprotein receptor-related protein 4 |
| 1030 | 0,0004628 | 0,00884 | 128,78 | 10,2 | 12,63 | [1438140_a_at](https://www.affymetrix.com/LinkServlet?probeset=1438140_a_at) | [Zfp64](http://www.ncbi.nlm.nih.gov/entrez/query.fcgi?cmd=search&db=gene&term=Zfp64) | zinc finger protein 64 |
| 1031 | 0,0004636 | 0,00884 | 1107,65 | 453,75 | 2,44 | [1421708_a_at](https://www.affymetrix.com/LinkServlet?probeset=1421708_a_at) | [Stat6](http://www.ncbi.nlm.nih.gov/entrez/query.fcgi?cmd=search&db=gene&term=Stat6) | signal transducer and activator of transcription 6 |
| 1032 | 0,0004643 | 0,00885 | 11,37 | 26,9 | 0,42 | [1437914_at](https://www.affymetrix.com/LinkServlet?probeset=1437914_at) | [E2f6](http://www.ncbi.nlm.nih.gov/entrez/query.fcgi?cmd=search&db=gene&term=E2f6) | E2F transcription factor 6 |
| 1033 | 0,000466 | 0,00887 | 51,37 | 879,57 | 0,058 | [1448898_at](https://www.affymetrix.com/LinkServlet?probeset=1448898_at) | [Ccl9](http://www.ncbi.nlm.nih.gov/entrez/query.fcgi?cmd=search&db=gene&term=Ccl9) | chemokine (C-C motif) ligand 9 |
| 1034 | 0,0004667 | 0,00888 | 41,69 | 5,62 | 7,42 | [1436743_at](https://www.affymetrix.com/LinkServlet?probeset=1436743_at) | [Cadm2](http://www.ncbi.nlm.nih.gov/entrez/query.fcgi?cmd=search&db=gene&term=Cadm2) | cell adhesion molecule 2 |
| 1035 | 0,0004691 | 0,0089 | 14,92 | 603,44 | 0,025 | [1443832_s_at](https://www.affymetrix.com/LinkServlet?probeset=1443832_s_at) | [Sdpr](http://www.ncbi.nlm.nih.gov/entrez/query.fcgi?cmd=search&db=gene&term=Sdpr) | serum deprivation response |
| 1036 | 0,0004693 | 0,0089 | 34,19 | 85,49 | 0,4 | [1434101_at](https://www.affymetrix.com/LinkServlet?probeset=1434101_at) | [Nfib](http://www.ncbi.nlm.nih.gov/entrez/query.fcgi?cmd=search&db=gene&term=Nfib) | nuclear factor I/B |
| 1037 | 0,0004695 | 0,0089 | 240,61 | 7011,74 | 0,034 | [1415800_at](https://www.affymetrix.com/LinkServlet?probeset=1415800_at) | [Gja1](http://www.ncbi.nlm.nih.gov/entrez/query.fcgi?cmd=search&db=gene&term=Gja1) | gap junction protein, alpha 1 |
| 1038 | 0,0004714 | 0,00893 | 1319,66 | 2893,97 | 0,46 | [1434225_at](https://www.affymetrix.com/LinkServlet?probeset=1434225_at) | [Swap70](http://www.ncbi.nlm.nih.gov/entrez/query.fcgi?cmd=search&db=gene&term=Swap70) | SWA-70 protein |
| 1039 | 0,000472 | 0,00893 | 1126,74 | 635,74 | 1,77 | [1424460_s_at](https://www.affymetrix.com/LinkServlet?probeset=1424460_s_at) | [Lpcat1](http://www.ncbi.nlm.nih.gov/entrez/query.fcgi?cmd=search&db=gene&term=Lpcat1) | lysophosphatidylcholine acyltransferase 1 |
| 1040 | 0,0004737 | 0,00895 | 1188 | 415,69 | 2,86 | [1430600_at](https://www.affymetrix.com/LinkServlet?probeset=1430600_at) | [Cmtm5](http://www.ncbi.nlm.nih.gov/entrez/query.fcgi?cmd=search&db=gene&term=Cmtm5) | CKLF-like MARVEL transmembrane domain containing 5 |
| 1041 | 0,0004739 | 0,00895 | 2238,66 | 773,23 | 2,9 | [1427048_at](https://www.affymetrix.com/LinkServlet?probeset=1427048_at) | [Smo](http://www.ncbi.nlm.nih.gov/entrez/query.fcgi?cmd=search&db=gene&term=Smo) | smoothened homolog (Drosophila) |
| 1042 | 0,0004746 | 0,00895 | 4539,6 | 975,6 | 4,65 | [1420953_at](https://www.affymetrix.com/LinkServlet?probeset=1420953_at) | [Add1](http://www.ncbi.nlm.nih.gov/entrez/query.fcgi?cmd=search&db=gene&term=Add1) | adducin 1 (alpha) |
| 1043 | 0,0004749 | 0,00895 | 16,01 | 161,99 | 0,099 | [1417756_a_at](https://www.affymetrix.com/LinkServlet?probeset=1417756_a_at) | [Lsp1](http://www.ncbi.nlm.nih.gov/entrez/query.fcgi?cmd=search&db=gene&term=Lsp1) | lymphocyte specific 1 |
| 1044 | 0,0004764 | 0,00897 | 1812,51 | 3229,36 | 0,56 | [1428796_at](https://www.affymetrix.com/LinkServlet?probeset=1428796_at) | [Bbx](http://www.ncbi.nlm.nih.gov/entrez/query.fcgi?cmd=search&db=gene&term=Bbx) | bobby sox homolog (Drosophila) |
| 1045 | 0,0004772 | 0,00898 | 2984,15 | 1446,11 | 2,06 | [1415739_at](https://www.affymetrix.com/LinkServlet?probeset=1415739_at) | [Rbm42](http://www.ncbi.nlm.nih.gov/entrez/query.fcgi?cmd=search&db=gene&term=Rbm42) | RNA binding motif protein 42 |
| 1046 | 0,0004805 | 0,00903 | 9847,61 | 2464,66 | 4 | [1419258_at](https://www.affymetrix.com/LinkServlet?probeset=1419258_at) | [Tcea1](http://www.ncbi.nlm.nih.gov/entrez/query.fcgi?cmd=search&db=gene&term=Tcea1) | transcription elongation factor A (SII) 1 |
| 1047 | 0,0004808 | 0,00903 | 68,88 | 6,32 | 10,9 | [1438479_at](https://www.affymetrix.com/LinkServlet?probeset=1438479_at) | [Zfp213](http://www.ncbi.nlm.nih.gov/entrez/query.fcgi?cmd=search&db=gene&term=Zfp213) | zinc finger protein 213 |
| 1048 | 0,0004812 | 0,00903 | 26,75 | 509,95 | 0,052 | [1419639_at](https://www.affymetrix.com/LinkServlet?probeset=1419639_at) | [Efnb2](http://www.ncbi.nlm.nih.gov/entrez/query.fcgi?cmd=search&db=gene&term=Efnb2) | ephrin B2 |
| 1049 | 0,0004814 | 0,00903 | 8080,2 | 4480,81 | 1,8 | [1451237_s_at](https://www.affymetrix.com/LinkServlet?probeset=1451237_s_at) | [Rbm7](http://www.ncbi.nlm.nih.gov/entrez/query.fcgi?cmd=search&db=gene&term=Rbm7) | RNA binding motif protein 7 |
| 1050 | 0,0004827 | 0,00903 | 1208,32 | 753,39 | 1,6 | [1460644_at](https://www.affymetrix.com/LinkServlet?probeset=1460644_at) | [Bckdk](http://www.ncbi.nlm.nih.gov/entrez/query.fcgi?cmd=search&db=gene&term=Bckdk) | branched chain ketoacid dehydrogenase kinase |
| 1051 | 0,0004828 | 0,00903 | 8293,05 | 11351,55 | 0,73 | [1439375_x_at](https://www.affymetrix.com/LinkServlet?probeset=1439375_x_at) | [Aldoa](http://www.ncbi.nlm.nih.gov/entrez/query.fcgi?cmd=search&db=gene&term=Aldoa) | aldolase A, fructose-bisphosphate |
| 1052 | 0,0004828 | 0,00903 | 68,16 | 168,05 | 0,41 | [1426908_at](https://www.affymetrix.com/LinkServlet?probeset=1426908_at) | [Galnt7](http://www.ncbi.nlm.nih.gov/entrez/query.fcgi?cmd=search&db=gene&term=Galnt7) | UDP-N-acetyl-alpha-D-galactosamine: polypeptide N-acetylgalactosaminyltransferase 7 |
| 1053 | 0,0004837 | 0,00903 | 180,14 | 722,13 | 0,25 | [1427978_at](https://www.affymetrix.com/LinkServlet?probeset=1427978_at) | [4732418C07Rik](http://www.ncbi.nlm.nih.gov/entrez/query.fcgi?cmd=search&db=gene&term=4732418C07Rik) | RIKEN cDNA 4732418C07 gene |
| 1054 | 0,0004842 | 0,00903 | 348,31 | 2113,1 | 0,16 | [1417373_a_at](https://www.affymetrix.com/LinkServlet?probeset=1417373_a_at) | [Tuba4a](http://www.ncbi.nlm.nih.gov/entrez/query.fcgi?cmd=search&db=gene&term=Tuba4a) | tubulin, alpha 4A |
| 1055 | 0,000485 | 0,00904 | 2953,39 | 1318,19 | 2,24 | [1448569_at](https://www.affymetrix.com/LinkServlet?probeset=1448569_at) | [Mlec](http://www.ncbi.nlm.nih.gov/entrez/query.fcgi?cmd=search&db=gene&term=Mlec) | malectin |
| 1056 | 0,000486 | 0,00905 | 6,78 | 39,95 | 0,17 | [1438721_a_at](https://www.affymetrix.com/LinkServlet?probeset=1438721_a_at) | [Irf3](http://www.ncbi.nlm.nih.gov/entrez/query.fcgi?cmd=search&db=gene&term=Irf3) | interferon regulatory factor 3 |
| 1057 | 0,000487 | 0,00906 | 116,54 | 557,47 | 0,21 | [1427226_at](https://www.affymetrix.com/LinkServlet?probeset=1427226_at) | [Epn2](http://www.ncbi.nlm.nih.gov/entrez/query.fcgi?cmd=search&db=gene&term=Epn2) | epsin 2 |
| 1058 | 0,000488 | 0,00907 | 313,32 | 615,5 | 0,51 | [1424244_at](https://www.affymetrix.com/LinkServlet?probeset=1424244_at) | [Rwdd4a](http://www.ncbi.nlm.nih.gov/entrez/query.fcgi?cmd=search&db=gene&term=Rwdd4a) | RWD domain containing 4A |
| 1059 | 0,0004894 | 0,00908 | 3057,66 | 1570,18 | 1,95 | [1452669_at](https://www.affymetrix.com/LinkServlet?probeset=1452669_at) | [Fam53c](http://www.ncbi.nlm.nih.gov/entrez/query.fcgi?cmd=search&db=gene&term=Fam53c) | family with sequence similarity 53, member C |
| 1060 | 0,0004897 | 0,00908 | 266,13 | 538,32 | 0,49 | [1415791_at](https://www.affymetrix.com/LinkServlet?probeset=1415791_at) | [Rnf34](http://www.ncbi.nlm.nih.gov/entrez/query.fcgi?cmd=search&db=gene&term=Rnf34) | ring finger protein 34 |
| 1061 | 0,0004899 | 0,00908 | 72,43 | 6,32 | 11,45 | [1428462_at](https://www.affymetrix.com/LinkServlet?probeset=1428462_at) | [Ppp2r5e](http://www.ncbi.nlm.nih.gov/entrez/query.fcgi?cmd=search&db=gene&term=Ppp2r5e) | protein phosphatase 2, regulatory subunit B (B56), epsilon isoform |
| 1062 | 0,0004901 | 0,00908 | 3823,16 | 9961,11 | 0,38 | [1439459_x_at](https://www.affymetrix.com/LinkServlet?probeset=1439459_x_at) | [Acly](http://www.ncbi.nlm.nih.gov/entrez/query.fcgi?cmd=search&db=gene&term=Acly) | ATP citrate lyase |
| 1063 | 0,0004908 | 0,00908 | 14755,44 | 11146,76 | 1,32 | [1424707_at](https://www.affymetrix.com/LinkServlet?probeset=1424707_at) | [Tmed10](http://www.ncbi.nlm.nih.gov/entrez/query.fcgi?cmd=search&db=gene&term=Tmed10) | transmembrane emp24-like trafficking protein 10 (yeast) |
| 1064 | 0,0004911 | 0,00908 | 1238,99 | 3775,51 | 0,33 | [1429564_at](https://www.affymetrix.com/LinkServlet?probeset=1429564_at) | [Pcgf5](http://www.ncbi.nlm.nih.gov/entrez/query.fcgi?cmd=search&db=gene&term=Pcgf5) | polycomb group ring finger 5 |
| 1065 | 0,0004919 | 0,00908 | 299,92 | 693,33 | 0,43 | [1423386_at](https://www.affymetrix.com/LinkServlet?probeset=1423386_at) | [Psmd9](http://www.ncbi.nlm.nih.gov/entrez/query.fcgi?cmd=search&db=gene&term=Psmd9) | proteasome (prosome, macropain) 26S subunit, non-ATPase, 9 |
| 1066 | 0,0004935 | 0,00909 | 23,08 | 7,9 | 2,92 | [1434110_x_at](https://www.affymetrix.com/LinkServlet?probeset=1434110_x_at) | [NA](http://www.ncbi.nlm.nih.gov/entrez/query.fcgi?cmd=search&db=gene&term=NA) | NA |
| 1067 | 0,0004938 | 0,00909 | 11,97 | 77,21 | 0,16 | [1438131_at](https://www.affymetrix.com/LinkServlet?probeset=1438131_at) | [Fbxw2](http://www.ncbi.nlm.nih.gov/entrez/query.fcgi?cmd=search&db=gene&term=Fbxw2) | F-box and WD-40 domain protein 2 |
| 1068 | 0,0004938 | 0,00909 | 1229,65 | 2258,56 | 0,54 | [1428073_a_at](https://www.affymetrix.com/LinkServlet?probeset=1428073_a_at) | [Nup88](http://www.ncbi.nlm.nih.gov/entrez/query.fcgi?cmd=search&db=gene&term=Nup88) | nucleoporin 88 |
| 1069 | 0,0004963 | 0,00913 | 12,55 | 57,59 | 0,22 | [1421005_at](https://www.affymetrix.com/LinkServlet?probeset=1421005_at) | [Cep110](http://www.ncbi.nlm.nih.gov/entrez/query.fcgi?cmd=search&db=gene&term=Cep110) | centrosomal protein 110 |
| 1070 | 0,0004965 | 0,00913 | 909,34 | 138,61 | 6,56 | [1427164_at](https://www.affymetrix.com/LinkServlet?probeset=1427164_at) | [Il13ra1](http://www.ncbi.nlm.nih.gov/entrez/query.fcgi?cmd=search&db=gene&term=Il13ra1) | interleukin 13 receptor, alpha 1 |
| 1071 | 0,0004972 | 0,00913 | 371,33 | 7,96 | 46,63 | [1421241_at](https://www.affymetrix.com/LinkServlet?probeset=1421241_at) | [Ngfr](http://www.ncbi.nlm.nih.gov/entrez/query.fcgi?cmd=search&db=gene&term=Ngfr) | nerve growth factor receptor (TNFR superfamily, member 16) |
| 1072 | 0,0004984 | 0,00914 | 39,03 | 10,21 | 3,82 | [1421160_a_at](https://www.affymetrix.com/LinkServlet?probeset=1421160_a_at) | [Rfng](http://www.ncbi.nlm.nih.gov/entrez/query.fcgi?cmd=search&db=gene&term=Rfng) | RFNG O-fucosylpeptide 3-beta-N-acetylglucosaminyltransferase |
| 1073 | 0,0004986 | 0,00914 | 7115,9 | 16391,97 | 0,43 | [1422706_at](https://www.affymetrix.com/LinkServlet?probeset=1422706_at) | [Pmepa1](http://www.ncbi.nlm.nih.gov/entrez/query.fcgi?cmd=search&db=gene&term=Pmepa1) | prostate transmembrane protein, androgen induced 1 |
| 1074 | 0,0004995 | 0,00915 | 114,47 | 7,95 | 14,39 | [1438220_at](https://www.affymetrix.com/LinkServlet?probeset=1438220_at) | [Foxj3](http://www.ncbi.nlm.nih.gov/entrez/query.fcgi?cmd=search&db=gene&term=Foxj3) | forkhead box J3 |
| 1075 | 0,0005018 | 0,00917 | 1282,45 | 749,47 | 1,71 | [1421276_a_at](https://www.affymetrix.com/LinkServlet?probeset=1421276_a_at) | [Dst](http://www.ncbi.nlm.nih.gov/entrez/query.fcgi?cmd=search&db=gene&term=Dst) | dystonin |
| 1076 | 0,0005018 | 0,00917 | 4,54 | 62,04 | 0,073 | [1429443_at](https://www.affymetrix.com/LinkServlet?probeset=1429443_at) | [Cpne4](http://www.ncbi.nlm.nih.gov/entrez/query.fcgi?cmd=search&db=gene&term=Cpne4) | copine IV |
| 1077 | 0,0005033 | 0,00919 | 1170,47 | 8123,73 | 0,14 | [1418431_at](https://www.affymetrix.com/LinkServlet?probeset=1418431_at) | [Kif5b](http://www.ncbi.nlm.nih.gov/entrez/query.fcgi?cmd=search&db=gene&term=Kif5b) | kinesin family member 5B |
| 1078 | 0,0005043 | 0,0092 | 44,04 | 130 | 0,34 | [1429797_at](https://www.affymetrix.com/LinkServlet?probeset=1429797_at) | [Atl3](http://www.ncbi.nlm.nih.gov/entrez/query.fcgi?cmd=search&db=gene&term=Atl3) | atlastin GTPase 3 |
| 1079 | 0,000505 | 0,0092 | 4,5 | 30,04 | 0,15 | [1431413_at](https://www.affymetrix.com/LinkServlet?probeset=1431413_at) | [Ramp1](http://www.ncbi.nlm.nih.gov/entrez/query.fcgi?cmd=search&db=gene&term=Ramp1) | receptor (calcitonin) activity modifying protein 1 |
| 1080 | 0,0005096 | 0,00928 | 1111,1 | 336,4 | 3,3 | [1437801_at](https://www.affymetrix.com/LinkServlet?probeset=1437801_at) | [Morf4l1](http://www.ncbi.nlm.nih.gov/entrez/query.fcgi?cmd=search&db=gene&term=Morf4l1) | mortality factor 4 like 1 |
| 1081 | 0,0005109 | 0,00929 | 922,83 | 2175,16 | 0,42 | [1435492_at](https://www.affymetrix.com/LinkServlet?probeset=1435492_at) | [Socs6](http://www.ncbi.nlm.nih.gov/entrez/query.fcgi?cmd=search&db=gene&term=Socs6) | suppressor of cytokine signaling 6 |
| 1082 | 0,0005119 | 0,0093 | 2840,56 | 307,57 | 9,24 | [1420653_at](https://www.affymetrix.com/LinkServlet?probeset=1420653_at) | [Tgfb1](http://www.ncbi.nlm.nih.gov/entrez/query.fcgi?cmd=search&db=gene&term=Tgfb1) | transforming growth factor, beta 1 |
| 1083 | 0,0005144 | 0,00934 | 109,59 | 205,63 | 0,53 | [1456908_at](https://www.affymetrix.com/LinkServlet?probeset=1456908_at) | [BC023202](http://www.ncbi.nlm.nih.gov/entrez/query.fcgi?cmd=search&db=gene&term=BC023202) | cDNA sequence BC023202 |
| 1084 | 0,0005166 | 0,00937 | 9,35 | 20,13 | 0,46 | [1440934_at](https://www.affymetrix.com/LinkServlet?probeset=1440934_at) | [6230409E13Rik](http://www.ncbi.nlm.nih.gov/entrez/query.fcgi?cmd=search&db=gene&term=6230409E13Rik) | RIKEN cDNA 6230409E13 gene |
| 1085 | 0,0005176 | 0,00937 | 5,8 | 46,13 | 0,13 | [1450853_at](https://www.affymetrix.com/LinkServlet?probeset=1450853_at) | [Tle4](http://www.ncbi.nlm.nih.gov/entrez/query.fcgi?cmd=search&db=gene&term=Tle4) | transducin-like enhancer of split 4, homolog of Drosophila E(spl) |
| 1086 | 0,0005176 | 0,00937 | 5271,14 | 7622,84 | 0,69 | [1448934_at](https://www.affymetrix.com/LinkServlet?probeset=1448934_at) | [Ndufa10](http://www.ncbi.nlm.nih.gov/entrez/query.fcgi?cmd=search&db=gene&term=Ndufa10) | NADH dehydrogenase (ubiquinone) 1 alpha subcomplex 10 |
| 1087 | 0,0005179 | 0,00937 | 252,55 | 463,44 | 0,54 | [1422870_at](https://www.affymetrix.com/LinkServlet?probeset=1422870_at) | [Hoxc4](http://www.ncbi.nlm.nih.gov/entrez/query.fcgi?cmd=search&db=gene&term=Hoxc4) | homeobox C4 |
| 1088 | 0,0005186 | 0,00937 | 5146,81 | 8350,96 | 0,62 | [1436992_x_at](https://www.affymetrix.com/LinkServlet?probeset=1436992_x_at) | [Vdac1](http://www.ncbi.nlm.nih.gov/entrez/query.fcgi?cmd=search&db=gene&term=Vdac1) | voltage-dependent anion channel 1 |
| 1089 | 0,000519 | 0,00937 | 165,99 | 329,68 | 0,5 | [1448996_at](https://www.affymetrix.com/LinkServlet?probeset=1448996_at) | [Rom1](http://www.ncbi.nlm.nih.gov/entrez/query.fcgi?cmd=search&db=gene&term=Rom1) | rod outer segment membrane protein 1 |
| 1090 | 0,0005198 | 0,00938 | 1903,22 | 648,83 | 2,93 | [1454727_at](https://www.affymetrix.com/LinkServlet?probeset=1454727_at) | [Afap1l1](http://www.ncbi.nlm.nih.gov/entrez/query.fcgi?cmd=search&db=gene&term=Afap1l1) | actin filament associated protein 1-like 1 |
| 1091 | 0,0005201 | 0,00938 | 823,19 | 1667,45 | 0,49 | [1418772_at](https://www.affymetrix.com/LinkServlet?probeset=1418772_at) | [BC016423](http://www.ncbi.nlm.nih.gov/entrez/query.fcgi?cmd=search&db=gene&term=BC016423) | cDNA sequence BC016423 |
| 1092 | 0,0005215 | 0,00939 | 1175,17 | 4331,07 | 0,27 | [1426523_a_at](https://www.affymetrix.com/LinkServlet?probeset=1426523_a_at) | [Gnpda2](http://www.ncbi.nlm.nih.gov/entrez/query.fcgi?cmd=search&db=gene&term=Gnpda2) | glucosamine-6-phosphate deaminase 2 |
| 1093 | 0,0005216 | 0,00939 | 1128,82 | 2694,09 | 0,42 | [1448400_a_at](https://www.affymetrix.com/LinkServlet?probeset=1448400_a_at) | [Smarcd2](http://www.ncbi.nlm.nih.gov/entrez/query.fcgi?cmd=search&db=gene&term=Smarcd2) | SWI/SNF related, matrix associated, actin dependent regulator of chromatin, subfamily d, member 2 |
| 1094 | 0,0005221 | 0,00939 | 15,12 | 104,88 | 0,14 | [1442407_at](https://www.affymetrix.com/LinkServlet?probeset=1442407_at) | [NA](http://www.ncbi.nlm.nih.gov/entrez/query.fcgi?cmd=search&db=gene&term=NA) | NA |
| 1095 | 0,0005235 | 0,0094 | 291,9 | 804,44 | 0,36 | [1451315_at](https://www.affymetrix.com/LinkServlet?probeset=1451315_at) | [Tmem101](http://www.ncbi.nlm.nih.gov/entrez/query.fcgi?cmd=search&db=gene&term=Tmem101) | transmembrane protein 101 |
| 1096 | 0,0005239 | 0,0094 | 397,88 | 83,36 | 4,77 | [1416955_at](https://www.affymetrix.com/LinkServlet?probeset=1416955_at) | [Slc25a10](http://www.ncbi.nlm.nih.gov/entrez/query.fcgi?cmd=search&db=gene&term=Slc25a10) | solute carrier family 25 (mitochondrial carrier, dicarboxylate transporter), member 10 |
| 1097 | 0,0005242 | 0,0094 | 3408,31 | 792,06 | 4,3 | [1448565_at](https://www.affymetrix.com/LinkServlet?probeset=1448565_at) | [Ppp1r11](http://www.ncbi.nlm.nih.gov/entrez/query.fcgi?cmd=search&db=gene&term=Ppp1r11) | protein phosphatase 1, regulatory (inhibitor) subunit 11 |
| 1098 | 0,0005254 | 0,00941 | 975,63 | 1709,91 | 0,57 | [1417753_at](https://www.affymetrix.com/LinkServlet?probeset=1417753_at) | [Pkd2](http://www.ncbi.nlm.nih.gov/entrez/query.fcgi?cmd=search&db=gene&term=Pkd2) | polycystic kidney disease 2 |
| 1099 | 0,000529 | 0,00947 | 398,72 | 1109,21 | 0,36 | [1455427_at](https://www.affymetrix.com/LinkServlet?probeset=1455427_at) | [Angpt4](http://www.ncbi.nlm.nih.gov/entrez/query.fcgi?cmd=search&db=gene&term=Angpt4) | angiopoietin 4 |
| 1100 | 0,0005316 | 0,0095 | 555,42 | 170,08 | 3,27 | [1416713_at](https://www.affymetrix.com/LinkServlet?probeset=1416713_at) | [Tppp3](http://www.ncbi.nlm.nih.gov/entrez/query.fcgi?cmd=search&db=gene&term=Tppp3) | tubulin polymerization-promoting protein family member 3 |
| 1101 | 0,0005324 | 0,00951 | 219,35 | 1503,08 | 0,15 | [1417847_at](https://www.affymetrix.com/LinkServlet?probeset=1417847_at) | [Ulk2](http://www.ncbi.nlm.nih.gov/entrez/query.fcgi?cmd=search&db=gene&term=Ulk2) | Unc-51 like kinase 2 (C. elegans) |
| 1102 | 0,0005338 | 0,00953 | 2377,58 | 8058,66 | 0,3 | [1416667_at](https://www.affymetrix.com/LinkServlet?probeset=1416667_at) | [Ebp](http://www.ncbi.nlm.nih.gov/entrez/query.fcgi?cmd=search&db=gene&term=Ebp) | phenylalkylamine Ca2+ antagonist (emopamil) binding protein |
| 1103 | 0,0005346 | 0,00953 | 1209,45 | 4186,54 | 0,29 | [1424684_at](https://www.affymetrix.com/LinkServlet?probeset=1424684_at) | [Rab5c](http://www.ncbi.nlm.nih.gov/entrez/query.fcgi?cmd=search&db=gene&term=Rab5c) | RAB5C, member RAS oncogene family |
| 1104 | 0,0005361 | 0,00955 | 2158,71 | 5480,22 | 0,39 | [1441959_s_at](https://www.affymetrix.com/LinkServlet?probeset=1441959_s_at) | [Jkamp](http://www.ncbi.nlm.nih.gov/entrez/query.fcgi?cmd=search&db=gene&term=Jkamp) | JNK1/MAPK8-associated membrane protein |
| 1105 | 0,0005368 | 0,00955 | 302,41 | 9,06 | 33,37 | [1416704_at](https://www.affymetrix.com/LinkServlet?probeset=1416704_at) | [Mapk14](http://www.ncbi.nlm.nih.gov/entrez/query.fcgi?cmd=search&db=gene&term=Mapk14) | mitogen-activated protein kinase 14 |
| 1106 | 0,0005378 | 0,00956 | 75,42 | 311,32 | 0,24 | [1419184_a_at](https://www.affymetrix.com/LinkServlet?probeset=1419184_a_at) | [Fhl2](http://www.ncbi.nlm.nih.gov/entrez/query.fcgi?cmd=search&db=gene&term=Fhl2) | four and a half LIM domains 2 |
| 1107 | 0,0005385 | 0,00956 | 8,26 | 18,44 | 0,45 | [1423856_at](https://www.affymetrix.com/LinkServlet?probeset=1423856_at) | [NA](http://www.ncbi.nlm.nih.gov/entrez/query.fcgi?cmd=search&db=gene&term=NA) | NA |
| 1108 | 0,0005388 | 0,00956 | 4,85 | 22 | 0,22 | [1442069_at](https://www.affymetrix.com/LinkServlet?probeset=1442069_at) | [Ept1](http://www.ncbi.nlm.nih.gov/entrez/query.fcgi?cmd=search&db=gene&term=Ept1) | ethanolaminephosphotransferase 1 (CDP-ethanolamine-specific) |
| 1109 | 0,0005409 | 0,00958 | 6,53 | 47,76 | 0,14 | [1431147_at](https://www.affymetrix.com/LinkServlet?probeset=1431147_at) | [Rint1](http://www.ncbi.nlm.nih.gov/entrez/query.fcgi?cmd=search&db=gene&term=Rint1) | RAD50 interactor 1 |
| 1110 | 0,000542 | 0,00958 | 1063,62 | 2442,86 | 0,44 | [1449353_at](https://www.affymetrix.com/LinkServlet?probeset=1449353_at) | [Zmat3](http://www.ncbi.nlm.nih.gov/entrez/query.fcgi?cmd=search&db=gene&term=Zmat3) | zinc finger matrin type 3 |
| 1111 | 0,0005425 | 0,00958 | 4047,64 | 1941,65 | 2,08 | [1432848_a_at](https://www.affymetrix.com/LinkServlet?probeset=1432848_a_at) | [Frmd8](http://www.ncbi.nlm.nih.gov/entrez/query.fcgi?cmd=search&db=gene&term=Frmd8) | FERM domain containing 8 |
| 1112 | 0,0005426 | 0,00958 | 4,42 | 286,73 | 0,015 | [1443990_at](https://www.affymetrix.com/LinkServlet?probeset=1443990_at) | [Ntrk1](http://www.ncbi.nlm.nih.gov/entrez/query.fcgi?cmd=search&db=gene&term=Ntrk1) | neurotrophic tyrosine kinase, receptor, type 1 |
| 1113 | 0,0005435 | 0,00958 | 714,84 | 2112,48 | 0,34 | [1417564_at](https://www.affymetrix.com/LinkServlet?probeset=1417564_at) | [Med7](http://www.ncbi.nlm.nih.gov/entrez/query.fcgi?cmd=search&db=gene&term=Med7) | mediator complex subunit 7 |
| 1114 | 0,000544 | 0,00958 | 95,24 | 14,7 | 6,48 | [1438676_at](https://www.affymetrix.com/LinkServlet?probeset=1438676_at) | [Mpa2l](http://www.ncbi.nlm.nih.gov/entrez/query.fcgi?cmd=search&db=gene&term=Mpa2l) | macrophage activation 2 like |
| 1115 | 0,0005445 | 0,00958 | 4733,15 | 1144,34 | 4,14 | [1419054_a_at](https://www.affymetrix.com/LinkServlet?probeset=1419054_a_at) | [Ptpn21](http://www.ncbi.nlm.nih.gov/entrez/query.fcgi?cmd=search&db=gene&term=Ptpn21) | protein tyrosine phosphatase, non-receptor type 21 |
| 1116 | 0,0005446 | 0,00958 | 7,38 | 45,45 | 0,16 | [1441481_at](https://www.affymetrix.com/LinkServlet?probeset=1441481_at) | [Mfap3l](http://www.ncbi.nlm.nih.gov/entrez/query.fcgi?cmd=search&db=gene&term=Mfap3l) | microfibrillar-associated protein 3-like |
| 1117 | 0,0005448 | 0,00958 | 604,68 | 214,13 | 2,82 | [1428931_a_at](https://www.affymetrix.com/LinkServlet?probeset=1428931_a_at) | [Parp6](http://www.ncbi.nlm.nih.gov/entrez/query.fcgi?cmd=search&db=gene&term=Parp6) | poly (ADP-ribose) polymerase family, member 6 |
| 1118 | 0,0005451 | 0,00958 | 32,94 | 222,64 | 0,15 | [1427566_at](https://www.affymetrix.com/LinkServlet?probeset=1427566_at) | [Wfikkn1](http://www.ncbi.nlm.nih.gov/entrez/query.fcgi?cmd=search&db=gene&term=Wfikkn1) | WAP, FS, Ig, KU, and NTR-containing protein 1 |
| 1119 | 0,0005452 | 0,00958 | 1411,35 | 4149,23 | 0,34 | [1423176_at](https://www.affymetrix.com/LinkServlet?probeset=1423176_at) | [Tob1](http://www.ncbi.nlm.nih.gov/entrez/query.fcgi?cmd=search&db=gene&term=Tob1) | transducer of ErbB-2.1 |
| 1120 | 0,0005459 | 0,00959 | 179,27 | 31,14 | 5,76 | [1452201_at](https://www.affymetrix.com/LinkServlet?probeset=1452201_at) | [2310047B19Rik](http://www.ncbi.nlm.nih.gov/entrez/query.fcgi?cmd=search&db=gene&term=2310047B19Rik) | RIKEN cDNA 2310047B19 gene |
| 1121 | 0,0005488 | 0,00963 | 64,49 | 233,27 | 0,28 | [1436623_at](https://www.affymetrix.com/LinkServlet?probeset=1436623_at) | [Entpd7](http://www.ncbi.nlm.nih.gov/entrez/query.fcgi?cmd=search&db=gene&term=Entpd7) | ectonucleoside triphosphate diphosphohydrolase 7 |
| 1122 | 0,0005503 | 0,00963 | 1917 | 5973,24 | 0,32 | [1451611_at](https://www.affymetrix.com/LinkServlet?probeset=1451611_at) | [Pla2g16](http://www.ncbi.nlm.nih.gov/entrez/query.fcgi?cmd=search&db=gene&term=Pla2g16) | phospholipase A2, group XVI |
| 1123 | 0,0005507 | 0,00963 | 3394,78 | 6022,45 | 0,56 | [1433822_x_at](https://www.affymetrix.com/LinkServlet?probeset=1433822_x_at) | [Anapc5](http://www.ncbi.nlm.nih.gov/entrez/query.fcgi?cmd=search&db=gene&term=Anapc5) | anaphase-promoting complex subunit 5 |
| 1124 | 0,0005513 | 0,00963 | 6,78 | 11,4 | 0,59 | [1443682_at](https://www.affymetrix.com/LinkServlet?probeset=1443682_at) | [Eng](http://www.ncbi.nlm.nih.gov/entrez/query.fcgi?cmd=search&db=gene&term=Eng) | endoglin |
| 1125 | 0,000552 | 0,00963 | 1628,43 | 585,14 | 2,78 | [1417930_at](https://www.affymetrix.com/LinkServlet?probeset=1417930_at) | [Nab2](http://www.ncbi.nlm.nih.gov/entrez/query.fcgi?cmd=search&db=gene&term=Nab2) | Ngfi-A binding protein 2 |
| 1126 | 0,0005523 | 0,00963 | 377,51 | 1689,2 | 0,22 | [1434864_at](https://www.affymetrix.com/LinkServlet?probeset=1434864_at) | [Nipa1](http://www.ncbi.nlm.nih.gov/entrez/query.fcgi?cmd=search&db=gene&term=Nipa1) | non imprinted in Prader-Willi/Angelman syndrome 1 homolog (human) |
| 1127 | 0,0005523 | 0,00963 | 572,74 | 1366,49 | 0,42 | [1417203_at](https://www.affymetrix.com/LinkServlet?probeset=1417203_at) | [Ethe1](http://www.ncbi.nlm.nih.gov/entrez/query.fcgi?cmd=search&db=gene&term=Ethe1) | ethylmalonic encephalopathy 1 |
| 1128 | 0,0005527 | 0,00963 | 618,96 | 1667,5 | 0,37 | [1459861_s_at](https://www.affymetrix.com/LinkServlet?probeset=1459861_s_at) | [Kdm2b](http://www.ncbi.nlm.nih.gov/entrez/query.fcgi?cmd=search&db=gene&term=Kdm2b) | lysine (K)-specific demethylase 2B |
| 1129 | 0,0005532 | 0,00963 | 17,56 | 9,68 | 1,82 | [1449836_x_at](https://www.affymetrix.com/LinkServlet?probeset=1449836_x_at) | [Bik](http://www.ncbi.nlm.nih.gov/entrez/query.fcgi?cmd=search&db=gene&term=Bik) | BCL2-interacting killer |
| 1130 | 0,0005533 | 0,00963 | 58,59 | 362,69 | 0,16 | [1433450_at](https://www.affymetrix.com/LinkServlet?probeset=1433450_at) | [Cdk5r1](http://www.ncbi.nlm.nih.gov/entrez/query.fcgi?cmd=search&db=gene&term=Cdk5r1) | cyclin-dependent kinase 5, regulatory subunit 1 (p35) |
| 1131 | 0,0005539 | 0,00963 | 362,13 | 12,8 | 28,29 | [1439048_at](https://www.affymetrix.com/LinkServlet?probeset=1439048_at) | [NA](http://www.ncbi.nlm.nih.gov/entrez/query.fcgi?cmd=search&db=gene&term=NA) | NA |
| 1132 | 0,0005545 | 0,00963 | 193,27 | 1114,83 | 0,17 | [1440920_at](https://www.affymetrix.com/LinkServlet?probeset=1440920_at) | [Mmp14](http://www.ncbi.nlm.nih.gov/entrez/query.fcgi?cmd=search&db=gene&term=Mmp14) | matrix metallopeptidase 14 (membrane-inserted) |
| 1133 | 0,0005546 | 0,00963 | 1448,6 | 291,95 | 4,96 | [1416113_at](https://www.affymetrix.com/LinkServlet?probeset=1416113_at) | [Fkbp8](http://www.ncbi.nlm.nih.gov/entrez/query.fcgi?cmd=search&db=gene&term=Fkbp8) | FK506 binding protein 8 |
| 1134 | 0,0005554 | 0,00963 | 879,6 | 1837,14 | 0,48 | [1416479_a_at](https://www.affymetrix.com/LinkServlet?probeset=1416479_a_at) | [Tmem14c](http://www.ncbi.nlm.nih.gov/entrez/query.fcgi?cmd=search&db=gene&term=Tmem14c) | transmembrane protein 14C |
| 1135 | 0,0005563 | 0,00963 | 433,52 | 2091,52 | 0,21 | [1436512_at](https://www.affymetrix.com/LinkServlet?probeset=1436512_at) | [Arl4c](http://www.ncbi.nlm.nih.gov/entrez/query.fcgi?cmd=search&db=gene&term=Arl4c) | ADP-ribosylation factor-like 4C |
| 1136 | 0,0005568 | 0,00963 | 1001,92 | 725,05 | 1,38 | [1428728_at](https://www.affymetrix.com/LinkServlet?probeset=1428728_at) | [Ddx51](http://www.ncbi.nlm.nih.gov/entrez/query.fcgi?cmd=search&db=gene&term=Ddx51) | DEAD (Asp-Glu-Ala-Asp) box polypeptide 51 |
| 1137 | 0,000557 | 0,00963 | 9,09 | 42,12 | 0,22 | [1459948_at](https://www.affymetrix.com/LinkServlet?probeset=1459948_at) | [NA](http://www.ncbi.nlm.nih.gov/entrez/query.fcgi?cmd=search&db=gene&term=NA) | NA |
| 1138 | 0,0005578 | 0,00963 | 393,23 | 48,72 | 8,07 | [1451743_at](https://www.affymetrix.com/LinkServlet?probeset=1451743_at) | [D19Wsu162e](http://www.ncbi.nlm.nih.gov/entrez/query.fcgi?cmd=search&db=gene&term=D19Wsu162e) | DNA segment, Chr 19, Wayne State University 162, expressed |
| 1139 | 0,0005578 | 0,00963 | 3260,14 | 1063,56 | 3,07 | [1438255_at](https://www.affymetrix.com/LinkServlet?probeset=1438255_at) | [Foxn3](http://www.ncbi.nlm.nih.gov/entrez/query.fcgi?cmd=search&db=gene&term=Foxn3) | forkhead box N3 |
| 1140 | 0,0005592 | 0,00965 | 275,18 | 64,75 | 4,25 | [1448952_at](https://www.affymetrix.com/LinkServlet?probeset=1448952_at) | [A030009H04Rik](http://www.ncbi.nlm.nih.gov/entrez/query.fcgi?cmd=search&db=gene&term=A030009H04Rik) | RIKEN cDNA A030009H04 gene |
| 1141 | 0,0005599 | 0,00965 | 30,6 | 504,92 | 0,061 | [1419440_at](https://www.affymetrix.com/LinkServlet?probeset=1419440_at) | [Trim54](http://www.ncbi.nlm.nih.gov/entrez/query.fcgi?cmd=search&db=gene&term=Trim54) | tripartite motif-containing 54 |
| 1142 | 0,0005609 | 0,00966 | 410,74 | 1588,63 | 0,26 | [1450071_at](https://www.affymetrix.com/LinkServlet?probeset=1450071_at) | [Ash1l](http://www.ncbi.nlm.nih.gov/entrez/query.fcgi?cmd=search&db=gene&term=Ash1l) | ash1 (absent, small, or homeotic)-like (Drosophila) |
| 1143 | 0,0005619 | 0,00967 | 608,77 | 1515,61 | 0,4 | [1448708_at](https://www.affymetrix.com/LinkServlet?probeset=1448708_at) | [Med1](http://www.ncbi.nlm.nih.gov/entrez/query.fcgi?cmd=search&db=gene&term=Med1) | mediator complex subunit 1 |
| 1144 | 0,0005655 | 0,00971 | 1287,65 | 2373,05 | 0,54 | [1428113_at](https://www.affymetrix.com/LinkServlet?probeset=1428113_at) | [Tmtc4](http://www.ncbi.nlm.nih.gov/entrez/query.fcgi?cmd=search&db=gene&term=Tmtc4) | transmembrane and tetratricopeptide repeat containing 4 |
| 1145 | 0,0005658 | 0,00971 | 2342,78 | 855,99 | 2,74 | [1416637_at](https://www.affymetrix.com/LinkServlet?probeset=1416637_at) | [Slc4a2](http://www.ncbi.nlm.nih.gov/entrez/query.fcgi?cmd=search&db=gene&term=Slc4a2) | solute carrier family 4 (anion exchanger), member 2 |
| 1146 | 0,0005658 | 0,00971 | 83,52 | 470,36 | 0,18 | [1439888_at](https://www.affymetrix.com/LinkServlet?probeset=1439888_at) | [NA](http://www.ncbi.nlm.nih.gov/entrez/query.fcgi?cmd=search&db=gene&term=NA) | NA |
| 1147 | 0,0005675 | 0,00973 | 1075,2 | 2578,34 | 0,42 | [1438407_at](https://www.affymetrix.com/LinkServlet?probeset=1438407_at) | [Dsel](http://www.ncbi.nlm.nih.gov/entrez/query.fcgi?cmd=search&db=gene&term=Dsel) | dermatan sulfate epimerase-like |
| 1148 | 0,0005677 | 0,00973 | 2878,61 | 363,11 | 7,93 | [1436858_at](https://www.affymetrix.com/LinkServlet?probeset=1436858_at) | [Mbnl2](http://www.ncbi.nlm.nih.gov/entrez/query.fcgi?cmd=search&db=gene&term=Mbnl2) | muscleblind-like 2 |
| 1149 | 0,0005702 | 0,00976 | 8,25 | 24,69 | 0,33 | [1450045_at](https://www.affymetrix.com/LinkServlet?probeset=1450045_at) | [Srrm1](http://www.ncbi.nlm.nih.gov/entrez/query.fcgi?cmd=search&db=gene&term=Srrm1) | serine/arginine repetitive matrix 1 |
| 1150 | 0,0005712 | 0,00977 | 359,52 | 81,63 | 4,4 | [1418100_at](https://www.affymetrix.com/LinkServlet?probeset=1418100_at) | [A030009H04Rik](http://www.ncbi.nlm.nih.gov/entrez/query.fcgi?cmd=search&db=gene&term=A030009H04Rik) | RIKEN cDNA A030009H04 gene |
| 1151 | 0,0005722 | 0,00977 | 668,4 | 286,99 | 2,33 | [1417703_at](https://www.affymetrix.com/LinkServlet?probeset=1417703_at) | [Pvrl2](http://www.ncbi.nlm.nih.gov/entrez/query.fcgi?cmd=search&db=gene&term=Pvrl2) | poliovirus receptor-related 2 |
| 1152 | 0,0005727 | 0,00977 | 4401,4 | 8761,14 | 0,5 | [1416382_at](https://www.affymetrix.com/LinkServlet?probeset=1416382_at) | [Ctsc](http://www.ncbi.nlm.nih.gov/entrez/query.fcgi?cmd=search&db=gene&term=Ctsc) | cathepsin C |
| 1153 | 0,0005729 | 0,00977 | 1298,54 | 267,51 | 4,85 | [1416741_at](https://www.affymetrix.com/LinkServlet?probeset=1416741_at) | [Col5a1](http://www.ncbi.nlm.nih.gov/entrez/query.fcgi?cmd=search&db=gene&term=Col5a1) | collagen, type V, alpha 1 |
| 1154 | 0,0005734 | 0,00977 | 213,2 | 140,45 | 1,52 | [1426891_at](https://www.affymetrix.com/LinkServlet?probeset=1426891_at) | [Rpap1](http://www.ncbi.nlm.nih.gov/entrez/query.fcgi?cmd=search&db=gene&term=Rpap1) | RNA polymerase II associated protein 1 |
| 1155 | 0,0005741 | 0,00977 | 445,65 | 151,78 | 2,94 | [1436026_at](https://www.affymetrix.com/LinkServlet?probeset=1436026_at) | [Zfp703](http://www.ncbi.nlm.nih.gov/entrez/query.fcgi?cmd=search&db=gene&term=Zfp703) | zinc finger protein 703 |
| 1156 | 0,0005741 | 0,00977 | 4252,36 | 7684,7 | 0,55 | [1438557_x_at](https://www.affymetrix.com/LinkServlet?probeset=1438557_x_at) | [Dnpep](http://www.ncbi.nlm.nih.gov/entrez/query.fcgi?cmd=search&db=gene&term=Dnpep) | aspartyl aminopeptidase |
| 1157 | 0,0005764 | 0,00979 | 539,3 | 235,09 | 2,29 | [1418746_at](https://www.affymetrix.com/LinkServlet?probeset=1418746_at) | [Pnkd](http://www.ncbi.nlm.nih.gov/entrez/query.fcgi?cmd=search&db=gene&term=Pnkd) | paroxysmal nonkinesiogenic dyskinesia |
| 1158 | 0,0005769 | 0,00979 | 227,98 | 866,37 | 0,26 | [1427450_x_at](https://www.affymetrix.com/LinkServlet?probeset=1427450_x_at) | [Myo1b](http://www.ncbi.nlm.nih.gov/entrez/query.fcgi?cmd=search&db=gene&term=Myo1b) | myosin IB |
| 1159 | 0,0005773 | 0,00979 | 445,12 | 134,14 | 3,32 | [1434611_at](https://www.affymetrix.com/LinkServlet?probeset=1434611_at) | [Rnf123](http://www.ncbi.nlm.nih.gov/entrez/query.fcgi?cmd=search&db=gene&term=Rnf123) | ring finger protein 123 |
| 1160 | 0,0005775 | 0,00979 | 1211,07 | 681,55 | 1,78 | [1434326_x_at](https://www.affymetrix.com/LinkServlet?probeset=1434326_x_at) | [Coro2b](http://www.ncbi.nlm.nih.gov/entrez/query.fcgi?cmd=search&db=gene&term=Coro2b) | coronin, actin binding protein, 2B |
| 1161 | 0,0005791 | 0,00981 | 3199,75 | 7326,25 | 0,44 | [1437947_x_at](https://www.affymetrix.com/LinkServlet?probeset=1437947_x_at) | [Vdac1](http://www.ncbi.nlm.nih.gov/entrez/query.fcgi?cmd=search&db=gene&term=Vdac1) | voltage-dependent anion channel 1 |
| 1162 | 0,0005809 | 0,00983 | 420,63 | 140,24 | 3 | [1452033_at](https://www.affymetrix.com/LinkServlet?probeset=1452033_at) | [Gle1](http://www.ncbi.nlm.nih.gov/entrez/query.fcgi?cmd=search&db=gene&term=Gle1) | GLE1 RNA export mediator (yeast) |
| 1163 | 0,000581 | 0,00983 | 167,75 | 13,87 | 12,09 | [1460305_at](https://www.affymetrix.com/LinkServlet?probeset=1460305_at) | [Itga3](http://www.ncbi.nlm.nih.gov/entrez/query.fcgi?cmd=search&db=gene&term=Itga3) | integrin alpha 3 |
| 1164 | 0,000583 | 0,00983 | 1782,01 | 2857,14 | 0,62 | [1433749_at](https://www.affymetrix.com/LinkServlet?probeset=1433749_at) | [Gna13](http://www.ncbi.nlm.nih.gov/entrez/query.fcgi?cmd=search&db=gene&term=Gna13) | guanine nucleotide binding protein, alpha 13 |
| 1165 | 0,0005834 | 0,00983 | 285,13 | 33,06 | 8,63 | [1453742_at](https://www.affymetrix.com/LinkServlet?probeset=1453742_at) | [Vps33a](http://www.ncbi.nlm.nih.gov/entrez/query.fcgi?cmd=search&db=gene&term=Vps33a) | vacuolar protein sorting 33A (yeast) |
| 1166 | 0,0005839 | 0,00983 | 188,77 | 562,34 | 0,34 | [1450972_at](https://www.affymetrix.com/LinkServlet?probeset=1450972_at) | [3110040N11Rik](http://www.ncbi.nlm.nih.gov/entrez/query.fcgi?cmd=search&db=gene&term=3110040N11Rik) | RIKEN cDNA 3110040N11 gene |
| 1167 | 0,0005841 | 0,00983 | 7,94 | 33,56 | 0,24 | [1459415_at](https://www.affymetrix.com/LinkServlet?probeset=1459415_at) | [Specc1](http://www.ncbi.nlm.nih.gov/entrez/query.fcgi?cmd=search&db=gene&term=Specc1) | sperm antigen with calponin homology and coiled-coil domains 1 |
| 1168 | 0,0005843 | 0,00983 | 34,15 | 581,27 | 0,059 | [1455257_at](https://www.affymetrix.com/LinkServlet?probeset=1455257_at) | [Itgb3](http://www.ncbi.nlm.nih.gov/entrez/query.fcgi?cmd=search&db=gene&term=Itgb3) | integrin beta 3 |
| 1169 | 0,0005847 | 0,00983 | 13018,63 | 10080,69 | 1,29 | [1448103_s_at](https://www.affymetrix.com/LinkServlet?probeset=1448103_s_at) | [Nono](http://www.ncbi.nlm.nih.gov/entrez/query.fcgi?cmd=search&db=gene&term=Nono) | non-POU-domain-containing, octamer binding protein |
| 1170 | 0,0005847 | 0,00983 | 315,41 | 2352,6 | 0,13 | [1416527_at](https://www.affymetrix.com/LinkServlet?probeset=1416527_at) | [Rab32](http://www.ncbi.nlm.nih.gov/entrez/query.fcgi?cmd=search&db=gene&term=Rab32) | RAB32, member RAS oncogene family |
| 1171 | 0,0005861 | 0,00984 | 211,57 | 405,98 | 0,52 | [1455020_at](https://www.affymetrix.com/LinkServlet?probeset=1455020_at) | [Snx25](http://www.ncbi.nlm.nih.gov/entrez/query.fcgi?cmd=search&db=gene&term=Snx25) | sorting nexin 25 |
| 1172 | 0,0005869 | 0,00985 | 7485,93 | 10922,48 | 0,69 | [1456739_x_at](https://www.affymetrix.com/LinkServlet?probeset=1456739_x_at) | [Armcx2](http://www.ncbi.nlm.nih.gov/entrez/query.fcgi?cmd=search&db=gene&term=Armcx2) | armadillo repeat containing, X-linked 2 |
| 1173 | 0,0005873 | 0,00985 | 144,22 | 880,46 | 0,16 | [1434776_at](https://www.affymetrix.com/LinkServlet?probeset=1434776_at) | [Sema5a](http://www.ncbi.nlm.nih.gov/entrez/query.fcgi?cmd=search&db=gene&term=Sema5a) | sema domain, seven thrombospondin repeats (type 1 and type 1-like), transmembrane domain (TM) and short cytoplasmic domain, (semaphorin) 5A |
| 1174 | 0,0005881 | 0,00985 | 20,48 | 154,61 | 0,13 | [1443969_at](https://www.affymetrix.com/LinkServlet?probeset=1443969_at) | [Irs2](http://www.ncbi.nlm.nih.gov/entrez/query.fcgi?cmd=search&db=gene&term=Irs2) | insulin receptor substrate 2 |
| 1175 | 0,0005897 | 0,00985 | 4,47 | 40,26 | 0,11 | [1424801_at](https://www.affymetrix.com/LinkServlet?probeset=1424801_at) | [Enah](http://www.ncbi.nlm.nih.gov/entrez/query.fcgi?cmd=search&db=gene&term=Enah) | enabled homolog (Drosophila) |
| 1176 | 0,00059 | 0,00985 | 27042,28 | 17692,02 | 1,53 | [1422186_s_at](https://www.affymetrix.com/LinkServlet?probeset=1422186_s_at) | [Cyb5r3](http://www.ncbi.nlm.nih.gov/entrez/query.fcgi?cmd=search&db=gene&term=Cyb5r3) | cytochrome b5 reductase 3 |
| 1177 | 0,0005902 | 0,00985 | 10824,3 | 8259,36 | 1,31 | [1448319_at](https://www.affymetrix.com/LinkServlet?probeset=1448319_at) | [Akr1b3](http://www.ncbi.nlm.nih.gov/entrez/query.fcgi?cmd=search&db=gene&term=Akr1b3) | aldo-keto reductase family 1, member B3 (aldose reductase) |
| 1178 | 0,0005902 | 0,00985 | 369,89 | 129,73 | 2,85 | [1429337_at](https://www.affymetrix.com/LinkServlet?probeset=1429337_at) | [Tmem87b](http://www.ncbi.nlm.nih.gov/entrez/query.fcgi?cmd=search&db=gene&term=Tmem87b) | transmembrane protein 87B |
| 1179 | 0,0005903 | 0,00985 | 3817,11 | 9418,05 | 0,41 | [1418566_s_at](https://www.affymetrix.com/LinkServlet?probeset=1418566_s_at) | [Nudcd2](http://www.ncbi.nlm.nih.gov/entrez/query.fcgi?cmd=search&db=gene&term=Nudcd2) | NudC domain containing 2 |
| 1180 | 0,0005916 | 0,00985 | 244,31 | 1044,03 | 0,23 | [1417374_at](https://www.affymetrix.com/LinkServlet?probeset=1417374_at) | [Tuba4a](http://www.ncbi.nlm.nih.gov/entrez/query.fcgi?cmd=search&db=gene&term=Tuba4a) | tubulin, alpha 4A |
| 1181 | 0,0005923 | 0,00985 | 4,64 | 94,46 | 0,049 | [1457686_at](https://www.affymetrix.com/LinkServlet?probeset=1457686_at) | [Akap17b](http://www.ncbi.nlm.nih.gov/entrez/query.fcgi?cmd=search&db=gene&term=Akap17b) | A kinase (PRKA) anchor protein 17B |
| 1182 | 0,0005924 | 0,00985 | 3520,76 | 7164,81 | 0,49 | [1433771_at](https://www.affymetrix.com/LinkServlet?probeset=1433771_at) | [Fam108b](http://www.ncbi.nlm.nih.gov/entrez/query.fcgi?cmd=search&db=gene&term=Fam108b) | family with sequence similarity 108, member B |
| 1183 | 0,0005926 | 0,00985 | 6,05 | 34,48 | 0,18 | [1451679_at](https://www.affymetrix.com/LinkServlet?probeset=1451679_at) | [Arxes1](http://www.ncbi.nlm.nih.gov/entrez/query.fcgi?cmd=search&db=gene&term=Arxes1) | adipocyte-related X-chromosome expressed sequence 1 |
| 1184 | 0,0005963 | 0,00989 | 2497,74 | 636,26 | 3,93 | [1431798_a_at](https://www.affymetrix.com/LinkServlet?probeset=1431798_a_at) | [Syde1](http://www.ncbi.nlm.nih.gov/entrez/query.fcgi?cmd=search&db=gene&term=Syde1) | synapse defective 1, Rho GTPase, homolog 1 (C. elegans) |
| 1185 | 0,0005965 | 0,00989 | 8,55 | 39,76 | 0,22 | [1444021_at](https://www.affymetrix.com/LinkServlet?probeset=1444021_at) | [NA](http://www.ncbi.nlm.nih.gov/entrez/query.fcgi?cmd=search&db=gene&term=NA) | NA |
| 1186 | 0,0005967 | 0,00989 | 1385,58 | 1040,66 | 1,33 | [1415680_at](https://www.affymetrix.com/LinkServlet?probeset=1415680_at) | [Anapc1](http://www.ncbi.nlm.nih.gov/entrez/query.fcgi?cmd=search&db=gene&term=Anapc1) | anaphase promoting complex subunit 1 |
| 1187 | 0,0005975 | 0,0099 | 2001,54 | 685,37 | 2,92 | [1424380_at](https://www.affymetrix.com/LinkServlet?probeset=1424380_at) | [Vps37b](http://www.ncbi.nlm.nih.gov/entrez/query.fcgi?cmd=search&db=gene&term=Vps37b) | vacuolar protein sorting 37B (yeast) |
| 1188 | 0,0005992 | 0,00992 | 9,61 | 65,41 | 0,15 | [1454305_at](https://www.affymetrix.com/LinkServlet?probeset=1454305_at) | [Cbx3](http://www.ncbi.nlm.nih.gov/entrez/query.fcgi?cmd=search&db=gene&term=Cbx3) | chromobox homolog 3 (Drosophila HP1 gamma) |
| 1189 | 0,0005997 | 0,00992 | 1005,82 | 1954,69 | 0,51 | [1441870_s_at](https://www.affymetrix.com/LinkServlet?probeset=1441870_s_at) | [Pkd2](http://www.ncbi.nlm.nih.gov/entrez/query.fcgi?cmd=search&db=gene&term=Pkd2) | polycystic kidney disease 2 |
| 1190 | 0,0006005 | 0,00992 | 742,72 | 1244,52 | 0,6 | [1417319_at](https://www.affymetrix.com/LinkServlet?probeset=1417319_at) | [Pvrl3](http://www.ncbi.nlm.nih.gov/entrez/query.fcgi?cmd=search&db=gene&term=Pvrl3) | poliovirus receptor-related 3 |
| 1191 | 0,0006046 | 0,00998 | 6759,18 | 4296,6 | 1,57 | [1419964_s_at](https://www.affymetrix.com/LinkServlet?probeset=1419964_s_at) | [Hdgf](http://www.ncbi.nlm.nih.gov/entrez/query.fcgi?cmd=search&db=gene&term=Hdgf) | hepatoma-derived growth factor |
| 1192 | 0,0006055 | 0,00999 | 411,48 | 121,11 | 3,4 | [1420812_at](https://www.affymetrix.com/LinkServlet?probeset=1420812_at) | [Hdac7](http://www.ncbi.nlm.nih.gov/entrez/query.fcgi?cmd=search&db=gene&term=Hdac7) | histone deacetylase 7 |
| 1193 | 0,0006069 | 0,01 | 542,02 | 197,59 | 2,74 | [1428109_at](https://www.affymetrix.com/LinkServlet?probeset=1428109_at) | [Vps11](http://www.ncbi.nlm.nih.gov/entrez/query.fcgi?cmd=search&db=gene&term=Vps11) | vacuolar protein sorting 11 (yeast) |
| 1194 | 0,000612 | 0,0101 | 137,8 | 27,31 | 5,05 | [1418297_at](https://www.affymetrix.com/LinkServlet?probeset=1418297_at) | [Dpysl4](http://www.ncbi.nlm.nih.gov/entrez/query.fcgi?cmd=search&db=gene&term=Dpysl4) | dihydropyrimidinase-like 4 |
| 1195 | 0,0006131 | 0,0101 | 629,14 | 198,91 | 3,16 | [1422521_at](https://www.affymetrix.com/LinkServlet?probeset=1422521_at) | [Dctn1](http://www.ncbi.nlm.nih.gov/entrez/query.fcgi?cmd=search&db=gene&term=Dctn1) | dynactin 1 |
| 1196 | 0,000614 | 0,0101 | 7399,96 | 2327,73 | 3,18 | [1449942_a_at](https://www.affymetrix.com/LinkServlet?probeset=1449942_a_at) | [Ilk](http://www.ncbi.nlm.nih.gov/entrez/query.fcgi?cmd=search&db=gene&term=Ilk) | integrin linked kinase |
| 1197 | 0,0006145 | 0,0101 | 15,84 | 58,41 | 0,27 | [1440965_at](https://www.affymetrix.com/LinkServlet?probeset=1440965_at) | [Pigl](http://www.ncbi.nlm.nih.gov/entrez/query.fcgi?cmd=search&db=gene&term=Pigl) | phosphatidylinositol glycan anchor biosynthesis, class L |
| 1198 | 0,0006155 | 0,0101 | 5426,06 | 2664,67 | 2,04 | [1434920_a_at](https://www.affymetrix.com/LinkServlet?probeset=1434920_a_at) | [Evl](http://www.ncbi.nlm.nih.gov/entrez/query.fcgi?cmd=search&db=gene&term=Evl) | Ena-vasodilator stimulated phosphoprotein |
| 1199 | 0,0006171 | 0,0101 | 523,42 | 1639,25 | 0,32 | [1448835_at](https://www.affymetrix.com/LinkServlet?probeset=1448835_at) | [E2f6](http://www.ncbi.nlm.nih.gov/entrez/query.fcgi?cmd=search&db=gene&term=E2f6) | E2F transcription factor 6 |
| 1200 | 0,0006174 | 0,0101 | 150,19 | 620,45 | 0,24 | [1428541_at](https://www.affymetrix.com/LinkServlet?probeset=1428541_at) | [Fam115a](http://www.ncbi.nlm.nih.gov/entrez/query.fcgi?cmd=search&db=gene&term=Fam115a) | family with sequence similarity 115, member A |
| 1201 | 0,0006185 | 0,0101 | 5,27 | 90,21 | 0,058 | [1435308_at](https://www.affymetrix.com/LinkServlet?probeset=1435308_at) | [Fut9](http://www.ncbi.nlm.nih.gov/entrez/query.fcgi?cmd=search&db=gene&term=Fut9) | fucosyltransferase 9 |
| 1202 | 0,0006201 | 0,0101 | 20,22 | 497,26 | 0,041 | [1453836_a_at](https://www.affymetrix.com/LinkServlet?probeset=1453836_a_at) | [Mgll](http://www.ncbi.nlm.nih.gov/entrez/query.fcgi?cmd=search&db=gene&term=Mgll) | monoglyceride lipase |
| 1203 | 0,0006215 | 0,0102 | 744,41 | 276,86 | 2,69 | [1452958_at](https://www.affymetrix.com/LinkServlet?probeset=1452958_at) | [Asphd2](http://www.ncbi.nlm.nih.gov/entrez/query.fcgi?cmd=search&db=gene&term=Asphd2) | aspartate beta-hydroxylase domain containing 2 |
| 1204 | 0,0006219 | 0,0102 | 1657,13 | 602,52 | 2,75 | [1453556_x_at](https://www.affymetrix.com/LinkServlet?probeset=1453556_x_at) | [Cd99](http://www.ncbi.nlm.nih.gov/entrez/query.fcgi?cmd=search&db=gene&term=Cd99) | CD99 antigen |
| 1205 | 0,0006223 | 0,0102 | 243,13 | 131,87 | 1,84 | [1415717_at](https://www.affymetrix.com/LinkServlet?probeset=1415717_at) | [Rnf220](http://www.ncbi.nlm.nih.gov/entrez/query.fcgi?cmd=search&db=gene&term=Rnf220) | ring finger protein 220 |
| 1206 | 0,000625 | 0,0102 | 1980,46 | 1165,67 | 1,7 | [1418644_a_at](https://www.affymetrix.com/LinkServlet?probeset=1418644_a_at) | [Stk11](http://www.ncbi.nlm.nih.gov/entrez/query.fcgi?cmd=search&db=gene&term=Stk11) | serine/threonine kinase 11 |
| 1207 | 0,0006265 | 0,0102 | 29,34 | 322,71 | 0,091 | [1456681_at](https://www.affymetrix.com/LinkServlet?probeset=1456681_at) | [Ptchd1](http://www.ncbi.nlm.nih.gov/entrez/query.fcgi?cmd=search&db=gene&term=Ptchd1) | patched domain containing 1 |
| 1208 | 0,0006274 | 0,0102 | 433,89 | 248,51 | 1,75 | [1431232_a_at](https://www.affymetrix.com/LinkServlet?probeset=1431232_a_at) | [Mga](http://www.ncbi.nlm.nih.gov/entrez/query.fcgi?cmd=search&db=gene&term=Mga) | MAX gene associated |
| 1209 | 0,0006297 | 0,0102 | 97,05 | 9,53 | 10,18 | [1450243_a_at](https://www.affymetrix.com/LinkServlet?probeset=1450243_a_at) | [Rcan2](http://www.ncbi.nlm.nih.gov/entrez/query.fcgi?cmd=search&db=gene&term=Rcan2) | regulator of calcineurin 2 |
| 1210 | 0,0006303 | 0,0102 | 7763,63 | 4423,57 | 1,76 | [1415919_at](https://www.affymetrix.com/LinkServlet?probeset=1415919_at) | [Npdc1](http://www.ncbi.nlm.nih.gov/entrez/query.fcgi?cmd=search&db=gene&term=Npdc1) | neural proliferation, differentiation and control gene 1 |
| 1211 | 0,0006311 | 0,0102 | 12,89 | 33,67 | 0,38 | [1439236_at](https://www.affymetrix.com/LinkServlet?probeset=1439236_at) | [D15Ertd509e](http://www.ncbi.nlm.nih.gov/entrez/query.fcgi?cmd=search&db=gene&term=D15Ertd509e) | DNA segment, Chr 15, ERATO Doi 509, expressed |
| 1212 | 0,0006322 | 0,0103 | 255,15 | 3048,41 | 0,084 | [1421679_a_at](https://www.affymetrix.com/LinkServlet?probeset=1421679_a_at) | [Cdkn1a](http://www.ncbi.nlm.nih.gov/entrez/query.fcgi?cmd=search&db=gene&term=Cdkn1a) | cyclin-dependent kinase inhibitor 1A (P21) |
| 1213 | 0,0006338 | 0,0103 | 431,67 | 105 | 4,11 | [1454676_s_at](https://www.affymetrix.com/LinkServlet?probeset=1454676_s_at) | [Ticam1](http://www.ncbi.nlm.nih.gov/entrez/query.fcgi?cmd=search&db=gene&term=Ticam1) | toll-like receptor adaptor molecule 1 |
| 1214 | 0,000636 | 0,0103 | 626,62 | 161,37 | 3,88 | [1455476_a_at](https://www.affymetrix.com/LinkServlet?probeset=1455476_a_at) | [Gse1](http://www.ncbi.nlm.nih.gov/entrez/query.fcgi?cmd=search&db=gene&term=Gse1) | genetic suppressor element 1 |
| 1215 | 0,0006364 | 0,0103 | 273,42 | 70,09 | 3,9 | [1457736_at](https://www.affymetrix.com/LinkServlet?probeset=1457736_at) | [Vps37d](http://www.ncbi.nlm.nih.gov/entrez/query.fcgi?cmd=search&db=gene&term=Vps37d) | vacuolar protein sorting 37D (yeast) |
| 1216 | 0,0006393 | 0,0103 | 1635,13 | 3561,6 | 0,46 | [1418573_a_at](https://www.affymetrix.com/LinkServlet?probeset=1418573_a_at) | [Raly](http://www.ncbi.nlm.nih.gov/entrez/query.fcgi?cmd=search&db=gene&term=Raly) | hnRNP-associated with lethal yellow |
| 1217 | 0,0006396 | 0,0103 | 10,96 | 38,96 | 0,28 | [1456592_at](https://www.affymetrix.com/LinkServlet?probeset=1456592_at) | [NA](http://www.ncbi.nlm.nih.gov/entrez/query.fcgi?cmd=search&db=gene&term=NA) | NA |
| 1218 | 0,0006419 | 0,0104 | 1386,67 | 8272,42 | 0,17 | [1436746_at](https://www.affymetrix.com/LinkServlet?probeset=1436746_at) | [Wnk1](http://www.ncbi.nlm.nih.gov/entrez/query.fcgi?cmd=search&db=gene&term=Wnk1) | WNK lysine deficient protein kinase 1 |
| 1219 | 0,0006442 | 0,0104 | 9,77 | 90,6 | 0,11 | [1440868_at](https://www.affymetrix.com/LinkServlet?probeset=1440868_at) | [Gabpb2](http://www.ncbi.nlm.nih.gov/entrez/query.fcgi?cmd=search&db=gene&term=Gabpb2) | GA repeat binding protein, beta 2 |
| 1220 | 0,0006444 | 0,0104 | 1152,9 | 3994,44 | 0,29 | [1421346_a_at](https://www.affymetrix.com/LinkServlet?probeset=1421346_a_at) | [Slc6a6](http://www.ncbi.nlm.nih.gov/entrez/query.fcgi?cmd=search&db=gene&term=Slc6a6) | solute carrier family 6 (neurotransmitter transporter, taurine), member 6 |
| 1221 | 0,0006446 | 0,0104 | 4084,92 | 1401,29 | 2,92 | [1418011_a_at](https://www.affymetrix.com/LinkServlet?probeset=1418011_a_at) | [Sh3glb1](http://www.ncbi.nlm.nih.gov/entrez/query.fcgi?cmd=search&db=gene&term=Sh3glb1) | SH3-domain GRB2-like B1 (endophilin) |
| 1222 | 0,0006476 | 0,0104 | 1366,68 | 1915,46 | 0,71 | [1423200_at](https://www.affymetrix.com/LinkServlet?probeset=1423200_at) | [Ncor1](http://www.ncbi.nlm.nih.gov/entrez/query.fcgi?cmd=search&db=gene&term=Ncor1) | nuclear receptor co-repressor 1 |
| 1223 | 0,0006518 | 0,0105 | 1032,58 | 1633,96 | 0,63 | [1415729_at](https://www.affymetrix.com/LinkServlet?probeset=1415729_at) | [Pdpk1](http://www.ncbi.nlm.nih.gov/entrez/query.fcgi?cmd=search&db=gene&term=Pdpk1) | 3-phosphoinositide dependent protein kinase 1 |
| 1224 | 0,000652 | 0,0105 | 25,94 | 144,66 | 0,18 | [1454256_s_at](https://www.affymetrix.com/LinkServlet?probeset=1454256_s_at) | [1700113I22Rik](http://www.ncbi.nlm.nih.gov/entrez/query.fcgi?cmd=search&db=gene&term=1700113I22Rik) | RIKEN cDNA 1700113I22 gene |
| 1225 | 0,0006529 | 0,0105 | 319,14 | 1195,08 | 0,27 | [1424159_at](https://www.affymetrix.com/LinkServlet?probeset=1424159_at) | [Fam134c](http://www.ncbi.nlm.nih.gov/entrez/query.fcgi?cmd=search&db=gene&term=Fam134c) | family with sequence similarity 134, member C |
| 1226 | 0,0006542 | 0,0105 | 4375,72 | 1775,57 | 2,46 | [1438058_s_at](https://www.affymetrix.com/LinkServlet?probeset=1438058_s_at) | [Ptov1](http://www.ncbi.nlm.nih.gov/entrez/query.fcgi?cmd=search&db=gene&term=Ptov1) | prostate tumor over expressed gene 1 |
| 1227 | 0,0006556 | 0,0105 | 590,2 | 307,23 | 1,92 | [1451189_at](https://www.affymetrix.com/LinkServlet?probeset=1451189_at) | [Zswim1](http://www.ncbi.nlm.nih.gov/entrez/query.fcgi?cmd=search&db=gene&term=Zswim1) | zinc finger, SWIM domain containing 1 |
| 1228 | 0,0006557 | 0,0105 | 828,51 | 33,17 | 24,98 | [1450977_s_at](https://www.affymetrix.com/LinkServlet?probeset=1450977_s_at) | [Ndrg1](http://www.ncbi.nlm.nih.gov/entrez/query.fcgi?cmd=search&db=gene&term=Ndrg1) | N-myc downstream regulated gene 1 |
| 1229 | 0,0006578 | 0,0105 | 5,06 | 85,84 | 0,059 | [1459749_s_at](https://www.affymetrix.com/LinkServlet?probeset=1459749_s_at) | [Fat4](http://www.ncbi.nlm.nih.gov/entrez/query.fcgi?cmd=search&db=gene&term=Fat4) | FAT tumor suppressor homolog 4 (Drosophila) |
| 1230 | 0,0006579 | 0,0105 | 412,67 | 117,5 | 3,51 | [1418064_at](https://www.affymetrix.com/LinkServlet?probeset=1418064_at) | [Tfpt](http://www.ncbi.nlm.nih.gov/entrez/query.fcgi?cmd=search&db=gene&term=Tfpt) | TCF3 (E2A) fusion partner |
| 1231 | 0,0006583 | 0,0105 | 1110,71 | 207,07 | 5,36 | [1422450_at](https://www.affymetrix.com/LinkServlet?probeset=1422450_at) | [Ctnnd1](http://www.ncbi.nlm.nih.gov/entrez/query.fcgi?cmd=search&db=gene&term=Ctnnd1) | catenin (cadherin associated protein), delta 1 |
| 1232 | 0,0006588 | 0,0105 | 208,23 | 323,45 | 0,64 | [1452583_s_at](https://www.affymetrix.com/LinkServlet?probeset=1452583_s_at) | [Galm](http://www.ncbi.nlm.nih.gov/entrez/query.fcgi?cmd=search&db=gene&term=Galm) | galactose mutarotase |
| 1233 | 0,0006597 | 0,0105 | 93,59 | 217,67 | 0,43 | [1430407_at](https://www.affymetrix.com/LinkServlet?probeset=1430407_at) | [3110035C09Rik](http://www.ncbi.nlm.nih.gov/entrez/query.fcgi?cmd=search&db=gene&term=3110035C09Rik) | RIKEN cDNA 3110035C09 gene |
| 1234 | 0,00066 | 0,0105 | 4166,95 | 2394,71 | 1,74 | [1433995_s_at](https://www.affymetrix.com/LinkServlet?probeset=1433995_s_at) | [Ccdc50](http://www.ncbi.nlm.nih.gov/entrez/query.fcgi?cmd=search&db=gene&term=Ccdc50) | coiled-coil domain containing 50 |
| 1235 | 0,000661 | 0,0105 | 288,13 | 1078,28 | 0,27 | [1428447_at](https://www.affymetrix.com/LinkServlet?probeset=1428447_at) | [Tmem14a](http://www.ncbi.nlm.nih.gov/entrez/query.fcgi?cmd=search&db=gene&term=Tmem14a) | transmembrane protein 14A |
| 1236 | 0,0006635 | 0,0106 | 1239,61 | 823,31 | 1,51 | [1434329_s_at](https://www.affymetrix.com/LinkServlet?probeset=1434329_s_at) | [Adipor2](http://www.ncbi.nlm.nih.gov/entrez/query.fcgi?cmd=search&db=gene&term=Adipor2) | adiponectin receptor 2 |
| 1237 | 0,0006636 | 0,0106 | 1004,01 | 158,69 | 6,33 | [1448310_at](https://www.affymetrix.com/LinkServlet?probeset=1448310_at) | [Ick](http://www.ncbi.nlm.nih.gov/entrez/query.fcgi?cmd=search&db=gene&term=Ick) | intestinal cell kinase |
| 1238 | 0,0006648 | 0,0106 | 628,19 | 227,39 | 2,76 | [1452745_at](https://www.affymetrix.com/LinkServlet?probeset=1452745_at) | [Trappc9](http://www.ncbi.nlm.nih.gov/entrez/query.fcgi?cmd=search&db=gene&term=Trappc9) | trafficking protein particle complex 9 |
| 1239 | 0,0006667 | 0,0106 | 628,7 | 1003,73 | 0,63 | [1427171_at](https://www.affymetrix.com/LinkServlet?probeset=1427171_at) | [Rlf](http://www.ncbi.nlm.nih.gov/entrez/query.fcgi?cmd=search&db=gene&term=Rlf) | rearranged L-myc fusion sequence |
| 1240 | 0,0006672 | 0,0106 | 1254,08 | 266,06 | 4,71 | [1451252_at](https://www.affymetrix.com/LinkServlet?probeset=1451252_at) | [Irf2bp1](http://www.ncbi.nlm.nih.gov/entrez/query.fcgi?cmd=search&db=gene&term=Irf2bp1) | interferon regulatory factor 2 binding protein 1 |
| 1241 | 0,0006683 | 0,0106 | 371,49 | 566,9 | 0,66 | [1460195_at](https://www.affymetrix.com/LinkServlet?probeset=1460195_at) | [Mrps11](http://www.ncbi.nlm.nih.gov/entrez/query.fcgi?cmd=search&db=gene&term=Mrps11) | mitochondrial ribosomal protein S11 |
| 1242 | 0,0006708 | 0,0106 | 4,82 | 106,55 | 0,045 | [1453317_a_at](https://www.affymetrix.com/LinkServlet?probeset=1453317_a_at) | [Khdrbs3](http://www.ncbi.nlm.nih.gov/entrez/query.fcgi?cmd=search&db=gene&term=Khdrbs3) | KH domain containing, RNA binding, signal transduction associated 3 |
| 1243 | 0,0006717 | 0,0106 | 271,72 | 97,62 | 2,78 | [1428117_x_at](https://www.affymetrix.com/LinkServlet?probeset=1428117_x_at) | [NA](http://www.ncbi.nlm.nih.gov/entrez/query.fcgi?cmd=search&db=gene&term=NA) | NA |
| 1244 | 0,0006725 | 0,0106 | 1002,36 | 110,79 | 9,05 | [1451291_at](https://www.affymetrix.com/LinkServlet?probeset=1451291_at) | [Obfc2b](http://www.ncbi.nlm.nih.gov/entrez/query.fcgi?cmd=search&db=gene&term=Obfc2b) | oligonucleotide/oligosaccharide-binding fold containing 2B |
| 1245 | 0,0006736 | 0,0106 | 185,3 | 486,07 | 0,38 | [1449694_s_at](https://www.affymetrix.com/LinkServlet?probeset=1449694_s_at) | [Commd5](http://www.ncbi.nlm.nih.gov/entrez/query.fcgi?cmd=search&db=gene&term=Commd5) | COMM domain containing 5 |
| 1246 | 0,0006754 | 0,0107 | 443,79 | 41,73 | 10,64 | [1419213_at](https://www.affymetrix.com/LinkServlet?probeset=1419213_at) | [Nat6](http://www.ncbi.nlm.nih.gov/entrez/query.fcgi?cmd=search&db=gene&term=Nat6) | N-acetyltransferase 6 |
| 1247 | 0,0006761 | 0,0107 | 1532,15 | 2429,62 | 0,63 | [1452061_s_at](https://www.affymetrix.com/LinkServlet?probeset=1452061_s_at) | [Strbp](http://www.ncbi.nlm.nih.gov/entrez/query.fcgi?cmd=search&db=gene&term=Strbp) | spermatid perinuclear RNA binding protein |
| 1248 | 0,0006763 | 0,0107 | 935,5 | 453,84 | 2,06 | [1423098_at](https://www.affymetrix.com/LinkServlet?probeset=1423098_at) | [Capn7](http://www.ncbi.nlm.nih.gov/entrez/query.fcgi?cmd=search&db=gene&term=Capn7) | calpain 7 |
| 1249 | 0,0006807 | 0,0107 | 637,68 | 1999,2 | 0,32 | [1421051_s_at](https://www.affymetrix.com/LinkServlet?probeset=1421051_s_at) | [Vps25](http://www.ncbi.nlm.nih.gov/entrez/query.fcgi?cmd=search&db=gene&term=Vps25) | vacuolar protein sorting 25 (yeast) |
| 1250 | 0,0006812 | 0,0107 | 1106,22 | 511,94 | 2,16 | [1423658_at](https://www.affymetrix.com/LinkServlet?probeset=1423658_at) | [Sppl3](http://www.ncbi.nlm.nih.gov/entrez/query.fcgi?cmd=search&db=gene&term=Sppl3) | signal peptide peptidase 3 |
| 1251 | 0,0006832 | 0,0107 | 9524,63 | 5796,65 | 1,64 | [1417493_at](https://www.affymetrix.com/LinkServlet?probeset=1417493_at) | [Bmi1](http://www.ncbi.nlm.nih.gov/entrez/query.fcgi?cmd=search&db=gene&term=Bmi1) | Bmi1 polycomb ring finger oncogene |
| 1252 | 0,0006846 | 0,0107 | 496,77 | 248,22 | 2 | [1424369_at](https://www.affymetrix.com/LinkServlet?probeset=1424369_at) | [Psmf1](http://www.ncbi.nlm.nih.gov/entrez/query.fcgi?cmd=search&db=gene&term=Psmf1) | proteasome (prosome, macropain) inhibitor subunit 1 |
| 1253 | 0,0006846 | 0,0107 | 2184,87 | 4737,01 | 0,46 | [1417622_at](https://www.affymetrix.com/LinkServlet?probeset=1417622_at) | [Slc12a2](http://www.ncbi.nlm.nih.gov/entrez/query.fcgi?cmd=search&db=gene&term=Slc12a2) | solute carrier family 12, member 2 |
| 1254 | 0,000686 | 0,0108 | 422,76 | 48,13 | 8,78 | [1421900_at](https://www.affymetrix.com/LinkServlet?probeset=1421900_at) | [Eif2ak1](http://www.ncbi.nlm.nih.gov/entrez/query.fcgi?cmd=search&db=gene&term=Eif2ak1) | eukaryotic translation initiation factor 2 alpha kinase 1 |
| 1255 | 0,0006867 | 0,0108 | 1725,41 | 2682,49 | 0,64 | [1422676_at](https://www.affymetrix.com/LinkServlet?probeset=1422676_at) | [Smarce1](http://www.ncbi.nlm.nih.gov/entrez/query.fcgi?cmd=search&db=gene&term=Smarce1) | SWI/SNF related, matrix associated, actin dependent regulator of chromatin, subfamily e, member 1 |
| 1256 | 0,0006874 | 0,0108 | 342,01 | 746,25 | 0,46 | [1453983_a_at](https://www.affymetrix.com/LinkServlet?probeset=1453983_a_at) | [Mett10d](http://www.ncbi.nlm.nih.gov/entrez/query.fcgi?cmd=search&db=gene&term=Mett10d) | methyltransferase 10 domain containing |
| 1257 | 0,0006878 | 0,0108 | 178,46 | 68,17 | 2,62 | [1419006_s_at](https://www.affymetrix.com/LinkServlet?probeset=1419006_s_at) | [Peli2](http://www.ncbi.nlm.nih.gov/entrez/query.fcgi?cmd=search&db=gene&term=Peli2) | pellino 2 |
| 1258 | 0,0006878 | 0,0108 | 3354,75 | 1431,9 | 2,34 | [1424027_at](https://www.affymetrix.com/LinkServlet?probeset=1424027_at) | [Pxn](http://www.ncbi.nlm.nih.gov/entrez/query.fcgi?cmd=search&db=gene&term=Pxn) | paxillin |
| 1259 | 0,0006889 | 0,0108 | 502,52 | 308,96 | 1,63 | [1420961_a_at](https://www.affymetrix.com/LinkServlet?probeset=1420961_a_at) | [Ivns1abp](http://www.ncbi.nlm.nih.gov/entrez/query.fcgi?cmd=search&db=gene&term=Ivns1abp) | influenza virus NS1A binding protein |
| 1260 | 0,0006907 | 0,0108 | 10,92 | 41,13 | 0,27 | [1437776_at](https://www.affymetrix.com/LinkServlet?probeset=1437776_at) | [Tmcc1](http://www.ncbi.nlm.nih.gov/entrez/query.fcgi?cmd=search&db=gene&term=Tmcc1) | transmembrane and coiled coil domains 1 |
| 1261 | 0,0006917 | 0,0108 | 1865,52 | 11569,98 | 0,16 | [1423489_at](https://www.affymetrix.com/LinkServlet?probeset=1423489_at) | [Mmd](http://www.ncbi.nlm.nih.gov/entrez/query.fcgi?cmd=search&db=gene&term=Mmd) | monocyte to macrophage differentiation-associated |
| 1262 | 0,0006938 | 0,0108 | 488,75 | 233,57 | 2,09 | [1451233_at](https://www.affymetrix.com/LinkServlet?probeset=1451233_at) | [Traf2](http://www.ncbi.nlm.nih.gov/entrez/query.fcgi?cmd=search&db=gene&term=Traf2) | TNF receptor-associated factor 2 |
| 1263 | 0,0006943 | 0,0108 | 1183,83 | 623,68 | 1,9 | [1416137_at](https://www.affymetrix.com/LinkServlet?probeset=1416137_at) | [Anxa7](http://www.ncbi.nlm.nih.gov/entrez/query.fcgi?cmd=search&db=gene&term=Anxa7) | annexin A7 |
| 1264 | 0,0006945 | 0,0108 | 452,52 | 906,86 | 0,5 | [1452370_s_at](https://www.affymetrix.com/LinkServlet?probeset=1452370_s_at) | [B230208H17Rik](http://www.ncbi.nlm.nih.gov/entrez/query.fcgi?cmd=search&db=gene&term=B230208H17Rik) | RIKEN cDNA B230208H17 gene |
| 1265 | 0,0006974 | 0,0108 | 24,95 | 226,67 | 0,11 | [1448465_at](https://www.affymetrix.com/LinkServlet?probeset=1448465_at) | [Nipsnap1](http://www.ncbi.nlm.nih.gov/entrez/query.fcgi?cmd=search&db=gene&term=Nipsnap1) | 4-nitrophenylphosphatase domain and non-neuronal SNAP25-like protein homolog 1 (C. elegans) |
| 1266 | 0,0006977 | 0,0108 | 357,13 | 40,11 | 8,9 | [1427039_at](https://www.affymetrix.com/LinkServlet?probeset=1427039_at) | [Epn1](http://www.ncbi.nlm.nih.gov/entrez/query.fcgi?cmd=search&db=gene&term=Epn1) | epsin 1 |
| 1267 | 0,0006997 | 0,0109 | 2597,75 | 4481,67 | 0,58 | [1419649_s_at](https://www.affymetrix.com/LinkServlet?probeset=1419649_s_at) | [Myo1c](http://www.ncbi.nlm.nih.gov/entrez/query.fcgi?cmd=search&db=gene&term=Myo1c) | myosin IC |
| 1268 | 0,0006999 | 0,0109 | 2039,45 | 357,68 | 5,7 | [1428432_at](https://www.affymetrix.com/LinkServlet?probeset=1428432_at) | [Zcchc24](http://www.ncbi.nlm.nih.gov/entrez/query.fcgi?cmd=search&db=gene&term=Zcchc24) | zinc finger, CCHC domain containing 24 |
| 1269 | 0,0007017 | 0,0109 | 175,9 | 46,49 | 3,78 | [1427023_at](https://www.affymetrix.com/LinkServlet?probeset=1427023_at) | [Phyhipl](http://www.ncbi.nlm.nih.gov/entrez/query.fcgi?cmd=search&db=gene&term=Phyhipl) | phytanoyl-CoA hydroxylase interacting protein-like |
| 1270 | 0,0007029 | 0,0109 | 433,08 | 209,98 | 2,06 | [1427980_at](https://www.affymetrix.com/LinkServlet?probeset=1427980_at) | [4933407C03Rik](http://www.ncbi.nlm.nih.gov/entrez/query.fcgi?cmd=search&db=gene&term=4933407C03Rik) | RIKEN cDNA 4933407C03 gene |
| 1271 | 0,0007035 | 0,0109 | 2117,74 | 3421,88 | 0,62 | [1454731_at](https://www.affymetrix.com/LinkServlet?probeset=1454731_at) | [Myo10](http://www.ncbi.nlm.nih.gov/entrez/query.fcgi?cmd=search&db=gene&term=Myo10) | myosin X |
| 1272 | 0,0007039 | 0,0109 | 746,67 | 530,75 | 1,41 | [1435243_at](https://www.affymetrix.com/LinkServlet?probeset=1435243_at) | [Zfp746](http://www.ncbi.nlm.nih.gov/entrez/query.fcgi?cmd=search&db=gene&term=Zfp746) | zinc finger protein 746 |
| 1273 | 0,0007049 | 0,0109 | 5,07 | 130,86 | 0,039 | [1422617_at](https://www.affymetrix.com/LinkServlet?probeset=1422617_at) | [NA](http://www.ncbi.nlm.nih.gov/entrez/query.fcgi?cmd=search&db=gene&term=NA) | NA |
| 1274 | 0,0007053 | 0,0109 | 5928,37 | 3104,38 | 1,91 | [1437974_a_at](https://www.affymetrix.com/LinkServlet?probeset=1437974_a_at) | [Hk1](http://www.ncbi.nlm.nih.gov/entrez/query.fcgi?cmd=search&db=gene&term=Hk1) | hexokinase 1 |
| 1275 | 0,0007066 | 0,0109 | 1140,21 | 6236,06 | 0,18 | [1417135_at](https://www.affymetrix.com/LinkServlet?probeset=1417135_at) | [Srpk2](http://www.ncbi.nlm.nih.gov/entrez/query.fcgi?cmd=search&db=gene&term=Srpk2) | serine/arginine-rich protein specific kinase 2 |
| 1276 | 0,0007073 | 0,0109 | 2021,7 | 4726,34 | 0,43 | [1427877_at](https://www.affymetrix.com/LinkServlet?probeset=1427877_at) | [Zc3h15](http://www.ncbi.nlm.nih.gov/entrez/query.fcgi?cmd=search&db=gene&term=Zc3h15) | zinc finger CCCH-type containing 15 |
| 1277 | 0,0007103 | 0,0109 | 934,18 | 1401,3 | 0,67 | [1433729_x_at](https://www.affymetrix.com/LinkServlet?probeset=1433729_x_at) | [Pmpcb](http://www.ncbi.nlm.nih.gov/entrez/query.fcgi?cmd=search&db=gene&term=Pmpcb) | peptidase (mitochondrial processing) beta |
| 1278 | 0,0007121 | 0,011 | 260,55 | 158,64 | 1,64 | [1426049_a_at](https://www.affymetrix.com/LinkServlet?probeset=1426049_a_at) | [Terf2ip](http://www.ncbi.nlm.nih.gov/entrez/query.fcgi?cmd=search&db=gene&term=Terf2ip) | telomeric repeat binding factor 2, interacting protein |
| 1279 | 0,000714 | 0,011 | 242,91 | 1198,77 | 0,2 | [1451412_a_at](https://www.affymetrix.com/LinkServlet?probeset=1451412_a_at) | [Ift20](http://www.ncbi.nlm.nih.gov/entrez/query.fcgi?cmd=search&db=gene&term=Ift20) | intraflagellar transport 20 homolog (Chlamydomonas) |
| 1280 | 0,0007156 | 0,011 | 221 | 56,81 | 3,89 | [1436053_at](https://www.affymetrix.com/LinkServlet?probeset=1436053_at) | [Tbc1d22b](http://www.ncbi.nlm.nih.gov/entrez/query.fcgi?cmd=search&db=gene&term=Tbc1d22b) | TBC1 domain family, member 22B |
| 1281 | 0,0007163 | 0,011 | 6649,72 | 13261,09 | 0,5 | [1423653_at](https://www.affymetrix.com/LinkServlet?probeset=1423653_at) | [Atp1a1](http://www.ncbi.nlm.nih.gov/entrez/query.fcgi?cmd=search&db=gene&term=Atp1a1) | ATPase, Na+/K+ transporting, alpha 1 polypeptide |
| 1282 | 0,0007167 | 0,011 | 5,33 | 141,83 | 0,038 | [1417732_at](https://www.affymetrix.com/LinkServlet?probeset=1417732_at) | [Anxa8](http://www.ncbi.nlm.nih.gov/entrez/query.fcgi?cmd=search&db=gene&term=Anxa8) | annexin A8 |
| 1283 | 0,0007173 | 0,011 | 1095,78 | 548,72 | 2 | [1421733_a_at](https://www.affymetrix.com/LinkServlet?probeset=1421733_a_at) | [Tpst1](http://www.ncbi.nlm.nih.gov/entrez/query.fcgi?cmd=search&db=gene&term=Tpst1) | protein-tyrosine sulfotransferase 1 |
| 1284 | 0,0007187 | 0,011 | 6,02 | 58,83 | 0,1 | [1435708_at](https://www.affymetrix.com/LinkServlet?probeset=1435708_at) | [NA](http://www.ncbi.nlm.nih.gov/entrez/query.fcgi?cmd=search&db=gene&term=NA) | NA |
| 1285 | 0,0007203 | 0,011 | 35,34 | 67,32 | 0,52 | [1445064_at](https://www.affymetrix.com/LinkServlet?probeset=1445064_at) | [NA](http://www.ncbi.nlm.nih.gov/entrez/query.fcgi?cmd=search&db=gene&term=NA) | NA |
| 1286 | 0,0007216 | 0,011 | 116,66 | 312,6 | 0,37 | [1429059_s_at](https://www.affymetrix.com/LinkServlet?probeset=1429059_s_at) | [Tmem107](http://www.ncbi.nlm.nih.gov/entrez/query.fcgi?cmd=search&db=gene&term=Tmem107) | transmembrane protein 107 |
| 1287 | 0,0007218 | 0,011 | 141,33 | 14,18 | 9,97 | [1455509_at](https://www.affymetrix.com/LinkServlet?probeset=1455509_at) | [Nacad](http://www.ncbi.nlm.nih.gov/entrez/query.fcgi?cmd=search&db=gene&term=Nacad) | NAC alpha domain containing |
| 1288 | 0,0007227 | 0,011 | 4293,36 | 2705,03 | 1,59 | [1448668_a_at](https://www.affymetrix.com/LinkServlet?probeset=1448668_a_at) | [Irak1](http://www.ncbi.nlm.nih.gov/entrez/query.fcgi?cmd=search&db=gene&term=Irak1) | interleukin-1 receptor-associated kinase 1 |
| 1289 | 0,000723 | 0,011 | 158,93 | 669,26 | 0,24 | [1434939_at](https://www.affymetrix.com/LinkServlet?probeset=1434939_at) | [Foxf1a](http://www.ncbi.nlm.nih.gov/entrez/query.fcgi?cmd=search&db=gene&term=Foxf1a) | forkhead box F1a |
| 1290 | 0,0007256 | 0,011 | 511,87 | 14,83 | 34,51 | [1421839_at](https://www.affymetrix.com/LinkServlet?probeset=1421839_at) | [Abca1](http://www.ncbi.nlm.nih.gov/entrez/query.fcgi?cmd=search&db=gene&term=Abca1) | ATP-binding cassette, sub-family A (ABC1), member 1 |
| 1291 | 0,0007256 | 0,011 | 1432,76 | 463,96 | 3,09 | [1454783_at](https://www.affymetrix.com/LinkServlet?probeset=1454783_at) | [Il13ra1](http://www.ncbi.nlm.nih.gov/entrez/query.fcgi?cmd=search&db=gene&term=Il13ra1) | interleukin 13 receptor, alpha 1 |
| 1292 | 0,0007257 | 0,011 | 57 | 124,94 | 0,46 | [1439073_at](https://www.affymetrix.com/LinkServlet?probeset=1439073_at) | [NA](http://www.ncbi.nlm.nih.gov/entrez/query.fcgi?cmd=search&db=gene&term=NA) | NA |
| 1293 | 0,0007277 | 0,0111 | 6115,46 | 8725,38 | 0,7 | [1420609_at](https://www.affymetrix.com/LinkServlet?probeset=1420609_at) | [7-mars](http://www.ncbi.nlm.nih.gov/entrez/query.fcgi?cmd=search&db=gene&term=March7) | membrane-associated ring finger (C3HC4) 7 |
| 1294 | 0,0007284 | 0,0111 | 709,74 | 506,5 | 1,4 | [1425956_a_at](https://www.affymetrix.com/LinkServlet?probeset=1425956_a_at) | [Cdadc1](http://www.ncbi.nlm.nih.gov/entrez/query.fcgi?cmd=search&db=gene&term=Cdadc1) | cytidine and dCMP deaminase domain containing 1 |
| 1295 | 0,0007291 | 0,0111 | 1131,09 | 666,03 | 1,7 | [1433803_at](https://www.affymetrix.com/LinkServlet?probeset=1433803_at) | [Jak1](http://www.ncbi.nlm.nih.gov/entrez/query.fcgi?cmd=search&db=gene&term=Jak1) | Janus kinase 1 |
| 1296 | 0,0007299 | 0,0111 | 6305,68 | 14733,49 | 0,43 | [1423148_at](https://www.affymetrix.com/LinkServlet?probeset=1423148_at) | [Skp1a](http://www.ncbi.nlm.nih.gov/entrez/query.fcgi?cmd=search&db=gene&term=Skp1a) | S-phase kinase-associated protein 1A |
| 1297 | 0,0007302 | 0,0111 | 116,87 | 8,5 | 13,74 | [1421070_at](https://www.affymetrix.com/LinkServlet?probeset=1421070_at) | [Fam48a](http://www.ncbi.nlm.nih.gov/entrez/query.fcgi?cmd=search&db=gene&term=Fam48a) | family with sequence similarity 48, member A |
| 1298 | 0,0007306 | 0,0111 | 787,11 | 226,77 | 3,47 | [1454700_at](https://www.affymetrix.com/LinkServlet?probeset=1454700_at) | [Lrfn4](http://www.ncbi.nlm.nih.gov/entrez/query.fcgi?cmd=search&db=gene&term=Lrfn4) | leucine rich repeat and fibronectin type III domain containing 4 |
| 1299 | 0,0007308 | 0,0111 | 841,29 | 2653,13 | 0,32 | [1455009_at](https://www.affymetrix.com/LinkServlet?probeset=1455009_at) | [Cpd](http://www.ncbi.nlm.nih.gov/entrez/query.fcgi?cmd=search&db=gene&term=Cpd) | carboxypeptidase D |
| 1300 | 0,0007312 | 0,0111 | 704,93 | 30,84 | 22,86 | [1456029_a_at](https://www.affymetrix.com/LinkServlet?probeset=1456029_a_at) | [Ttc7b](http://www.ncbi.nlm.nih.gov/entrez/query.fcgi?cmd=search&db=gene&term=Ttc7b) | tetratricopeptide repeat domain 7B |
| 1301 | 0,0007318 | 0,0111 | 98,75 | 1205,44 | 0,082 | [1422632_at](https://www.affymetrix.com/LinkServlet?probeset=1422632_at) | [Ctsw](http://www.ncbi.nlm.nih.gov/entrez/query.fcgi?cmd=search&db=gene&term=Ctsw) | cathepsin W |
| 1302 | 0,0007327 | 0,0111 | 1105,85 | 3233,13 | 0,34 | [1433934_at](https://www.affymetrix.com/LinkServlet?probeset=1433934_at) | [Sec24a](http://www.ncbi.nlm.nih.gov/entrez/query.fcgi?cmd=search&db=gene&term=Sec24a) | Sec24 related gene family, member A (S. cerevisiae) |
| 1303 | 0,0007334 | 0,0111 | 158,45 | 422,46 | 0,38 | [1426277_at](https://www.affymetrix.com/LinkServlet?probeset=1426277_at) | [Tmem203](http://www.ncbi.nlm.nih.gov/entrez/query.fcgi?cmd=search&db=gene&term=Tmem203) | transmembrane protein 203 |
| 1304 | 0,0007345 | 0,0111 | 563,78 | 269,75 | 2,09 | [1436378_at](https://www.affymetrix.com/LinkServlet?probeset=1436378_at) | [Lrrfip2](http://www.ncbi.nlm.nih.gov/entrez/query.fcgi?cmd=search&db=gene&term=Lrrfip2) | leucine rich repeat (in FLII) interacting protein 2 |
| 1305 | 0,0007357 | 0,0111 | 217,12 | 128 | 1,7 | [1435535_at](https://www.affymetrix.com/LinkServlet?probeset=1435535_at) | [Depdc5](http://www.ncbi.nlm.nih.gov/entrez/query.fcgi?cmd=search&db=gene&term=Depdc5) | DEP domain containing 5 |
| 1306 | 0,0007361 | 0,0111 | 278,62 | 33,83 | 8,24 | [1429719_at](https://www.affymetrix.com/LinkServlet?probeset=1429719_at) | [Foxp4](http://www.ncbi.nlm.nih.gov/entrez/query.fcgi?cmd=search&db=gene&term=Foxp4) | forkhead box P4 |
| 1307 | 0,0007365 | 0,0111 | 411,98 | 1832,5 | 0,22 | [1421027_a_at](https://www.affymetrix.com/LinkServlet?probeset=1421027_a_at) | [Mef2c](http://www.ncbi.nlm.nih.gov/entrez/query.fcgi?cmd=search&db=gene&term=Mef2c) | myocyte enhancer factor 2C |
| 1308 | 0,0007372 | 0,0111 | 3104,33 | 5725,31 | 0,54 | [1437192_x_at](https://www.affymetrix.com/LinkServlet?probeset=1437192_x_at) | [Vdac1](http://www.ncbi.nlm.nih.gov/entrez/query.fcgi?cmd=search&db=gene&term=Vdac1) | voltage-dependent anion channel 1 |
| 1309 | 0,0007378 | 0,0111 | 124,72 | 3895,58 | 0,032 | [1436343_at](https://www.affymetrix.com/LinkServlet?probeset=1436343_at) | [Chd4](http://www.ncbi.nlm.nih.gov/entrez/query.fcgi?cmd=search&db=gene&term=Chd4) | chromodomain helicase DNA binding protein 4 |
| 1310 | 0,0007384 | 0,0111 | 6538,11 | 15792,04 | 0,41 | [1448406_at](https://www.affymetrix.com/LinkServlet?probeset=1448406_at) | [Eid1](http://www.ncbi.nlm.nih.gov/entrez/query.fcgi?cmd=search&db=gene&term=Eid1) | EP300 interacting inhibitor of differentiation 1 |
| 1311 | 0,0007388 | 0,0111 | 705,37 | 1746,87 | 0,4 | [1429503_at](https://www.affymetrix.com/LinkServlet?probeset=1429503_at) | [Fam69a](http://www.ncbi.nlm.nih.gov/entrez/query.fcgi?cmd=search&db=gene&term=Fam69a) | family with sequence similarity 69, member A |
| 1312 | 0,0007396 | 0,0111 | 14351,36 | 20341,87 | 0,71 | [1416921_x_at](https://www.affymetrix.com/LinkServlet?probeset=1416921_x_at) | [Aldoa](http://www.ncbi.nlm.nih.gov/entrez/query.fcgi?cmd=search&db=gene&term=Aldoa) | aldolase A, fructose-bisphosphate |
| 1313 | 0,0007403 | 0,0111 | 1252,48 | 2800,05 | 0,45 | [1426477_at](https://www.affymetrix.com/LinkServlet?probeset=1426477_at) | [Rasa1](http://www.ncbi.nlm.nih.gov/entrez/query.fcgi?cmd=search&db=gene&term=Rasa1) | RAS p21 protein activator 1 |
| 1314 | 0,0007403 | 0,0111 | 891,48 | 380,34 | 2,34 | [1416338_at](https://www.affymetrix.com/LinkServlet?probeset=1416338_at) | [Sh3gl1](http://www.ncbi.nlm.nih.gov/entrez/query.fcgi?cmd=search&db=gene&term=Sh3gl1) | SH3-domain GRB2-like 1 |
| 1315 | 0,0007405 | 0,0111 | 275,39 | 98,98 | 2,78 | [1427956_at](https://www.affymetrix.com/LinkServlet?probeset=1427956_at) | [Pcgf1](http://www.ncbi.nlm.nih.gov/entrez/query.fcgi?cmd=search&db=gene&term=Pcgf1) | polycomb group ring finger 1 |
| 1316 | 0,0007406 | 0,0111 | 8399,76 | 3546,03 | 2,37 | [1448605_at](https://www.affymetrix.com/LinkServlet?probeset=1448605_at) | [Rhoc](http://www.ncbi.nlm.nih.gov/entrez/query.fcgi?cmd=search&db=gene&term=Rhoc) | ras homolog gene family, member C |
| 1317 | 0,0007417 | 0,0111 | 391,02 | 17,71 | 22,08 | [1416601_a_at](https://www.affymetrix.com/LinkServlet?probeset=1416601_a_at) | [Rcan1](http://www.ncbi.nlm.nih.gov/entrez/query.fcgi?cmd=search&db=gene&term=Rcan1) | regulator of calcineurin 1 |
| 1318 | 0,0007419 | 0,0111 | 121,12 | 37,19 | 3,26 | [1448833_at](https://www.affymetrix.com/LinkServlet?probeset=1448833_at) | [Foxm1](http://www.ncbi.nlm.nih.gov/entrez/query.fcgi?cmd=search&db=gene&term=Foxm1) | forkhead box M1 |
| 1319 | 0,0007426 | 0,0111 | 157,34 | 17,59 | 8,95 | [1428118_at](https://www.affymetrix.com/LinkServlet?probeset=1428118_at) | [Lingo1](http://www.ncbi.nlm.nih.gov/entrez/query.fcgi?cmd=search&db=gene&term=Lingo1) | leucine rich repeat and Ig domain containing 1 |
| 1320 | 0,0007452 | 0,0111 | 284,16 | 1415,88 | 0,2 | [1449311_at](https://www.affymetrix.com/LinkServlet?probeset=1449311_at) | [Bach1](http://www.ncbi.nlm.nih.gov/entrez/query.fcgi?cmd=search&db=gene&term=Bach1) | BTB and CNC homology 1 |
| 1321 | 0,000747 | 0,0111 | 29,94 | 102,2 | 0,29 | [1438336_at](https://www.affymetrix.com/LinkServlet?probeset=1438336_at) | [Fbxw11](http://www.ncbi.nlm.nih.gov/entrez/query.fcgi?cmd=search&db=gene&term=Fbxw11) | F-box and WD-40 domain protein 11 |
| 1322 | 0,000747 | 0,0111 | 8317,8 | 11420,03 | 0,73 | [1423667_at](https://www.affymetrix.com/LinkServlet?probeset=1423667_at) | [Mat2a](http://www.ncbi.nlm.nih.gov/entrez/query.fcgi?cmd=search&db=gene&term=Mat2a) | methionine adenosyltransferase II, alpha |
| 1323 | 0,0007488 | 0,0111 | 977,29 | 321,07 | 3,04 | [1425601_a_at](https://www.affymetrix.com/LinkServlet?probeset=1425601_a_at) | [Rtkn](http://www.ncbi.nlm.nih.gov/entrez/query.fcgi?cmd=search&db=gene&term=Rtkn) | rhotekin |
| 1324 | 0,0007511 | 0,0111 | 793,19 | 2456,03 | 0,32 | [1435634_at](https://www.affymetrix.com/LinkServlet?probeset=1435634_at) | [Pcmtd1](http://www.ncbi.nlm.nih.gov/entrez/query.fcgi?cmd=search&db=gene&term=Pcmtd1) | protein-L-isoaspartate (D-aspartate) O-methyltransferase domain containing 1 |
| 1325 | 0,0007512 | 0,0111 | 180,25 | 1354,28 | 0,13 | [1419073_at](https://www.affymetrix.com/LinkServlet?probeset=1419073_at) | [Tmeff2](http://www.ncbi.nlm.nih.gov/entrez/query.fcgi?cmd=search&db=gene&term=Tmeff2) | transmembrane protein with EGF-like and two follistatin-like domains 2 |
| 1326 | 0,0007512 | 0,0111 | 3391,7 | 4806,87 | 0,71 | [1428715_at](https://www.affymetrix.com/LinkServlet?probeset=1428715_at) | [Gfpt1](http://www.ncbi.nlm.nih.gov/entrez/query.fcgi?cmd=search&db=gene&term=Gfpt1) | glutamine fructose-6-phosphate transaminase 1 |
| 1327 | 0,0007525 | 0,0112 | 175,83 | 20,19 | 8,71 | [1418939_at](https://www.affymetrix.com/LinkServlet?probeset=1418939_at) | [Hlx](http://www.ncbi.nlm.nih.gov/entrez/query.fcgi?cmd=search&db=gene&term=Hlx) | H2.0-like homeobox |
| 1328 | 0,0007536 | 0,0112 | 14,36 | 10,13 | 1,42 | [1444154_at](https://www.affymetrix.com/LinkServlet?probeset=1444154_at) | [Ndufa11](http://www.ncbi.nlm.nih.gov/entrez/query.fcgi?cmd=search&db=gene&term=Ndufa11) | NADH dehydrogenase (ubiquinone) 1 alpha subcomplex 11 |
| 1329 | 0,0007547 | 0,0112 | 3567,34 | 5764,25 | 0,62 | [1456695_x_at](https://www.affymetrix.com/LinkServlet?probeset=1456695_x_at) | [Anapc5](http://www.ncbi.nlm.nih.gov/entrez/query.fcgi?cmd=search&db=gene&term=Anapc5) | anaphase-promoting complex subunit 5 |
| 1330 | 0,0007572 | 0,0112 | 5,85 | 24,88 | 0,24 | [1459951_at](https://www.affymetrix.com/LinkServlet?probeset=1459951_at) | [Rps6kb1](http://www.ncbi.nlm.nih.gov/entrez/query.fcgi?cmd=search&db=gene&term=Rps6kb1) | ribosomal protein S6 kinase, polypeptide 1 |
| 1331 | 0,0007572 | 0,0112 | 4461,14 | 865,39 | 5,16 | [1434610_at](https://www.affymetrix.com/LinkServlet?probeset=1434610_at) | [Plec](http://www.ncbi.nlm.nih.gov/entrez/query.fcgi?cmd=search&db=gene&term=Plec) | plectin |
| 1332 | 0,0007576 | 0,0112 | 7195,03 | 5188,13 | 1,39 | [1416005_at](https://www.affymetrix.com/LinkServlet?probeset=1416005_at) | [Psmc1](http://www.ncbi.nlm.nih.gov/entrez/query.fcgi?cmd=search&db=gene&term=Psmc1) | protease (prosome, macropain) 26S subunit, ATPase 1 |
| 1333 | 0,0007588 | 0,0112 | 18077,03 | 8070,25 | 2,24 | [1423052_at](https://www.affymetrix.com/LinkServlet?probeset=1423052_at) | [Arf4](http://www.ncbi.nlm.nih.gov/entrez/query.fcgi?cmd=search&db=gene&term=Arf4) | ADP-ribosylation factor 4 |
| 1334 | 0,0007595 | 0,0112 | 191,37 | 842,35 | 0,23 | [1416002_x_at](https://www.affymetrix.com/LinkServlet?probeset=1416002_x_at) | [Cotl1](http://www.ncbi.nlm.nih.gov/entrez/query.fcgi?cmd=search&db=gene&term=Cotl1) | coactosin-like 1 (Dictyostelium) |
| 1335 | 0,0007605 | 0,0112 | 27,8 | 449,03 | 0,062 | [1429696_at](https://www.affymetrix.com/LinkServlet?probeset=1429696_at) | [Gpr123](http://www.ncbi.nlm.nih.gov/entrez/query.fcgi?cmd=search&db=gene&term=Gpr123) | G protein-coupled receptor 123 |
| 1336 | 0,0007608 | 0,0112 | 474,61 | 948,17 | 0,5 | [1449633_s_at](https://www.affymetrix.com/LinkServlet?probeset=1449633_s_at) | [Nt5c3l](http://www.ncbi.nlm.nih.gov/entrez/query.fcgi?cmd=search&db=gene&term=Nt5c3l) | 5'-nucleotidase, cytosolic III-like |
| 1337 | 0,0007608 | 0,0112 | 190,08 | 62,06 | 3,06 | [1455172_at](https://www.affymetrix.com/LinkServlet?probeset=1455172_at) | [AU020094](http://www.ncbi.nlm.nih.gov/entrez/query.fcgi?cmd=search&db=gene&term=AU020094) | expressed sequence AU020094 |
| 1338 | 0,0007609 | 0,0112 | 770,68 | 31,34 | 24,59 | [1434908_at](https://www.affymetrix.com/LinkServlet?probeset=1434908_at) | [Scaf1](http://www.ncbi.nlm.nih.gov/entrez/query.fcgi?cmd=search&db=gene&term=Scaf1) | SR-related CTD-associated factor 1 |
| 1339 | 0,0007624 | 0,0112 | 2354,46 | 43 | 54,75 | [1429992_at](https://www.affymetrix.com/LinkServlet?probeset=1429992_at) | [Speer4b](http://www.ncbi.nlm.nih.gov/entrez/query.fcgi?cmd=search&db=gene&term=Speer4b) | spermatogenesis associated glutamate (E)-rich protein 4b |
| 1340 | 0,000763 | 0,0112 | 602,54 | 344,03 | 1,75 | [1431339_a_at](https://www.affymetrix.com/LinkServlet?probeset=1431339_a_at) | [Efhd2](http://www.ncbi.nlm.nih.gov/entrez/query.fcgi?cmd=search&db=gene&term=Efhd2) | EF hand domain containing 2 |
| 1341 | 0,000763 | 0,0112 | 1044,51 | 361,56 | 2,89 | [1416425_at](https://www.affymetrix.com/LinkServlet?probeset=1416425_at) | [Pex19](http://www.ncbi.nlm.nih.gov/entrez/query.fcgi?cmd=search&db=gene&term=Pex19) | peroxisomal biogenesis factor 19 |
| 1342 | 0,0007634 | 0,0112 | 927,83 | 389,95 | 2,38 | [1451226_at](https://www.affymetrix.com/LinkServlet?probeset=1451226_at) | [Pex6](http://www.ncbi.nlm.nih.gov/entrez/query.fcgi?cmd=search&db=gene&term=Pex6) | peroxisomal biogenesis factor 6 |
| 1343 | 0,0007638 | 0,0112 | 3936,3 | 1143,8 | 3,44 | [1452055_at](https://www.affymetrix.com/LinkServlet?probeset=1452055_at) | [Ctdsp1](http://www.ncbi.nlm.nih.gov/entrez/query.fcgi?cmd=search&db=gene&term=Ctdsp1) | CTD (carboxy-terminal domain, RNA polymerase II, polypeptide A) small phosphatase 1 |
| 1344 | 0,0007672 | 0,0112 | 54,81 | 491,1 | 0,11 | [1449455_at](https://www.affymetrix.com/LinkServlet?probeset=1449455_at) | [Hck](http://www.ncbi.nlm.nih.gov/entrez/query.fcgi?cmd=search&db=gene&term=Hck) | hemopoietic cell kinase |
| 1345 | 0,000769 | 0,0112 | 1793,67 | 7774,12 | 0,23 | [1419038_a_at](https://www.affymetrix.com/LinkServlet?probeset=1419038_a_at) | [NA](http://www.ncbi.nlm.nih.gov/entrez/query.fcgi?cmd=search&db=gene&term=NA) | NA |
| 1346 | 0,0007693 | 0,0112 | 176,14 | 39,88 | 4,42 | [1453853_a_at](https://www.affymetrix.com/LinkServlet?probeset=1453853_a_at) | [Arhgef12](http://www.ncbi.nlm.nih.gov/entrez/query.fcgi?cmd=search&db=gene&term=Arhgef12) | Rho guanine nucleotide exchange factor (GEF) 12 |
| 1347 | 0,0007693 | 0,0112 | 953,94 | 1805,1 | 0,53 | [1449111_a_at](https://www.affymetrix.com/LinkServlet?probeset=1449111_a_at) | [Grb2](http://www.ncbi.nlm.nih.gov/entrez/query.fcgi?cmd=search&db=gene&term=Grb2) | growth factor receptor bound protein 2 |
| 1348 | 0,0007721 | 0,0113 | 756,98 | 328,58 | 2,3 | [1455026_at](https://www.affymetrix.com/LinkServlet?probeset=1455026_at) | [Sbno1](http://www.ncbi.nlm.nih.gov/entrez/query.fcgi?cmd=search&db=gene&term=Sbno1) | sno, strawberry notch homolog 1 (Drosophila) |
| 1349 | 0,0007723 | 0,0113 | 2967,33 | 5170,29 | 0,57 | [1429085_at](https://www.affymetrix.com/LinkServlet?probeset=1429085_at) | [Vezf1](http://www.ncbi.nlm.nih.gov/entrez/query.fcgi?cmd=search&db=gene&term=Vezf1) | vascular endothelial zinc finger 1 |
| 1350 | 0,0007724 | 0,0113 | 1830,28 | 754,04 | 2,43 | [1416158_at](https://www.affymetrix.com/LinkServlet?probeset=1416158_at) | [Nr2f2](http://www.ncbi.nlm.nih.gov/entrez/query.fcgi?cmd=search&db=gene&term=Nr2f2) | nuclear receptor subfamily 2, group F, member 2 |
| 1351 | 0,0007729 | 0,0113 | 152,19 | 298,98 | 0,51 | [1436198_at](https://www.affymetrix.com/LinkServlet?probeset=1436198_at) | [2310075K07Rik](http://www.ncbi.nlm.nih.gov/entrez/query.fcgi?cmd=search&db=gene&term=2310075K07Rik) | RIKEN cDNA 2310075K07 gene |
| 1352 | 0,0007742 | 0,0113 | 576,79 | 316,95 | 1,82 | [1438195_at](https://www.affymetrix.com/LinkServlet?probeset=1438195_at) | [Gpd1l](http://www.ncbi.nlm.nih.gov/entrez/query.fcgi?cmd=search&db=gene&term=Gpd1l) | glycerol-3-phosphate dehydrogenase 1-like |
| 1353 | 0,000775 | 0,0113 | 9776,6 | 19826,2 | 0,49 | [1433572_a_at](https://www.affymetrix.com/LinkServlet?probeset=1433572_a_at) | [Fam120a](http://www.ncbi.nlm.nih.gov/entrez/query.fcgi?cmd=search&db=gene&term=Fam120a) | family with sequence similarity 120, member A |
| 1354 | 0,0007756 | 0,0113 | 364,01 | 211,04 | 1,72 | [1435619_at](https://www.affymetrix.com/LinkServlet?probeset=1435619_at) | [Phf21a](http://www.ncbi.nlm.nih.gov/entrez/query.fcgi?cmd=search&db=gene&term=Phf21a) | PHD finger protein 21A |
| 1355 | 0,0007763 | 0,0113 | 54,2 | 586,65 | 0,092 | [1419853_a_at](https://www.affymetrix.com/LinkServlet?probeset=1419853_a_at) | [P2rx7](http://www.ncbi.nlm.nih.gov/entrez/query.fcgi?cmd=search&db=gene&term=P2rx7) | purinergic receptor P2X, ligand-gated ion channel, 7 |
| 1356 | 0,0007775 | 0,0113 | 768,76 | 1894,44 | 0,41 | [1429046_at](https://www.affymetrix.com/LinkServlet?probeset=1429046_at) | [Smurf2](http://www.ncbi.nlm.nih.gov/entrez/query.fcgi?cmd=search&db=gene&term=Smurf2) | SMAD specific E3 ubiquitin protein ligase 2 |
| 1357 | 0,000779 | 0,0113 | 835,34 | 1382,85 | 0,6 | [1435017_at](https://www.affymetrix.com/LinkServlet?probeset=1435017_at) | [Cisd3](http://www.ncbi.nlm.nih.gov/entrez/query.fcgi?cmd=search&db=gene&term=Cisd3) | CDGSH iron sulfur domain 3 |
| 1358 | 0,000781 | 0,0113 | 411,54 | 190,79 | 2,16 | [1428946_at](https://www.affymetrix.com/LinkServlet?probeset=1428946_at) | [Uba6](http://www.ncbi.nlm.nih.gov/entrez/query.fcgi?cmd=search&db=gene&term=Uba6) | ubiquitin-like modifier activating enzyme 6 |
| 1359 | 0,0007814 | 0,0113 | 360,78 | 615,12 | 0,59 | [1429418_at](https://www.affymetrix.com/LinkServlet?probeset=1429418_at) | [Cdc14b](http://www.ncbi.nlm.nih.gov/entrez/query.fcgi?cmd=search&db=gene&term=Cdc14b) | CDC14 cell division cycle 14 homolog B (S. cerevisiae) |
| 1360 | 0,0007818 | 0,0113 | 1543,82 | 550,83 | 2,8 | [1415952_at](https://www.affymetrix.com/LinkServlet?probeset=1415952_at) | [Mark2](http://www.ncbi.nlm.nih.gov/entrez/query.fcgi?cmd=search&db=gene&term=Mark2) | MAP/microtubule affinity-regulating kinase 2 |
| 1361 | 0,0007827 | 0,0113 | 425,78 | 8,97 | 47,46 | [1419099_x_at](https://www.affymetrix.com/LinkServlet?probeset=1419099_x_at) | [Stom](http://www.ncbi.nlm.nih.gov/entrez/query.fcgi?cmd=search&db=gene&term=Stom) | stomatin |
| 1362 | 0,0007828 | 0,0113 | 135,19 | 36,13 | 3,74 | [1422063_a_at](https://www.affymetrix.com/LinkServlet?probeset=1422063_a_at) | [Pex5](http://www.ncbi.nlm.nih.gov/entrez/query.fcgi?cmd=search&db=gene&term=Pex5) | peroxisomal biogenesis factor 5 |
| 1363 | 0,0007831 | 0,0113 | 20164,74 | 10421,87 | 1,93 | [1451086_s_at](https://www.affymetrix.com/LinkServlet?probeset=1451086_s_at) | [Rac1](http://www.ncbi.nlm.nih.gov/entrez/query.fcgi?cmd=search&db=gene&term=Rac1) | RAS-related C3 botulinum substrate 1 |
| 1364 | 0,0007846 | 0,0113 | 9,91 | 258,06 | 0,038 | [1421230_a_at](https://www.affymetrix.com/LinkServlet?probeset=1421230_a_at) | [Msi2](http://www.ncbi.nlm.nih.gov/entrez/query.fcgi?cmd=search&db=gene&term=Msi2) | Musashi homolog 2 (Drosophila) |
| 1365 | 0,0007854 | 0,0113 | 447,73 | 144,81 | 3,09 | [1434309_at](https://www.affymetrix.com/LinkServlet?probeset=1434309_at) | [Fntb](http://www.ncbi.nlm.nih.gov/entrez/query.fcgi?cmd=search&db=gene&term=Fntb) | farnesyltransferase, CAAX box, beta |
| 1366 | 0,0007859 | 0,0113 | 7959,6 | 4395,7 | 1,81 | [1428272_at](https://www.affymetrix.com/LinkServlet?probeset=1428272_at) | [Eif1b](http://www.ncbi.nlm.nih.gov/entrez/query.fcgi?cmd=search&db=gene&term=Eif1b) | eukaryotic translation initiation factor 1B |
| 1367 | 0,0007885 | 0,0113 | 160,57 | 9,02 | 17,8 | [1436780_at](https://www.affymetrix.com/LinkServlet?probeset=1436780_at) | [Ogt](http://www.ncbi.nlm.nih.gov/entrez/query.fcgi?cmd=search&db=gene&term=Ogt) | O-linked N-acetylglucosamine (GlcNAc) transferase (UDP-N-acetylglucosamine:polypeptide-N-acetylglucosaminyl transferase) |
| 1368 | 0,0007887 | 0,0113 | 27,07 | 442,7 | 0,061 | [1457262_at](https://www.affymetrix.com/LinkServlet?probeset=1457262_at) | [Smg1](http://www.ncbi.nlm.nih.gov/entrez/query.fcgi?cmd=search&db=gene&term=Smg1) | SMG1 homolog, phosphatidylinositol 3-kinase-related kinase (C. elegans) |
| 1369 | 0,0007898 | 0,0113 | 370,12 | 7,38 | 50,15 | [1426888_at](https://www.affymetrix.com/LinkServlet?probeset=1426888_at) | [Ehmt2](http://www.ncbi.nlm.nih.gov/entrez/query.fcgi?cmd=search&db=gene&term=Ehmt2) | euchromatic histone lysine N-methyltransferase 2 |
| 1370 | 0,0007907 | 0,0113 | 105,36 | 409,19 | 0,26 | [1448009_at](https://www.affymetrix.com/LinkServlet?probeset=1448009_at) | [Uggt1](http://www.ncbi.nlm.nih.gov/entrez/query.fcgi?cmd=search&db=gene&term=Uggt1) | UDP-glucose glycoprotein glucosyltransferase 1 |
| 1371 | 0,0007916 | 0,0113 | 1674,17 | 570,38 | 2,94 | [1460675_at](https://www.affymetrix.com/LinkServlet?probeset=1460675_at) | [Igsf8](http://www.ncbi.nlm.nih.gov/entrez/query.fcgi?cmd=search&db=gene&term=Igsf8) | immunoglobulin superfamily, member 8 |
| 1372 | 0,0007923 | 0,0113 | 5948,34 | 4167,54 | 1,43 | [1424760_a_at](https://www.affymetrix.com/LinkServlet?probeset=1424760_a_at) | [Smyd2](http://www.ncbi.nlm.nih.gov/entrez/query.fcgi?cmd=search&db=gene&term=Smyd2) | SET and MYND domain containing 2 |
| 1373 | 0,0007924 | 0,0113 | 112,83 | 187,11 | 0,6 | [1417277_at](https://www.affymetrix.com/LinkServlet?probeset=1417277_at) | [Cyp4f16](http://www.ncbi.nlm.nih.gov/entrez/query.fcgi?cmd=search&db=gene&term=Cyp4f16) | cytochrome P450, family 4, subfamily f, polypeptide 16 |
| 1374 | 0,0007924 | 0,0113 | 905,08 | 1182,41 | 0,77 | [1437067_at](https://www.affymetrix.com/LinkServlet?probeset=1437067_at) | [Phtf2](http://www.ncbi.nlm.nih.gov/entrez/query.fcgi?cmd=search&db=gene&term=Phtf2) | putative homeodomain transcription factor 2 |
| 1375 | 0,0007949 | 0,0114 | 296,21 | 24,98 | 11,86 | [1453762_at](https://www.affymetrix.com/LinkServlet?probeset=1453762_at) | [Vps26b](http://www.ncbi.nlm.nih.gov/entrez/query.fcgi?cmd=search&db=gene&term=Vps26b) | vacuolar protein sorting 26 homolog B (yeast) |
| 1376 | 0,0007949 | 0,0114 | 54,81 | 256,8 | 0,21 | [1450387_s_at](https://www.affymetrix.com/LinkServlet?probeset=1450387_s_at) | [Ak4](http://www.ncbi.nlm.nih.gov/entrez/query.fcgi?cmd=search&db=gene&term=Ak4) | adenylate kinase 4 |
| 1377 | 0,0007959 | 0,0114 | 1032,99 | 1665,61 | 0,62 | [1418128_at](https://www.affymetrix.com/LinkServlet?probeset=1418128_at) | [Adcy6](http://www.ncbi.nlm.nih.gov/entrez/query.fcgi?cmd=search&db=gene&term=Adcy6) | adenylate cyclase 6 |
| 1378 | 0,0007981 | 0,0114 | 28,52 | 144,35 | 0,2 | [1455461_at](https://www.affymetrix.com/LinkServlet?probeset=1455461_at) | [A530017D24Rik](http://www.ncbi.nlm.nih.gov/entrez/query.fcgi?cmd=search&db=gene&term=A530017D24Rik) | RIKEN cDNA A530017D24 gene |
| 1379 | 0,0007988 | 0,0114 | 4,42 | 106,64 | 0,041 | [1423258_at](https://www.affymetrix.com/LinkServlet?probeset=1423258_at) | [Syt9](http://www.ncbi.nlm.nih.gov/entrez/query.fcgi?cmd=search&db=gene&term=Syt9) | synaptotagmin IX |
| 1380 | 0,0007994 | 0,0114 | 3618,43 | 2598,92 | 1,39 | [1454808_at](https://www.affymetrix.com/LinkServlet?probeset=1454808_at) | [Efha1](http://www.ncbi.nlm.nih.gov/entrez/query.fcgi?cmd=search&db=gene&term=Efha1) | EF hand domain family A1 |
| 1381 | 0,0008021 | 0,0114 | 2448,05 | 1723,75 | 1,42 | [1416045_a_at](https://www.affymetrix.com/LinkServlet?probeset=1416045_a_at) | [Smarcb1](http://www.ncbi.nlm.nih.gov/entrez/query.fcgi?cmd=search&db=gene&term=Smarcb1) | SWI/SNF related, matrix associated, actin dependent regulator of chromatin, subfamily b, member 1 |
| 1382 | 0,0008028 | 0,0114 | 1953,93 | 343,16 | 5,69 | [1437841_x_at](https://www.affymetrix.com/LinkServlet?probeset=1437841_x_at) | [Csdc2](http://www.ncbi.nlm.nih.gov/entrez/query.fcgi?cmd=search&db=gene&term=Csdc2) | cold shock domain containing C2, RNA binding |
| 1383 | 0,0008043 | 0,0114 | 6,17 | 134,35 | 0,046 | [1435945_a_at](https://www.affymetrix.com/LinkServlet?probeset=1435945_a_at) | [Kcnn4](http://www.ncbi.nlm.nih.gov/entrez/query.fcgi?cmd=search&db=gene&term=Kcnn4) | potassium intermediate/small conductance calcium-activated channel, subfamily N, member 4 |
| 1384 | 0,0008046 | 0,0114 | 293,15 | 1880,74 | 0,16 | [1448300_at](https://www.affymetrix.com/LinkServlet?probeset=1448300_at) | [Mgst3](http://www.ncbi.nlm.nih.gov/entrez/query.fcgi?cmd=search&db=gene&term=Mgst3) | microsomal glutathione S-transferase 3 |
| 1385 | 0,0008052 | 0,0114 | 18,15 | 118,92 | 0,15 | [1453636_at](https://www.affymetrix.com/LinkServlet?probeset=1453636_at) | [Pcgf5](http://www.ncbi.nlm.nih.gov/entrez/query.fcgi?cmd=search&db=gene&term=Pcgf5) | polycomb group ring finger 5 |
| 1386 | 0,0008068 | 0,0114 | 18,68 | 268,96 | 0,069 | [1422123_s_at](https://www.affymetrix.com/LinkServlet?probeset=1422123_s_at) | [NA](http://www.ncbi.nlm.nih.gov/entrez/query.fcgi?cmd=search&db=gene&term=NA) | NA |
| 1387 | 0,0008077 | 0,0115 | 284,89 | 38,06 | 7,49 | [1428430_at](https://www.affymetrix.com/LinkServlet?probeset=1428430_at) | [Rgmb](http://www.ncbi.nlm.nih.gov/entrez/query.fcgi?cmd=search&db=gene&term=Rgmb) | RGM domain family, member B |
| 1388 | 0,0008104 | 0,0115 | 2545,17 | 1512,64 | 1,68 | [1433812_at](https://www.affymetrix.com/LinkServlet?probeset=1433812_at) | [Lix1l](http://www.ncbi.nlm.nih.gov/entrez/query.fcgi?cmd=search&db=gene&term=Lix1l) | Lix1-like |
| 1389 | 0,0008119 | 0,0115 | 1235,59 | 563,62 | 2,19 | [1423493_a_at](https://www.affymetrix.com/LinkServlet?probeset=1423493_a_at) | [Nfix](http://www.ncbi.nlm.nih.gov/entrez/query.fcgi?cmd=search&db=gene&term=Nfix) | nuclear factor I/X |
| 1390 | 0,0008131 | 0,0115 | 1757,16 | 3372,87 | 0,52 | [1450663_at](https://www.affymetrix.com/LinkServlet?probeset=1450663_at) | [Thbs2](http://www.ncbi.nlm.nih.gov/entrez/query.fcgi?cmd=search&db=gene&term=Thbs2) | thrombospondin 2 |
| 1391 | 0,0008138 | 0,0115 | 36,32 | 82,25 | 0,44 | [1455204_at](https://www.affymetrix.com/LinkServlet?probeset=1455204_at) | [Pitpnc1](http://www.ncbi.nlm.nih.gov/entrez/query.fcgi?cmd=search&db=gene&term=Pitpnc1) | phosphatidylinositol transfer protein, cytoplasmic 1 |
| 1392 | 0,000816 | 0,0115 | 1006,5 | 587,19 | 1,71 | [1419465_at](https://www.affymetrix.com/LinkServlet?probeset=1419465_at) | [Nkd2](http://www.ncbi.nlm.nih.gov/entrez/query.fcgi?cmd=search&db=gene&term=Nkd2) | naked cuticle 2 homolog (Drosophila) |
| 1393 | 0,0008161 | 0,0115 | 16,8 | 50,95 | 0,33 | [1421545_a_at](https://www.affymetrix.com/LinkServlet?probeset=1421545_a_at) | [Syne1](http://www.ncbi.nlm.nih.gov/entrez/query.fcgi?cmd=search&db=gene&term=Syne1) | synaptic nuclear envelope 1 |
| 1394 | 0,0008163 | 0,0115 | 182,79 | 5,7 | 32,09 | [1427414_at](https://www.affymetrix.com/LinkServlet?probeset=1427414_at) | [Prkar2a](http://www.ncbi.nlm.nih.gov/entrez/query.fcgi?cmd=search&db=gene&term=Prkar2a) | protein kinase, cAMP dependent regulatory, type II alpha |
| 1395 | 0,0008171 | 0,0115 | 2791,73 | 1838,44 | 1,52 | [1460209_at](https://www.affymetrix.com/LinkServlet?probeset=1460209_at) | [Usp39](http://www.ncbi.nlm.nih.gov/entrez/query.fcgi?cmd=search&db=gene&term=Usp39) | ubiquitin specific peptidase 39 |
| 1396 | 0,0008176 | 0,0115 | 799,08 | 1976,17 | 0,4 | [1449799_s_at](https://www.affymetrix.com/LinkServlet?probeset=1449799_s_at) | [Pkp2](http://www.ncbi.nlm.nih.gov/entrez/query.fcgi?cmd=search&db=gene&term=Pkp2) | plakophilin 2 |
| 1397 | 0,0008181 | 0,0115 | 1065,47 | 2003,6 | 0,53 | [1424632_a_at](https://www.affymetrix.com/LinkServlet?probeset=1424632_a_at) | [Rev3l](http://www.ncbi.nlm.nih.gov/entrez/query.fcgi?cmd=search&db=gene&term=Rev3l) | REV3-like, catalytic subunit of DNA polymerase zeta RAD54 like (S. cerevisiae) |
| 1398 | 0,0008217 | 0,0116 | 13458,37 | 5197,67 | 2,59 | [1451689_a_at](https://www.affymetrix.com/LinkServlet?probeset=1451689_a_at) | [Sox10](http://www.ncbi.nlm.nih.gov/entrez/query.fcgi?cmd=search&db=gene&term=Sox10) | SRY-box containing gene 10 |
| 1399 | 0,0008229 | 0,0116 | 6013,83 | 3416,53 | 1,76 | [1419490_at](https://www.affymetrix.com/LinkServlet?probeset=1419490_at) | [Fam19a5](http://www.ncbi.nlm.nih.gov/entrez/query.fcgi?cmd=search&db=gene&term=Fam19a5) | family with sequence similarity 19, member A5 |
| 1400 | 0,0008237 | 0,0116 | 98,85 | 353,8 | 0,28 | [1424682_at](https://www.affymetrix.com/LinkServlet?probeset=1424682_at) | [Gpn3](http://www.ncbi.nlm.nih.gov/entrez/query.fcgi?cmd=search&db=gene&term=Gpn3) | GPN-loop GTPase 3 |
| 1401 | 0,0008254 | 0,0116 | 5,07 | 22,31 | 0,23 | [1425874_at](https://www.affymetrix.com/LinkServlet?probeset=1425874_at) | [Hoxc13](http://www.ncbi.nlm.nih.gov/entrez/query.fcgi?cmd=search&db=gene&term=Hoxc13) | homeobox C13 |
| 1402 | 0,0008288 | 0,0116 | 697,59 | 356,86 | 1,95 | [1418404_at](https://www.affymetrix.com/LinkServlet?probeset=1418404_at) | [Rad9](http://www.ncbi.nlm.nih.gov/entrez/query.fcgi?cmd=search&db=gene&term=Rad9) | RAD9 homolog (S. pombe) |
| 1403 | 0,000829 | 0,0116 | 105,98 | 33,69 | 3,15 | [1438683_at](https://www.affymetrix.com/LinkServlet?probeset=1438683_at) | [Wasf2](http://www.ncbi.nlm.nih.gov/entrez/query.fcgi?cmd=search&db=gene&term=Wasf2) | WAS protein family, member 2 |
| 1404 | 0,0008308 | 0,0116 | 383,11 | 3729 | 0,1 | [1449292_at](https://www.affymetrix.com/LinkServlet?probeset=1449292_at) | [Rb1cc1](http://www.ncbi.nlm.nih.gov/entrez/query.fcgi?cmd=search&db=gene&term=Rb1cc1) | RB1-inducible coiled-coil 1 |
| 1405 | 0,0008325 | 0,0116 | 1503,08 | 590,91 | 2,54 | [1416273_at](https://www.affymetrix.com/LinkServlet?probeset=1416273_at) | [Tnfaip2](http://www.ncbi.nlm.nih.gov/entrez/query.fcgi?cmd=search&db=gene&term=Tnfaip2) | tumor necrosis factor, alpha-induced protein 2 |
| 1406 | 0,000833 | 0,0116 | 97,86 | 258,56 | 0,38 | [1451678_at](https://www.affymetrix.com/LinkServlet?probeset=1451678_at) | [Narf](http://www.ncbi.nlm.nih.gov/entrez/query.fcgi?cmd=search&db=gene&term=Narf) | nuclear prelamin A recognition factor |
| 1407 | 0,0008333 | 0,0116 | 193,72 | 374,12 | 0,52 | [1416195_at](https://www.affymetrix.com/LinkServlet?probeset=1416195_at) | [Inpp5k](http://www.ncbi.nlm.nih.gov/entrez/query.fcgi?cmd=search&db=gene&term=Inpp5k) | inositol polyphosphate 5-phosphatase K |
| 1408 | 0,0008347 | 0,0117 | 3572,24 | 2116,83 | 1,69 | [1428714_at](https://www.affymetrix.com/LinkServlet?probeset=1428714_at) | [Pgrmc2](http://www.ncbi.nlm.nih.gov/entrez/query.fcgi?cmd=search&db=gene&term=Pgrmc2) | progesterone receptor membrane component 2 |
| 1409 | 0,000838 | 0,0117 | 8783,02 | 4994,34 | 1,76 | [1416138_at](https://www.affymetrix.com/LinkServlet?probeset=1416138_at) | [Anxa7](http://www.ncbi.nlm.nih.gov/entrez/query.fcgi?cmd=search&db=gene&term=Anxa7) | annexin A7 |
| 1410 | 0,0008388 | 0,0117 | 196,25 | 383,27 | 0,51 | [1436317_at](https://www.affymetrix.com/LinkServlet?probeset=1436317_at) | [NA](http://www.ncbi.nlm.nih.gov/entrez/query.fcgi?cmd=search&db=gene&term=NA) | NA |
| 1411 | 0,0008408 | 0,0117 | 362,8 | 12,02 | 30,18 | [1420547_at](https://www.affymetrix.com/LinkServlet?probeset=1420547_at) | [Galc](http://www.ncbi.nlm.nih.gov/entrez/query.fcgi?cmd=search&db=gene&term=Galc) | galactosylceramidase |
| 1412 | 0,0008446 | 0,0118 | 607,12 | 10,77 | 56,39 | [1427646_a_at](https://www.affymetrix.com/LinkServlet?probeset=1427646_a_at) | [Arhgef2](http://www.ncbi.nlm.nih.gov/entrez/query.fcgi?cmd=search&db=gene&term=Arhgef2) | rho/rac guanine nucleotide exchange factor (GEF) 2 |
| 1413 | 0,0008454 | 0,0118 | 624,2 | 1069,86 | 0,58 | [1455152_at](https://www.affymetrix.com/LinkServlet?probeset=1455152_at) | [AI462493](http://www.ncbi.nlm.nih.gov/entrez/query.fcgi?cmd=search&db=gene&term=AI462493) | expressed sequence AI462493 |
| 1414 | 0,0008508 | 0,0118 | 5462,08 | 2696,25 | 2,03 | [1415753_at](https://www.affymetrix.com/LinkServlet?probeset=1415753_at) | [Fam108a](http://www.ncbi.nlm.nih.gov/entrez/query.fcgi?cmd=search&db=gene&term=Fam108a) | family with sequence similarity 108, member A |
| 1415 | 0,0008563 | 0,0119 | 22928,81 | 9258,15 | 2,48 | [1426385_x_at](https://www.affymetrix.com/LinkServlet?probeset=1426385_x_at) | [Ywhae](http://www.ncbi.nlm.nih.gov/entrez/query.fcgi?cmd=search&db=gene&term=Ywhae) | tyrosine 3-monooxygenase/tryptophan 5-monooxygenase activation protein, epsilon polypeptide |
| 1416 | 0,0008582 | 0,0119 | 2061,76 | 6278,66 | 0,33 | [1452152_at](https://www.affymetrix.com/LinkServlet?probeset=1452152_at) | [Clint1](http://www.ncbi.nlm.nih.gov/entrez/query.fcgi?cmd=search&db=gene&term=Clint1) | clathrin interactor 1 |
| 1417 | 0,0008592 | 0,0119 | 7441,22 | 3363,62 | 2,21 | [1423680_at](https://www.affymetrix.com/LinkServlet?probeset=1423680_at) | [Fads1](http://www.ncbi.nlm.nih.gov/entrez/query.fcgi?cmd=search&db=gene&term=Fads1) | fatty acid desaturase 1 |
| 1418 | 0,0008593 | 0,0119 | 596,19 | 239,2 | 2,49 | [1436758_at](https://www.affymetrix.com/LinkServlet?probeset=1436758_at) | [Hdac4](http://www.ncbi.nlm.nih.gov/entrez/query.fcgi?cmd=search&db=gene&term=Hdac4) | histone deacetylase 4 |
| 1419 | 0,0008616 | 0,0119 | 16,66 | 144,58 | 0,12 | [1435282_at](https://www.affymetrix.com/LinkServlet?probeset=1435282_at) | [Fam189a2](http://www.ncbi.nlm.nih.gov/entrez/query.fcgi?cmd=search&db=gene&term=Fam189a2) | family with sequence similarity 189, member A2 |
| 1420 | 0,0008639 | 0,012 | 28 | 231,93 | 0,12 | [1425050_at](https://www.affymetrix.com/LinkServlet?probeset=1425050_at) | [Isoc1](http://www.ncbi.nlm.nih.gov/entrez/query.fcgi?cmd=search&db=gene&term=Isoc1) | isochorismatase domain containing 1 |
| 1421 | 0,0008641 | 0,012 | 1221,21 | 752,71 | 1,62 | [1421810_at](https://www.affymetrix.com/LinkServlet?probeset=1421810_at) | [Dgcr2](http://www.ncbi.nlm.nih.gov/entrez/query.fcgi?cmd=search&db=gene&term=Dgcr2) | DiGeorge syndrome critical region gene 2 |
| 1422 | 0,0008642 | 0,012 | 284,48 | 110,57 | 2,57 | [1449490_at](https://www.affymetrix.com/LinkServlet?probeset=1449490_at) | [Mbd4](http://www.ncbi.nlm.nih.gov/entrez/query.fcgi?cmd=search&db=gene&term=Mbd4) | methyl-CpG binding domain protein 4 |
| 1423 | 0,0008653 | 0,012 | 699,47 | 75,07 | 9,32 | [1431285_at](https://www.affymetrix.com/LinkServlet?probeset=1431285_at) | [Mgrn1](http://www.ncbi.nlm.nih.gov/entrez/query.fcgi?cmd=search&db=gene&term=Mgrn1) | mahogunin, ring finger 1 |
| 1424 | 0,0008661 | 0,012 | 42,35 | 73,84 | 0,57 | [1458678_at](https://www.affymetrix.com/LinkServlet?probeset=1458678_at) | [Ndufab1](http://www.ncbi.nlm.nih.gov/entrez/query.fcgi?cmd=search&db=gene&term=Ndufab1) | NADH dehydrogenase (ubiquinone) 1, alpha/beta subcomplex, 1 |
| 1425 | 0,0008663 | 0,012 | 173,15 | 53,89 | 3,21 | [1431985_at](https://www.affymetrix.com/LinkServlet?probeset=1431985_at) | [Zfp846](http://www.ncbi.nlm.nih.gov/entrez/query.fcgi?cmd=search&db=gene&term=Zfp846) | zinc finger protein 846 |
| 1426 | 0,0008682 | 0,012 | 3244,77 | 937,59 | 3,46 | [1429314_at](https://www.affymetrix.com/LinkServlet?probeset=1429314_at) | [Syt11](http://www.ncbi.nlm.nih.gov/entrez/query.fcgi?cmd=search&db=gene&term=Syt11) | synaptotagmin XI |
| 1427 | 0,0008686 | 0,012 | 154,82 | 1293,5 | 0,12 | [1457281_at](https://www.affymetrix.com/LinkServlet?probeset=1457281_at) | [Dnajc21](http://www.ncbi.nlm.nih.gov/entrez/query.fcgi?cmd=search&db=gene&term=Dnajc21) | DnaJ (Hsp40) homolog, subfamily C, member 21 |
| 1428 | 0,0008693 | 0,012 | 901,9 | 1239,96 | 0,73 | [1423711_at](https://www.affymetrix.com/LinkServlet?probeset=1423711_at) | [Ndufaf1](http://www.ncbi.nlm.nih.gov/entrez/query.fcgi?cmd=search&db=gene&term=Ndufaf1) | NADH dehydrogenase (ubiquinone) 1 alpha subcomplex, assembly factor 1 |
| 1429 | 0,0008694 | 0,012 | 17855,89 | 8655,44 | 2,06 | [1426384_a_at](https://www.affymetrix.com/LinkServlet?probeset=1426384_a_at) | [Ywhae](http://www.ncbi.nlm.nih.gov/entrez/query.fcgi?cmd=search&db=gene&term=Ywhae) | tyrosine 3-monooxygenase/tryptophan 5-monooxygenase activation protein, epsilon polypeptide |
| 1430 | 0,0008699 | 0,012 | 15,55 | 253,19 | 0,061 | [1460011_at](https://www.affymetrix.com/LinkServlet?probeset=1460011_at) | [Cyp26b1](http://www.ncbi.nlm.nih.gov/entrez/query.fcgi?cmd=search&db=gene&term=Cyp26b1) | cytochrome P450, family 26, subfamily b, polypeptide 1 |
| 1431 | 0,0008715 | 0,012 | 570,68 | 192,49 | 2,96 | [1416662_at](https://www.affymetrix.com/LinkServlet?probeset=1416662_at) | [Sardh](http://www.ncbi.nlm.nih.gov/entrez/query.fcgi?cmd=search&db=gene&term=Sardh) | sarcosine dehydrogenase |
| 1432 | 0,0008723 | 0,012 | 79,94 | 7,02 | 11,38 | [1423213_at](https://www.affymetrix.com/LinkServlet?probeset=1423213_at) | [Plxnc1](http://www.ncbi.nlm.nih.gov/entrez/query.fcgi?cmd=search&db=gene&term=Plxnc1) | plexin C1 |
| 1433 | 0,0008741 | 0,012 | 2984,61 | 325,95 | 9,16 | [1449264_at](https://www.affymetrix.com/LinkServlet?probeset=1449264_at) | [Syt11](http://www.ncbi.nlm.nih.gov/entrez/query.fcgi?cmd=search&db=gene&term=Syt11) | synaptotagmin XI |
| 1434 | 0,0008741 | 0,012 | 6714,17 | 4313,36 | 1,56 | [1417311_at](https://www.affymetrix.com/LinkServlet?probeset=1417311_at) | [Crip2](http://www.ncbi.nlm.nih.gov/entrez/query.fcgi?cmd=search&db=gene&term=Crip2) | cysteine rich protein 2 |
| 1435 | 0,0008754 | 0,012 | 717,92 | 65,51 | 10,96 | [1424757_at](https://www.affymetrix.com/LinkServlet?probeset=1424757_at) | [BC018242](http://www.ncbi.nlm.nih.gov/entrez/query.fcgi?cmd=search&db=gene&term=BC018242) | cDNA sequence BC018242 |
| 1436 | 0,0008754 | 0,012 | 649,51 | 939,67 | 0,69 | [1428806_at](https://www.affymetrix.com/LinkServlet?probeset=1428806_at) | [Csnk1g1](http://www.ncbi.nlm.nih.gov/entrez/query.fcgi?cmd=search&db=gene&term=Csnk1g1) | casein kinase 1, gamma 1 |
| 1437 | 0,0008761 | 0,012 | 1660,21 | 1121,33 | 1,48 | [1423651_at](https://www.affymetrix.com/LinkServlet?probeset=1423651_at) | [Isca1](http://www.ncbi.nlm.nih.gov/entrez/query.fcgi?cmd=search&db=gene&term=Isca1) | iron-sulfur cluster assembly 1 homolog (S. cerevisiae) |
| 1438 | 0,0008786 | 0,012 | 1072,23 | 2005,78 | 0,53 | [1433599_at](https://www.affymetrix.com/LinkServlet?probeset=1433599_at) | [Baz1a](http://www.ncbi.nlm.nih.gov/entrez/query.fcgi?cmd=search&db=gene&term=Baz1a) | bromodomain adjacent to zinc finger domain 1A |
| 1439 | 0,0008795 | 0,012 | 265,52 | 54,18 | 4,9 | [1435587_at](https://www.affymetrix.com/LinkServlet?probeset=1435587_at) | [Pcid2](http://www.ncbi.nlm.nih.gov/entrez/query.fcgi?cmd=search&db=gene&term=Pcid2) | PCI domain containing 2 |
| 1440 | 0,0008803 | 0,012 | 454,95 | 167,94 | 2,71 | [1439623_at](https://www.affymetrix.com/LinkServlet?probeset=1439623_at) | [NA](http://www.ncbi.nlm.nih.gov/entrez/query.fcgi?cmd=search&db=gene&term=NA) | NA |
| 1441 | 0,0008823 | 0,012 | 8346,15 | 5861,74 | 1,42 | [1434646_s_at](https://www.affymetrix.com/LinkServlet?probeset=1434646_s_at) | [NA](http://www.ncbi.nlm.nih.gov/entrez/query.fcgi?cmd=search&db=gene&term=NA) | NA |
| 1442 | 0,0008838 | 0,0121 | 1125,13 | 503,65 | 2,23 | [1448802_at](https://www.affymetrix.com/LinkServlet?probeset=1448802_at) | [Nufip1](http://www.ncbi.nlm.nih.gov/entrez/query.fcgi?cmd=search&db=gene&term=Nufip1) | nuclear fragile X mental retardation protein interacting protein 1 |
| 1443 | 0,0008853 | 0,0121 | 199,6 | 12,95 | 15,41 | [1422015_a_at](https://www.affymetrix.com/LinkServlet?probeset=1422015_a_at) | [Abcb8](http://www.ncbi.nlm.nih.gov/entrez/query.fcgi?cmd=search&db=gene&term=Abcb8) | ATP-binding cassette, sub-family B (MDR/TAP), member 8 |
| 1444 | 0,0008875 | 0,0121 | 1448,88 | 457,62 | 3,17 | [1418965_at](https://www.affymetrix.com/LinkServlet?probeset=1418965_at) | [Nosip](http://www.ncbi.nlm.nih.gov/entrez/query.fcgi?cmd=search&db=gene&term=Nosip) | nitric oxide synthase interacting protein |
| 1445 | 0,0008878 | 0,0121 | 841,04 | 1785,05 | 0,47 | [1423825_at](https://www.affymetrix.com/LinkServlet?probeset=1423825_at) | [Wls](http://www.ncbi.nlm.nih.gov/entrez/query.fcgi?cmd=search&db=gene&term=Wls) | wntless homolog (Drosophila) |
| 1446 | 0,000888 | 0,0121 | 553,27 | 2231,02 | 0,25 | [1449152_at](https://www.affymetrix.com/LinkServlet?probeset=1449152_at) | [Cdkn2b](http://www.ncbi.nlm.nih.gov/entrez/query.fcgi?cmd=search&db=gene&term=Cdkn2b) | cyclin-dependent kinase inhibitor 2B (p15, inhibits CDK4) |
| 1447 | 0,0008881 | 0,0121 | 5413,27 | 3475,57 | 1,56 | [1420093_s_at](https://www.affymetrix.com/LinkServlet?probeset=1420093_s_at) | [Hnrpdl](http://www.ncbi.nlm.nih.gov/entrez/query.fcgi?cmd=search&db=gene&term=Hnrpdl) | heterogeneous nuclear ribonucleoprotein D-like |
| 1448 | 0,0008916 | 0,0121 | 405,27 | 6,56 | 61,79 | [1426085_a_at](https://www.affymetrix.com/LinkServlet?probeset=1426085_a_at) | [Pxn](http://www.ncbi.nlm.nih.gov/entrez/query.fcgi?cmd=search&db=gene&term=Pxn) | paxillin |
| 1449 | 0,0008927 | 0,0121 | 27,47 | 12,57 | 2,18 | [1452875_at](https://www.affymetrix.com/LinkServlet?probeset=1452875_at) | [Hddc3](http://www.ncbi.nlm.nih.gov/entrez/query.fcgi?cmd=search&db=gene&term=Hddc3) | HD domain containing 3 |
| 1450 | 0,0008928 | 0,0121 | 8,29 | 68,22 | 0,12 | [1427669_a_at](https://www.affymetrix.com/LinkServlet?probeset=1427669_a_at) | [Cit](http://www.ncbi.nlm.nih.gov/entrez/query.fcgi?cmd=search&db=gene&term=Cit) | citron |
| 1451 | 0,0008962 | 0,0121 | 390,93 | 205,3 | 1,9 | [1426822_at](https://www.affymetrix.com/LinkServlet?probeset=1426822_at) | [Rhot2](http://www.ncbi.nlm.nih.gov/entrez/query.fcgi?cmd=search&db=gene&term=Rhot2) | ras homolog gene family, member T2 |
| 1452 | 0,0008963 | 0,0121 | 3229,56 | 1672,09 | 1,93 | [1423865_at](https://www.affymetrix.com/LinkServlet?probeset=1423865_at) | [Slc44a1](http://www.ncbi.nlm.nih.gov/entrez/query.fcgi?cmd=search&db=gene&term=Slc44a1) | solute carrier family 44, member 1 |
| 1453 | 0,0008965 | 0,0121 | 91,92 | 474,36 | 0,19 | [1451204_at](https://www.affymetrix.com/LinkServlet?probeset=1451204_at) | [Scara5](http://www.ncbi.nlm.nih.gov/entrez/query.fcgi?cmd=search&db=gene&term=Scara5) | scavenger receptor class A, member 5 (putative) |
| 1454 | 0,0009027 | 0,0122 | 284,31 | 42,76 | 6,65 | [1438451_at](https://www.affymetrix.com/LinkServlet?probeset=1438451_at) | [Arhgap32](http://www.ncbi.nlm.nih.gov/entrez/query.fcgi?cmd=search&db=gene&term=Arhgap32) | Rho GTPase activating protein 32 |
| 1455 | 0,0009035 | 0,0122 | 30,7 | 63,91 | 0,48 | [1440078_at](https://www.affymetrix.com/LinkServlet?probeset=1440078_at) | [NA](http://www.ncbi.nlm.nih.gov/entrez/query.fcgi?cmd=search&db=gene&term=NA) | NA |
| 1456 | 0,0009043 | 0,0122 | 63,76 | 606,05 | 0,11 | [1415776_at](https://www.affymetrix.com/LinkServlet?probeset=1415776_at) | [Aldh3a2](http://www.ncbi.nlm.nih.gov/entrez/query.fcgi?cmd=search&db=gene&term=Aldh3a2) | aldehyde dehydrogenase family 3, subfamily A2 |
| 1457 | 0,0009045 | 0,0122 | 7,48 | 92,97 | 0,08 | [1438130_at](https://www.affymetrix.com/LinkServlet?probeset=1438130_at) | [Taf15](http://www.ncbi.nlm.nih.gov/entrez/query.fcgi?cmd=search&db=gene&term=Taf15) | TAF15 RNA polymerase II, TATA box binding protein (TBP)-associated factor |
| 1458 | 0,000905 | 0,0122 | 483,1 | 1891,52 | 0,26 | [1438663_at](https://www.affymetrix.com/LinkServlet?probeset=1438663_at) | [Prrc2c](http://www.ncbi.nlm.nih.gov/entrez/query.fcgi?cmd=search&db=gene&term=Prrc2c) | proline-rich coiled-coil 2C |
| 1459 | 0,0009062 | 0,0122 | 5,1 | 13,81 | 0,37 | [1452477_at](https://www.affymetrix.com/LinkServlet?probeset=1452477_at) | [NA](http://www.ncbi.nlm.nih.gov/entrez/query.fcgi?cmd=search&db=gene&term=NA) | NA |
| 1460 | 0,0009071 | 0,0122 | 457,14 | 871,6 | 0,52 | [1433684_at](https://www.affymetrix.com/LinkServlet?probeset=1433684_at) | [Chmp6](http://www.ncbi.nlm.nih.gov/entrez/query.fcgi?cmd=search&db=gene&term=Chmp6) | chromatin modifying protein 6 |
| 1461 | 0,0009078 | 0,0122 | 5,53 | 212,6 | 0,026 | [1439036_a_at](https://www.affymetrix.com/LinkServlet?probeset=1439036_a_at) | [Atp1b1](http://www.ncbi.nlm.nih.gov/entrez/query.fcgi?cmd=search&db=gene&term=Atp1b1) | ATPase, Na+/K+ transporting, beta 1 polypeptide |
| 1462 | 0,0009085 | 0,0122 | 907,91 | 474,22 | 1,91 | [1420505_a_at](https://www.affymetrix.com/LinkServlet?probeset=1420505_a_at) | [Stxbp1](http://www.ncbi.nlm.nih.gov/entrez/query.fcgi?cmd=search&db=gene&term=Stxbp1) | syntaxin binding protein 1 |
| 1463 | 0,0009088 | 0,0122 | 71,67 | 32,51 | 2,2 | [1425135_a_at](https://www.affymetrix.com/LinkServlet?probeset=1425135_a_at) | [Dnm2](http://www.ncbi.nlm.nih.gov/entrez/query.fcgi?cmd=search&db=gene&term=Dnm2) | dynamin 2 |
| 1464 | 0,0009095 | 0,0122 | 4865,09 | 1289,72 | 3,77 | [1455176_a_at](https://www.affymetrix.com/LinkServlet?probeset=1455176_a_at) | [Syt11](http://www.ncbi.nlm.nih.gov/entrez/query.fcgi?cmd=search&db=gene&term=Syt11) | synaptotagmin XI |
| 1465 | 0,0009101 | 0,0122 | 35,91 | 110,06 | 0,33 | [1424761_at](https://www.affymetrix.com/LinkServlet?probeset=1424761_at) | [Fam115c](http://www.ncbi.nlm.nih.gov/entrez/query.fcgi?cmd=search&db=gene&term=Fam115c) | family with sequence similarity 115, member C |
| 1466 | 0,0009103 | 0,0122 | 3334,54 | 1589,29 | 2,1 | [1424619_at](https://www.affymetrix.com/LinkServlet?probeset=1424619_at) | [Sf3b4](http://www.ncbi.nlm.nih.gov/entrez/query.fcgi?cmd=search&db=gene&term=Sf3b4) | splicing factor 3b, subunit 4 |
| 1467 | 0,0009108 | 0,0122 | 835,4 | 178,03 | 4,69 | [1416759_at](https://www.affymetrix.com/LinkServlet?probeset=1416759_at) | [Mical1](http://www.ncbi.nlm.nih.gov/entrez/query.fcgi?cmd=search&db=gene&term=Mical1) | microtubule associated monoxygenase, calponin and LIM domain containing 1 |
| 1468 | 0,0009126 | 0,0122 | 113,67 | 37,32 | 3,05 | [1455769_at](https://www.affymetrix.com/LinkServlet?probeset=1455769_at) | [NA](http://www.ncbi.nlm.nih.gov/entrez/query.fcgi?cmd=search&db=gene&term=NA) | NA |
| 1469 | 0,0009138 | 0,0122 | 108,45 | 478,22 | 0,23 | [1457265_at](https://www.affymetrix.com/LinkServlet?probeset=1457265_at) | [Akap17b](http://www.ncbi.nlm.nih.gov/entrez/query.fcgi?cmd=search&db=gene&term=Akap17b) | A kinase (PRKA) anchor protein 17B |
| 1470 | 0,0009139 | 0,0122 | 26,87 | 745,28 | 0,036 | [1416779_at](https://www.affymetrix.com/LinkServlet?probeset=1416779_at) | [Sdpr](http://www.ncbi.nlm.nih.gov/entrez/query.fcgi?cmd=search&db=gene&term=Sdpr) | serum deprivation response |
| 1471 | 0,000914 | 0,0122 | 896,84 | 307,93 | 2,91 | [1426046_a_at](https://www.affymetrix.com/LinkServlet?probeset=1426046_a_at) | [Rabggta](http://www.ncbi.nlm.nih.gov/entrez/query.fcgi?cmd=search&db=gene&term=Rabggta) | Rab geranylgeranyl transferase, a subunit |
| 1472 | 0,000916 | 0,0122 | 23762,69 | 2892,81 | 8,21 | [1426114_at](https://www.affymetrix.com/LinkServlet?probeset=1426114_at) | [Hnrnpab](http://www.ncbi.nlm.nih.gov/entrez/query.fcgi?cmd=search&db=gene&term=Hnrnpab) | heterogeneous nuclear ribonucleoprotein A/B |
| 1473 | 0,0009168 | 0,0122 | 397,08 | 50302,49 | 0,0079 | [1418188_a_at](https://www.affymetrix.com/LinkServlet?probeset=1418188_a_at) | [NA](http://www.ncbi.nlm.nih.gov/entrez/query.fcgi?cmd=search&db=gene&term=NA) | NA |
| 1474 | 0,0009183 | 0,0123 | 970 | 2862,53 | 0,34 | [1456390_at](https://www.affymetrix.com/LinkServlet?probeset=1456390_at) | [Ppp2ca](http://www.ncbi.nlm.nih.gov/entrez/query.fcgi?cmd=search&db=gene&term=Ppp2ca) | protein phosphatase 2 (formerly 2A), catalytic subunit, alpha isoform |
| 1475 | 0,0009203 | 0,0123 | 1161,87 | 1674,87 | 0,69 | [1428944_at](https://www.affymetrix.com/LinkServlet?probeset=1428944_at) | [Uba6](http://www.ncbi.nlm.nih.gov/entrez/query.fcgi?cmd=search&db=gene&term=Uba6) | ubiquitin-like modifier activating enzyme 6 |
| 1476 | 0,000921 | 0,0123 | 1830,81 | 3628,43 | 0,5 | [1452247_at](https://www.affymetrix.com/LinkServlet?probeset=1452247_at) | [Fxr1](http://www.ncbi.nlm.nih.gov/entrez/query.fcgi?cmd=search&db=gene&term=Fxr1) | fragile X mental retardation gene 1, autosomal homolog |
| 1477 | 0,0009216 | 0,0123 | 802,52 | 1105,51 | 0,73 | [1424277_at](https://www.affymetrix.com/LinkServlet?probeset=1424277_at) | [Haus7](http://www.ncbi.nlm.nih.gov/entrez/query.fcgi?cmd=search&db=gene&term=Haus7) | HAUS augmin-like complex, subunit 7 |
| 1478 | 0,0009221 | 0,0123 | 42,72 | 620,76 | 0,069 | [1424588_at](https://www.affymetrix.com/LinkServlet?probeset=1424588_at) | [Srgap3](http://www.ncbi.nlm.nih.gov/entrez/query.fcgi?cmd=search&db=gene&term=Srgap3) | SLIT-ROBO Rho GTPase activating protein 3 |
| 1479 | 0,0009224 | 0,0123 | 777,51 | 274,07 | 2,84 | [1424622_at](https://www.affymetrix.com/LinkServlet?probeset=1424622_at) | [Hsf1](http://www.ncbi.nlm.nih.gov/entrez/query.fcgi?cmd=search&db=gene&term=Hsf1) | heat shock factor 1 |
| 1480 | 0,0009237 | 0,0123 | 3307,62 | 27,8 | 118,99 | [1429993_s_at](https://www.affymetrix.com/LinkServlet?probeset=1429993_s_at) | [NA](http://www.ncbi.nlm.nih.gov/entrez/query.fcgi?cmd=search&db=gene&term=NA) | NA |
| 1481 | 0,0009241 | 0,0123 | 1064 | 197,77 | 5,38 | [1452141_a_at](https://www.affymetrix.com/LinkServlet?probeset=1452141_a_at) | [Sepp1](http://www.ncbi.nlm.nih.gov/entrez/query.fcgi?cmd=search&db=gene&term=Sepp1) | selenoprotein P, plasma, 1 |
| 1482 | 0,00093 | 0,0123 | 522,88 | 106,31 | 4,92 | [1436341_at](https://www.affymetrix.com/LinkServlet?probeset=1436341_at) | [NA](http://www.ncbi.nlm.nih.gov/entrez/query.fcgi?cmd=search&db=gene&term=NA) | NA |
| 1483 | 0,0009305 | 0,0123 | 114,5 | 404,98 | 0,28 | [1431035_at](https://www.affymetrix.com/LinkServlet?probeset=1431035_at) | [Daam1](http://www.ncbi.nlm.nih.gov/entrez/query.fcgi?cmd=search&db=gene&term=Daam1) | dishevelled associated activator of morphogenesis 1 |
| 1484 | 0,0009314 | 0,0123 | 690,68 | 419,11 | 1,65 | [1417955_at](https://www.affymetrix.com/LinkServlet?probeset=1417955_at) | [Ccdc71](http://www.ncbi.nlm.nih.gov/entrez/query.fcgi?cmd=search&db=gene&term=Ccdc71) | coiled-coil domain containing 71 |
| 1485 | 0,0009334 | 0,0124 | 31,26 | 607,86 | 0,051 | [1438973_x_at](https://www.affymetrix.com/LinkServlet?probeset=1438973_x_at) | [Gja1](http://www.ncbi.nlm.nih.gov/entrez/query.fcgi?cmd=search&db=gene&term=Gja1) | gap junction protein, alpha 1 |
| 1486 | 0,0009356 | 0,0124 | 220,18 | 84,07 | 2,62 | [1425016_at](https://www.affymetrix.com/LinkServlet?probeset=1425016_at) | [Ephb2](http://www.ncbi.nlm.nih.gov/entrez/query.fcgi?cmd=search&db=gene&term=Ephb2) | Eph receptor B2 |
| 1487 | 0,0009365 | 0,0124 | 1555,6 | 2311,57 | 0,67 | [1454711_at](https://www.affymetrix.com/LinkServlet?probeset=1454711_at) | [Trio](http://www.ncbi.nlm.nih.gov/entrez/query.fcgi?cmd=search&db=gene&term=Trio) | triple functional domain (PTPRF interacting) |
| 1488 | 0,0009383 | 0,0124 | 214,98 | 87,39 | 2,46 | [1421646_a_at](https://www.affymetrix.com/LinkServlet?probeset=1421646_a_at) | [Pias3](http://www.ncbi.nlm.nih.gov/entrez/query.fcgi?cmd=search&db=gene&term=Pias3) | protein inhibitor of activated STAT 3 |
| 1489 | 0,0009396 | 0,0124 | 144,17 | 661,4 | 0,22 | [1433471_at](https://www.affymetrix.com/LinkServlet?probeset=1433471_at) | [Tcf7](http://www.ncbi.nlm.nih.gov/entrez/query.fcgi?cmd=search&db=gene&term=Tcf7) | transcription factor 7, T-cell specific |
| 1490 | 0,0009403 | 0,0124 | 903,03 | 48,67 | 18,55 | [1420975_at](https://www.affymetrix.com/LinkServlet?probeset=1420975_at) | [Baz1b](http://www.ncbi.nlm.nih.gov/entrez/query.fcgi?cmd=search&db=gene&term=Baz1b) | bromodomain adjacent to zinc finger domain, 1B |
| 1491 | 0,0009415 | 0,0124 | 63,56 | 130,62 | 0,49 | [1446325_at](https://www.affymetrix.com/LinkServlet?probeset=1446325_at) | [Pcyox1](http://www.ncbi.nlm.nih.gov/entrez/query.fcgi?cmd=search&db=gene&term=Pcyox1) | prenylcysteine oxidase 1 |
| 1492 | 0,0009422 | 0,0124 | 290,18 | 529,06 | 0,55 | [1452009_at](https://www.affymetrix.com/LinkServlet?probeset=1452009_at) | [Ttc39b](http://www.ncbi.nlm.nih.gov/entrez/query.fcgi?cmd=search&db=gene&term=Ttc39b) | tetratricopeptide repeat domain 39B |
| 1493 | 0,0009425 | 0,0124 | 10,45 | 18,61 | 0,56 | [1439210_at](https://www.affymetrix.com/LinkServlet?probeset=1439210_at) | [Mrps9](http://www.ncbi.nlm.nih.gov/entrez/query.fcgi?cmd=search&db=gene&term=Mrps9) | mitochondrial ribosomal protein S9 |
| 1494 | 0,0009435 | 0,0124 | 411,05 | 116,53 | 3,53 | [1448490_at](https://www.affymetrix.com/LinkServlet?probeset=1448490_at) | [Adck4](http://www.ncbi.nlm.nih.gov/entrez/query.fcgi?cmd=search&db=gene&term=Adck4) | aarF domain containing kinase 4 |
| 1495 | 0,0009437 | 0,0124 | 7,21 | 5,38 | 1,34 | [1431180_at](https://www.affymetrix.com/LinkServlet?probeset=1431180_at) | [Zfp433](http://www.ncbi.nlm.nih.gov/entrez/query.fcgi?cmd=search&db=gene&term=Zfp433) | RIKEN cDNA 1700123A16 gene |
| 1496 | 0,0009442 | 0,0124 | 55,13 | 16,97 | 3,25 | [1425118_at](https://www.affymetrix.com/LinkServlet?probeset=1425118_at) | [Spire2](http://www.ncbi.nlm.nih.gov/entrez/query.fcgi?cmd=search&db=gene&term=Spire2) | spire homolog 2 (Drosophila) |
| 1497 | 0,0009446 | 0,0124 | 11,13 | 45,39 | 0,25 | [1439532_s_at](https://www.affymetrix.com/LinkServlet?probeset=1439532_s_at) | [Kif13a](http://www.ncbi.nlm.nih.gov/entrez/query.fcgi?cmd=search&db=gene&term=Kif13a) | kinesin family member 13A |
| 1498 | 0,0009453 | 0,0124 | 5371,68 | 1681,58 | 3,19 | [1420760_s_at](https://www.affymetrix.com/LinkServlet?probeset=1420760_s_at) | [Ndrg1](http://www.ncbi.nlm.nih.gov/entrez/query.fcgi?cmd=search&db=gene&term=Ndrg1) | N-myc downstream regulated gene 1 |
| 1499 | 0,0009476 | 0,0124 | 31,68 | 452,5 | 0,07 | [1455786_at](https://www.affymetrix.com/LinkServlet?probeset=1455786_at) | [Zfp820](http://www.ncbi.nlm.nih.gov/entrez/query.fcgi?cmd=search&db=gene&term=Zfp820) | zinc finger protein 820 |
| 1500 | 0,0009483 | 0,0124 | 426,3 | 39,87 | 10,69 | [1421324_a_at](https://www.affymetrix.com/LinkServlet?probeset=1421324_a_at) | [Akt2](http://www.ncbi.nlm.nih.gov/entrez/query.fcgi?cmd=search&db=gene&term=Akt2) | thymoma viral proto-oncogene 2 |
| 1501 | 0,0009483 | 0,0124 | 72,67 | 8,14 | 8,93 | [1431208_a_at](https://www.affymetrix.com/LinkServlet?probeset=1431208_a_at) | [Slc9a3r2](http://www.ncbi.nlm.nih.gov/entrez/query.fcgi?cmd=search&db=gene&term=Slc9a3r2) | solute carrier family 9 (sodium/hydrogen exchanger), member 3 regulator 2 |
| 1502 | 0,0009493 | 0,0124 | 661,23 | 45,43 | 14,56 | [1451775_s_at](https://www.affymetrix.com/LinkServlet?probeset=1451775_s_at) | [Il13ra1](http://www.ncbi.nlm.nih.gov/entrez/query.fcgi?cmd=search&db=gene&term=Il13ra1) | interleukin 13 receptor, alpha 1 |
| 1503 | 0,0009497 | 0,0124 | 1735,34 | 983,09 | 1,77 | [1452739_at](https://www.affymetrix.com/LinkServlet?probeset=1452739_at) | [Fbxo7](http://www.ncbi.nlm.nih.gov/entrez/query.fcgi?cmd=search&db=gene&term=Fbxo7) | F-box protein 7 |
| 1504 | 0,0009512 | 0,0124 | 57,28 | 167,26 | 0,34 | [1457279_at](https://www.affymetrix.com/LinkServlet?probeset=1457279_at) | [NA](http://www.ncbi.nlm.nih.gov/entrez/query.fcgi?cmd=search&db=gene&term=NA) | NA |
| 1505 | 0,0009513 | 0,0124 | 93,01 | 4,4 | 21,13 | [1452448_at](https://www.affymetrix.com/LinkServlet?probeset=1452448_at) | [Aqr](http://www.ncbi.nlm.nih.gov/entrez/query.fcgi?cmd=search&db=gene&term=Aqr) | aquarius |
| 1506 | 0,0009527 | 0,0124 | 95,23 | 13,77 | 6,92 | [1418082_at](https://www.affymetrix.com/LinkServlet?probeset=1418082_at) | [Nmt1](http://www.ncbi.nlm.nih.gov/entrez/query.fcgi?cmd=search&db=gene&term=Nmt1) | N-myristoyltransferase 1 |
| 1507 | 0,0009533 | 0,0124 | 38,74 | 352,09 | 0,11 | [1449154_at](https://www.affymetrix.com/LinkServlet?probeset=1449154_at) | [Col11a1](http://www.ncbi.nlm.nih.gov/entrez/query.fcgi?cmd=search&db=gene&term=Col11a1) | collagen, type XI, alpha 1 |
| 1508 | 0,0009549 | 0,0124 | 149,63 | 3462,77 | 0,043 | [1438650_x_at](https://www.affymetrix.com/LinkServlet?probeset=1438650_x_at) | [Gja1](http://www.ncbi.nlm.nih.gov/entrez/query.fcgi?cmd=search&db=gene&term=Gja1) | gap junction protein, alpha 1 |
| 1509 | 0,0009551 | 0,0124 | 909,27 | 484,77 | 1,88 | [1453087_at](https://www.affymetrix.com/LinkServlet?probeset=1453087_at) | [6330403L08Rik](http://www.ncbi.nlm.nih.gov/entrez/query.fcgi?cmd=search&db=gene&term=6330403L08Rik) | RIKEN cDNA 6330403L08 gene |
| 1510 | 0,0009566 | 0,0125 | 449,42 | 137,57 | 3,27 | [1424082_at](https://www.affymetrix.com/LinkServlet?probeset=1424082_at) | [Tbc1d13](http://www.ncbi.nlm.nih.gov/entrez/query.fcgi?cmd=search&db=gene&term=Tbc1d13) | TBC1 domain family, member 13 |
| 1511 | 0,0009605 | 0,0125 | 561,08 | 99,61 | 5,63 | [1439797_at](https://www.affymetrix.com/LinkServlet?probeset=1439797_at) | [Ppard](http://www.ncbi.nlm.nih.gov/entrez/query.fcgi?cmd=search&db=gene&term=Ppard) | peroxisome proliferator activator receptor delta |
| 1512 | 0,0009625 | 0,0125 | 4329,01 | 8340,24 | 0,52 | [1436157_at](https://www.affymetrix.com/LinkServlet?probeset=1436157_at) | [Ccar1](http://www.ncbi.nlm.nih.gov/entrez/query.fcgi?cmd=search&db=gene&term=Ccar1) | cell division cycle and apoptosis regulator 1 |
| 1513 | 0,0009644 | 0,0125 | 10,32 | 24,92 | 0,41 | [1425513_at](https://www.affymetrix.com/LinkServlet?probeset=1425513_at) | [Map2k7](http://www.ncbi.nlm.nih.gov/entrez/query.fcgi?cmd=search&db=gene&term=Map2k7) | mitogen-activated protein kinase kinase 7 |
| 1514 | 0,0009647 | 0,0125 | 51,51 | 468,4 | 0,11 | [1449038_at](https://www.affymetrix.com/LinkServlet?probeset=1449038_at) | [Hsd11b1](http://www.ncbi.nlm.nih.gov/entrez/query.fcgi?cmd=search&db=gene&term=Hsd11b1) | hydroxysteroid 11-beta dehydrogenase 1 |
| 1515 | 0,0009648 | 0,0125 | 469,86 | 2647,5 | 0,18 | [1434411_at](https://www.affymetrix.com/LinkServlet?probeset=1434411_at) | [Col12a1](http://www.ncbi.nlm.nih.gov/entrez/query.fcgi?cmd=search&db=gene&term=Col12a1) | collagen, type XII, alpha 1 |
| 1516 | 0,0009666 | 0,0125 | 39,32 | 108,34 | 0,36 | [1456232_at](https://www.affymetrix.com/LinkServlet?probeset=1456232_at) | [Gmeb1](http://www.ncbi.nlm.nih.gov/entrez/query.fcgi?cmd=search&db=gene&term=Gmeb1) | glucocorticoid modulatory element binding protein 1 |
| 1517 | 0,0009667 | 0,0125 | 5438,9 | 1538,35 | 3,54 | [1427903_at](https://www.affymetrix.com/LinkServlet?probeset=1427903_at) | [Phpt1](http://www.ncbi.nlm.nih.gov/entrez/query.fcgi?cmd=search&db=gene&term=Phpt1) | phosphohistidine phosphatase 1 |
| 1518 | 0,0009678 | 0,0125 | 750,53 | 1589,96 | 0,47 | [1418371_at](https://www.affymetrix.com/LinkServlet?probeset=1418371_at) | [Dynll2](http://www.ncbi.nlm.nih.gov/entrez/query.fcgi?cmd=search&db=gene&term=Dynll2) | dynein light chain LC8-type 2 |
| 1519 | 0,0009679 | 0,0125 | 1776,82 | 5966,71 | 0,3 | [1416028_a_at](https://www.affymetrix.com/LinkServlet?probeset=1416028_a_at) | [Hn1](http://www.ncbi.nlm.nih.gov/entrez/query.fcgi?cmd=search&db=gene&term=Hn1) | hematological and neurological expressed sequence 1 |
| 1520 | 0,0009684 | 0,0125 | 186,34 | 21,92 | 8,5 | [1422321_a_at](https://www.affymetrix.com/LinkServlet?probeset=1422321_a_at) | [Sf1](http://www.ncbi.nlm.nih.gov/entrez/query.fcgi?cmd=search&db=gene&term=Sf1) | splicing factor 1 |
| 1521 | 0,000972 | 0,0126 | 13,51 | 100,56 | 0,13 | [1438726_at](https://www.affymetrix.com/LinkServlet?probeset=1438726_at) | [Mical2](http://www.ncbi.nlm.nih.gov/entrez/query.fcgi?cmd=search&db=gene&term=Mical2) | microtubule associated monoxygenase, calponin and LIM domain containing 2 |
| 1522 | 0,0009746 | 0,0126 | 2424,96 | 5429,66 | 0,45 | [1419812_s_at](https://www.affymetrix.com/LinkServlet?probeset=1419812_s_at) | [Ccdc56](http://www.ncbi.nlm.nih.gov/entrez/query.fcgi?cmd=search&db=gene&term=Ccdc56) | coiled-coil domain containing 56 |
| 1523 | 0,0009757 | 0,0126 | 1445,6 | 380,09 | 3,8 | [1441750_x_at](https://www.affymetrix.com/LinkServlet?probeset=1441750_x_at) | [4930447F24Rik](http://www.ncbi.nlm.nih.gov/entrez/query.fcgi?cmd=search&db=gene&term=4930447F24Rik) | RIKEN cDNA 4930447F24 gene |
| 1524 | 0,0009776 | 0,0126 | 950,77 | 521,76 | 1,82 | [1424399_at](https://www.affymetrix.com/LinkServlet?probeset=1424399_at) | [Uck1](http://www.ncbi.nlm.nih.gov/entrez/query.fcgi?cmd=search&db=gene&term=Uck1) | uridine-cytidine kinase 1 |
| 1525 | 0,0009806 | 0,0126 | 99,22 | 31,72 | 3,13 | [1415989_at](https://www.affymetrix.com/LinkServlet?probeset=1415989_at) | [Vcam1](http://www.ncbi.nlm.nih.gov/entrez/query.fcgi?cmd=search&db=gene&term=Vcam1) | vascular cell adhesion molecule 1 |
| 1526 | 0,0009807 | 0,0126 | 1312,31 | 228,68 | 5,74 | [1428393_at](https://www.affymetrix.com/LinkServlet?probeset=1428393_at) | [Nrn1](http://www.ncbi.nlm.nih.gov/entrez/query.fcgi?cmd=search&db=gene&term=Nrn1) | neuritin 1 |
| 1527 | 0,0009814 | 0,0126 | 39,85 | 18,11 | 2,2 | [1423794_at](https://www.affymetrix.com/LinkServlet?probeset=1423794_at) | [Atg13](http://www.ncbi.nlm.nih.gov/entrez/query.fcgi?cmd=search&db=gene&term=Atg13) | ATG13 autophagy related 13 homolog (S. cerevisiae) |
| 1528 | 0,0009816 | 0,0126 | 89,65 | 232,81 | 0,39 | [1455101_at](https://www.affymetrix.com/LinkServlet?probeset=1455101_at) | [Phactr2](http://www.ncbi.nlm.nih.gov/entrez/query.fcgi?cmd=search&db=gene&term=Phactr2) | phosphatase and actin regulator 2 |
| 1529 | 0,0009818 | 0,0126 | 7994,57 | 10895,64 | 0,73 | [1425194_a_at](https://www.affymetrix.com/LinkServlet?probeset=1425194_a_at) | [6330577E15Rik](http://www.ncbi.nlm.nih.gov/entrez/query.fcgi?cmd=search&db=gene&term=6330577E15Rik) | RIKEN cDNA 6330577E15 gene |
| 1530 | 0,0009848 | 0,0127 | 170,59 | 287,2 | 0,59 | [1437357_at](https://www.affymetrix.com/LinkServlet?probeset=1437357_at) | [Ythdc2](http://www.ncbi.nlm.nih.gov/entrez/query.fcgi?cmd=search&db=gene&term=Ythdc2) | YTH domain containing 2 |
| 1531 | 0,0009858 | 0,0127 | 1110,83 | 79,43 | 13,98 | [1450097_s_at](https://www.affymetrix.com/LinkServlet?probeset=1450097_s_at) | [Gna12](http://www.ncbi.nlm.nih.gov/entrez/query.fcgi?cmd=search&db=gene&term=Gna12) | guanine nucleotide binding protein, alpha 12 |
| 1532 | 0,0009892 | 0,0127 | 62,12 | 364,14 | 0,17 | [1422005_at](https://www.affymetrix.com/LinkServlet?probeset=1422005_at) | [Eif2ak2](http://www.ncbi.nlm.nih.gov/entrez/query.fcgi?cmd=search&db=gene&term=Eif2ak2) | eukaryotic translation initiation factor 2-alpha kinase 2 |
| 1533 | 0,0009892 | 0,0127 | 297,15 | 76,08 | 3,91 | [1454211_a_at](https://www.affymetrix.com/LinkServlet?probeset=1454211_a_at) | [Shroom3](http://www.ncbi.nlm.nih.gov/entrez/query.fcgi?cmd=search&db=gene&term=Shroom3) | shroom family member 3 |
| 1534 | 0,0009893 | 0,0127 | 910,47 | 22420,3 | 0,041 | [1419734_at](https://www.affymetrix.com/LinkServlet?probeset=1419734_at) | [Actb](http://www.ncbi.nlm.nih.gov/entrez/query.fcgi?cmd=search&db=gene&term=Actb) | actin, beta |
| 1535 | 0,0009908 | 0,0127 | 248,02 | 582,61 | 0,43 | [1437714_x_at](https://www.affymetrix.com/LinkServlet?probeset=1437714_x_at) | [Usp14](http://www.ncbi.nlm.nih.gov/entrez/query.fcgi?cmd=search&db=gene&term=Usp14) | ubiquitin specific peptidase 14 |
| 1536 | 0,0009919 | 0,0127 | 5,98 | 53,36 | 0,11 | [1435645_at](https://www.affymetrix.com/LinkServlet?probeset=1435645_at) | [Mmd](http://www.ncbi.nlm.nih.gov/entrez/query.fcgi?cmd=search&db=gene&term=Mmd) | monocyte to macrophage differentiation-associated |
| 1537 | 0,000992 | 0,0127 | 2037,71 | 815,68 | 2,5 | [1428850_x_at](https://www.affymetrix.com/LinkServlet?probeset=1428850_x_at) | [Cd99](http://www.ncbi.nlm.nih.gov/entrez/query.fcgi?cmd=search&db=gene&term=Cd99) | CD99 antigen |
| 1538 | 0,0009921 | 0,0127 | 253,29 | 106,62 | 2,38 | [1438964_x_at](https://www.affymetrix.com/LinkServlet?probeset=1438964_x_at) | [Tfpt](http://www.ncbi.nlm.nih.gov/entrez/query.fcgi?cmd=search&db=gene&term=Tfpt) | TCF3 (E2A) fusion partner |
| 1539 | 0,0009938 | 0,0127 | 7301,16 | 3071,19 | 2,38 | [1418049_at](https://www.affymetrix.com/LinkServlet?probeset=1418049_at) | [Ltbp3](http://www.ncbi.nlm.nih.gov/entrez/query.fcgi?cmd=search&db=gene&term=Ltbp3) | latent transforming growth factor beta binding protein 3 |
| 1540 | 0,0009952 | 0,0127 | 2140,34 | 3145,08 | 0,68 | [1425149_a_at](https://www.affymetrix.com/LinkServlet?probeset=1425149_a_at) | [Pdcl](http://www.ncbi.nlm.nih.gov/entrez/query.fcgi?cmd=search&db=gene&term=Pdcl) | phosducin-like |
| 1541 | 0,0009953 | 0,0127 | 1646,82 | 805,28 | 2,05 | [1435737_a_at](https://www.affymetrix.com/LinkServlet?probeset=1435737_a_at) | [Nde1](http://www.ncbi.nlm.nih.gov/entrez/query.fcgi?cmd=search&db=gene&term=Nde1) | nuclear distribution gene E homolog 1 (A nidulans) |
| 1542 | 0,0009962 | 0,0127 | 3352,7 | 1743,51 | 1,92 | [1415985_at](https://www.affymetrix.com/LinkServlet?probeset=1415985_at) | [Sf3b3](http://www.ncbi.nlm.nih.gov/entrez/query.fcgi?cmd=search&db=gene&term=Sf3b3) | splicing factor 3b, subunit 3 |
| 1543 | 0,000997 | 0,0127 | 12,58 | 66,86 | 0,19 | [1418346_at](https://www.affymetrix.com/LinkServlet?probeset=1418346_at) | [Insl6](http://www.ncbi.nlm.nih.gov/entrez/query.fcgi?cmd=search&db=gene&term=Insl6) | insulin-like 6 |
| 1544 | 0,0009984 | 0,0127 | 452,35 | 228,81 | 1,98 | [1452724_at](https://www.affymetrix.com/LinkServlet?probeset=1452724_at) | [Ppp1r16a](http://www.ncbi.nlm.nih.gov/entrez/query.fcgi?cmd=search&db=gene&term=Ppp1r16a) | protein phosphatase 1, regulatory (inhibitor) subunit 16A |

**Table S1**
