# Supplementary figures and images for: Correction: In Vivo Tumorigenesis Was Observed after Injection of In Vitro Expanded Neural Crest Stem Cells Isolated from Adult Bone Marrow
Source: PLoS One. 2021 Sep 28;16(9):e0256477. doi: 10.1371/journal.pone.0256477 (PMC8478235; doi:10.1371/journal.pone.0256477)

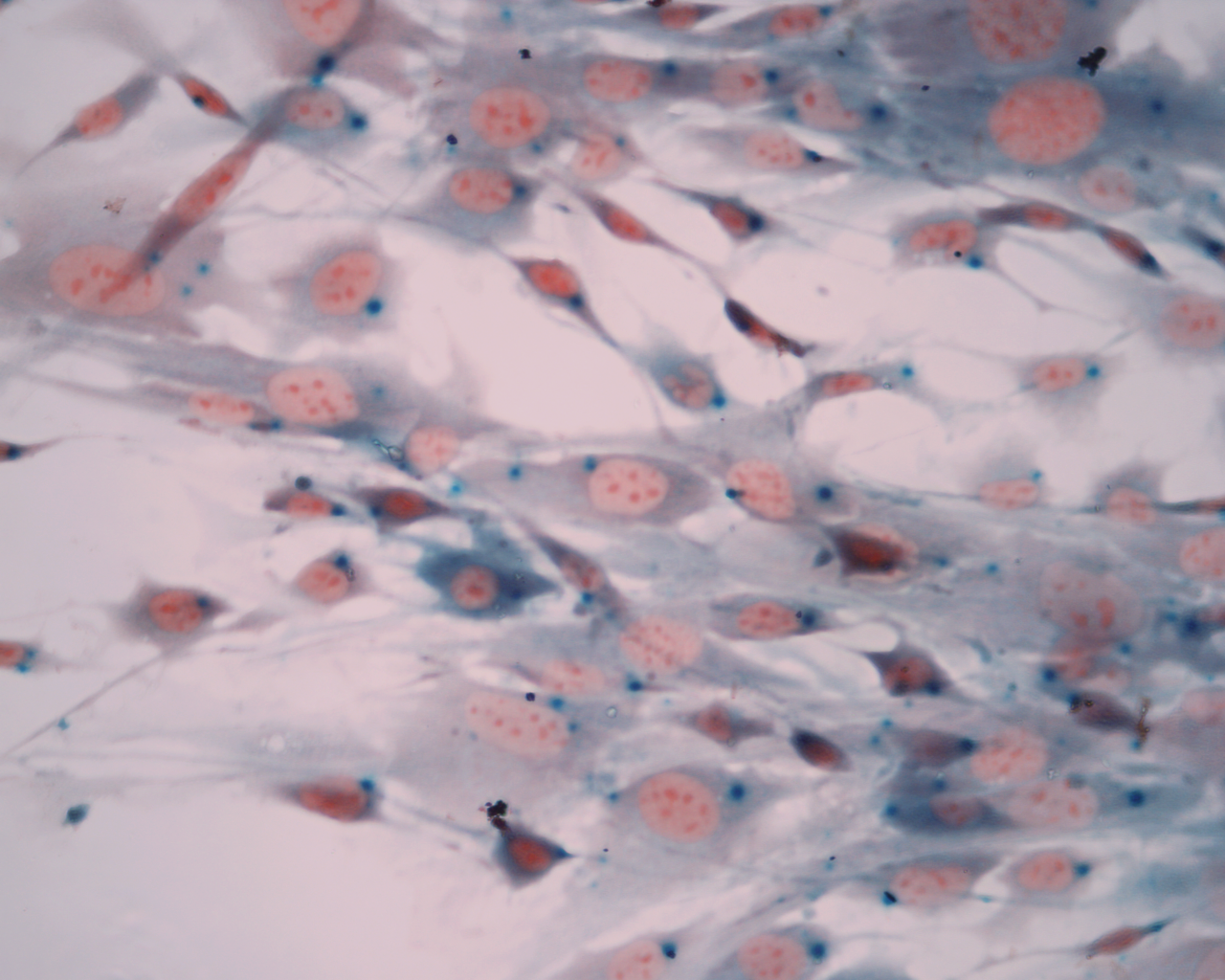

Supplement: S1 File — (ZIP) [file pone.0256477.s001.zip › Raw data Figure 1/Fig 1A.tif]

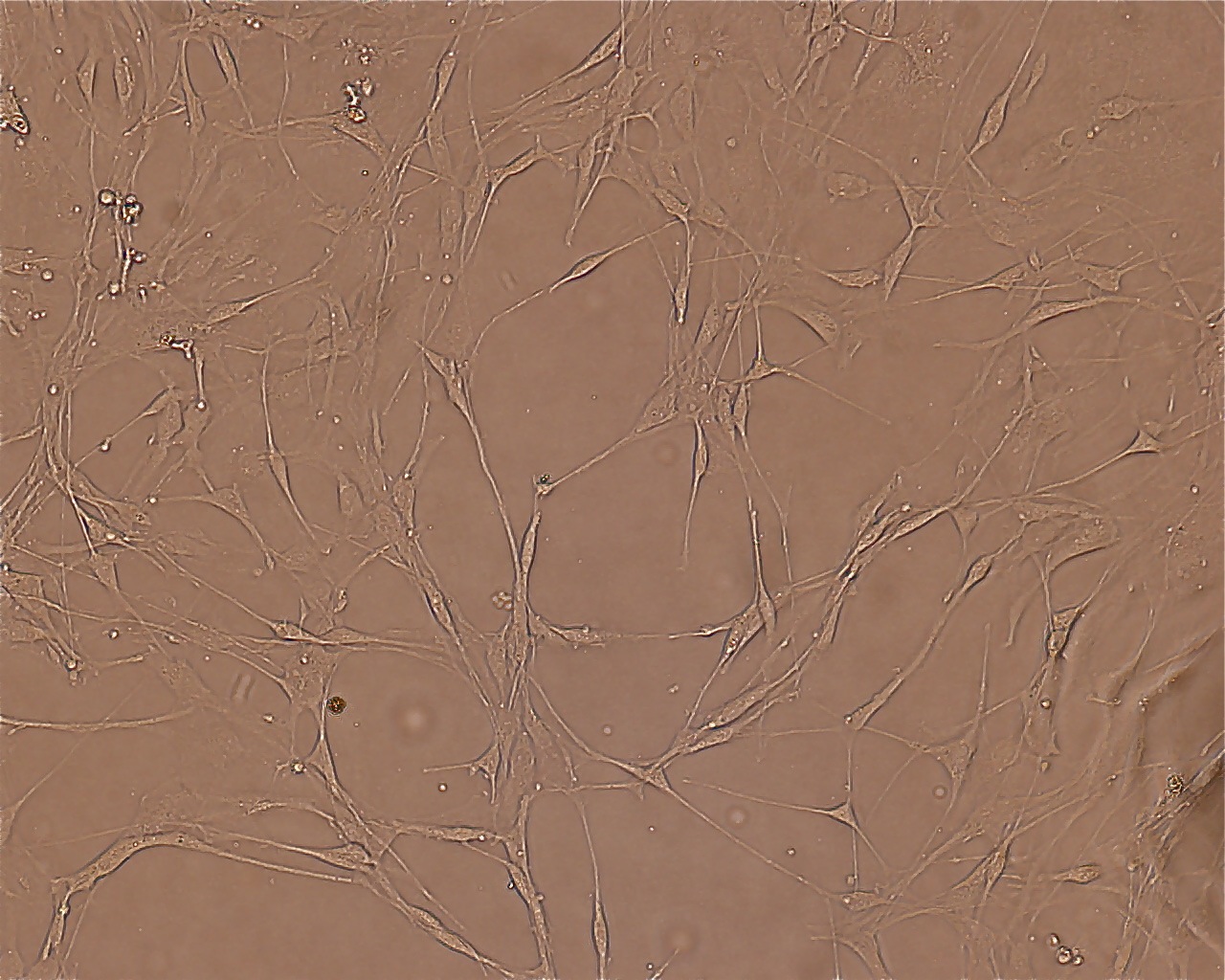

Supplement: S1 File — (ZIP) [file pone.0256477.s001.zip › Raw data Figure 1/Fig 1B.jpg]

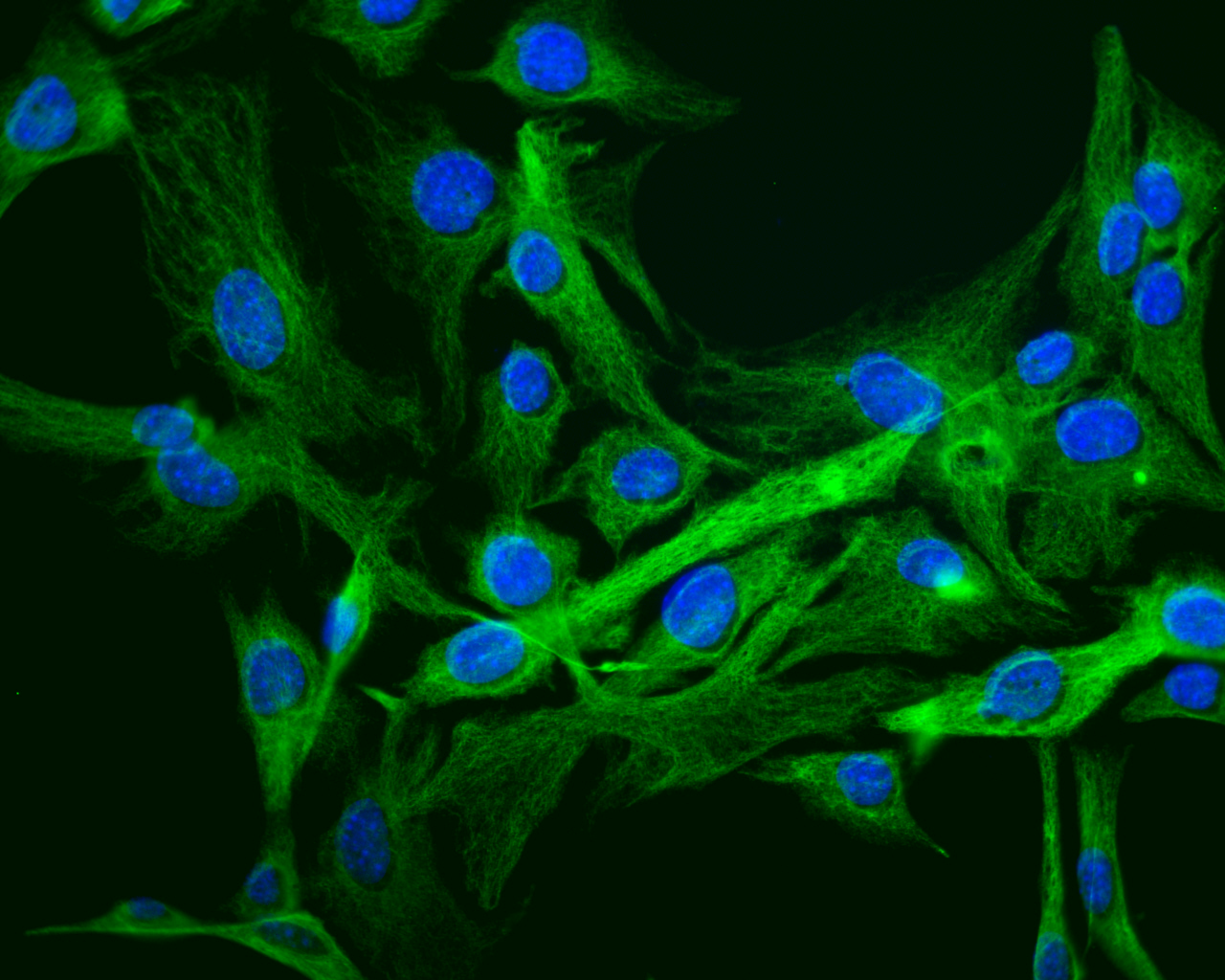

Supplement: S1 File — (ZIP) [file pone.0256477.s001.zip › Raw data Figure 1/Fig 1C.tif]

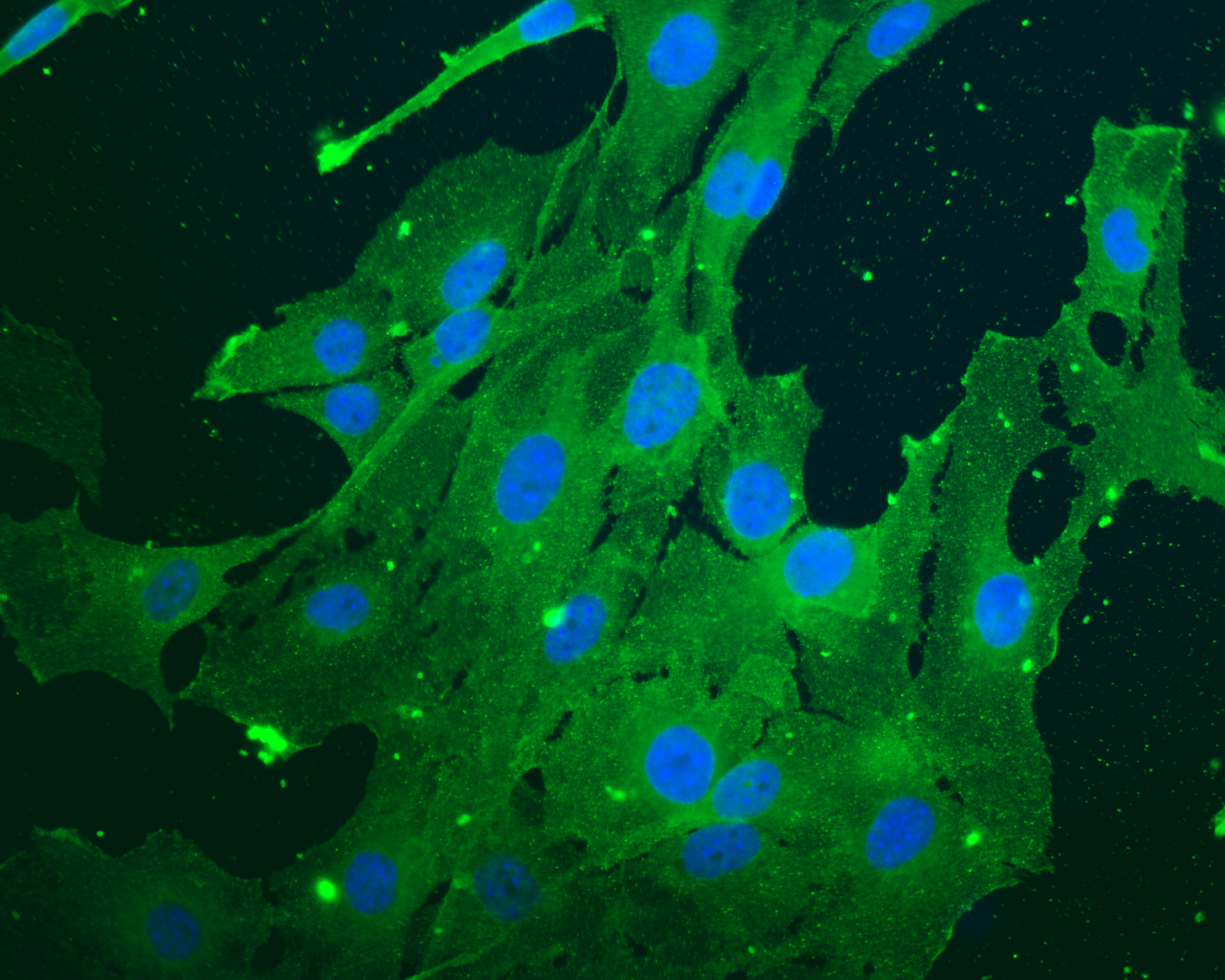

Supplement: S1 File — (ZIP) [file pone.0256477.s001.zip › Raw data Figure 1/Fig 1D.tif]

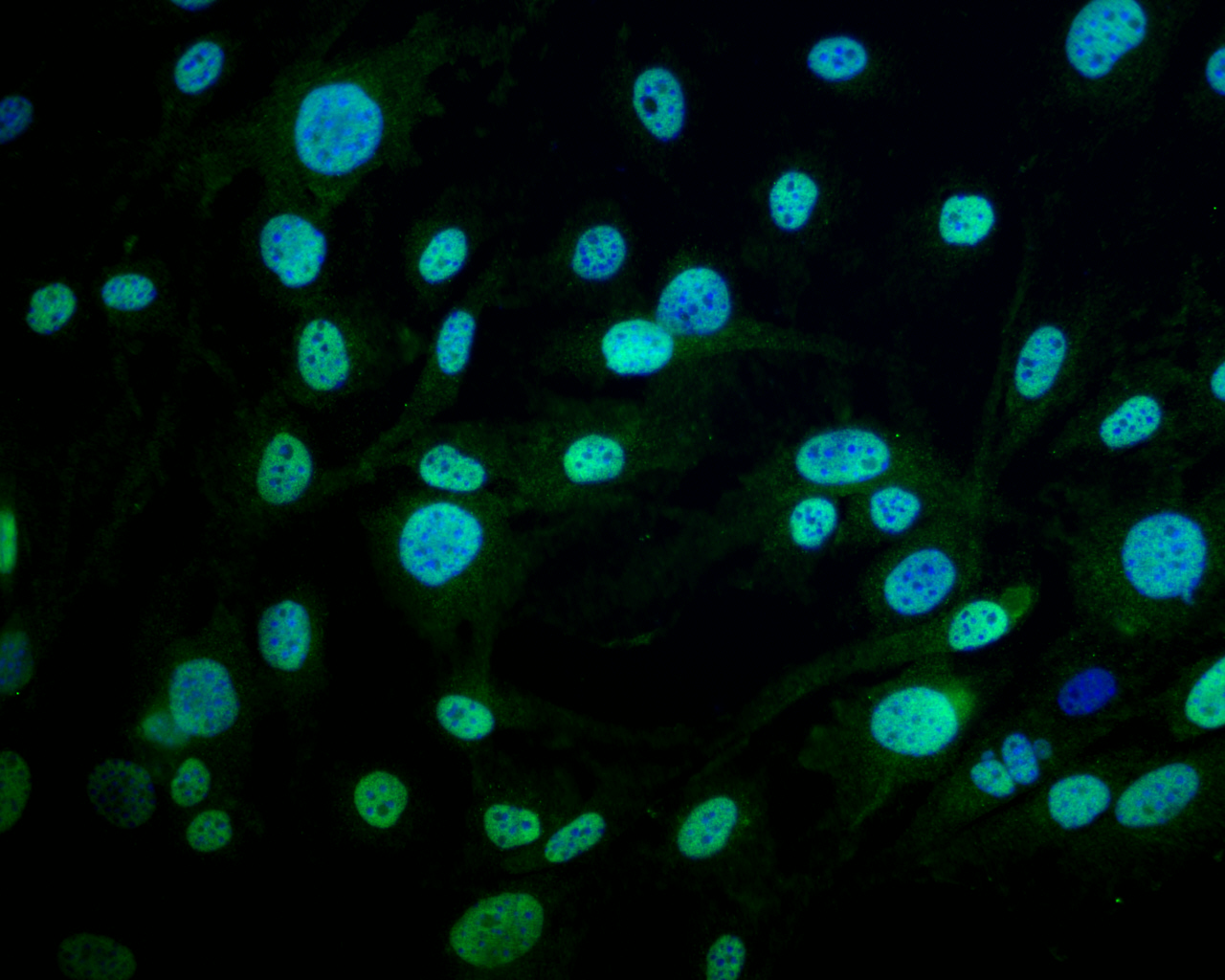

Supplement: S1 File — (ZIP) [file pone.0256477.s001.zip › Raw data Figure 1/Fig 1E.tif]

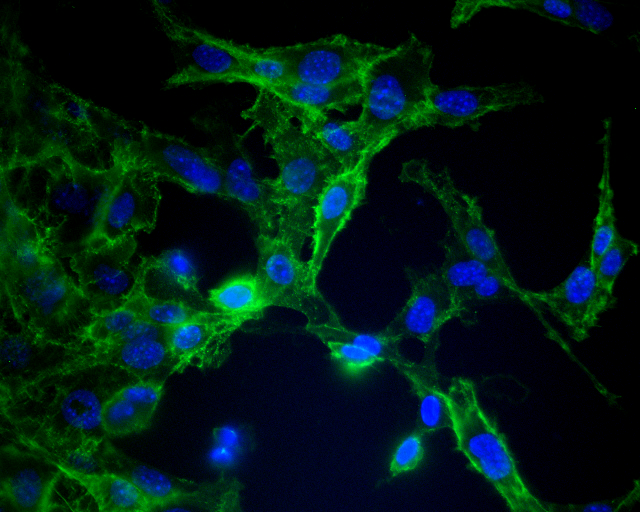

Supplement: S1 File — (ZIP) [file pone.0256477.s001.zip › Raw data Figure 1/Fig 1F.tif]

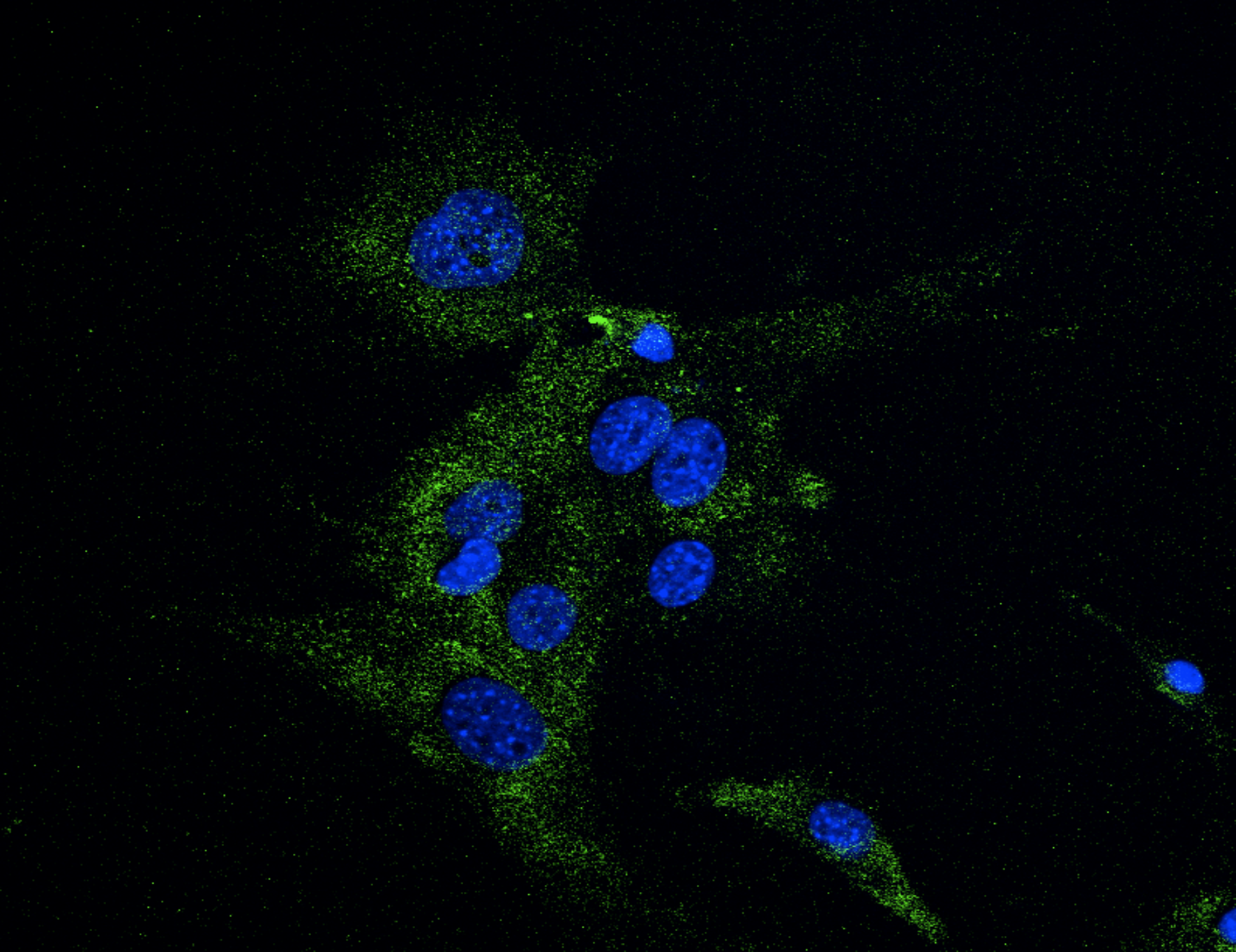

Supplement: S1 File — (ZIP) [file pone.0256477.s001.zip › Raw data Figure 1/Fig 1G.tif]

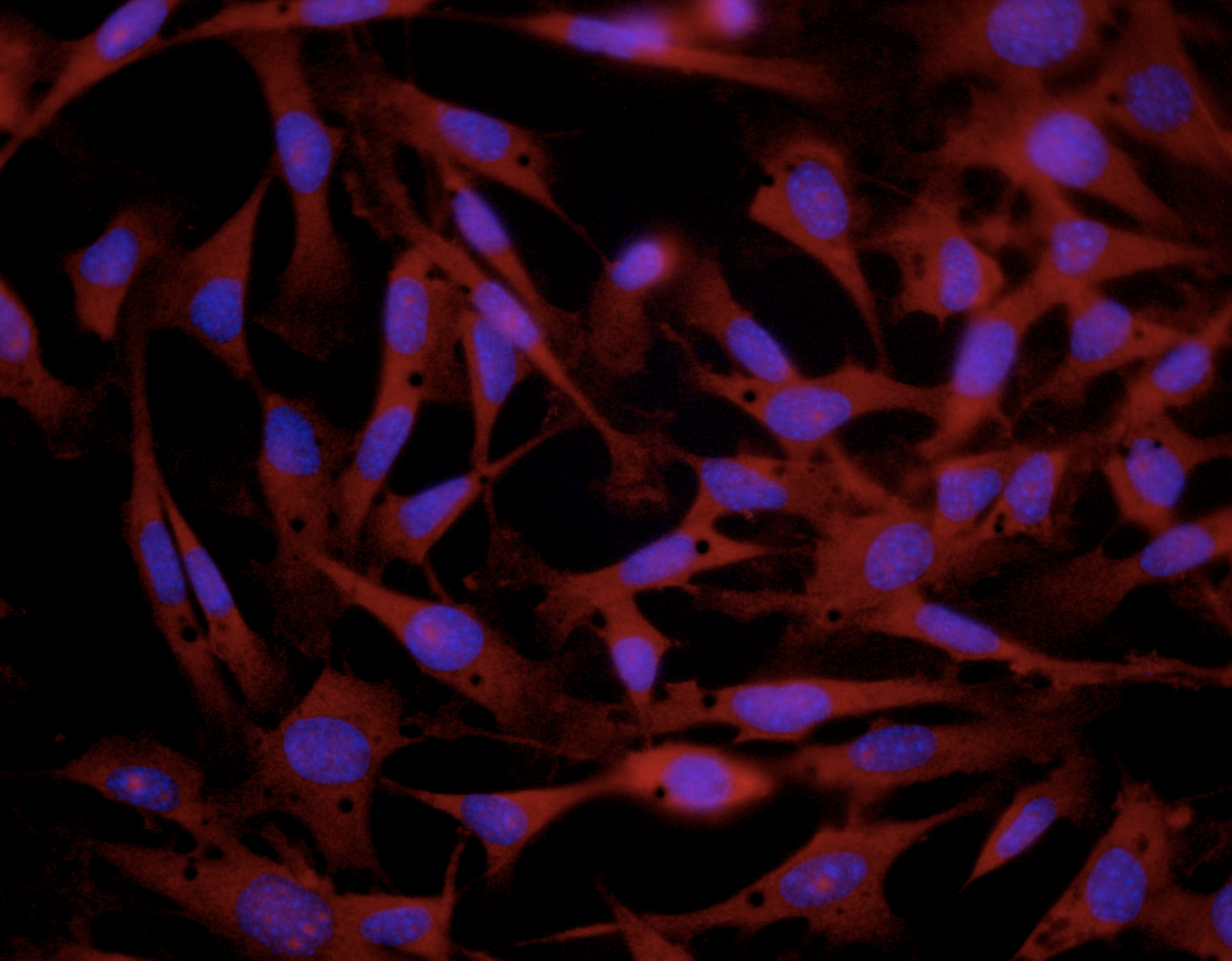

Supplement: S1 File — (ZIP) [file pone.0256477.s001.zip › Raw data Figure 1/Fig 1H.tif]

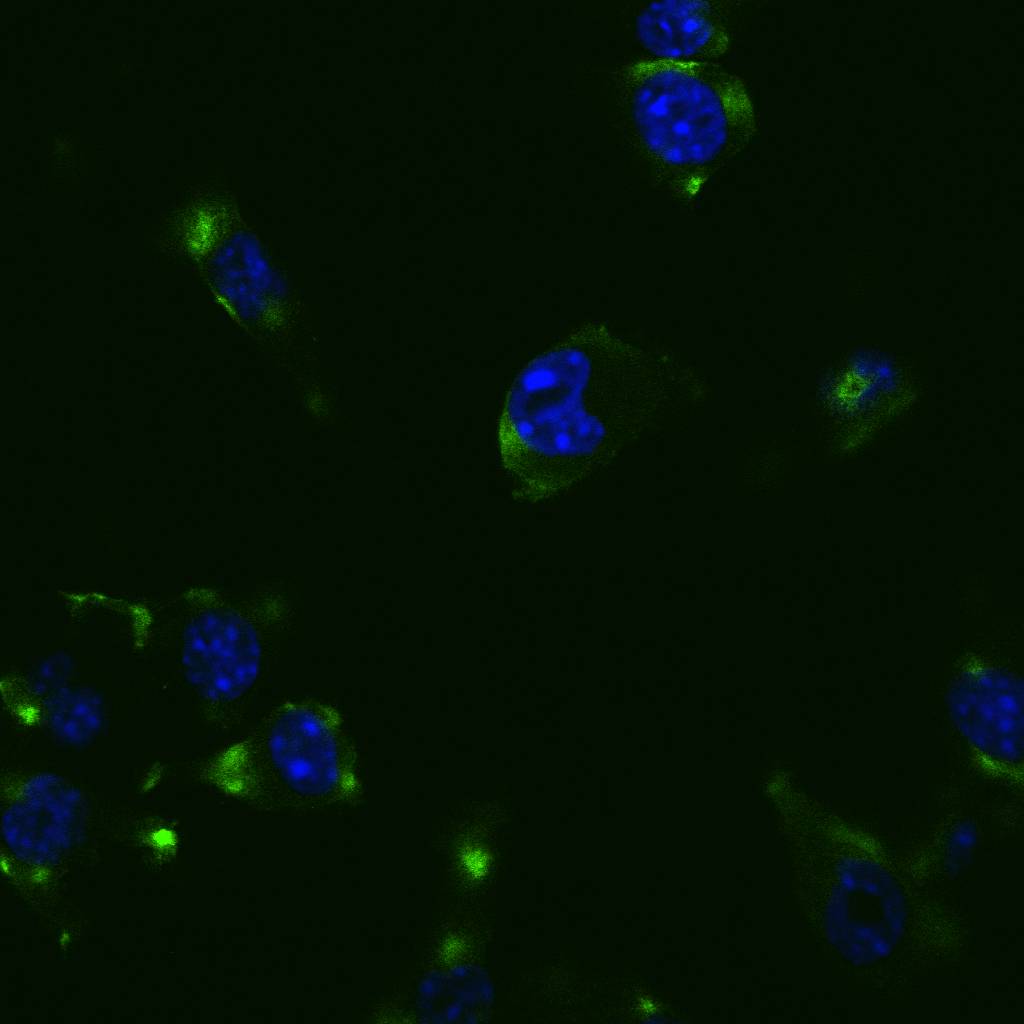

Supplement: S1 File — (ZIP) [file pone.0256477.s001.zip › Raw data Figure 1/Fig 1I.jpg]

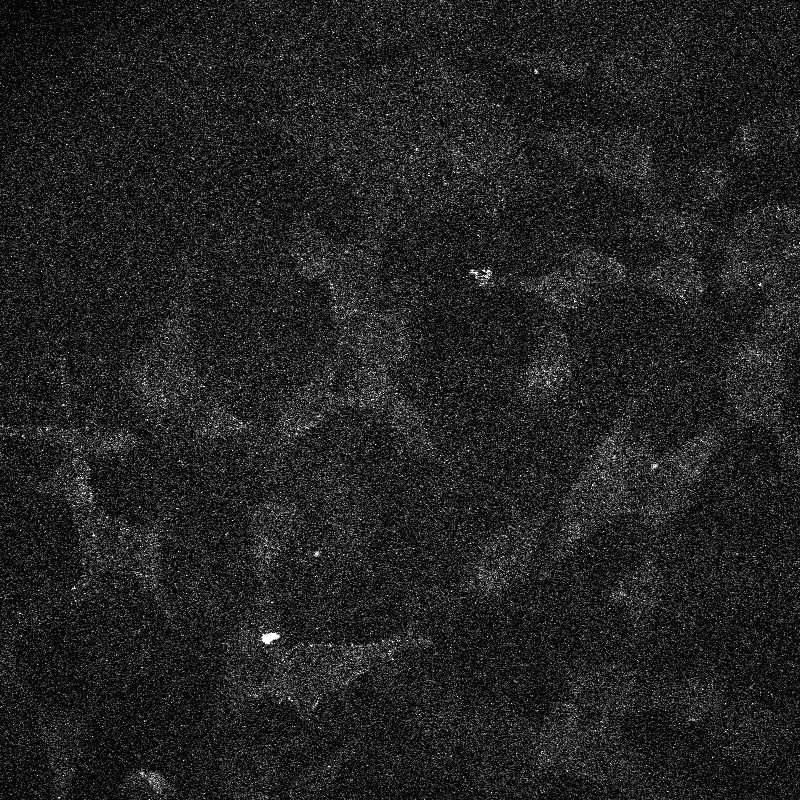

Supplement: S1 File — (ZIP) [file pone.0256477.s001.zip › Raw data Figure 1/Fig 1J.tif]

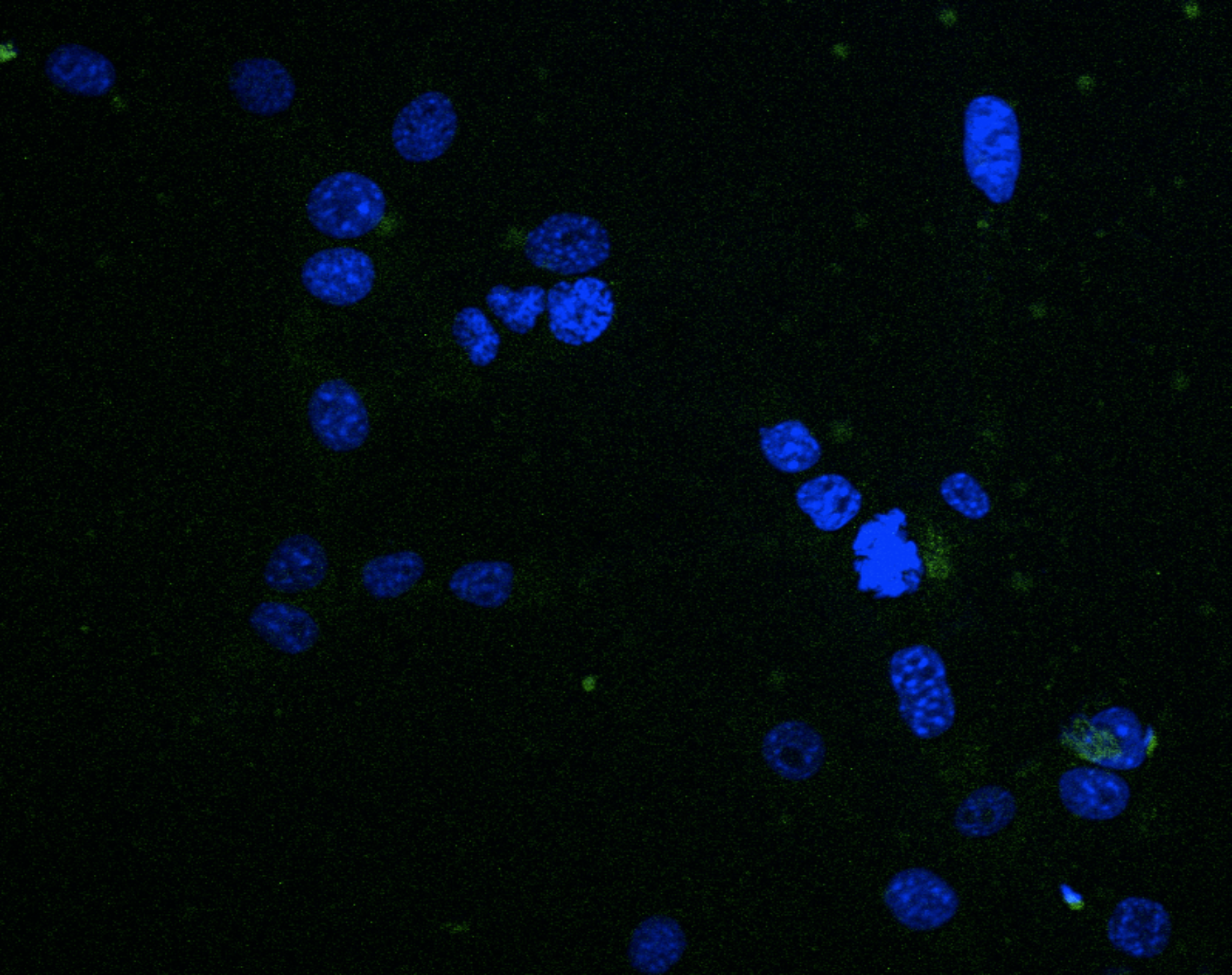

Supplement: S1 File — (ZIP) [file pone.0256477.s001.zip › Raw data Figure 1/Fig 1K.tif]

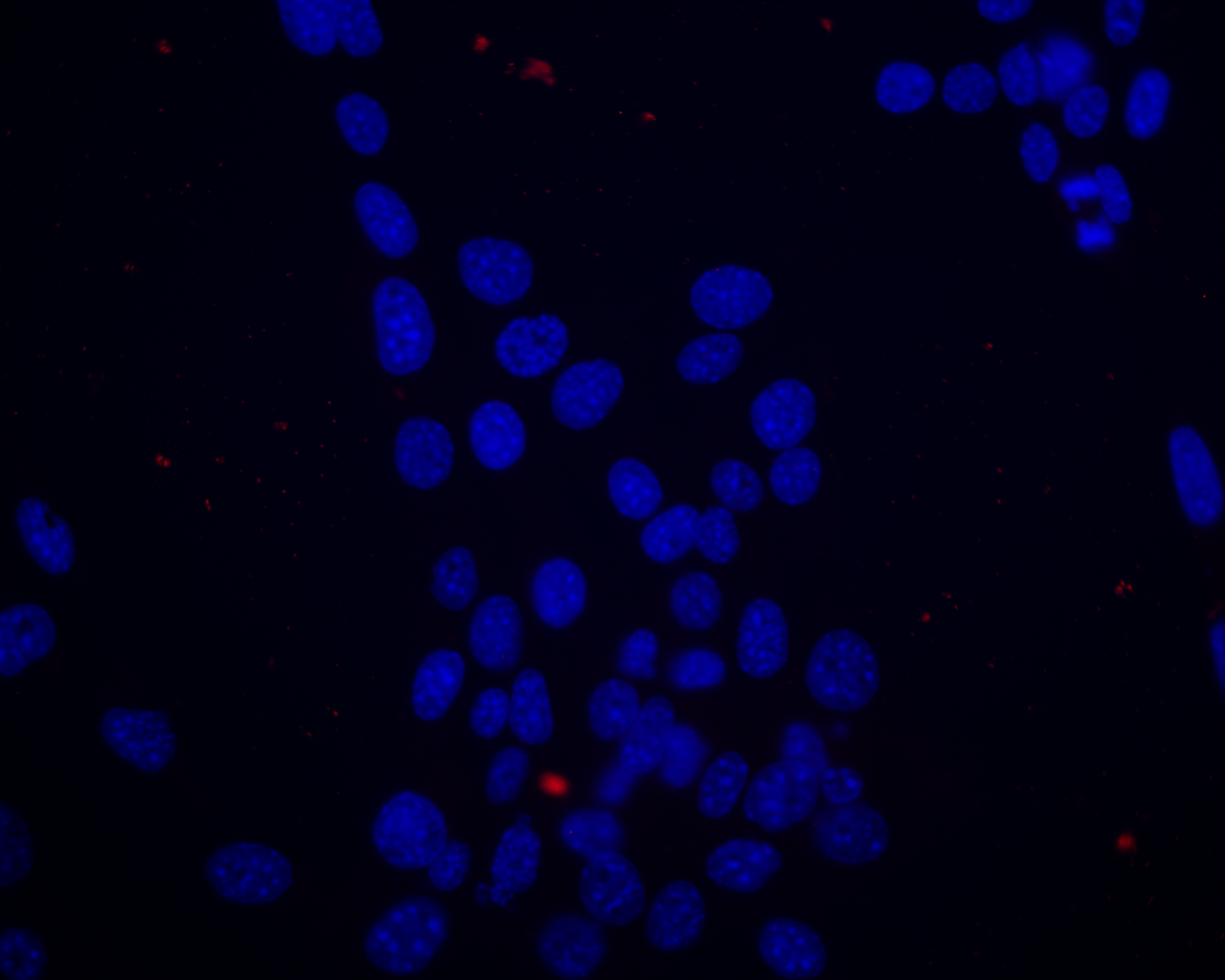

Supplement: S1 File — (ZIP) [file pone.0256477.s001.zip › Raw data Figure 1/Fig 1L.tif]

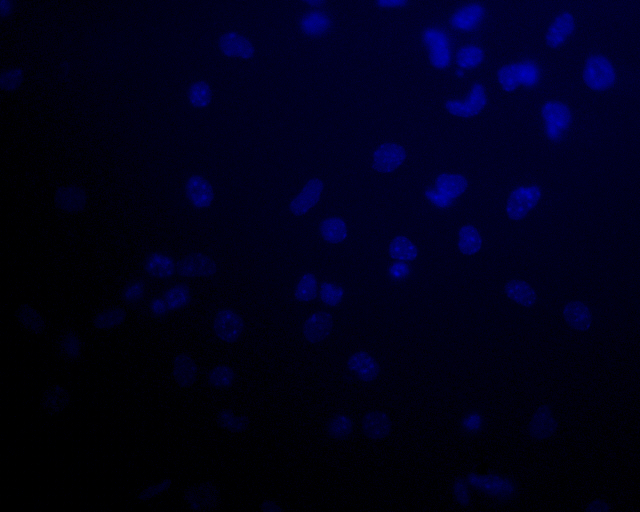

Supplement: S1 File — (ZIP) [file pone.0256477.s001.zip › Raw data Figure 1/Fig 1M Dapi.jpg]

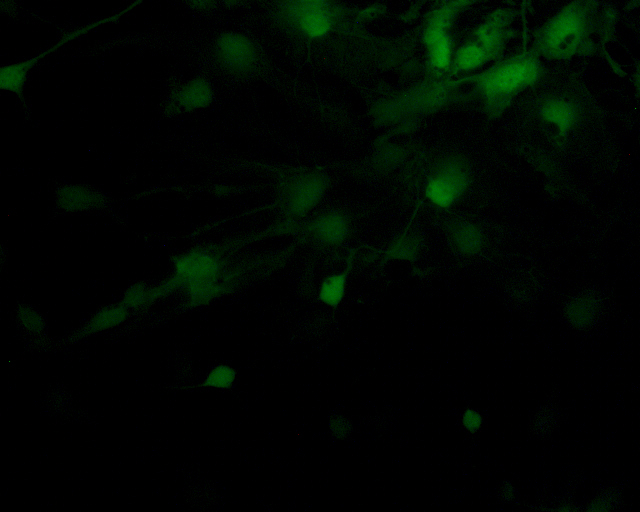

Supplement: S1 File — (ZIP) [file pone.0256477.s001.zip › Raw data Figure 1/Fig 1M GFP.jpg]

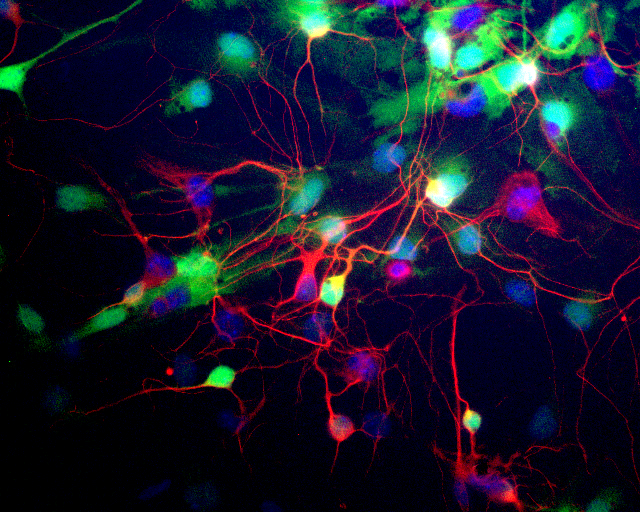

Supplement: S1 File — (ZIP) [file pone.0256477.s001.zip › Raw data Figure 1/Fig 1M MERGE.tif]

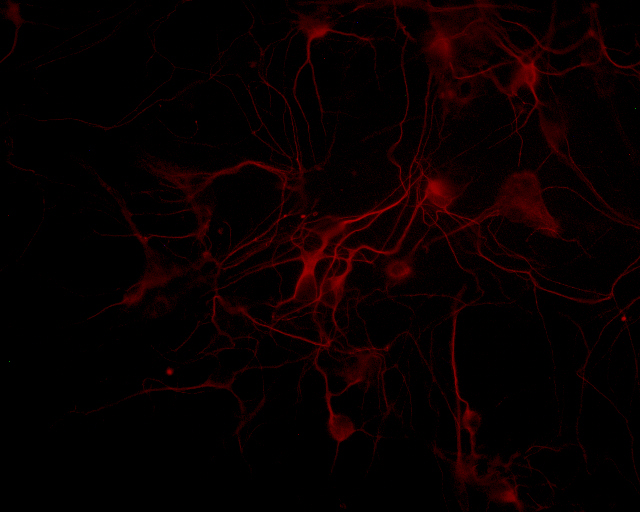

Supplement: S1 File — (ZIP) [file pone.0256477.s001.zip › Raw data Figure 1/Fig 1M Tuj1.jpg]
